# Antioxidative Potency of Dolphin Serum Albumin Is Stronger Than That of Human Serum Albumin Irrespective of Substitution of ^34^Cysteine With Serine

**DOI:** 10.3389/fphys.2020.598451

**Published:** 2020-11-02

**Authors:** Miwa Suzuki, Makoto Anraku, Wataru Hakamata, Takushi Kishida, Keiichi Ueda, Tomoko Endoh

**Affiliations:** ^1^Laboratory of Aquatic Animal Physiology, Department of Marine Science and Resources, College of Bioresource Sciences, Nihon University, Fujisawa, Japan; ^2^Laboratory of Physical Pharmaceutics, Faculty of Pharmaceutical Sciences, Sojo University, Kumamoto, Japan; ^3^Laboratory of Biological Chemistry, Department of Chemistry and Life Science, College of Bioresource Sciences, Nihon University, Fujisawa, Japan; ^4^Wildlife Research Center, Kyoto University, Kyoto, Japan; ^5^Museum of Natural and Environmental History, Shizuoka, Japan; ^6^Okinawa Churashima Foundation, Motobu, Japan; ^7^Shinagawa Aquarium, Shinagawa, Japan

**Keywords:** dolphin, serum albumin, antioxidant, oxidative stress, amino acid residues

## Abstract

Serum albumin (SA), the most abundant protein in circulation, functions as a carrier protein, osmoregulator, and antioxidant. Generally, SA exerts its antioxidative effects by scavenging reactive oxygen species. Because marine mammals are superior divers, they are intermittently exposed to oxidative stress induced by rapid reperfusion of oxygen to ischemic tissues after the dive. Although several antioxidants in marine mammals have been described, SA activity remains largely uncharacterized. In this study, we investigated the antioxidative activity of SA in marine mammals by comparing features of the primary and steric structures, biochemical properties, and antioxidative activities of common bottlenose dolphin SA (DSA) and human SA (HSA). Our results revealed that DSA lacked free cysteine at position 34 that is important for the antioxidative activity of HSA; however, the antioxidative capacity and thiol activity of DSA were stronger than those of HSA. Circular dichroism spectra showed different patterns in DSA and HSA. Ultraviolet fluorescence intensities of DSA were higher than those of HSA, suggesting lower surface hydrophobicity of DSA. Additionally, DSA showed higher excess heat capacity than HSA. We then compared a homology model of DSA with a 3D model of HSA. Our results indicate that DSA was more unstable than HSA at least in the body-temperature range, probably due to the mode of molecules involved in the disulfide bonds and/or the lower surface hydrophobicity, and it may be related to the equivalent or stronger antioxidant potency of DSA. These data show that DSA is an effective antioxidant in the circulation of the dolphin.

## Introduction

Marine mammals habitually dive to feed, avoid predators, and rest (Thompson et al., 1991; Boyd, 1997; Heithaus and Frid, 2003; Sparling et al., 2007). During diving, they undergo cardiovascular adjustments for oxygen consumption. These include bradycardia and peripheral vasoconstriction, which lead to a blood shift to prioritize the flow of blood into the brain and heart (Kooyman and Ponganis, 1998; Ponganis, 2011). Restriction of blood flow leads to ischemia of peripheral tissues, while reperfusion with tachycardia after diving induces production of reactive oxygen species (ROS) in these tissues (Vázquez-Medina et al., 2007; Zenteno-Savin et al., 2012; Chouchani et al., 2014) Generally, antioxidants counteract oxidative stress (Pisoschi and Pop, 2015). In cetacean deep/long divers (*Kogia* spp.), oxidative stress in tissues are more severe than in cetacean shallow/short divers (*Tursiops truncatus*) (Cantú-Medellín et al., 2011); this shows that diving behavior may generate oxidative stress in marine mammals.

Albumin is the most abundant serum protein in mammals, and serum albumin (SA) is found at concentrations of 30–55 mg/mL in the bottlenose dolphin (Dierauf and Gulland, 2001; Fair et al., 2006; Hall et al., 2007) of which range is almost same as those in human (Peters, 1995) or in rat (Khasanah et al., 2015). As shown in human and rodents, SA is expressed as preproalbumin mainly in the liver. The signal peptide, containing 18 residues, is cleaved in rough endoplasmic reticulum, released as proalbumin, and translocated to Golgi body, where the N-terminal six residues of proalbumin are further cleaved. The product is then secreted into circulation as albumin (Strauss et al., 1977; Fanali et al., 2012). As a carrier protein, SA performs various functions using its exceptional ligand-binding capacity (Fasano et al., 2005). SA acts as an antioxidant by trapping radicals, carrying radical scavengers, or sequestering transition metal ions possessing pro-oxidative activity. More than 50% of the total antioxidative activity of plasma is due to SA (Roche et al., 2008; Taverna et al., 2013).

Relationships between protein structure and function have been studied in detail for human SA (HSA) (Fanali et al., 2012). The cysteine residue at position 34 (^34^Cys) of HSA does not participate in intramolecular disulfide bonds. The thiol group in ^34^Cys constitutes the largest portion of free thiol in the blood and is essential for antioxidative activity of albumin and its ability to scavenge radicals (Fanali et al., 2012). Replacing ^34^Cys with serine (Ser) impairs antioxidative activity of SA and renders SA easily degradable by hydrogen peroxide (Anraku et al., 2015). Methionine (Met) also contributes to antioxidative activity. The three Met residues of HSA at positions 123, 298, and 329 can prevent free radical formation by chelate-bonding to metals, while the Met residues at positions 87, 446, and 548 bind to oxidants. These Met and Cys residues account for 40–80% of the total antioxidative activity of HSA (Bourdon et al., 2005).

In marine divers, such as cetaceans, SA may help counteract the cellular damage caused by ROS after reperfusion. However, previous studies have focused mainly on antioxidative enzymes (catalase, glutathione reductase, glutathione peroxidase, glutathione S-transferase, and superoxide dismutase), glutathione, and hypoxia-inducible factor 1α (HIF1α) in tissues and cells (Elsner et al., 1998; Wilhelm Filho et al., 2002; Johnson et al., 2005; Vázquez-Medina et al., 2007; Cantú-Medellín et al., 2011; Zenteno-Savin et al., 2012). Thus far, the antioxidative properties of SA in marine mammals remain largely uncharacterized.

In the blood of white whales and bottlenose dolphins, oxygen partial pressure (PO_2_) is decreased to one-third and one-fourth, respectively, of its initial value during submergence (Shaffer et al., 1997; Williams et al., 1999). Ischemia and reperfusion after surfacing may induce ROS generation in blood and tissue, as well as in circulation where ROS are generated by autoxidation of hemoglobin (Abugo and Rifkind, 1994). Concentrations of oxidative-stress biomarkers and antioxidative activity are higher in the blood of *Mirounga leonina*, a deep-diving seal, than in the blood of *Arctocephalus australis*, a moderate-diving seal (Righetti et al., 2014). This suggests that circulating antioxidants are necessary for counteracting the high production of ROS induced by extended diving time. The studies by Righetti et al. (2014) and García-Castañeda et al. (2017) show higher reduced-form glutathione concentrations in the erythrocytes of marine mammals than in those of semi-aquatic or terrestrial mammals. These studies, performed in pinnipeds, focused on glutathione and antioxidative enzymes. To investigate the antioxidative potential of blood in cetaceans, we examined the antioxidative characteristics of SA in the common bottlenose dolphin (DSA). We then compared these characteristics with those of human SA (HSA), the properties of which have been extensively characterized.

## Materials and Methods

### Comparing the 34th Amino Acid Residue of SA Among Mammals

To compare the inter-species differences around the 34th amino acid residue of SA among mammals, the protein sequences of broad taxa of aquatic and terrestrial mammals were retrieved from the NCBI database^1^ as listed in Supplementary Table S1. SA sequences for some of these species were identified using genome assembly because SA-sequence information in public databases is scant (Supplementary Table S1). In such cases, the albumin exon 3 sequence (containing the 34th amino acid residue) for each species was searched against the conspecific genome assembly using TBLASTN program in the BLAST + v2.6.0 package (Stephen, 1997) with a cutoff *E*-value of 1e-19 (for cetartyodactyls) or 1e-10 (for non-cetartiodactyls). The deduced amino acid sequence for exon 3 of the common bottlenose dolphin albumin gene (accession no. XP_004322082.2) was used as query.

### Purification of SA

#### Experimental Ethics

All experiments in this study were performed in accordance with the guidelines for animal experiments in the College of Bioresource Sciences, Nihon University (Approved No. AP18BRS067-1). Dolphins in the aquarium were treated properly during all experiments under the supervision of veterinarians.

#### Serum Collection

Blood samples were collected during routine health check from 2 male dolphins housed in the Shinagawa Aquarium, Tokyo, Japan, for N-terminus amino acid sequencing of DSA and from 5 common bottlenose dolphins (two males and three females) housed in the Okinawa Expo Park, Okinawa, Japan, for other analyses. These blood samples were immediately centrifuged at 3,000 × *g* for 10 min, frozen in liquid nitrogen, and maintained at −80°C until analysis. The human sera used for control were commercially obtained (*n* = 3; lot numbers: SLBX-0342, SLBX-0344, and SLBX-6020; H4522-20ML; Sigma-Aldrich, St. Louis, MO, United States).

#### Albumin Extraction From Sera

Serum albumin was extracted from the sera using a Pierce^TM^ Albumin Depletion Kit (Thermo Scientific, MA, United States) followed by purification using Amicon^®^ Ultra-15 mL 50K (Millipore Sigma, MA, United States) in accordance with the manufacturer’s instructions. Briefly, serum, mixed with an equal volume of bind and wash buffer, was applied to the resin settled in a spin column, and the column was centrifuged at 12,000 × *g* for 1 min. The resin containing the absorbed SA was washed using the bind and wash buffer to remove extraneous substances. Then, elution buffer (20 mM sodium phosphate, 250 mM sodium thiocyanate, pH 7.2) was applied to the resin, and centrifugation was performed at 12,000 × *g* for 1 min to elute the SA. This elution process was repeated four times for thorough elution of SA. The eluted fluid was applied onto a 50 K filter device and centrifuged at 14,000 × *g* for 10 min to filter out proteins smaller than 50 kDa and to concentrate the desired protein. Then, 4 mL 0.1 M phosphate buffer (PB) (pH 7.4) was applied onto the filter and centrifuged at 14,000 × *g* for 10 min to replace the buffer. The retained SA solution was retrieved, and protein concentration in the solution was measured using Bradford protein assay.

#### SA Delipidation

For characterization of SA structure, lipids binding to the extracted SA were removed by delipidation according to the method developed by Chen (1967). Briefly, a half weight of activated charcoal was added to the extracted SA solution and vortexed to mix. Subsequently, pH was lowered to 3.0 by the addition of 0.3 N HCl and incubated for 1 h at 4°C. Centrifugation was carried out at 20,000 × *g* and 2°C for 20 min; the resultant supernatant was collected into a new tube. After an additional centrifugation under the same conditions, the supernatant was filtered through a 0.45 μm filter to completely remove the active charcoal. Then, 0.3 N NaOH was added to the filtered SA solution to adjust the pH to 7.0. The filtered SA was freeze-dried and used for experiments after rehydration with 67 mM PB.

### Characterizing the Structures of DSA

#### Circular Dichroism Spectrum Test

To detect differences in secondary protein structure of HSA and DSA, the measurements were done using a Jasco J-720 type spectropolarimeter (Jasco Co., Tokyo, Japan) at 25°C. Far-UV and near-UV spectra were recorded at protein concentrations of 5 μM in 67 mM PB. Measurements were conducted at the wavelength of 250–200 or 250–350 nm, scanning speed of 10 nm/min, sensitivity of 500 mdeg or 100 nm, time constant of 8 s, and step solution of 0.5 nm. Mean residue ellipticity ([θ]) was calculated using the following formula:

[θ]=θ/10⁢cl

in which θ is molecular ellipticity, l is cell length, and c indicates mean residue molecular concentration. Mean residue molecular concentration (c) was calculated using the following formula:

c=nCp

in which n is number of amino acid residues of the protein and Cp is molecular concentration.

#### Surface Hydrophobicity Assay

A sensitive indicator of protein folding, 1-anilinonaphthalene-8-sulfonic acid (ANS), becomes fluorescent when binding to hydrophobic regions on protein surfaces. Therefore, the hydrophobicity of DSA was compared with that of HSA using ANS. For this, 2 μM SA in 67 mM PB was mixed with 60 μM ANS from 10 mM ANS stock solution in EtOH, and vortexed, after which fluorescence characteristics were immediately evaluated at the Ex/Em = 350/400–500 nm, and band widths were evaluated at the Ex/Em of 5 or 10 mm and at 25°C.

#### Ultraviolet Fluorescent Assay

Ultraviolet fluorescence emitted from aromatic amino acids, especially tryptophan (Trp), was detected by monitoring the fluorescence spectrum of the protein. SA was prepared at the concentration of 2 μM in 67 mM PB. The fluorescence of each SA was measured at Ex/Em = 295/320–400 nm, and bandwidths were evaluated at the Ex/Em of 5 or 10 nm using a fluoro-spectrophotometer (F-2500, Hitachi High-Tech, Minato, Tokyo, Japan).

#### Differential Scanning Calorimetric Assay

Next, we evaluated the thermal stability of SA using a differential scanning calorimetric assay on a Nano DSC differential scanning calorimeter (TA Instruments, New Castle, DE, United States). For this, we used 20 μM SA, prepared in 67 mM PB (pH 7.4), with scanning speed set at 1 K/min. The reversibility of thermal denaturation was evaluated by re-heating the cooled SA solution that resulted from the first scanning assessment. The thermogram obtained by the first assessment was fitted with a non-linear fitting algorithm using OriginLab scientific plotting software. The resultant excess heat capacity curves were analyzed to compare DSA and HSA.

#### Sequencing the N-terminus of DSA

To certify the primary structure of circulating SA in the dolphin, amino acid sequencing of the N-terminus of DSA was performed using sera from the two dolphins in Shinagawa Aquarium. After performing the albumin extraction using the method described in subsection above, the extract was analyzed using SDS-PAGE on a 10% polyacrylamide gel (SuperSepTM Ace, FUJIFILM-Wako), followed by transfer to a PVDF membrane using a Trans-blot TurboTM Transfer System (Bio-Rad Laboratories, Hercules, CA, United States). The membrane was washed with 50% methanol and Milli-Q water, and the section at around 66 kDa was excised. Sequencing of the initial five amino acids at the N-terminus was outsourced to the Institute for Protein Research in Osaka University (Suita, Osaka, Japan).

### Biochemical Assessment of HSA and DSA

#### Radical Scavenging Ability of HSA and DSA

Antioxidant activities of DSA and HSA were determined using 1,1-diphenyl-2-picrylhydrazyl (DPPH), a stable free radical, according to the method of Sassa et al. (1990). For this, concentration-dependent DSA and HSA was added to a DPPH solution (0.25 mM DPPH in ethanol and 67 mM PB [pH 7.4], at a ratio of 2:1) and incubated for 20 min at 25°C. At 0 and 20 min, absorbance was measured at 540 nm using a spectrophotometer, and DPPH-scavenging activities of DSA and HSA were calculated as a reduction in the absorbance. For comparison purpose, trolox was used as reference. Radical scavenging ability was calculated using the following formula after reading the absorbance at 540 nm.

$$\%\,\mathrm{Radical}\,\mathrm{scavenging}\,\mathrm{ability}=\left[\left({\text{Abs}}_{\text{control}}-{\text{Abs}}_{\text{sample}}\right)/{\text{Abs}}_{\text{control}}\right]\times 100$$

Antioxidant activities of DSA and HSA were also determined using 2,2′-azino-bis(3-ethylbenzothiazoline-6-sulfonic acid) (ATBS^•+^) assay as described by Huang et al. (2011). The ABTS^•+^ radical (7 mM) and potassium persulfate (2.45 mM) were dissolved in water to a final concentration and left for 16 h in the dark at room temperature. For comparison purpose, trolox was also used as reference. Twenty μL of each sample was mixed with 180 μL ABTS^•+^ solution. Sixty min after addition of sample, absorbances were read at 734 nm. Radical scavenging ability was calculated using the above formula with the absorbance.

In addition, total antioxidant capacity of DSA and HSA was measured using PAO kit (JaICA, Fukuroi City, Japan), according to the method of Tomida et al. (2009). PAO kit detects both hydrophilic and hydrophobic antioxidants in the solution. In principal, the kit estimates the copper reducing power by the antioxidants present in the serum. Uric acid was used as standard in the kit and uric acid equivalents of copper reducing power was calculated using the formula, 1 mM uric acid = μmol/L of copper reducing power.

#### Free Thiol Activity Assay for Whole Serum and SA

To investigate the antioxidative capacity of SA and whole serum, a free thiol assay was carried out using a Thiol Quantification Assay Kit (Fluorometric) (Abcam; Tirana, Albania) according to the manufacturer’s instructions. In brief, 50 μL whole serum/0.625 μM extracted albumin or GSH standard solution was applied to the wells of a 96-well plate in duplication. A GSH reaction mixture containing Thiol Green Indicator and assay buffer was added to the wells and allowed to incubate with gentle shaking for 60 min at 25°C under dark conditions. The fluorescence (Ex/Em = 490/520 nm) of each well was measured by a fluorometer (Infinite 200 Pro Tecan; Männedorf, Switzerland), while thiol activity of the assayed solution was calculated as a cysteine dose (μM).

#### Degradation of HSA and DSA by Hydrogen Peroxide

To evaluate the degradation of SA by hydrogen peroxide (H_2_O_2_), an oxidation assay was carried out according to the method of Anraku et al. (2015). Briefly, 2 mL 0.625 μM dolphin or human albumin was dissolved in phosphate buffer. H_2_O_2_ was added to the solution at the final concentration of 25 mM, and the resulting solution was incubated for 60 min at 25°C. The controls were as follows: (1) SA incubated without H_2_O_2_ for 6 h and (2) SA not incubated with H_2_O_2_ (0 h). Then, the reaction mixture was added to 4 × Laemmili sample buffer (BioRad Laboratories, CA, United States) containing a final volume of 10% dithiothreitol, and the resulting mixture was heated for 5 min at 100°C. The mixture was separated by SDS-polyacrylamide gel electrophoresis and visualized using CBB staining. The intensity of albumin bands was analyzed using band analysis tool in ImageJ (Wayne Rasband, NIH, United States).

### 3D Structure Analysis

Next, we constructed a 3D structure-homology model of DSA in order to determine the characteristics contributing to the antioxidative activity of dolphin albumin. To construct a 3D-model for the SA of the common bottlenose dolphin, we initially searched for the most appropriate template of a 3D homology model (HM) using Swiss-model^2^. The SA of Bos taurus (PDB ID: 4f5s chain A) was utilized because of the same number of amino acids (583 residues), high homology of the primary sequence (83.19%), and adequate resolution (2.47 Å). The resulting HM, constructed using the primary sequence of bottlenose dolphin albumin (NCBI accession no. XP_004322082.2) and bovine SA as template, showed no errors in its structure. Details in DSA-HM are shown in Supplementary text data_DSA. To understand the oxidative-reductive characteristics of DSA, an energy minimization calculation was performed for the dihedral angles of all side chains in DSA-HM using Molegro Virtual Docker (Ver. 7.0)^3^. The structure of the main chain was unchanged by the calculation. A conformational alignment of the constructed DSA-HM with HSA (PDB ID: 1BJ5) was performed using Molegro Virtual Docker. In order to investigate the characteristics of cysteine residues involved in the SS bonds of each SA, the distance between sulfur atoms, and angle between the sulfur atoms forming the SS bond and each β-carbon atom, were measured using 3D models of the SAs.

### Statistical Analysis

Differences in radical-scavenging ability and averages of thiol activities were compared between DSA and HSA using Student’s *t*-test. The reduction rate of band volumes relative to those of the controls was calculated based on band intensities, and averages of reduction rates were compared using Student’ *t*-test. Equalities of variances for distances between sulfur atoms and angles between the sulfur atoms forming the SS bond and each β-carbon atom were compared between DSA than in HSA by *F*-test.

## Results

### ^34^Cys of SA Is Broadly Replaced With Ser in the Cetacean Linage

To characterize cetacean SA, an alignment of albumin sequences among various mammalian species was performed *in silico*. The comparison revealed that ^34^Cys of SA, which is a critically important residue for the antioxidative activity of HSA, was replaced with Ser in nearly all the sequenced cetacean species with the exception of Yangtze River dolphin (Table 1). In the SA of marine mammals, including the walrus and the West-Indian manatee, and in the SA of the hippopotamus and the elephant, we observed the same replacement (C34S).

**TABLE 1:** Alignment of serum albumin at positions 32–36.

		Species	Position of the residue in SA				
			
			32	33	34	35	36
Laurosiatheria	Cetacea	Common bottlenose dolphin	Q	Q	S	P	F
		Killer whale	Q	Q	S	P	F
		Yangtze Finless porpoise	Q	Q	S	P	F
		Harbor porpoise	Q	Q	S	P	F
		Yangtze River dolphin	Q	Q	C	P	F
		Beluga whale	Q	Q	S	P	F
		Sperm whale	Q	Q	S	P	F
		Bowhead whale	Q	Q	S	P	F
		Minke whale	Q	Q	S	P	F
		Grey whale	Q	Q	S	P	F
		Hippopotamus	Q	Q	S	P	F
		Cattle	Q	Q	C	P	F
		Pig	Q	Q	C	P	Y
		Goat	Q	Q	C	P	F
		Przewalski’s horse	Q	Q	C	P	F
		Domestic cat	Q	Q	C	P	F
		Walrus	Q	Q	S	P	F
		Weddell seal	Q	Q	C	P	F
		Sea otter	Q	Q	C	P	F
		Dog	Q	Q	C	P	F
		Polar bear	Q	Q	C	P	F
		Great roundleaf bat	Q	Q	C	P	F
Euarcho-ntoglires		Human	Q	Q	C	P	F
		Rhesus macaque	Q	Q	C	P	F
		House mouse	Q	K	C	S	Y
Xemar-thra		Hoffmann’s two-fingered sloth	Q	K	N	S	S
		Nine-banded armadillo	Q	K	C	P	F
Afrotheria		West Indian manatee	Q	K	S	P	F
		African savanna elephant	Q	K	S	P	F
		Aardvark	Q	Q	C	P	Y
		Cape golden mole	Q	K	C	P	Y
		Cape rock hyrax	Q	K	C	P	Y
		Cape elephant shrew	Q	K	C	P	Y

### The Properties of DSA Differ From Those of HSA in Protein Structures

**FIGURE 1:**
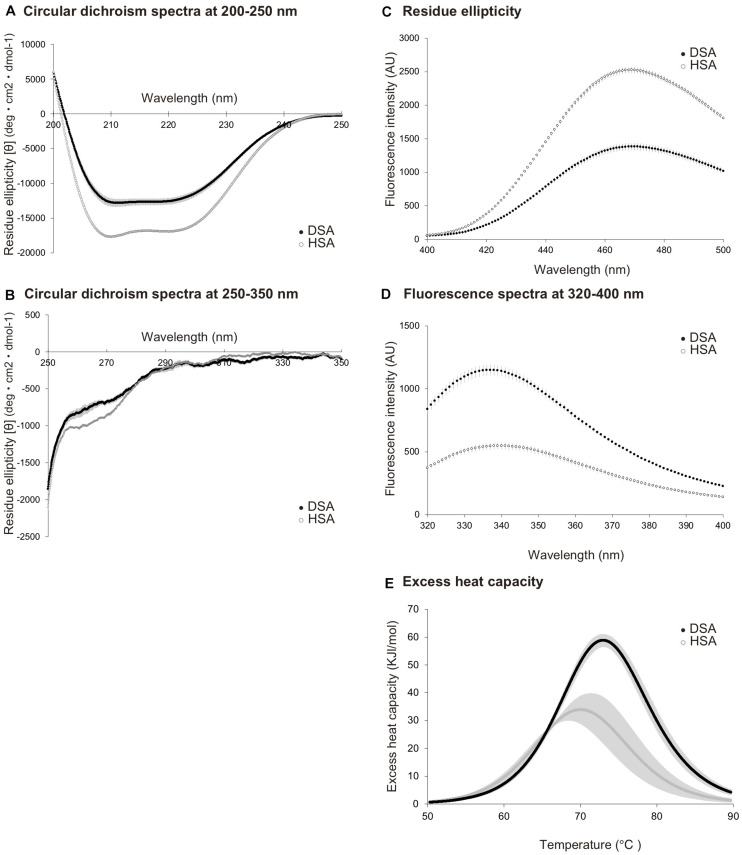
Comparison of biochemical properties between dolphin serum albumin (*N* = 5) and human serum albumin (*N* = 3). Circular dichroism spectra for dolphin serum albumin (DSA) and human serum albumin (HSA), measured at the wavelength of 200–250 nm **(A)** and 250–350 nm **(B)**. Residue ellipticity values are calculated as [θ] deg × cm^2^ × dmol-1. **(C)** Fluorescence spectra for DSA and HSA, obtained using surface hydrophobicity assay with 1-anilinonaphthalene-8-sulfonic acid used as indicator. Fluorescence intensities are expressed as arbitrary unit (AU). **(D)** Fluorescence spectra obtained using an ultraviolet fluorescence assay for DSA and HSA. Intensities are presented as AU. **(E)** Excess heat capacity of DSA and HSA. Capacities were calculated from the results of differential scanning calorimetric assay as Kcal/mol/°C. Dashed lines represent the calculated Tm values. All values are expressed as mean ± standard deviation. Black and white circles represent the values of DSA and HSA, respectively.

A circular dichroism spectrum analysis was used to estimate differences in the secondary structures of DSA and HSA. Spectral data collected in the range of 200–250 nm (Figure 1A) demonstrated that DSA showed a different pattern from that of HSA, with increased average residue ellipticity. Circular dichroism spectra, ranging from 250 to 350 nm (Figure 1B), also showed differences between DSA and HSA. Analysis of SA surface hydrophobicity, measured using ANS as indicator, also demonstrated different curves of mean fluorescence intensities for DSA and HSA, with DSA showing lower values than those for HSA (Figure 1C). We then performed an ultraviolet fluorescence assay to evaluate the state of aromatic amino acids. Our results indicated that the values for the fluorescence spectrum of DSA were approximately twofold higher than those of HSA (Figure 1D). Differential scanning calorimetric assay, performed to compare thermal stability between the species, revealed that thermal-transition temperature and transition enthalpy of DSA were greater than those of HSA (Tm: 74.2°C, ΔHcal: 267 kJ/mol) were significantly greater than those of HSA (Tm: 67.9°C, ΔHcal: 252 kJ/mol) (*p* < 0.05, Figure 1E). Using protein sequencing, the initial five amino acids at the N-terminus of DSA were confirmed as DTHKS (Asp-Thr-His-Lys-Ser) for the two dolphins.

### Antioxidative Activity Is Higher in DSA Than in HSA

**FIGURE 2:**
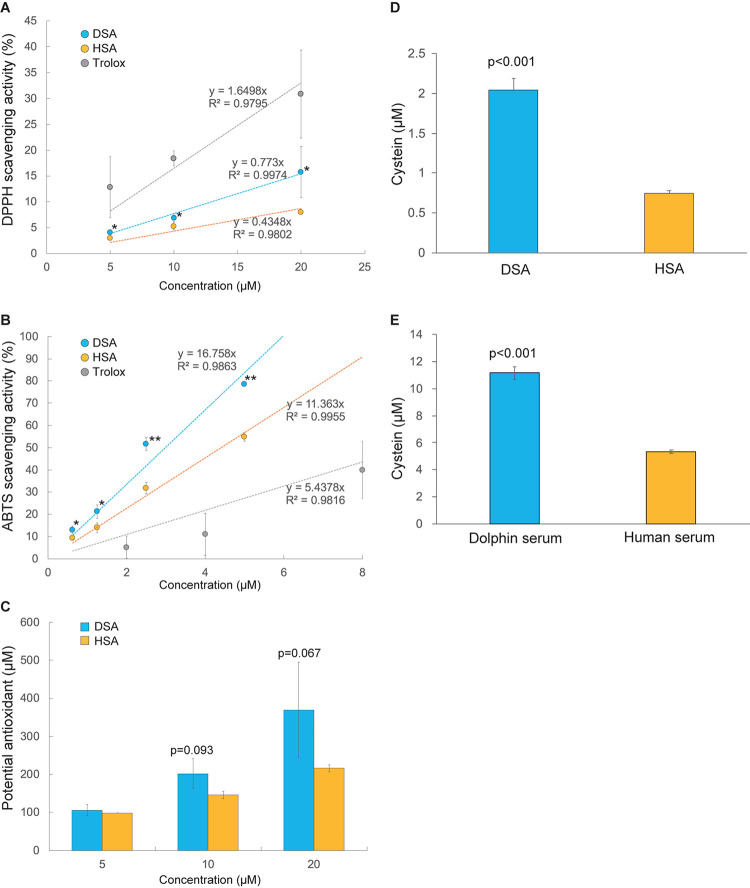
Comparison of antioxidant potencies between dolphin serum albumin (DSA) and human serum albumin (HSA). **(A)** The 2,2-diphenyl-1-picrylhydrazyl (DPPH) scavenging activities of DSA (*N* = 5) and HSA (*N* = 3). **(B)** 2,2′-Azino-bis (3-ethylbenzothiazoline-6-sulfonic acid (ABTS) radical scavenging activities of DSA (*N* = 5) and HSA (*N* = 3). **(C)** Total antioxidant capacity of DSA (*N* = 4) and HSA (*N* = 3) measured using the PAO kit. Intensities of these tests **(A–C)** are represented as percentage (%) of the absorbance of each sample to that of the blank (PB). Free-thiol activities were calculated as mole cysteine (μM) in serum ablumin; Astarisks indicate statistically significant differences between DSA and HSA (*P* < 0.05, ^∗∗^*P* < 0.01). **(D)** and whole serum **(E)** of the common bottlenose dolphin (*N* = 5) and human (*N* = 3). Values are expressed as mean ± standard error of mean.

The antioxidative activities of SAs were compared using DPPH. As shown in Figure 2A, DPPH radical scavenging activity was 2.1 times higher in DSA than in HSA (*p* < 0.05). This result is supported by a significant increase of ABTS radical scavenging ability and total antioxidant capacity of DSA compared to HSA (Figures 2B,C). We then measured the quantities of free thiol residues in SA and whole serum of the dolphin and human. Our results show that DSA had 2.7 times more free cysteine residues than HSA (*p* < 0.001, Figure 2D). In addition, the value for dolphin whole serum was 2.1 times higher than that for human whole serum (*p* < 0.001, Figure 2E). The ratio of free thiol residue number for DSA to that of whole dolphin serum was 0.2. An assay conducted to evaluate oxidation of SA by hydrogen peroxidase showed no significant difference in the degree of degradation between DSA and HSA (87.3 ± 5.2 and 91.5% ± 2.5% of the control remained, respectively; *p* = 0.587).

### 3D Structure Analysis

**FIGURE 3:**
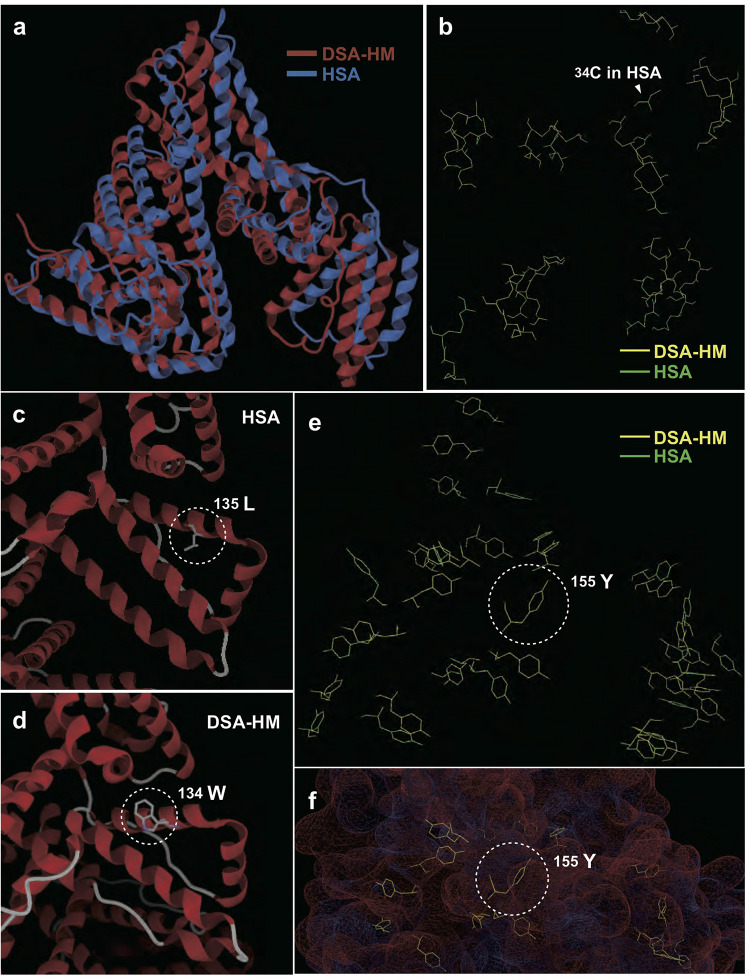
Comparison of 3D structures of dolphin serum albumin homology model (DSA-HM) and human serum albumin (HSA). **(A)** Comparison of whole 3D conformations. **(B)** Selective display of cysteine residues in DSA-HM and HSA shows similarity, except for the lack of 34Cys in DSA. Difference between 135Leu **(C)** and the corresponding residue (134Trp) in DSA-HM **(D)**. Selective display of tyrosine residues in DSA-HM and HSA **(E)**, and 155Tyr positioning on the surface of DSA-HM **(F)**.

To determine the characteristics contributing to antioxidative activity of dolphin albumin, we constructed a DSA-HM, and performed a conformational alignment of HM with HSA. As shown in Figure 3A, the 3D structures of DSA-HM and HSA were similar to each other. The positions of cysteine residues in HSA resembled those of DSA-HM except for ^34^Cys, and 17 pairs of disulfide bonds, generally observed in HSA, were replicated in DSA-HM (Figure 3B). Surface positioning and orientation of the hydroxyl group in ^34^Ser in DSA-HM was nearly identical to those of the thiol group in ^34^Cys of HSA. We observed characteristic substitutions of HSA residues (^135^Leu and ^156^Phe in HSA to ^134^Trp and ^155^Tyr in DSA-HM, respectively), and the residues were positioned at the surface of the protein (Figures 3C–F). Distances between sulfur atoms were similar between DSA-HM and HSA; however, angles between the sulfur atoms forming the SS bond and each β-carbon atom showed larger variances in DSA than in HSA (*F*-test, *p* < 0.05 for α1, *p* < 0.01 for α2, *p* < 0.001 for total; Supplementary Table S2).

## Discussion

Primary structure analysis of SAs showed a spontaneous substitution of ^34^Cys to Ser in the SA of cetacean lineages (with the exception of Yangtze River dolphin SA), as well as in some other marine mammals, hippopotamus, and African elephant. Methionine residues that can potentially bind to oxidants (^87^Met, ^446^Met, and ^548^Met) (Bourdon et al., 2005) were conserved in DSA (Supplementary Table S3). The importance of ^34^Cys in the antioxidative potential of SA has been shown in previous studies (Anraku et al., 2011; Bruschi et al., 2013). Anraku et al. (2015) reported that the free Cys substantially contributes to the antioxidative activity of SA, while substitution of ^34^Cys to Ser in HSA substantially impairs its antioxidative potency. Based on this report, we expected that DSA containing the same substitution (C34S) would show decreased antioxidative activity compared with that of HSA. However, our data unexpectedly show that both antioxidative capacity and thiol activity were greater in DSA than in HSA. DPPH scavenging activity of DSA was approximately 2 times higher than that of HSA, and ABTS and PAO tests also showed the stronger antioxidant capacity of DSA than that of HSA. In addition, thiol activities of DSA and dolphin whole serum were also 2.7 and 2.1 times higher than those of HSA and human whole serum, respectively.

To account for this discrepancy, we hypothesized that any pair potentially forming an SS bond in HSA is decomposed in DSA, because decomposition of SS bond may provide thiol group of free Cys contributing to catalyze ROS. Thus, the distance between sulfur atoms, and angle between the sulfur atoms forming an SS bond and each β-carbon atom, were measured for DSA-HM and HSA. This was done because these factors affect the stability of SS bonds. Creighton (1988) stated that cysteine residues involved in SS bonds require a distance of 2.05 ± 0.03 Å and an angle of approximately 103° for stable formation of an SS bond. In practice, the mean distance and angle in 13,030 types of proteins are 104.7° and 2.046Å, respectively. Additionally, SS bonds are rendered labile by increased conformational stress of the secondary structure if the angle is stretched or distance is elongated (Pijning et al., 2018). Our analyses, conducted using 3D models of SA, reveal that the distances between sulfur atoms in all SS bonds were appropriate and similar between HSA and DSA-HM. Sequence propriety was confirmed by N-terminus sequencing, which showed that mature DSA was obtained after the removal of the N-terminal pro-peptide (six residues) and signal peptide (18 residues) that are common to HSA (Peters, 1995). However, in DSA-HM, the angle between the sulfur and β-carbon atoms showed increased deviation, in which two SS bonds (^123^Cys-^168^Cys with 106.20° and 108.28°, and ^277^Cys-^288^Cys with 108.72° and 106.61°, respectively, Supplementary Table S2) had stretching angles of over 106.01° in both cysteine residues; this is the mean value of labile SS bonds in 551 types of proteins (Pijning et al., 2018). Conversely, in ^559^Cys, 106.01° was the greatest stretching angle in the SS bonds of HSA. The stress of the second structure, caused by stretching angles in DSA, potentially leads to the dissociation of SS bonds. Sulfur atoms within these bonds may act as free thiol groups, resulting in stronger antioxidant activity than that of HSA, irrespective of the absence of ^34^Cys. Concurrently, if these SS bonds in DSA are dissociated, the resultant free-thiol groups possessing reduction activity may be susceptible to oxidation, such as during reperfusion after a dive, which may render DSA a target for degradation. Additional studies such as crystallization, mass spectrometry, and mutation strategies are necessary to determine the actual behaviors of DSA.

DSA protein structure and structural perturbations differed from those of HSA. The circular spectra of DSA, detected at the wavelength of 250-200 and 250-350 nm, were discriminated from those of HSA, suggesting that the secondary and tertiary protein structures in DSA may differ from those of HSA (Kelly and Price, 2000). Mean fluorescence intensities, detected for SA using ANS, were lower in DSA than in HSA. This result indicates that surface hydrophobicity of DSA was likely lower than that of HSA, potentially leading to instability of protein structure (Pace et al., 2011). Differences in several amino acids between DSA and HSA are possibly one of the factors accounting for this difference in hydrophobicity. DSA-HM analysis revealed that the hydroxyl group of ^155^Tyr, which corresponded to ^156^Phe in HSA, was located on the surface of the protein. Additionally, ultraviolet fluorescence intensities of DSA, which were nearly 2-fold higher than those of HSA, indicate that the number of tryptophan residues at the surface of the protein may be greater in DSA than in HSA. Indeed, comparison using DSA-HM indicates that ^135^Leu in HSA was substituted with ^134^Trp in DSA-HM, positioned at the protein surface. This substitution may also contribute to the lower hydrophobicity of DSA because the hydropathy index is lower in tryptophan (−0.9) than in leucin (3.8) (Kyte and Doolittle, 1982). The α-helix to β-sheet ratios of the protein, calculated using CD spectra and 3D models, were 51.2 and 73.2% in DSA, and 69.8 and 72.6% in HSA, respectively (data not shown). The gap of the ratio in DSA was greater than that in HSA, indicating that DSA was more unstable than HSA. Differential scanning calorimetric assay showed that thermal transition temperature and transition enthalpy of DSA were higher than those of HSA, suggesting that DSA had higher thermal stability than that of HSA. Protein thermal stability is determined using the balance of hydrophobic or hydrogen bonding and entropy terms of a protein. If the maximum-stability temperature of a protein shifts to high-temperature side, the protein becomes unstable in the low-temperature range (Brandts, 1969). Collectively, our data suggest that DSA is likely more unstable than HSA in the body-temperature range, although additional studies are needed to confirm the instability of DSA. In addition, the potential occurrence of oxidation of SAs throughout the experiment procedures should be validated, although all experiments were carefully carried out according to references and/or manufacture’s manuals. At any hand, protein conformation plays a critical role in the stability and flexibility of a protein, while flexibility is important for exertion of protein functions (Ansari et al., 1985). Future studies should investigate whether instability of DSA contributes to its enhanced antioxidative activity or to its other physiological functions.

The high antioxidative potency of DSA, revealed in our present study, may contribute to countering the oxidative stress induced by rapid reperfusion of oxygen to ischemic tissues after a dive. Interestingly, the substitution of C34S is observed only in marine mammals (cetacea, walrus, and manatee) and their relatives (hippopotamus and the African elephant) (Boisserie et al., 2005; Nishihara et al., 2005), suggesting that ancestors of the African elephants were likely aquatic (Gaeth et al., 1999; Liu et al., 2008). These findings imply that this substitution imparts an evolutionary advantage for aquatic life, although the adaptational significance of this substitution needs to be investigated in future studies.

## Conclusion

Our present study reveals that DSA, which contained the substitution of ^34^Cys and showed biochemical properties that differed from those of HSA, possessed increased antioxidative capacity. This increased antioxidative capacity of DSA likely resulted from enhanced reduction activity caused by the dissociation of several disulfide bonds. Our data highlight the structural and functional singularity of SA, which is not only an antioxidant but also a circulating unstable protein in the aquatic mammals.

## Data Availability Statement

The raw data supporting the conclusions of this article will be made available by the authors, without undue reservation.

## Ethics Statement

The animal study was reviewed and approved by College of Bioresource Sciences, Nihon University.

## Author Contributions

MS designed and coordinated the study and drafted the manuscript. MS and MA conducted the experiments and performed the data analysis. WH and TK participated in protein structural analysis. KU and TE performed the blood sampling during health management of the dolphins. All the authors have read the manuscript and approved the final version for publication.

## Conflict of Interest

The authors declare that the research was conducted in the absence of any commercial or financial relationships that could be construed as a potential conflict of interest.

## Footnotes

1 https://www.ncbi.nlm.nih.gov/

2 https://swissmodel.expasy.org

3 http://molexus.io/

## Supplementary Material

Supplementary Table 1 Accession numbers of albumin sequences analyzed in this study.

Supplementary Table 2 Distance between sulfur atoms and angle between sulfur atoms forming an SS bond and each β-carbon atom (DSA-HM, dolphin serum albumin homology model; HSA, human serum albumin).

Supplementary Table 3 Comparison of amino acid sequences in dolphin serum albumin (DSA) and human serum albumin (HSA).

Supplementary text data_DSA Details in the atomic coordinate to construct DSA-HM are provided as a text file.
