# Supplementary material for: Antioxidative Potency of Dolphin Serum Albumin Is Stronger Than That of Human Serum Albumin Irrespective of Substitution of 34Cysteine With Serine
Source: Front Physiol. 2020 Nov 2;11:598451. doi: 10.3389/fphys.2020.598451 (PMC7667151; doi:10.3389/fphys.2020.598451)
Supplement: Supplementary Table 1 — Accession numbers of albumin sequences analyzed in this study. [file Data_Sheet_1.PDF]

Table S1. Accession numbers of albumin sequences analyzed in this study

Species	Scientific name	Accession Number
Common bottlenose dolphin	*Tursiops truncatus*	XP_004322082.2
Killer whale	*Orcinus orca*	XP_004283607.1
Yangtze	*Neophocaena asiaeorientalis*	XP_024609874.1
Harbor porpoise	*Phocoena phocoena*	GCA_003071005.1
Yangtze River dolphin	*Lipotes vexillifer*	XP_007467630.1
Beluga whale	*Delphinapterus leucas*	XP_022455292.1
Sperm whale	*Physeter catodon*	XP_023971164.1
Bowhead whale	*Balaena mysticetus*	Assembled by Keane et al. (2015)*
North Pacific minke whale	*Balaenoptera acutorostrata scammoni*	XP_007179910.1
Grey whale	*Eschrichtius robustus*	GCA_002189225.1
Hippopotamus	*Hippopotamus amphibius*	GCA_002995585.1
Cattle	*Bos taurus*	AAI02743.1
Pig	*Sus scrofa*	NP_001005208.1
Goat	*Capra hircus*	XP_005681801.1
Przewalski's horse	*Equus przewalskii*	XP_008524663.1
Domestic cat	*Felis catus*	NP_001009961.1
Walrus	*Odocoileus virginianus texanus*	XP_020741012.1
Weddell seal	*Leptonychotes weddellii*	XP_006729869.1
Sea otter	*Enhydra lutris kenyoni*	XP_022361396.1
Dog	*Canis lupus familiaris*	NP_001003026.1
Polar bear	*Ursus maritimus*	GCA_000687225.1
Great roundleaf bat	*Hipposideros armiger*	XP_019520870.1
Human	*Homo sapiens*	AAX63425.1
Rhesus macaque	*Macaca mulatta*	GCF_000772875.2
House mouse	*Mus musculus*	NP_033784.2
Hoffmann's two-fingered sloth	*Choloepus hoffmanni*	GCA_000164785.2
Nine-banded armadillo	*Dasypus novemcinctus*	GCA_000208655.2
West Indian manatee	*Trichechus manatus latirostris*	XP_023593546.1
African savanna elephant	*Loxodonta africana*	XP_003414202.1
Aardvark	*Orycteropus afer afer*	GCF_000298275.1
Cape golden mole	*Chrysochloris asiatica*	GCA_000296735.1
Cape rock hyrax	*Procavia capensis*	GCA_000152225.2
Cape elephant shrew	*Elephantulus edwardii*	GCA_000299155.1

[a] Keane M et al. (2015) Insights into the evolution of longevity from the bowhead whale genome. Cell Reports 10:112-122, doi:10.1016/j.celrep.2014.12.008.

Table S2. Distance between sulfur atoms and angle between sulfur atoms forming an SS bond and each β-carbon atom (DSA-HM, dolphin serum albumin homology model; HSA, human serum albumin)

	DSA_HM						HSA (PBD ID: 1BJ5)				
	Cys number	α1 angle	Cys number	α2 angle	distance (Å)		Cys number	α1 angle	Cys number	α2 angle	distance (Å)
1	53	103.81	62	104.84	2.03	1	53	102.91	62	103.90	2.03
2	75	101.79	91	101.96	2.03	2	75	103.73	91	103.54	2.03
3	90	105.57	101	103.79	2.02	3	90	103.70	101	102.12	2.02
4	123	**106.20**	168	**108.28**	2.04	4	124	102.12	169	103.70	2.02
5	167	102.46	176	**106.41**	2.03	5	168	105.37	177	102.31	2.03
6	199	104.00	245	101.72	2.03	6	200	104.76	246	103.80	2.03
7	244	103.24	252	104.55	2.02	7	245	104.84	253	105.73	2.03
8	264	101.54	278	99.43	2.02	8	265	102.18	279	103.72	2.03
9	277	**108.72**	288	**106.61**	2.05	9	278	104.57	289	102.73	2.03
10	315	103.76	360	**106.21**	2.03	10	316	104.85	361	104.42	2.03
11	359	104.05	368	104.26	2.02	11	360	104.21	369	104.56	2.03
12	391	103.77	437	104.19	2.04	12	392	104.54	438	105.00	2.03
13	436	104.31	447	105.42	2.04	13	437	105.00	448	103.88	2.03
14	460	100.61	476	100.64	2.01	14	461	104.06	477	103.43	2.03
15	475	103.68	486	**106.78**	2.04	15	476	102.98	487	104.58	2.02
16	513	103.98	558	104.71	2.02	16	514	104.98	559	**106.01**	2.03
17	557	104.52	566	105.42	2.04	17	558	103.16	567	102.52	2.03
average		103.88		104.42	2.03	average		104.00		103.88	2.03
SD		1.80		2.27	0.01	SD		0.98		1.07	0.00
variance		3.26*		5.16**	0.00	variance		0.97		1.15	0.00

[a]p < 0.05, **p < 0.01 vs HSA (F-test).

Table S3. Comparison of amino acid sequences in dolphin serum albumin (DSA) and human serum albumin (HSA)

```
                                                  34
DSA             DTHKSEIAHRFNDLGEENFKGLVLIAFSQYLQQSPFDEHVKLVNEITDFAKTCVADESAA  60
HSA             DAHKSEVAHRFKDLGEENFKALVLIAFAQYLQQCPFEDHVKLVNEVTEFAKTCVADESAE  60
                *:****:****:********.******:*****.**::*******:*:***********

                                          87
DSA             NCDKSLHTLFGDKLCAVASLRETYGEMADCCGKQDPERNECLLKHKDDNPDLPKLK-PDP  119
HSA             NCDKSLHTLFGDKLCTVATLRETYGEMADCCAKQEPERNECFLQHKDDNPNLPRLVRPEV  120
                ***************:**:************.**:******:*:******:**:*  *:

                     134/135                       155/156
DSA             ETFCTEFKENEQKFWGKYLYEIARRHPYFYAPELLYFAHQYKGVFAECCQAADKGACLIP  179
HSA             DVMCTAFHDNEETFLKKYLYEIARRHPYFYAPELLFFAKRYKAAFTECCQAADKAACLLP  180
                :.:** *::**:.*  *******************:**::**..*:********.***:*

DSA             KIETLREEVLASSARQRLKCTSIQKFGERALKAWSVARLSQKFPKADFAEVSKIVTDLTK  239
HSA             KLDELRDEGKASSAKQRLKCASLQKFGERAFKAWAVARLSQRFPKAEFAEVSKLVTDLTK  240
                *:: **:*  ****:*****:*:*******:***:******:****:******:******

DSA             VHKECCHGDLLECADDRADLAKYICENQATISSKLQKCCHKPLLEKSHCISEVEKDELPE  299
HSA             VHTECCHGDLLECADDRADLAKYICENQDSISSKLKECCEKPLLEKSHCIAEVENDEMPA  300
                **.*************************  :*****::**.**********:***:**:*

DSA             NLSLLAADFAEDKEVCKNYNEAKDVFLGTFLYDYARRHPEYSVSLLLRIAKGYEATLEDC  359
HSA             DLPSLAADFVESKDVCKNYAEAKDVFLGMFLYEYARRHPDYSVVLLLRLAKTYETTLEKC  360
                :*. *****.*.*:***** ******** ***:******:*** ****:** **:***.*

DSA             CAKDDPPACYATVFEKLRPLVEEPKNLIKQNCELFEKLGEYQFQNALIVRYTKKVPQVST  419
HSA             CAAADPHECYAKVFDEFKPLVEEPQNLIKQNCELFEQLGEYKFQNALLVRYTKKVPQVST  420
                **  **  ***.**::::******:***********:****:*****:************
                                        445/446
DSA             PTLVEVSRNLGRVGSKCCKNPESERMSCAEDYLSLVLNRLCVLHEKTPVSERVTKCCTES  479
HSA             PTLVEVSRNLGKVGSKCCKHPEAKRMPCAEDYLSVVLNQLCVLHEKTPVSDRVTKCCTES  480
                ***********:*******:**::**.*******:***:***********:*********

DSA             LVNRRPCFSALTVDETYEPKAFDEKTFTFHADLCTLPENEKQIKKQIALVELVKHKPKVT  539
HSA             LVNRRPCFSALEVDETYVPKEFNAETFTFHADICTLSEKERQIKKQTALVELVKHKPKAT  540
                *********** ***** ** *: :*******:***.*:*:***** ***********.*
                   547/548
DSA             EEQLKTVMGDFAAFVDKCCAADDKEACFALEGPKLVVKTREAIA-  583
HSA             KEQLKAVMDDFAAFVEKCCKADDKETCFAEEGKKLVAASQAALGL  585
                :****:**.******:*** *****:*** ** ***. :: *:.
```

Supplemental text data_DSA

```
HEADER    NONE
TITLE     HOMOLOGY MODEL OF TURSIOPS ALBUMIN
SOURCE    MOL_ID: 1;
SOURCE   2 ORGANISM_SCIENTIFIC: TURSIOPS TRUNCATUS;
SOURCE   3 ORGANISM_COMMON: COMMON BOLENOSE DOLPHIN;
SOURCE   4 ORGANISM_TAXID: 9739
KEYWDS    TURSIOPS ALBUMIN SERUM ALBUMIN, TRANSPORT PROTEIN
EXPDTA    THEORETICAL MODEL (SWISS-MODEL SERVER) WITH SIDE CHAIN MODIFICATION BY MOLEGRO
VIRTUAL DOCKER
AUTHOR    M. SUZUKI, M. ANRAKU, W. HAKAMATA, K. UEDA, T. ENDOH, T.KISHIDA
JRNL        AUTH   A.WATERHOUSE,M.BERTONI,S.BIENERT,G.STUDER,G.TAURIELLO,
JRNL        AUTH 2 R.GUMIENNY,F.T.HEER,T.A.P.DE BEER,C.REMPFER,L.BORDOLI,
JRNL        AUTH 3 R.LEPORE,T.SCHWEDE
JRNL        TITL   SWISS-MODEL: HOMOLOGY MODELLING OF PROTEIN STRUCTURES AND
JRNL        TITL 2 COMPLEXES
JRNL        REF    NUCLEIC.ACIDS.RES..           V.  46 W296  2018
JRNL        PMID   29788355
JRNL        DOI    10.1093/nar/gky427
REMARK   1
REMARK   1 REFERENCE 1
REMARK   1  AUTH   S.BIENERT,A.WATERHOUSE,T.A.P.DE BEER,G.TAURIELLO,G.STUDER,
REMARK   1  AUTH 2 L.BORDOLI,T.SCHWEDE
REMARK   1  TITL   THE SWISS-MODEL REPOSITORY - NEW FEATURES AND FUNCTIONALITY
REMARK   1  REF    NUCLEIC.ACIDS.RES..           V.  22       2017
REMARK   1  REFN                   ISSN 0305-1048
REMARK   1  PMID   27899672
REMARK   1  DOI    10.1093/nar/gkw1132
REMARK   1
REMARK   1 REFERENCE 2
REMARK   1  AUTH   N.GUEX,M.C.PEITSCH,T.SCHWEDE
REMARK   1  TITL   AUTOMATED COMPARATIVE PROTEIN STRUCTURE MODELING WITH
REMARK   1  TITL 2 SWISS-MODEL AND SWISS-PDBVIEWER: A HISTORICAL PERSPECTIVE
REMARK   1  REF    ELECTROPHORESIS               V.  30       2009
REMARK   1  REFN                   ISSN 0173-0835
REMARK   1  PMID   19517507
REMARK   1  DOI    10.1002/elps.200900140
REMARK   1
REMARK   1 REFERENCE 3
REMARK   1  AUTH   P.BENKERT,M.BIASINI,T.SCHWEDE
REMARK   1  TITL   TOWARD THE ESTIMATION OF THE ABSOLUTE QUALITY OF INDIVIDUAL
REMARK   1  TITL 2 PROTEIN STRUCTURE MODELS
REMARK   1  REF    BIOINFORMATICS                V.  27       2011
REMARK   1  REFN                   ISSN 1367-4803
REMARK   1  PMID   21134891
REMARK   1  DOI    10.1093/bioinformatics/btq662
REMARK   1
REMARK   1 REFERENCE 4
REMARK   1  AUTH   M.BERTONI,F.KIEFER,M.BIASINI,L.BORDOLI,T.SCHWEDE
REMARK   1  TITL   MODELING PROTEIN QUATERNARY STRUCTURE OF HOMO- AND
REMARK   1  TITL 2 HETERO-OLIGOMERS BEYOND BINARY INTERACTIONS BY HOMOLOGY
REMARK   1  REF    SCI.REP.                      V.   7       2017
REMARK   1  REFN                   ISSN
REMARK   1  PMID   28874689
REMARK   1  DOI    10.1038/s41598-017-09654-8
REMARK   1
REMARK   1 DISCLAIMER
REMARK   1 The SWISS-MODEL SERVER produces theoretical models for proteins.
REMARK   1 The results of any theoretical modelling procedure is
REMARK   1 NON-EXPERIMENTAL and MUST be considered with care. These models may
REMARK   1 contain significant errors. This is especially true for automated
REMARK   1 modeling since there is no human intervention during model
REMARK   1 building. Please read the header section and the logfile carefully
REMARK   1 to know what templates and alignments were used during the model
REMARK   1 building process. All information by the SWISS-MODEL SERVER is
REMARK   1 provided "AS-IS", without any warranty, expressed or implied.
REMARK   2
REMARK   2 COPYRIGHT NOTICE
REMARK   2 This SWISS-MODEL protein model is copyright. It is produced by the
REMARK   2 SWISS-MODEL server, developed by the Computational Structural
REMARK   2 Biology Group at the SIB Swiss Institute of Bioinformatics at the
REMARK   2 Biozentrum, University of Basel (https://swissmodel.expasy.org). This
REMARK   2 model is licensed under the CC BY-SA 4.0 Creative Commons
```

```
REMARK   2 Attribution-ShareAlike 4.0 International License
REMARK   2 (https://creativecommons.org/licenses/by-sa/4.0/legalcode), i.e. you
REMARK   2 can copy and redistribute the model in any medium or format,
REMARK   2 transform and build upon the model for any purpose, even
REMARK   2 commercially, under the following terms:
REMARK   2 Attribution - You must give appropriate credit, provide a link to
REMARK   2 the license, and indicate if changes were made. You may do so in any
REMARK   2 reasonable manner, but not in any way that suggests the licensor
REMARK   2 endorses you or your use. When you publish, patent or distribute
REMARK   2 results that were fully or partially based on the model, please cite
REMARK   2 the corresponding papers mentioned under JRNL.
REMARK   2 ShareAlike - If you remix, transform, or build upon the material,
REMARK   2 you must distribute your contributions under the same license as the
REMARK   2 original.
REMARK   2 No additional restrictions - you may not apply legal terms or
REMARK   2 technological measures that legally restrict others from doing
REMARK   2 anything the license permits.
REMARK   2 Find a human-readable summary of (and not a substitute for) the
REMARK   2 CC BY-SA 4.0 license at this link:
REMARK   2 https://creativecommons.org/licenses/by-sa/4.0/
REMARK   3
REMARK   3 MODEL INFORMATION
REMARK   3  ENGIN   PROMOD3
REMARK   3  VERSN   3.0.0
REMARK   3  OSTAT   monomer
REMARK   3  OSRSN   MONOMER (USER)
REMARK   3  QSPRD   0.000
REMARK   3  QMN4    -0.43
REMARK   3  MODT    FALSE
REMARK   3
REMARK   3 TEMPLATE 1
REMARK   3  PDBID   4f5s
REMARK   3  CHAIN   A
REMARK   3  MMCIF   A
REMARK   3  PDBV    2020-03-27
REMARK   3  SMTLE   4f5s.1.A
REMARK   3  SMTLV   2020-04-01
REMARK   3  MTHD    X-RAY DIFFRACTION 2.47 A
REMARK   3  FOUND   BLAST
REMARK   3  SIM     0.57
REMARK   3  SID     83.19
REMARK   3  OSTAT   monomer
REMARK   3  ALN A TRG DTHKSEIAHRFNDLGEENFKGLVLIAFSQYLQQSPFDEHVKLVNEITDFAKTCVADES
REMARK   3  ALN A TRG AANCDKSLHTLFGDKLCAVASLRETYGEMADCCGKQDPERNECLLKHKDDNPDLPKLK
REMARK   3  ALN A TRG PDPETFCTEFKENEQKFWGKYLYEIARRHPYFYAPELLYFAHQYKGVFAECCQAADKG
REMARK   3  ALN A TRG ACLIPKIETLREEVLASSARQRLKCTSIQKFGERALKAWSVARLSQKFPKADFAEVSK
REMARK   3  ALN A TRG IVTDLTKVHKECCHGDLLECADDRADLAKYICENQATISSKLQKCCHKPLLEKSHCIS
REMARK   3  ALN A TRG EVEKDELPENLSLLAADFAEDKEVCKNYNEAKDVFLGTFLYDYARRHPEYSVSLLLRI
REMARK   3  ALN A TRG AKGYEATLEDCCAKDDPPACYATVFEKLRPLVEEPKNLIKQNCELFEKLGEYQFQNAL
REMARK   3  ALN A TRG IVRYTKKVPQVSTPTLVEVSRNLGRVGSKCCKNPESERMSCAEDYLSLVLNRLCVLHE
REMARK   3  ALN A TRG KTPVSERVTKCCTESLVNRRPCFSALTVDETYEPKAFDEKTFTFHADLCTLPENEKQI
REMARK   3  ALN A TRG KKQIALVELVKHKPKVTEEQLKTVMGDFAAFVDKCCAADDKEACFALEGPKLVVKTRE
REMARK   3  ALN A TRG AIA
REMARK   3  ALN A TPL DTHKSEIAHRFKDLGEEHFKGLVLIAFSQYLQQCPFDEHVKLVNELTEFAKTCVADES
REMARK   3  ALN A TPL HAGCEKSLHTLFGDELCKVASLRETYGDMADCCEKQEPERNECFLSHKDDSPDLPKLK
REMARK   3  ALN A TPL PDPNTLCDEFKADEKKFWGKYLYEIARRHPYFYAPELLYYANKYNGVFQECCQAEDKG
REMARK   3  ALN A TPL ACLLPKIETMREKVLTSSARQRLRCASIQKFGERALKAWSVARLSQKFPKAEFVEVTK
REMARK   3  ALN A TPL LVTDLTKVHKECCHGDLLECADDRADLAKYICDNQDTISSKLKECCDKPLLEKSHCIA
REMARK   3  ALN A TPL EVEKDAIPENLPPLTADFAEDKDVCKNYQEAKDAFLGSFLYEYSRRHPEYAVSVLLRL
REMARK   3  ALN A TPL AKEYEATLEECCAKDDPHACYSTVFDKLKHLVDEPQNLIKQNCDQFEKLGEYGFQNAL
REMARK   3  ALN A TPL IVRYTRKVPQVSTPTLVEVSRSLGKVGTRCCTKPESERMPCTEDYLSLILNRLCVLHE
REMARK   3  ALN A TPL KTPVSEKVTKCCTESLVNRRPCFSALTPDETYVPKAFDEKLFTFHADICTLPDTEKQI
REMARK   3  ALN A TPL KKQTALVELLKHKPKATEEQLKTVMENFVAFVDKCCAADDKEACFAVEGPKLVVSTQT
REMARK   3  ALN A TPL ALA
REMARK   3  ALN A OFF 0
ATOM      1  N   ASP A   1     -15.767  24.814  73.434  1.00  0.40           N
ATOM      2  CA  ASP A   1     -15.352  25.432  74.708  1.00  0.40           C
ATOM      3  C   ASP A   1     -13.893  25.147  75.076  1.00  0.40           C
ATOM      4  O   ASP A   1     -13.588  24.725  76.183  1.00  0.40           O
ATOM      5  CB  ASP A   1     -16.357  24.835  75.711  1.00  0.40           C
ATOM      6  CG  ASP A   1     -16.961  26.052  76.373  1.00  0.40           C
ATOM      7  OD1 ASP A   1     -16.158  26.895  76.833  1.00  0.40           O
ATOM      8  OD2 ASP A   1     -18.193  26.204  76.240  1.00  0.40           O
ATOM      9  N   THR A   2     -12.923  25.306  74.136  1.00  0.26           N
ATOM     10  CA  THR A   2     -11.586  24.784  74.368  1.00  0.26           C
ATOM     11  C   THR A   2     -10.588  25.923  74.289  1.00  0.26           C
```

ATOM	12	O	THR	A	2	-10.289	26.456	73.224	1.00	0.26	O
ATOM	13	CB	THR	A	2	-11.267	23.578	73.471	1.00	0.26	C
ATOM	14	OG1	THR	A	2	-9.937	23.111	73.626	1.00	0.26	O
ATOM	15	CG2	THR	A	2	-11.513	23.828	71.974	1.00	0.26	C
ATOM	16	N	HIS	A	3	-10.071	26.336	75.470	1.00	0.37	N
ATOM	17	CA	HIS	A	3	-8.934	27.225	75.650	1.00	0.37	C
ATOM	18	C	HIS	A	3	-7.691	26.362	75.593	1.00	0.37	C
ATOM	19	O	HIS	A	3	-7.271	25.768	76.576	1.00	0.37	O
ATOM	20	CB	HIS	A	3	-8.999	27.941	77.018	1.00	0.37	C
ATOM	21	CG	HIS	A	3	-9.842	29.174	77.028	1.00	0.37	C
ATOM	22	ND1	HIS	A	3	-10.393	29.579	78.224	1.00	0.37	N
ATOM	23	CD2	HIS	A	3	-10.059	30.111	76.070	1.00	0.37	C
ATOM	24	CE1	HIS	A	3	-10.940	30.748	77.975	1.00	0.37	C
ATOM	25	NE2	HIS	A	3	-10.768	31.120	76.683	1.00	0.37	N
ATOM	26	N	LYS	A	4	-7.128	26.221	74.383	1.00	0.70	N
ATOM	27	CA	LYS	A	4	-6.341	25.060	74.042	1.00	0.70	C
ATOM	28	C	LYS	A	4	-4.949	24.811	74.665	1.00	0.70	C
ATOM	29	O	LYS	A	4	-4.683	23.731	75.193	1.00	0.70	O
ATOM	30	CB	LYS	A	4	-6.308	24.940	72.503	1.00	0.70	C
ATOM	31	CG	LYS	A	4	-6.217	26.242	71.688	1.00	0.70	C
ATOM	32	CD	LYS	A	4	-5.638	25.950	70.292	1.00	0.70	C
ATOM	33	CE	LYS	A	4	-6.395	24.903	69.469	1.00	0.70	C
ATOM	34	NZ	LYS	A	4	-5.492	24.329	68.444	1.00	0.70	N
ATOM	35	N	SER	A	5	-4.030	25.797	74.659	1.00	0.84	N
ATOM	36	CA	SER	A	5	-2.615	25.552	74.909	1.00	0.84	C
ATOM	37	C	SER	A	5	-2.270	25.951	76.340	1.00	0.84	C
ATOM	38	O	SER	A	5	-2.203	27.123	76.701	1.00	0.84	O
ATOM	39	CB	SER	A	5	-1.746	26.262	73.817	1.00	0.84	C
ATOM	40	OG	SER	A	5	-0.340	26.340	74.084	1.00	0.84	O
ATOM	41	N	GLU	A	6	-2.052	24.935	77.216	1.00	0.82	N
ATOM	42	CA	GLU	A	6	-1.680	25.112	78.621	1.00	0.82	C
ATOM	43	C	GLU	A	6	-0.343	25.821	78.835	1.00	0.82	C
ATOM	44	O	GLU	A	6	-0.205	26.672	79.717	1.00	0.82	O
ATOM	45	CB	GLU	A	6	-1.759	23.796	79.458	1.00	0.82	C
ATOM	46	CG	GLU	A	6	-3.096	23.640	80.234	1.00	0.82	C
ATOM	47	CD	GLU	A	6	-3.366	24.809	81.198	1.00	0.82	C
ATOM	48	OE1	GLU	A	6	-2.660	24.988	82.231	1.00	0.82	O
ATOM	49	OE2	GLU	A	6	-4.324	25.556	80.887	1.00	0.82	O
ATOM	50	N	ILE	A	7	0.698	25.542	78.012	1.00	0.85	N
ATOM	51	CA	ILE	A	7	1.968	26.265	78.114	1.00	0.85	C
ATOM	52	C	ILE	A	7	1.850	27.755	77.794	1.00	0.85	C
ATOM	53	O	ILE	A	7	2.469	28.597	78.441	1.00	0.85	O
ATOM	54	CB	ILE	A	7	3.166	25.630	77.388	1.00	0.85	C
ATOM	55	CG1	ILE	A	7	4.490	26.171	77.995	1.00	0.85	C
ATOM	56	CG2	ILE	A	7	3.092	25.815	75.854	1.00	0.85	C
ATOM	57	CD1	ILE	A	7	5.722	25.316	77.680	1.00	0.85	C
ATOM	58	N	ALA	A	8	1.026	28.102	76.777	1.00	0.91	N
ATOM	59	CA	ALA	A	8	0.728	29.461	76.386	1.00	0.91	C
ATOM	60	C	ALA	A	8	-0.115	30.209	77.413	1.00	0.91	C
ATOM	61	O	ALA	A	8	0.198	31.362	77.721	1.00	0.91	O
ATOM	62	CB	ALA	A	8	0.100	29.498	74.980	1.00	0.91	C
ATOM	63	N	HIS	A	9	-1.151	29.561	78.021	1.00	0.81	N
ATOM	64	CA	HIS	A	9	-1.947	30.154	79.104	1.00	0.81	C
ATOM	65	C	HIS	A	9	-1.046	30.584	80.261	1.00	0.81	C
ATOM	66	O	HIS	A	9	-0.985	31.753	80.627	1.00	0.81	O
ATOM	67	CB	HIS	A	9	-3.094	29.220	79.614	1.00	0.81	C
ATOM	68	CG	HIS	A	9	-3.991	29.794	80.678	1.00	0.81	C
ATOM	69	ND1	HIS	A	9	-4.164	29.085	81.848	1.00	0.81	N
ATOM	70	CD2	HIS	A	9	-4.654	30.982	80.747	1.00	0.81	C
ATOM	71	CE1	HIS	A	9	-4.913	29.862	82.613	1.00	0.81	C
ATOM	72	NE2	HIS	A	9	-5.229	31.031	82.002	1.00	0.81	N
ATOM	73	N	ARG	A	10	-0.196	29.665	80.763	1.00	0.81	N
ATOM	74	CA	ARG	A	10	0.720	29.966	81.851	1.00	0.81	C
ATOM	75	C	ARG	A	10	1.806	30.992	81.571	1.00	0.81	C
ATOM	76	O	ARG	A	10	2.142	31.809	82.429	1.00	0.81	O
ATOM	77	CB	ARG	A	10	1.385	28.682	82.388	1.00	0.81	C
ATOM	78	CG	ARG	A	10	0.370	27.657	82.929	1.00	0.81	C
ATOM	79	CD	ARG	A	10	-0.708	28.257	83.842	1.00	0.81	C
ATOM	80	NE	ARG	A	10	-1.685	27.183	84.115	1.00	0.81	N
ATOM	81	CZ	ARG	A	10	-2.838	27.408	84.750	1.00	0.81	C
ATOM	82	NH1	ARG	A	10	-3.196	28.572	85.284	1.00	0.81	N
ATOM	83	NH2	ARG	A	10	-3.683	26.383	84.787	1.00	0.81	N
ATOM	84	N	PHE	A	11	2.412	30.969	80.367	1.00	0.89	N
ATOM	85	CA	PHE	A	11	3.434	31.931	79.992	1.00	0.89	C
ATOM	86	C	PHE	A	11	2.913	33.366	79.980	1.00	0.89	C
ATOM	87	O	PHE	A	11	3.567	34.272	80.497	1.00	0.89	O

ATOM	88	CB	PHE	A	11	4.069	31.533	78.632	1.00	0.89	C
ATOM	89	CG	PHE	A	11	5.257	32.395	78.300	1.00	0.89	C
ATOM	90	CD1	PHE	A	11	5.117	33.570	77.543	1.00	0.89	C
ATOM	91	CD2	PHE	A	11	6.513	32.085	78.832	1.00	0.89	C
ATOM	92	CE1	PHE	A	11	6.209	34.419	77.329	1.00	0.89	C
ATOM	93	CE2	PHE	A	11	7.597	32.947	78.649	1.00	0.89	C
ATOM	94	CZ	PHE	A	11	7.452	34.108	77.882	1.00	0.89	C
ATOM	95	N	ASN	A	12	1.701	33.586	79.427	1.00	0.88	N
ATOM	96	CA	ASN	A	12	1.044	34.881	79.439	1.00	0.88	C
ATOM	97	C	ASN	A	12	0.747	35.397	80.838	1.00	0.88	C
ATOM	98	O	ASN	A	12	1.066	36.541	81.165	1.00	0.88	O
ATOM	99	CB	ASN	A	12	−0.309	34.785	78.704	1.00	0.88	C
ATOM	100	CG	ASN	A	12	−0.110	34.669	77.201	1.00	0.88	C
ATOM	101	OD1	ASN	A	12	0.926	35.014	76.630	1.00	0.88	O
ATOM	102	ND2	ASN	A	12	−1.177	34.197	76.514	1.00	0.88	N
ATOM	103	N	ASP	A	13	0.150	34.533	81.680	1.00	0.88	N
ATOM	104	CA	ASP	A	13	−0.361	34.900	82.983	1.00	0.88	C
ATOM	105	C	ASP	A	13	0.723	35.245	84.005	1.00	0.88	C
ATOM	106	O	ASP	A	13	0.655	36.253	84.711	1.00	0.88	O
ATOM	107	CB	ASP	A	13	−1.396	33.829	83.418	1.00	0.88	C
ATOM	108	CG	ASP	A	13	−2.655	33.912	82.535	1.00	0.88	C
ATOM	109	OD1	ASP	A	13	−3.620	33.150	82.785	1.00	0.88	O
ATOM	110	OD2	ASP	A	13	−2.686	34.739	81.580	1.00	0.88	O
ATOM	111	N	LEU	A	14	1.812	34.452	84.052	1.00	0.89	N
ATOM	112	CA	LEU	A	14	2.928	34.690	84.951	1.00	0.89	C
ATOM	113	C	LEU	A	14	3.949	35.705	84.456	1.00	0.89	C
ATOM	114	O	LEU	A	14	4.661	36.327	85.247	1.00	0.89	O
ATOM	115	CB	LEU	A	14	3.720	33.382	85.160	1.00	0.89	C
ATOM	116	CG	LEU	A	14	2.919	32.244	85.806	1.00	0.89	C
ATOM	117	CD1	LEU	A	14	3.689	30.922	85.675	1.00	0.89	C
ATOM	118	CD2	LEU	A	14	2.577	32.553	87.271	1.00	0.89	C
ATOM	119	N	GLY	A	15	4.056	35.872	83.122	1.00	0.95	N
ATOM	120	CA	GLY	A	15	5.133	36.590	82.447	1.00	0.95	C
ATOM	121	C	GLY	A	15	6.442	35.830	82.430	1.00	0.95	C
ATOM	122	O	GLY	A	15	6.611	34.837	83.139	1.00	0.95	O
ATOM	123	N	GLU	A	16	7.422	36.279	81.603	1.00	0.86	N
ATOM	124	CA	GLU	A	16	8.619	35.489	81.319	1.00	0.86	C
ATOM	125	C	GLU	A	16	9.473	35.159	82.546	1.00	0.86	C
ATOM	126	O	GLU	A	16	9.779	33.992	82.789	1.00	0.86	O
ATOM	127	CB	GLU	A	16	9.526	36.121	80.217	1.00	0.86	C
ATOM	128	CG	GLU	A	16	10.529	35.099	79.606	1.00	0.86	C
ATOM	129	CD	GLU	A	16	11.652	35.645	78.718	1.00	0.86	C
ATOM	130	OE1	GLU	A	16	12.585	36.360	79.105	1.00	0.86	O
ATOM	131	OE2	GLU	A	16	11.627	35.139	77.575	1.00	0.86	O
ATOM	132	N	GLU	A	17	9.804	36.162	83.394	1.00	0.85	N
ATOM	133	CA	GLU	A	17	10.707	35.996	84.528	1.00	0.85	C
ATOM	134	C	GLU	A	17	10.249	34.989	85.583	1.00	0.85	C
ATOM	135	O	GLU	A	17	11.001	34.129	86.050	1.00	0.85	O
ATOM	136	CB	GLU	A	17	10.994	37.361	85.221	1.00	0.85	C
ATOM	137	CG	GLU	A	17	11.569	38.496	84.328	1.00	0.85	C
ATOM	138	CD	GLU	A	17	12.606	37.996	83.320	1.00	0.85	C
ATOM	139	OE1	GLU	A	17	13.470	37.167	83.707	1.00	0.85	O
ATOM	140	OE2	GLU	A	17	12.506	38.437	82.151	1.00	0.85	O
ATOM	141	N	ASN	A	18	8.953	35.057	85.950	1.00	0.87	N
ATOM	142	CA	ASN	A	18	8.288	34.110	86.833	1.00	0.87	C
ATOM	143	C	ASN	A	18	8.130	32.744	86.197	1.00	0.87	C
ATOM	144	O	ASN	A	18	8.315	31.731	86.869	1.00	0.87	O
ATOM	145	CB	ASN	A	18	6.927	34.619	87.357	1.00	0.87	C
ATOM	146	CG	ASN	A	18	7.149	35.758	88.346	1.00	0.87	C
ATOM	147	OD1	ASN	A	18	8.222	35.935	88.924	1.00	0.87	O
ATOM	148	ND2	ASN	A	18	6.085	36.554	88.597	1.00	0.87	N
ATOM	149	N	PHE	A	19	7.823	32.675	84.880	1.00	0.89	N
ATOM	150	CA	PHE	A	19	7.830	31.420	84.144	1.00	0.89	C
ATOM	151	C	PHE	A	19	9.200	30.738	84.185	1.00	0.89	C
ATOM	152	O	PHE	A	19	9.309	29.589	84.600	1.00	0.89	O
ATOM	153	CB	PHE	A	19	7.372	31.653	82.675	1.00	0.89	C
ATOM	154	CG	PHE	A	19	7.334	30.397	81.840	1.00	0.89	C
ATOM	155	CD1	PHE	A	19	8.475	29.981	81.129	1.00	0.89	C
ATOM	156	CD2	PHE	A	19	6.166	29.626	81.748	1.00	0.89	C
ATOM	157	CE1	PHE	A	19	8.462	28.807	80.370	1.00	0.89	C
ATOM	158	CE2	PHE	A	19	6.144	28.462	80.968	1.00	0.89	C
ATOM	159	CZ	PHE	A	19	7.292	28.053	80.283	1.00	0.89	C
ATOM	160	N	LYS	A	20	10.291	31.448	83.841	1.00	0.87	N
ATOM	161	CA	LYS	A	20	11.646	30.917	83.898	1.00	0.87	C
ATOM	162	C	LYS	A	20	12.102	30.507	85.289	1.00	0.87	C
ATOM	163	O	LYS	A	20	12.690	29.441	85.483	1.00	0.87	O

ATOM	164	CB	LYS	A	20	12.642	31.955	83.350	1.00	0.87	C
ATOM	165	CG	LYS	A	20	12.666	32.033	81.821	1.00	0.87	C
ATOM	166	CD	LYS	A	20	13.654	33.118	81.370	1.00	0.87	C
ATOM	167	CE	LYS	A	20	15.112	32.651	81.320	1.00	0.87	C
ATOM	168	NZ	LYS	A	20	15.417	32.339	79.911	1.00	0.87	N
ATOM	169	N	GLY	A	21	11.816	31.353	86.296	1.00	0.93	N
ATOM	170	CA	GLY	A	21	12.012	31.055	87.708	1.00	0.93	C
ATOM	171	C	GLY	A	21	11.392	29.768	88.197	1.00	0.93	C
ATOM	172	O	GLY	A	21	12.063	28.927	88.791	1.00	0.93	O
ATOM	173	N	LEU	A	22	10.082	29.579	87.952	1.00	0.90	N
ATOM	174	CA	LEU	A	22	9.344	28.378	88.317	1.00	0.90	C
ATOM	175	C	LEU	A	22	9.771	27.123	87.593	1.00	0.90	C
ATOM	176	O	LEU	A	22	9.845	26.045	88.181	1.00	0.90	O
ATOM	177	CB	LEU	A	22	7.837	28.592	88.119	1.00	0.90	C
ATOM	178	CG	LEU	A	22	7.250	29.611	89.106	1.00	0.90	C
ATOM	179	CD1	LEU	A	22	5.885	30.074	88.604	1.00	0.90	C
ATOM	180	CD2	LEU	A	22	7.168	29.063	90.539	1.00	0.90	C
ATOM	181	N	VAL	A	23	10.070	27.227	86.292	1.00	0.91	N
ATOM	182	CA	VAL	A	23	10.595	26.131	85.505	1.00	0.91	C
ATOM	183	C	VAL	A	23	11.981	25.678	85.958	1.00	0.91	C
ATOM	184	O	VAL	A	23	12.248	24.476	86.035	1.00	0.91	O
ATOM	185	CB	VAL	A	23	10.602	26.518	84.049	1.00	0.91	C
ATOM	186	CG1	VAL	A	23	11.243	25.454	83.160	1.00	0.91	C
ATOM	187	CG2	VAL	A	23	9.163	26.697	83.532	1.00	0.91	C
ATOM	188	N	LEU	A	24	12.890	26.622	86.320	1.00	0.91	N
ATOM	189	CA	LEU	A	24	14.157	26.286	86.967	1.00	0.91	C
ATOM	190	C	LEU	A	24	13.922	25.550	88.269	1.00	0.91	C
ATOM	191	O	LEU	A	24	14.507	24.498	88.494	1.00	0.91	O
ATOM	192	CB	LEU	A	24	15.044	27.525	87.259	1.00	0.91	C
ATOM	193	CG	LEU	A	24	16.359	27.248	88.017	1.00	0.91	C
ATOM	194	CD1	LEU	A	24	17.392	26.484	87.174	1.00	0.91	C
ATOM	195	CD2	LEU	A	24	16.939	28.567	88.529	1.00	0.91	C
ATOM	196	N	ILE	A	25	12.999	26.046	89.129	1.00	0.91	N
ATOM	197	CA	ILE	A	25	12.678	25.366	90.380	1.00	0.91	C
ATOM	198	C	ILE	A	25	12.162	23.951	90.129	1.00	0.91	C
ATOM	199	O	ILE	A	25	12.654	22.993	90.720	1.00	0.91	O
ATOM	200	CB	ILE	A	25	11.668	26.145	91.222	1.00	0.91	C
ATOM	201	CG1	ILE	A	25	12.253	27.494	91.697	1.00	0.91	C
ATOM	202	CG2	ILE	A	25	11.158	25.323	92.434	1.00	0.91	C
ATOM	203	CD1	ILE	A	25	11.147	28.499	92.036	1.00	0.91	C
ATOM	204	N	ALA	A	26	11.217	23.762	89.181	1.00	0.93	N
ATOM	205	CA	ALA	A	26	10.687	22.447	88.859	1.00	0.93	C
ATOM	206	C	ALA	A	26	11.757	21.475	88.382	1.00	0.93	C
ATOM	207	O	ALA	A	26	11.831	20.347	88.855	1.00	0.93	O
ATOM	208	CB	ALA	A	26	9.569	22.513	87.795	1.00	0.93	C
ATOM	209	N	PHE	A	27	12.666	21.884	87.479	1.00	0.88	N
ATOM	210	CA	PHE	A	27	13.795	21.036	87.117	1.00	0.88	C
ATOM	211	C	PHE	A	27	14.760	20.715	88.262	1.00	0.88	C
ATOM	212	O	PHE	A	27	15.180	19.562	88.418	1.00	0.88	O
ATOM	213	CB	PHE	A	27	14.608	21.608	85.922	1.00	0.88	C
ATOM	214	CG	PHE	A	27	13.906	21.429	84.607	1.00	0.88	C
ATOM	215	CD1	PHE	A	27	13.620	22.476	83.711	1.00	0.88	C
ATOM	216	CD2	PHE	A	27	13.615	20.123	84.215	1.00	0.88	C
ATOM	217	CE1	PHE	A	27	13.028	22.200	82.467	1.00	0.88	C
ATOM	218	CE2	PHE	A	27	12.988	19.846	83.002	1.00	0.88	C
ATOM	219	CZ	PHE	A	27	12.694	20.886	82.120	1.00	0.88	C
ATOM	220	N	SER	A	28	15.104	21.713	89.101	1.00	0.91	N
ATOM	221	CA	SER	A	28	15.973	21.597	90.266	1.00	0.91	C
ATOM	222	C	SER	A	28	15.491	20.626	91.324	1.00	0.91	C
ATOM	223	O	SER	A	28	16.281	19.924	91.952	1.00	0.91	O
ATOM	224	CB	SER	A	28	16.154	22.970	90.951	1.00	0.91	C
ATOM	225	OG	SER	A	28	17.025	23.815	90.202	1.00	0.91	O
ATOM	226	N	GLN	A	29	14.169	20.568	91.559	1.00	0.87	N
ATOM	227	CA	GLN	A	29	13.579	19.634	92.499	1.00	0.87	C
ATOM	228	C	GLN	A	29	13.435	18.219	91.976	1.00	0.87	C
ATOM	229	O	GLN	A	29	13.339	17.276	92.754	1.00	0.87	O
ATOM	230	CB	GLN	A	29	12.183	20.113	92.929	1.00	0.87	C
ATOM	231	CG	GLN	A	29	12.248	21.503	93.578	1.00	0.87	C
ATOM	232	CD	GLN	A	29	10.907	21.941	94.151	1.00	0.87	C
ATOM	233	OE1	GLN	A	29	9.829	21.597	93.664	1.00	0.87	O
ATOM	234	NE2	GLN	A	29	10.984	22.734	95.247	1.00	0.87	N
ATOM	235	N	TYR	A	30	13.385	18.042	90.643	1.00	0.86	N
ATOM	236	CA	TYR	A	30	13.282	16.735	90.012	1.00	0.86	C
ATOM	237	C	TYR	A	30	14.634	16.058	89.870	1.00	0.86	C
ATOM	238	O	TYR	A	30	14.772	14.857	90.079	1.00	0.86	O
ATOM	239	CB	TYR	A	30	12.569	16.801	88.636	1.00	0.86	C

ATOM	240	CG	TYR	A	30	11.065	16.863	88.786	1.00	0.86	C
ATOM	241	CD1	TYR	A	30	10.251	15.913	88.146	1.00	0.86	C
ATOM	242	CD2	TYR	A	30	10.430	17.868	89.533	1.00	0.86	C
ATOM	243	CE1	TYR	A	30	8.853	15.978	88.237	1.00	0.86	C
ATOM	244	CE2	TYR	A	30	9.039	17.993	89.551	1.00	0.86	C
ATOM	245	CZ	TYR	A	30	8.250	17.050	88.898	1.00	0.86	C
ATOM	246	OH	TYR	A	30	6.854	17.195	88.902	1.00	0.86	O
ATOM	247	N	LEU	A	31	15.683	16.826	89.525	1.00	0.84	N
ATOM	248	CA	LEU	A	31	17.005	16.292	89.298	1.00	0.84	C
ATOM	249	C	LEU	A	31	17.968	17.105	90.145	1.00	0.84	C
ATOM	250	O	LEU	A	31	18.496	18.137	89.735	1.00	0.84	O
ATOM	251	CB	LEU	A	31	17.418	16.427	87.814	1.00	0.84	C
ATOM	252	CG	LEU	A	31	16.394	15.958	86.772	1.00	0.84	C
ATOM	253	CD1	LEU	A	31	16.862	16.527	85.423	1.00	0.84	C
ATOM	254	CD2	LEU	A	31	16.229	14.430	86.772	1.00	0.84	C
ATOM	255	N	GLN	A	32	18.234	16.653	91.382	1.00	0.85	N
ATOM	256	CA	GLN	A	32	18.857	17.499	92.382	1.00	0.85	C
ATOM	257	C	GLN	A	32	20.390	17.508	92.373	1.00	0.85	C
ATOM	258	O	GLN	A	32	21.044	18.365	92.965	1.00	0.85	O
ATOM	259	CB	GLN	A	32	18.364	16.978	93.745	1.00	0.85	C
ATOM	260	CG	GLN	A	32	16.823	16.987	93.879	1.00	0.85	C
ATOM	261	CD	GLN	A	32	16.353	16.170	95.077	1.00	0.85	C
ATOM	262	OE1	GLN	A	32	15.587	15.221	94.916	1.00	0.85	O
ATOM	263	NE2	GLN	A	32	16.816	16.518	96.290	1.00	0.85	N
ATOM	264	N	GLN	A	33	21.003	16.551	91.655	1.00	0.80	N
ATOM	265	CA	GLN	A	33	22.433	16.312	91.598	1.00	0.80	C
ATOM	266	C	GLN	A	33	23.020	16.912	90.325	1.00	0.80	C
ATOM	267	O	GLN	A	33	24.227	17.098	90.208	1.00	0.80	O
ATOM	268	CB	GLN	A	33	22.682	14.780	91.553	1.00	0.80	C
ATOM	269	CG	GLN	A	33	22.056	14.006	92.738	1.00	0.80	C
ATOM	270	CD	GLN	A	33	22.397	12.512	92.678	1.00	0.80	C
ATOM	271	OE1	GLN	A	33	23.553	12.138	92.493	1.00	0.80	O
ATOM	272	NE2	GLN	A	33	21.383	11.630	92.843	1.00	0.80	N
ATOM	273	N	SER	A	34	22.144	17.268	89.354	1.00	0.86	N
ATOM	274	CA	SER	A	34	22.481	17.850	88.053	1.00	0.86	C
ATOM	275	C	SER	A	34	23.097	19.240	88.272	1.00	0.86	C
ATOM	276	O	SER	A	34	22.555	19.974	89.098	1.00	0.86	O
ATOM	277	CB	SER	A	34	21.240	17.942	87.093	1.00	0.86	C
ATOM	278	OG	SER	A	34	21.532	18.297	85.740	1.00	0.86	O
ATOM	279	N	PRO	A	35	24.209	19.637	87.661	1.00	0.89	N
ATOM	280	CA	PRO	A	35	24.732	21.000	87.614	1.00	0.89	C
ATOM	281	C	PRO	A	35	23.769	22.067	87.170	1.00	0.89	C
ATOM	282	O	PRO	A	35	22.932	21.823	86.302	1.00	0.89	O
ATOM	283	CB	PRO	A	35	25.926	20.938	86.647	1.00	0.89	C
ATOM	284	CG	PRO	A	35	26.349	19.472	86.654	1.00	0.89	C
ATOM	285	CD	PRO	A	35	25.056	18.716	86.930	1.00	0.89	C
ATOM	286	N	PHE	A	36	23.961	23.295	87.694	1.00	0.90	N
ATOM	287	CA	PHE	A	36	23.163	24.461	87.382	1.00	0.90	C
ATOM	288	C	PHE	A	36	23.077	24.759	85.891	1.00	0.90	C
ATOM	289	O	PHE	A	36	21.989	24.948	85.354	1.00	0.90	O
ATOM	290	CB	PHE	A	36	23.784	25.660	88.153	1.00	0.90	C
ATOM	291	CG	PHE	A	36	23.120	26.985	87.879	1.00	0.90	C
ATOM	292	CD1	PHE	A	36	21.763	27.174	88.165	1.00	0.90	C
ATOM	293	CD2	PHE	A	36	23.829	28.030	87.262	1.00	0.90	C
ATOM	294	CE1	PHE	A	36	21.130	28.386	87.875	1.00	0.90	C
ATOM	295	CE2	PHE	A	36	23.207	29.257	87.001	1.00	0.90	C
ATOM	296	CZ	PHE	A	36	21.857	29.440	87.316	1.00	0.90	C
ATOM	297	N	ASP	A	37	24.217	24.744	85.178	1.00	0.87	N
ATOM	298	CA	ASP	A	37	24.316	25.108	83.785	1.00	0.87	C
ATOM	299	C	ASP	A	37	23.414	24.283	82.863	1.00	0.87	C
ATOM	300	O	ASP	A	37	22.851	24.798	81.888	1.00	0.87	O
ATOM	301	CB	ASP	A	37	25.802	25.005	83.361	1.00	0.87	C
ATOM	302	CG	ASP	A	37	26.617	26.231	83.780	1.00	0.87	C
ATOM	303	OD1	ASP	A	37	27.790	26.313	83.343	1.00	0.87	O
ATOM	304	OD2	ASP	A	37	26.091	27.106	84.523	1.00	0.87	O
ATOM	305	N	GLU	A	38	23.265	22.976	83.149	1.00	0.83	N
ATOM	306	CA	GLU	A	38	22.413	22.043	82.428	1.00	0.83	C
ATOM	307	C	GLU	A	38	20.954	22.428	82.555	1.00	0.83	C
ATOM	308	O	GLU	A	38	20.216	22.528	81.575	1.00	0.83	O
ATOM	309	CB	GLU	A	38	22.635	20.559	82.865	1.00	0.83	C
ATOM	310	CG	GLU	A	38	24.113	20.175	83.126	1.00	0.83	C
ATOM	311	CD	GLU	A	38	25.053	20.875	82.148	1.00	0.83	C
ATOM	312	OE1	GLU	A	38	24.800	20.868	80.913	1.00	0.83	O
ATOM	313	OE2	GLU	A	38	25.959	21.576	82.662	1.00	0.83	O
ATOM	314	N	HIS	A	39	20.532	22.752	83.790	1.00	0.85	N
ATOM	315	CA	HIS	A	39	19.203	23.245	84.106	1.00	0.85	C

ATOM	316	C	HIS	A	39	18.828	24.538	83.419	1.00	0.85	C
ATOM	317	O	HIS	A	39	17.708	24.683	82.933	1.00	0.85	O
ATOM	318	CB	HIS	A	39	19.005	23.362	85.617	1.00	0.85	C
ATOM	319	CG	HIS	A	39	18.836	22.012	86.188	1.00	0.85	C
ATOM	320	ND1	HIS	A	39	19.363	21.702	87.416	1.00	0.85	N
ATOM	321	CD2	HIS	A	39	18.025	21.018	85.749	1.00	0.85	C
ATOM	322	CE1	HIS	A	39	18.861	20.521	87.709	1.00	0.85	C
ATOM	323	NE2	HIS	A	39	18.031	20.063	86.736	1.00	0.85	N
ATOM	324	N	VAL	A	40	19.778	25.484	83.310	1.00	0.90	N
ATOM	325	CA	VAL	A	40	19.613	26.733	82.571	1.00	0.90	C
ATOM	326	C	VAL	A	40	19.303	26.507	81.094	1.00	0.90	C
ATOM	327	O	VAL	A	40	18.450	27.180	80.517	1.00	0.90	O
ATOM	328	CB	VAL	A	40	20.809	27.675	82.710	1.00	0.90	C
ATOM	329	CG1	VAL	A	40	20.524	29.043	82.053	1.00	0.90	C
ATOM	330	CG2	VAL	A	40	21.096	27.928	84.198	1.00	0.90	C
ATOM	331	N	LYS	A	41	19.960	25.524	80.440	1.00	0.82	N
ATOM	332	CA	LYS	A	41	19.651	25.116	79.071	1.00	0.82	C
ATOM	333	C	LYS	A	41	18.240	24.550	78.904	1.00	0.82	C
ATOM	334	O	LYS	A	41	17.504	24.932	77.993	1.00	0.82	O
ATOM	335	CB	LYS	A	41	20.670	24.062	78.580	1.00	0.82	C
ATOM	336	CG	LYS	A	41	22.078	24.639	78.354	1.00	0.82	C
ATOM	337	CD	LYS	A	41	23.161	23.546	78.260	1.00	0.82	C
ATOM	338	CE	LYS	A	41	24.599	24.034	78.493	1.00	0.82	C
ATOM	339	NZ	LYS	A	41	24.801	24.308	79.929	1.00	0.82	N
ATOM	340	N	LEU	A	42	17.820	23.656	79.825	1.00	0.88	N
ATOM	341	CA	LEU	A	42	16.491	23.061	79.806	1.00	0.88	C
ATOM	342	C	LEU	A	42	15.380	24.102	80.017	1.00	0.88	C
ATOM	343	O	LEU	A	42	14.337	24.066	79.363	1.00	0.88	O
ATOM	344	CB	LEU	A	42	16.295	21.860	80.785	1.00	0.88	C
ATOM	345	CG	LEU	A	42	17.380	20.754	80.906	1.00	0.88	C
ATOM	346	CD1	LEU	A	42	16.774	19.481	81.538	1.00	0.88	C
ATOM	347	CD2	LEU	A	42	18.074	20.381	79.588	1.00	0.88	C
ATOM	348	N	VAL	A	43	15.606	25.088	80.923	1.00	0.91	N
ATOM	349	CA	VAL	A	43	14.783	26.289	81.067	1.00	0.91	C
ATOM	350	C	VAL	A	43	14.769	27.186	79.840	1.00	0.91	C
ATOM	351	O	VAL	A	43	13.726	27.698	79.443	1.00	0.91	O
ATOM	352	CB	VAL	A	43	15.197	27.175	82.236	1.00	0.91	C
ATOM	353	CG1	VAL	A	43	14.277	28.411	82.356	1.00	0.91	C
ATOM	354	CG2	VAL	A	43	15.096	26.428	83.568	1.00	0.91	C
ATOM	355	N	ASN	A	44	15.920	27.451	79.195	1.00	0.87	N
ATOM	356	CA	ASN	A	44	15.924	28.290	78.011	1.00	0.87	C
ATOM	357	C	ASN	A	44	15.174	27.691	76.830	1.00	0.87	C
ATOM	358	O	ASN	A	44	14.359	28.372	76.216	1.00	0.87	O
ATOM	359	CB	ASN	A	44	17.343	28.687	77.565	1.00	0.87	C
ATOM	360	CG	ASN	A	44	17.966	29.677	78.545	1.00	0.87	C
ATOM	361	OD1	ASN	A	44	17.293	30.356	79.334	1.00	0.87	O
ATOM	362	ND2	ASN	A	44	19.305	29.838	78.422	1.00	0.87	N
ATOM	363	N	GLU	A	45	15.372	26.390	76.544	1.00	0.84	N
ATOM	364	CA	GLU	A	45	14.685	25.691	75.475	1.00	0.84	C
ATOM	365	C	GLU	A	45	13.168	25.611	75.676	1.00	0.84	C
ATOM	366	O	GLU	A	45	12.374	25.831	74.758	1.00	0.84	O
ATOM	367	CB	GLU	A	45	15.294	24.269	75.337	1.00	0.84	C
ATOM	368	CG	GLU	A	45	14.275	23.206	74.850	1.00	0.84	C
ATOM	369	CD	GLU	A	45	14.794	21.806	74.524	1.00	0.84	C
ATOM	370	OE1	GLU	A	45	14.682	21.405	73.341	1.00	0.84	O
ATOM	371	OE2	GLU	A	45	15.152	21.091	75.495	1.00	0.84	O
ATOM	372	N	ILE	A	46	12.701	25.297	76.909	1.00	0.89	N
ATOM	373	CA	ILE	A	46	11.271	25.213	77.202	1.00	0.89	C
ATOM	374	C	ILE	A	46	10.572	26.562	77.074	1.00	0.89	C
ATOM	375	O	ILE	A	46	9.423	26.649	76.638	1.00	0.89	O
ATOM	376	CB	ILE	A	46	10.991	24.507	78.534	1.00	0.89	C
ATOM	377	CG1	ILE	A	46	9.563	23.929	78.702	1.00	0.89	C
ATOM	378	CG2	ILE	A	46	11.299	25.423	79.718	1.00	0.89	C
ATOM	379	CD1	ILE	A	46	9.477	22.970	79.905	1.00	0.89	C
ATOM	380	N	THR	A	47	11.290	27.642	77.437	1.00	0.91	N
ATOM	381	CA	THR	A	47	10.916	29.050	77.309	1.00	0.91	C
ATOM	382	C	THR	A	47	10.807	29.559	75.873	1.00	0.91	C
ATOM	383	O	THR	A	47	9.900	30.327	75.553	1.00	0.91	O
ATOM	384	CB	THR	A	47	11.843	29.919	78.148	1.00	0.91	C
ATOM	385	OG1	THR	A	47	11.554	29.747	79.529	1.00	0.91	O
ATOM	386	CG2	THR	A	47	11.691	31.419	77.897	1.00	0.91	C
ATOM	387	N	ASP	A	48	11.697	29.138	74.947	1.00	0.88	N
ATOM	388	CA	ASP	A	48	11.561	29.415	73.519	1.00	0.88	C
ATOM	389	C	ASP	A	48	10.338	28.749	72.904	1.00	0.88	C
ATOM	390	O	ASP	A	48	9.564	29.343	72.156	1.00	0.88	O
ATOM	391	CB	ASP	A	48	12.796	28.926	72.731	1.00	0.88	C

ATOM	392	CG	ASP	A	48	14.020	29.804	72.947	1.00	0.88	C
ATOM	393	OD1	ASP	A	48	13.888	30.908	73.541	1.00	0.88	O
ATOM	394	OD2	ASP	A	48	15.104	29.376	72.476	1.00	0.88	O
ATOM	395	N	PHE	A	49	10.122	27.471	73.274	1.00	0.88	N
ATOM	396	CA	PHE	A	49	8.944	26.713	72.916	1.00	0.88	C
ATOM	397	C	PHE	A	49	7.643	27.382	73.425	1.00	0.88	C
ATOM	398	O	PHE	A	49	6.673	27.509	72.678	1.00	0.88	O
ATOM	399	CB	PHE	A	49	9.141	25.236	73.368	1.00	0.88	C
ATOM	400	CG	PHE	A	49	8.016	24.358	72.899	1.00	0.88	C
ATOM	401	CD1	PHE	A	49	7.886	24.022	71.545	1.00	0.88	C
ATOM	402	CD2	PHE	A	49	7.008	23.966	73.790	1.00	0.88	C
ATOM	403	CE1	PHE	A	49	6.738	23.377	71.074	1.00	0.88	C
ATOM	404	CE2	PHE	A	49	5.856	23.321	73.324	1.00	0.88	C
ATOM	405	CZ	PHE	A	49	5.708	23.061	71.959	1.00	0.88	C
ATOM	406	N	ALA	A	50	7.625	27.902	74.678	1.00	0.94	N
ATOM	407	CA	ALA	A	50	6.528	28.665	75.265	1.00	0.94	C
ATOM	408	C	ALA	A	50	6.177	29.949	74.499	1.00	0.94	C
ATOM	409	O	ALA	A	50	5.003	30.237	74.253	1.00	0.94	O
ATOM	410	CB	ALA	A	50	6.864	29.006	76.737	1.00	0.94	C
ATOM	411	N	LYS	A	51	7.194	30.728	74.059	1.00	0.83	N
ATOM	412	CA	LYS	A	51	7.018	31.900	73.199	1.00	0.83	C
ATOM	413	C	LYS	A	51	6.435	31.564	71.836	1.00	0.83	C
ATOM	414	O	LYS	A	51	5.567	32.270	71.323	1.00	0.83	O
ATOM	415	CB	LYS	A	51	8.331	32.666	72.909	1.00	0.83	C
ATOM	416	CG	LYS	A	51	8.986	33.350	74.112	1.00	0.83	C
ATOM	417	CD	LYS	A	51	10.474	33.596	73.808	1.00	0.83	C
ATOM	418	CE	LYS	A	51	11.322	33.879	75.028	1.00	0.83	C
ATOM	419	NZ	LYS	A	51	11.702	35.303	75.114	1.00	0.83	N
ATOM	420	N	THR	A	52	6.913	30.462	71.221	1.00	0.87	N
ATOM	421	CA	THR	A	52	6.380	29.919	69.967	1.00	0.87	C
ATOM	422	C	THR	A	52	4.930	29.529	70.076	1.00	0.87	C
ATOM	423	O	THR	A	52	4.142	29.861	69.193	1.00	0.87	O
ATOM	424	CB	THR	A	52	7.204	28.761	69.410	1.00	0.87	C
ATOM	425	OG1	THR	A	52	8.440	29.282	68.943	1.00	0.87	O
ATOM	426	CG2	THR	A	52	6.582	28.024	68.208	1.00	0.87	C
ATOM	427	N	CYS	A	53	4.522	28.873	71.182	1.00	0.89	N
ATOM	428	CA	CYS	A	53	3.129	28.532	71.414	1.00	0.89	C
ATOM	429	C	CYS	A	53	2.235	29.726	71.692	1.00	0.89	C
ATOM	430	O	CYS	A	53	1.053	29.704	71.377	1.00	0.89	O
ATOM	431	CB	CYS	A	53	2.949	27.486	72.531	1.00	0.89	C
ATOM	432	SG	CYS	A	53	3.729	25.892	72.155	1.00	0.89	S
ATOM	433	N	VAL	A	54	2.772	30.804	72.296	1.00	0.90	N
ATOM	434	CA	VAL	A	54	2.075	32.080	72.377	1.00	0.90	C
ATOM	435	C	VAL	A	54	1.850	32.722	71.014	1.00	0.90	C
ATOM	436	O	VAL	A	54	0.783	33.262	70.735	1.00	0.90	O
ATOM	437	CB	VAL	A	54	2.794	33.036	73.328	1.00	0.90	C
ATOM	438	CG1	VAL	A	54	2.617	34.538	73.004	1.00	0.90	C
ATOM	439	CG2	VAL	A	54	2.308	32.752	74.759	1.00	0.90	C
ATOM	440	N	ALA	A	55	2.873	32.685	70.134	1.00	0.90	N
ATOM	441	CA	ALA	A	55	2.778	33.183	68.776	1.00	0.90	C
ATOM	442	C	ALA	A	55	1.896	32.374	67.814	1.00	0.90	C
ATOM	443	O	ALA	A	55	1.117	32.948	67.054	1.00	0.90	O
ATOM	444	CB	ALA	A	55	4.194	33.348	68.191	1.00	0.90	C
ATOM	445	N	ASP	A	56	2.008	31.032	67.823	1.00	0.85	N
ATOM	446	CA	ASP	A	56	1.160	30.128	67.080	1.00	0.85	C
ATOM	447	C	ASP	A	56	0.711	29.015	68.034	1.00	0.85	C
ATOM	448	O	ASP	A	56	1.501	28.185	68.478	1.00	0.85	O
ATOM	449	CB	ASP	A	56	1.927	29.575	65.842	1.00	0.85	C
ATOM	450	CG	ASP	A	56	1.054	28.701	64.949	1.00	0.85	C
ATOM	451	OD1	ASP	A	56	-0.189	28.676	65.161	1.00	0.85	O
ATOM	452	OD2	ASP	A	56	1.638	28.012	64.076	1.00	0.85	O
ATOM	453	N	GLU	A	57	-0.599	28.970	68.362	1.00	0.80	N
ATOM	454	CA	GLU	A	57	-1.182	27.975	69.250	1.00	0.80	C
ATOM	455	C	GLU	A	57	-1.464	26.639	68.580	1.00	0.80	C
ATOM	456	O	GLU	A	57	-1.526	25.588	69.224	1.00	0.80	O
ATOM	457	CB	GLU	A	57	-2.462	28.551	69.893	1.00	0.80	C
ATOM	458	CG	GLU	A	57	-2.131	29.243	71.238	1.00	0.80	C
ATOM	459	CD	GLU	A	57	-3.348	29.855	71.930	1.00	0.80	C
ATOM	460	OE1	GLU	A	57	-4.219	30.432	71.230	1.00	0.80	O
ATOM	461	OE2	GLU	A	57	-3.432	29.712	73.180	1.00	0.80	O
ATOM	462	N	SER	A	58	-1.615	26.647	67.241	1.00	0.77	N
ATOM	463	CA	SER	A	58	-1.836	25.462	66.432	1.00	0.77	C
ATOM	464	C	SER	A	58	-0.575	24.688	66.195	1.00	0.77	C
ATOM	465	O	SER	A	58	-0.643	23.540	65.760	1.00	0.77	O
ATOM	466	CB	SER	A	58	-2.372	25.806	65.023	1.00	0.77	C
ATOM	467	OG	SER	A	58	-3.763	26.141	65.069	1.00	0.77	O

ATOM	468	N	ALA	A	59	0.604	25.291	66.451	1.00	0.84	N
ATOM	469	CA	ALA	A	59	1.876	24.644	66.227	1.00	0.84	C
ATOM	470	C	ALA	A	59	1.987	23.328	66.996	1.00	0.84	C
ATOM	471	O	ALA	A	59	1.446	23.148	68.090	1.00	0.84	O
ATOM	472	CB	ALA	A	59	3.065	25.585	66.535	1.00	0.84	C
ATOM	473	N	ALA	A	60	2.665	22.331	66.395	1.00	0.84	N
ATOM	474	CA	ALA	A	60	2.767	21.012	66.969	1.00	0.84	C
ATOM	475	C	ALA	A	60	3.336	21.013	68.381	1.00	0.84	C
ATOM	476	O	ALA	A	60	4.261	21.752	68.707	1.00	0.84	O
ATOM	477	CB	ALA	A	60	3.672	20.114	66.110	1.00	0.84	C
ATOM	478	N	ASN	A	61	2.746	20.195	69.265	1.00	0.82	N
ATOM	479	CA	ASN	A	61	3.186	20.019	70.640	1.00	0.82	C
ATOM	480	C	ASN	A	61	2.589	21.014	71.626	1.00	0.82	C
ATOM	481	O	ASN	A	61	2.609	20.753	72.826	1.00	0.82	O
ATOM	482	CB	ASN	A	61	4.719	19.875	70.923	1.00	0.82	C
ATOM	483	CG	ASN	A	61	5.363	18.694	70.220	1.00	0.82	C
ATOM	484	OD1	ASN	A	61	4.779	18.059	69.339	1.00	0.82	O
ATOM	485	ND2	ASN	A	61	6.557	18.281	70.731	1.00	0.82	N
ATOM	486	N	CYS	A	62	2.005	22.137	71.168	1.00	0.85	N
ATOM	487	CA	CYS	A	62	1.482	23.184	72.033	1.00	0.85	C
ATOM	488	C	CYS	A	62	0.311	22.763	72.934	1.00	0.85	C
ATOM	489	O	CYS	A	62	0.187	23.255	74.054	1.00	0.85	O
ATOM	490	CB	CYS	A	62	1.197	24.465	71.207	1.00	0.85	C
ATOM	491	SG	CYS	A	62	2.710	25.225	70.530	1.00	0.85	S
ATOM	492	N	ASP	A	63	−0.502	21.769	72.495	1.00	0.81	N
ATOM	493	CA	ASP	A	63	−1.640	21.238	73.225	1.00	0.81	C
ATOM	494	C	ASP	A	63	−1.226	20.062	74.138	1.00	0.81	C
ATOM	495	O	ASP	A	63	−2.043	19.484	74.854	1.00	0.81	O
ATOM	496	CB	ASP	A	63	−2.760	20.781	72.221	1.00	0.81	C
ATOM	497	CG	ASP	A	63	−3.390	21.913	71.394	1.00	0.81	C
ATOM	498	OD1	ASP	A	63	−3.333	23.093	71.815	1.00	0.81	O
ATOM	499	OD2	ASP	A	63	−3.969	21.616	70.311	1.00	0.81	O
ATOM	500	N	LYS	A	64	0.070	19.646	74.165	1.00	0.80	N
ATOM	501	CA	LYS	A	64	0.516	18.656	75.138	1.00	0.80	C
ATOM	502	C	LYS	A	64	0.488	19.191	76.559	1.00	0.80	C
ATOM	503	O	LYS	A	64	0.792	20.356	76.807	1.00	0.80	O
ATOM	504	CB	LYS	A	64	1.910	18.046	74.829	1.00	0.80	C
ATOM	505	CG	LYS	A	64	1.908	17.048	73.661	1.00	0.80	C
ATOM	506	CD	LYS	A	64	3.325	16.742	73.147	1.00	0.80	C
ATOM	507	CE	LYS	A	64	3.321	15.669	72.048	1.00	0.80	C
ATOM	508	NZ	LYS	A	64	4.148	16.048	70.893	1.00	0.80	N
ATOM	509	N	SER	A	65	0.125	18.329	77.540	1.00	0.83	N
ATOM	510	CA	SER	A	65	0.163	18.670	78.959	1.00	0.83	C
ATOM	511	C	SER	A	65	1.509	19.158	79.423	1.00	0.83	C
ATOM	512	O	SER	A	65	2.564	18.779	78.906	1.00	0.83	O
ATOM	513	CB	SER	A	65	−0.239	17.524	79.930	1.00	0.83	C
ATOM	514	OG	SER	A	65	−1.652	17.490	80.120	1.00	0.83	O
ATOM	515	N	LEU	A	66	1.508	20.013	80.464	1.00	0.85	N
ATOM	516	CA	LEU	A	66	2.733	20.502	81.050	1.00	0.85	C
ATOM	517	C	LEU	A	66	3.611	19.354	81.553	1.00	0.85	C
ATOM	518	O	LEU	A	66	4.805	19.340	81.257	1.00	0.85	O
ATOM	519	CB	LEU	A	66	2.457	21.626	82.086	1.00	0.85	C
ATOM	520	CG	LEU	A	66	2.187	22.997	81.422	1.00	0.85	C
ATOM	521	CD1	LEU	A	66	1.584	23.993	82.422	1.00	0.85	C
ATOM	522	CD2	LEU	A	66	3.467	23.582	80.798	1.00	0.85	C
ATOM	523	N	HIS	A	67	3.037	18.303	82.206	1.00	0.82	N
ATOM	524	CA	HIS	A	67	3.817	17.118	82.589	1.00	0.82	C
ATOM	525	C	HIS	A	67	4.513	16.430	81.438	1.00	0.82	C
ATOM	526	O	HIS	A	67	5.681	16.057	81.546	1.00	0.82	O
ATOM	527	CB	HIS	A	67	3.021	15.962	83.220	1.00	0.82	C
ATOM	528	CG	HIS	A	67	2.271	16.343	84.423	1.00	0.82	C
ATOM	529	ND1	HIS	A	67	0.923	16.067	84.457	1.00	0.82	N
ATOM	530	CD2	HIS	A	67	2.674	16.956	85.559	1.00	0.82	C
ATOM	531	CE1	HIS	A	67	0.523	16.534	85.626	1.00	0.82	C
ATOM	532	NE2	HIS	A	67	1.542	17.087	86.327	1.00	0.82	N
ATOM	533	N	THR	A	68	3.801	16.281	80.300	1.00	0.85	N
ATOM	534	CA	THR	A	68	4.329	15.710	79.077	1.00	0.85	C
ATOM	535	C	THR	A	68	5.579	16.456	78.626	1.00	0.85	C
ATOM	536	O	THR	A	68	6.665	15.899	78.544	1.00	0.85	O
ATOM	537	CB	THR	A	68	3.310	15.735	77.924	1.00	0.85	C
ATOM	538	OG1	THR	A	68	1.966	15.385	78.258	1.00	0.85	O
ATOM	539	CG2	THR	A	68	3.730	14.742	76.835	1.00	0.85	C
ATOM	540	N	LEU	A	69	5.483	17.788	78.440	1.00	0.85	N
ATOM	541	CA	LEU	A	69	6.579	18.620	77.969	1.00	0.85	C
ATOM	542	C	LEU	A	69	7.775	18.758	78.913	1.00	0.85	C
ATOM	543	O	LEU	A	69	8.915	18.905	78.457	1.00	0.85	O

ATOM	544	CB	LEU	A	69	6.087	20.046	77.655	1.00	0.85	C
ATOM	545	CG	LEU	A	69	5.017	20.160	76.555	1.00	0.85	C
ATOM	546	CD1	LEU	A	69	4.334	21.529	76.690	1.00	0.85	C
ATOM	547	CD2	LEU	A	69	5.586	19.956	75.140	1.00	0.85	C
ATOM	548	N	PHE	A	70	7.521	18.800	80.246	1.00	0.85	N
ATOM	549	CA	PHE	A	70	8.538	18.761	81.293	1.00	0.85	C
ATOM	550	C	PHE	A	70	9.257	17.420	81.297	1.00	0.85	C
ATOM	551	O	PHE	A	70	10.485	17.345	81.290	1.00	0.85	O
ATOM	552	CB	PHE	A	70	7.910	18.966	82.709	1.00	0.85	C
ATOM	553	CG	PHE	A	70	7.922	20.391	83.190	1.00	0.85	C
ATOM	554	CD1	PHE	A	70	9.113	20.927	83.690	1.00	0.85	C
ATOM	555	CD2	PHE	A	70	6.757	21.174	83.269	1.00	0.85	C
ATOM	556	CE1	PHE	A	70	9.155	22.220	84.211	1.00	0.85	C
ATOM	557	CE2	PHE	A	70	6.792	22.478	83.788	1.00	0.85	C
ATOM	558	CZ	PHE	A	70	7.999	22.999	84.259	1.00	0.85	C
ATOM	559	N	GLY	A	71	8.471	16.330	81.257	1.00	0.87	N
ATOM	560	CA	GLY	A	71	8.920	14.945	81.276	1.00	0.87	C
ATOM	561	C	GLY	A	71	9.681	14.491	80.052	1.00	0.87	C
ATOM	562	O	GLY	A	71	10.644	13.734	80.163	1.00	0.87	O
ATOM	563	N	ASP	A	72	9.292	14.973	78.851	1.00	0.84	N
ATOM	564	CA	ASP	A	72	9.976	14.741	77.582	1.00	0.84	C
ATOM	565	C	ASP	A	72	11.408	15.261	77.633	1.00	0.84	C
ATOM	566	O	ASP	A	72	12.360	14.586	77.244	1.00	0.84	O
ATOM	567	CB	ASP	A	72	9.197	15.388	76.396	1.00	0.84	C
ATOM	568	CG	ASP	A	72	7.879	14.671	76.091	1.00	0.84	C
ATOM	569	OD1	ASP	A	72	7.579	13.619	76.730	1.00	0.84	O
ATOM	570	OD2	ASP	A	72	7.157	15.193	75.199	1.00	0.84	O
ATOM	571	N	LYS	A	73	11.586	16.471	78.193	1.00	0.79	N
ATOM	572	CA	LYS	A	73	12.884	17.072	78.429	1.00	0.79	C
ATOM	573	C	LYS	A	73	13.734	16.384	79.466	1.00	0.79	C
ATOM	574	O	LYS	A	73	14.947	16.265	79.305	1.00	0.79	O
ATOM	575	CB	LYS	A	73	12.758	18.558	78.752	1.00	0.79	C
ATOM	576	CG	LYS	A	73	12.237	19.275	77.517	1.00	0.79	C
ATOM	577	CD	LYS	A	73	12.199	20.781	77.726	1.00	0.79	C
ATOM	578	CE	LYS	A	73	11.548	21.472	76.543	1.00	0.79	C
ATOM	579	NZ	LYS	A	73	10.113	21.131	76.532	1.00	0.79	N
ATOM	580	N	LEU	A	74	13.120	15.888	80.560	1.00	0.79	N
ATOM	581	CA	LEU	A	74	13.843	15.084	81.533	1.00	0.79	C
ATOM	582	C	LEU	A	74	14.400	13.794	80.964	1.00	0.79	C
ATOM	583	O	LEU	A	74	15.486	13.352	81.325	1.00	0.79	O
ATOM	584	CB	LEU	A	74	13.022	14.671	82.759	1.00	0.79	C
ATOM	585	CG	LEU	A	74	12.478	15.807	83.618	1.00	0.79	C
ATOM	586	CD1	LEU	A	74	11.727	15.190	84.784	1.00	0.79	C
ATOM	587	CD2	LEU	A	74	13.548	16.674	84.275	1.00	0.79	C
ATOM	588	N	CYS	A	75	13.648	13.160	80.051	1.00	0.79	N
ATOM	589	CA	CYS	A	75	14.042	11.917	79.421	1.00	0.79	C
ATOM	590	C	CYS	A	75	15.133	12.032	78.368	1.00	0.79	C
ATOM	591	O	CYS	A	75	15.755	11.030	78.025	1.00	0.79	O
ATOM	592	CB	CYS	A	75	12.816	11.220	78.792	1.00	0.79	C
ATOM	593	SG	CYS	A	75	11.721	10.534	80.061	1.00	0.79	S
ATOM	594	N	ALA	A	76	15.432	13.247	77.863	1.00	0.80	N
ATOM	595	CA	ALA	A	76	16.537	13.472	76.955	1.00	0.80	C
ATOM	596	C	ALA	A	76	17.817	13.885	77.683	1.00	0.80	C
ATOM	597	O	ALA	A	76	18.835	14.189	77.060	1.00	0.80	O
ATOM	598	CB	ALA	A	76	16.150	14.582	75.962	1.00	0.80	C
ATOM	599	N	VAL	A	77	17.836	13.885	79.036	1.00	0.78	N
ATOM	600	CA	VAL	A	77	19.062	14.121	79.784	1.00	0.78	C
ATOM	601	C	VAL	A	77	20.012	12.935	79.613	1.00	0.78	C
ATOM	602	O	VAL	A	77	19.721	11.812	80.020	1.00	0.78	O
ATOM	603	CB	VAL	A	77	18.837	14.402	81.273	1.00	0.78	C
ATOM	604	CG1	VAL	A	77	20.166	14.713	82.002	1.00	0.78	C
ATOM	605	CG2	VAL	A	77	17.876	15.597	81.431	1.00	0.78	C
ATOM	606	N	ALA	A	78	21.204	13.176	79.021	1.00	0.63	N
ATOM	607	CA	ALA	A	78	22.190	12.165	78.652	1.00	0.63	C
ATOM	608	C	ALA	A	78	22.642	11.241	79.778	1.00	0.63	C
ATOM	609	O	ALA	A	78	22.931	10.062	79.590	1.00	0.63	O
ATOM	610	CB	ALA	A	78	23.426	12.880	78.062	1.00	0.63	C
ATOM	611	N	SER	A	79	22.674	11.798	80.994	1.00	0.73	N
ATOM	612	CA	SER	A	79	23.239	11.199	82.173	1.00	0.73	C
ATOM	613	C	SER	A	79	22.164	10.642	83.107	1.00	0.73	C
ATOM	614	O	SER	A	79	22.458	10.279	84.239	1.00	0.73	O
ATOM	615	CB	SER	A	79	24.118	12.260	82.904	1.00	0.73	C
ATOM	616	OG	SER	A	79	25.353	12.511	82.227	1.00	0.73	O
ATOM	617	N	LEU	A	80	20.870	10.527	82.700	1.00	0.75	N
ATOM	618	CA	LEU	A	80	19.812	10.129	83.629	1.00	0.75	C
ATOM	619	C	LEU	A	80	20.020	8.780	84.343	1.00	0.75	C

ATOM	620	O	LEU	A	80	19.885	8.673	85.556	1.00	0.75	O
ATOM	621	CB	LEU	A	80	18.434	10.147	82.926	1.00	0.75	C
ATOM	622	CG	LEU	A	80	17.184	10.335	83.814	1.00	0.75	C
ATOM	623	CD1	LEU	A	80	17.175	11.655	84.603	1.00	0.75	C
ATOM	624	CD2	LEU	A	80	15.984	10.309	82.863	1.00	0.75	C
ATOM	625	N	ARG	A	81	20.420	7.712	83.628	1.00	0.70	N
ATOM	626	CA	ARG	A	81	20.700	6.429	84.254	1.00	0.70	C
ATOM	627	C	ARG	A	81	21.975	6.324	85.096	1.00	0.70	C
ATOM	628	O	ARG	A	81	21.963	5.748	86.182	1.00	0.70	O
ATOM	629	CB	ARG	A	81	20.791	5.354	83.159	1.00	0.70	C
ATOM	630	CG	ARG	A	81	21.091	3.930	83.675	1.00	0.70	C
ATOM	631	CD	ARG	A	81	21.306	2.947	82.524	1.00	0.70	C
ATOM	632	NE	ARG	A	81	22.211	1.841	83.002	1.00	0.70	N
ATOM	633	CZ	ARG	A	81	23.506	1.695	82.681	1.00	0.70	C
ATOM	634	NH1	ARG	A	81	24.160	2.561	81.910	1.00	0.70	N
ATOM	635	NH2	ARG	A	81	24.187	0.652	83.159	1.00	0.70	N
ATOM	636	N	GLU	A	82	23.127	6.833	84.613	1.00	0.72	N
ATOM	637	CA	GLU	A	82	24.397	6.668	85.302	1.00	0.72	C
ATOM	638	C	GLU	A	82	24.607	7.699	86.413	1.00	0.72	C
ATOM	639	O	GLU	A	82	25.572	7.615	87.172	1.00	0.72	O
ATOM	640	CB	GLU	A	82	25.549	6.653	84.270	1.00	0.72	C
ATOM	641	CG	GLU	A	82	25.752	7.944	83.450	1.00	0.72	C
ATOM	642	CD	GLU	A	82	26.720	7.672	82.296	1.00	0.72	C
ATOM	643	OE1	GLU	A	82	27.816	7.116	82.557	1.00	0.72	O
ATOM	644	OE2	GLU	A	82	26.332	7.974	81.140	1.00	0.72	O
ATOM	645	N	THR	A	83	23.650	8.649	86.571	1.00	0.80	N
ATOM	646	CA	THR	A	83	23.599	9.596	87.686	1.00	0.80	C
ATOM	647	C	THR	A	83	22.409	9.349	88.619	1.00	0.80	C
ATOM	648	O	THR	A	83	22.511	9.590	89.820	1.00	0.80	O
ATOM	649	CB	THR	A	83	23.539	11.034	87.170	1.00	0.80	C
ATOM	650	OG1	THR	A	83	24.697	11.341	86.407	1.00	0.80	O
ATOM	651	CG2	THR	A	83	23.467	12.128	88.252	1.00	0.80	C
ATOM	652	N	TYR	A	84	21.247	8.829	88.131	1.00	0.76	N
ATOM	653	CA	TYR	A	84	20.048	8.676	88.955	1.00	0.76	C
ATOM	654	C	TYR	A	84	19.455	7.257	88.996	1.00	0.76	C
ATOM	655	O	TYR	A	84	18.384	7.035	89.565	1.00	0.76	O
ATOM	656	CB	TYR	A	84	18.911	9.616	88.490	1.00	0.76	C
ATOM	657	CG	TYR	A	84	19.283	11.054	88.593	1.00	0.76	C
ATOM	658	CD1	TYR	A	84	18.926	11.775	89.738	1.00	0.76	C
ATOM	659	CD2	TYR	A	84	19.933	11.716	87.542	1.00	0.76	C
ATOM	660	CE1	TYR	A	84	19.237	13.132	89.850	1.00	0.76	C
ATOM	661	CE2	TYR	A	84	20.236	13.078	87.647	1.00	0.76	C
ATOM	662	CZ	TYR	A	84	19.907	13.773	88.813	1.00	0.76	C
ATOM	663	OH	TYR	A	84	20.274	15.113	88.965	1.00	0.76	O
ATOM	664	N	GLY	A	85	20.136	6.237	88.432	1.00	0.81	N
ATOM	665	CA	GLY	A	85	19.695	4.834	88.433	1.00	0.81	C
ATOM	666	C	GLY	A	85	18.307	4.513	87.875	1.00	0.81	C
ATOM	667	O	GLY	A	85	17.980	4.894	86.752	1.00	0.81	O
ATOM	668	N	GLU	A	86	17.471	3.772	88.653	1.00	0.71	N
ATOM	669	CA	GLU	A	86	16.093	3.352	88.362	1.00	0.71	C
ATOM	670	C	GLU	A	86	15.089	4.455	87.970	1.00	0.71	C
ATOM	671	O	GLU	A	86	13.998	4.210	87.457	1.00	0.71	O
ATOM	672	CB	GLU	A	86	15.539	2.623	89.614	1.00	0.71	C
ATOM	673	CG	GLU	A	86	16.164	1.230	89.903	1.00	0.71	C
ATOM	674	CD	GLU	A	86	15.579	0.562	91.154	1.00	0.71	C
ATOM	675	OE1	GLU	A	86	14.790	1.233	91.870	1.00	0.71	O
ATOM	676	OE2	GLU	A	86	15.941	−0.615	91.412	1.00	0.71	O
ATOM	677	N	MET	A	87	15.452	5.739	88.152	1.00	0.77	N
ATOM	678	CA	MET	A	87	14.729	6.890	87.642	1.00	0.77	C
ATOM	679	C	MET	A	87	14.641	6.942	86.122	1.00	0.77	C
ATOM	680	O	MET	A	87	13.681	7.449	85.545	1.00	0.77	O
ATOM	681	CB	MET	A	87	15.406	8.162	88.179	1.00	0.77	C
ATOM	682	CG	MET	A	87	14.810	9.493	87.691	1.00	0.77	C
ATOM	683	SD	MET	A	87	15.341	10.901	88.711	1.00	0.77	S
ATOM	684	CE	MET	A	87	14.039	12.004	88.114	1.00	0.77	C
ATOM	685	N	ALA	A	88	15.634	6.392	85.397	1.00	0.81	N
ATOM	686	CA	ALA	A	88	15.606	6.430	83.954	1.00	0.81	C
ATOM	687	C	ALA	A	88	14.618	5.432	83.340	1.00	0.81	C
ATOM	688	O	ALA	A	88	14.290	5.526	82.157	1.00	0.81	O
ATOM	689	CB	ALA	A	88	17.026	6.322	83.377	1.00	0.81	C
ATOM	690	N	ASP	A	89	14.048	4.509	84.151	1.00	0.79	N
ATOM	691	CA	ASP	A	89	13.017	3.583	83.727	1.00	0.79	C
ATOM	692	C	ASP	A	89	11.648	4.242	83.803	1.00	0.79	C
ATOM	693	O	ASP	A	89	10.647	3.749	83.276	1.00	0.79	O
ATOM	694	CB	ASP	A	89	13.039	2.304	84.587	1.00	0.79	C
ATOM	695	CG	ASP	A	89	14.447	1.730	84.583	1.00	0.79	C

ATOM	696	OD1	ASP	A	89	15.008	1.535	83.475	1.00	0.79	O
ATOM	697	OD2	ASP	A	89	14.980	1.503	85.695	1.00	0.79	O
ATOM	698	N	CYS	A	90	11.575	5.452	84.412	1.00	0.83	N
ATOM	699	CA	CYS	A	90	10.411	6.316	84.347	1.00	0.83	C
ATOM	700	C	CYS	A	90	10.111	6.676	82.901	1.00	0.83	C
ATOM	701	O	CYS	A	90	8.968	6.668	82.462	1.00	0.83	O
ATOM	702	CB	CYS	A	90	10.529	7.615	85.192	1.00	0.83	C
ATOM	703	SG	CYS	A	90	10.798	7.360	86.976	1.00	0.83	S
ATOM	704	N	CYS	A	91	11.172	6.919	82.108	1.00	0.82	N
ATOM	705	CA	CYS	A	91	11.134	7.221	80.688	1.00	0.82	C
ATOM	706	C	CYS	A	91	10.589	6.143	79.777	1.00	0.82	C
ATOM	707	O	CYS	A	91	10.310	6.413	78.611	1.00	0.82	O
ATOM	708	CB	CYS	A	91	12.531	7.664	80.215	1.00	0.82	C
ATOM	709	SG	CYS	A	91	12.953	9.235	81.013	1.00	0.82	S
ATOM	710	N	GLY	A	92	10.385	4.916	80.297	1.00	0.85	N
ATOM	711	CA	GLY	A	92	9.677	3.858	79.592	1.00	0.85	C
ATOM	712	C	GLY	A	92	8.179	3.846	79.821	1.00	0.85	C
ATOM	713	O	GLY	A	92	7.474	3.000	79.280	1.00	0.85	O
ATOM	714	N	LYS	A	93	7.648	4.757	80.662	1.00	0.80	N
ATOM	715	CA	LYS	A	93	6.246	4.817	81.033	1.00	0.80	C
ATOM	716	C	LYS	A	93	5.509	5.907	80.273	1.00	0.80	C
ATOM	717	O	LYS	A	93	6.104	6.857	79.770	1.00	0.80	O
ATOM	718	CB	LYS	A	93	6.096	5.143	82.535	1.00	0.80	C
ATOM	719	CG	LYS	A	93	6.782	4.117	83.440	1.00	0.80	C
ATOM	720	CD	LYS	A	93	6.725	4.564	84.908	1.00	0.80	C
ATOM	721	CE	LYS	A	93	7.132	3.503	85.926	1.00	0.80	C
ATOM	722	NZ	LYS	A	93	6.230	2.344	85.780	1.00	0.80	N
ATOM	723	N	GLN	A	94	4.166	5.806	80.208	1.00	0.80	N
ATOM	724	CA	GLN	A	94	3.307	6.825	79.651	1.00	0.80	C
ATOM	725	C	GLN	A	94	2.988	7.846	80.735	1.00	0.80	C
ATOM	726	O	GLN	A	94	3.307	7.645	81.907	1.00	0.80	O
ATOM	727	CB	GLN	A	94	1.986	6.192	79.140	1.00	0.80	C
ATOM	728	CG	GLN	A	94	2.182	5.107	78.052	1.00	0.80	C
ATOM	729	CD	GLN	A	94	0.872	4.367	77.763	1.00	0.80	C
ATOM	730	OE1	GLN	A	94	−0.226	4.841	78.040	1.00	0.80	O
ATOM	731	NE2	GLN	A	94	0.978	3.148	77.175	1.00	0.80	N
ATOM	732	N	ASP	A	95	2.379	8.985	80.370	1.00	0.84	N
ATOM	733	CA	ASP	A	95	1.771	9.901	81.304	1.00	0.84	C
ATOM	734	C	ASP	A	95	0.401	9.379	81.768	1.00	0.84	C
ATOM	735	O	ASP	A	95	−0.371	8.940	80.913	1.00	0.84	O
ATOM	736	CB	ASP	A	95	1.621	11.263	80.595	1.00	0.84	C
ATOM	737	CG	ASP	A	95	3.008	11.831	80.326	1.00	0.84	C
ATOM	738	OD1	ASP	A	95	3.304	12.137	79.144	1.00	0.84	O
ATOM	739	OD2	ASP	A	95	3.814	11.951	81.288	1.00	0.84	O
ATOM	740	N	PRO	A	96	0.029	9.400	83.054	1.00	0.86	N
ATOM	741	CA	PRO	A	96	0.645	10.202	84.097	1.00	0.86	C
ATOM	742	C	PRO	A	96	1.652	9.365	84.879	1.00	0.86	C
ATOM	743	O	PRO	A	96	2.380	9.939	85.690	1.00	0.86	O
ATOM	744	CB	PRO	A	96	−0.550	10.658	84.953	1.00	0.86	C
ATOM	745	CG	PRO	A	96	−1.538	9.495	84.860	1.00	0.86	C
ATOM	746	CD	PRO	A	96	−1.308	8.946	83.448	1.00	0.86	C
ATOM	747	N	GLU	A	97	1.752	8.030	84.658	1.00	0.81	N
ATOM	748	CA	GLU	A	97	2.576	7.123	85.449	1.00	0.81	C
ATOM	749	C	GLU	A	97	4.043	7.503	85.491	1.00	0.81	C
ATOM	750	O	GLU	A	97	4.746	7.315	86.486	1.00	0.81	O
ATOM	751	CB	GLU	A	97	2.531	5.667	84.926	1.00	0.81	C
ATOM	752	CG	GLU	A	97	1.183	4.955	85.171	1.00	0.81	C
ATOM	753	CD	GLU	A	97	0.175	5.159	84.039	1.00	0.81	C
ATOM	754	OE1	GLU	A	97	−0.884	4.490	84.103	1.00	0.81	O
ATOM	755	OE2	GLU	A	97	0.453	5.979	83.127	1.00	0.81	O
ATOM	756	N	ARG	A	98	4.533	8.043	84.366	1.00	0.77	N
ATOM	757	CA	ARG	A	98	5.846	8.621	84.213	1.00	0.77	C
ATOM	758	C	ARG	A	98	6.142	9.789	85.143	1.00	0.77	C
ATOM	759	O	ARG	A	98	7.208	9.832	85.750	1.00	0.77	O
ATOM	760	CB	ARG	A	98	6.058	9.076	82.755	1.00	0.77	C
ATOM	761	CG	ARG	A	98	7.486	9.575	82.477	1.00	0.77	C
ATOM	762	CD	ARG	A	98	7.798	9.777	80.995	1.00	0.77	C
ATOM	763	NE	ARG	A	98	7.114	11.053	80.607	1.00	0.77	N
ATOM	764	CZ	ARG	A	98	7.392	11.801	79.530	1.00	0.77	C
ATOM	765	NH1	ARG	A	98	8.317	11.481	78.625	1.00	0.77	N
ATOM	766	NH2	ARG	A	98	6.660	12.881	79.267	1.00	0.77	N
ATOM	767	N	ASN	A	99	5.201	10.744	85.305	1.00	0.84	N
ATOM	768	CA	ASN	A	99	5.331	11.863	86.224	1.00	0.84	C
ATOM	769	C	ASN	A	99	5.357	11.415	87.699	1.00	0.84	C
ATOM	770	O	ASN	A	99	6.204	11.854	88.477	1.00	0.84	O
ATOM	771	CB	ASN	A	99	4.257	12.945	85.932	1.00	0.84	C

```
ATOM    772  CG  ASN A  99       4.620  14.219  86.690  1.00  0.84           C
ATOM    773  OD1 ASN A  99       5.699  14.765  86.448  1.00  0.84           O
ATOM    774  ND2 ASN A  99       3.752  14.694  87.608  1.00  0.84           N
ATOM    775  N   GLU A 100       4.478  10.458  88.088  1.00  0.82           N
ATOM    776  CA  GLU A 100       4.410   9.890  89.435  1.00  0.82           C
ATOM    777  C   GLU A 100       5.717   9.239  89.852  1.00  0.82           C
ATOM    778  O   GLU A 100       6.176   9.322  90.992  1.00  0.82           O
ATOM    779  CB  GLU A 100       3.336   8.768  89.555  1.00  0.82           C
ATOM    780  CG  GLU A 100       1.862   9.141  89.248  1.00  0.82           C
ATOM    781  CD  GLU A 100       1.267  10.186  90.191  1.00  0.82           C
ATOM    782  OE1 GLU A 100       1.905  10.498  91.230  1.00  0.82           O
ATOM    783  OE2 GLU A 100       0.151  10.669  89.872  1.00  0.82           O
ATOM    784  N   CYS A 101       6.349   8.548  88.887  1.00  0.87           N
ATOM    785  CA  CYS A 101       7.640   7.909  89.017  1.00  0.87           C
ATOM    786  C   CYS A 101       8.742   8.898  89.353  1.00  0.87           C
ATOM    787  O   CYS A 101       9.452   8.709  90.333  1.00  0.87           O
ATOM    788  CB  CYS A 101       7.874   7.126  87.699  1.00  0.87           C
ATOM    789  SG  CYS A 101       9.346   6.079  87.567  1.00  0.87           S
ATOM    790  N   LEU A 102       8.828  10.032  88.631  1.00  0.83           N
ATOM    791  CA  LEU A 102       9.812  11.067  88.881  1.00  0.83           C
ATOM    792  C   LEU A 102       9.681  11.713  90.261  1.00  0.83           C
ATOM    793  O   LEU A 102      10.665  11.977  90.952  1.00  0.83           O
ATOM    794  CB  LEU A 102       9.656  12.163  87.817  1.00  0.83           C
ATOM    795  CG  LEU A 102       9.869  11.750  86.353  1.00  0.83           C
ATOM    796  CD1 LEU A 102       9.305  12.893  85.504  1.00  0.83           C
ATOM    797  CD2 LEU A 102      11.344  11.485  86.032  1.00  0.83           C
ATOM    798  N   LEU A 103       8.432  11.970  90.705  1.00  0.84           N
ATOM    799  CA  LEU A 103       8.149  12.520  92.022  1.00  0.84           C
ATOM    800  C   LEU A 103       8.575  11.642  93.194  1.00  0.84           C
ATOM    801  O   LEU A 103       9.032  12.125  94.228  1.00  0.84           O
ATOM    802  CB  LEU A 103       6.644  12.724  92.259  1.00  0.84           C
ATOM    803  CG  LEU A 103       5.911  13.737  91.376  1.00  0.84           C
ATOM    804  CD1 LEU A 103       4.503  13.934  91.943  1.00  0.84           C
ATOM    805  CD2 LEU A 103       6.610  15.093  91.306  1.00  0.84           C
ATOM    806  N   LYS A 104       8.400  10.315  93.064  1.00  0.79           N
ATOM    807  CA  LYS A 104       8.756   9.355  94.089  1.00  0.79           C
ATOM    808  C   LYS A 104      10.267   9.194  94.262  1.00  0.79           C
ATOM    809  O   LYS A 104      10.717   8.628  95.256  1.00  0.79           O
ATOM    810  CB  LYS A 104       8.069   8.000  93.815  1.00  0.79           C
ATOM    811  CG  LYS A 104       6.548   8.012  94.056  1.00  0.79           C
ATOM    812  CD  LYS A 104       5.958   6.608  93.848  1.00  0.79           C
ATOM    813  CE  LYS A 104       4.447   6.542  94.076  1.00  0.79           C
ATOM    814  NZ  LYS A 104       3.952   5.168  93.834  1.00  0.79           N
ATOM    815  N   HIS A 105      11.077   9.727  93.320  1.00  0.79           N
ATOM    816  CA  HIS A 105      12.520   9.826  93.456  1.00  0.79           C
ATOM    817  C   HIS A 105      13.005  11.197  93.929  1.00  0.79           C
ATOM    818  O   HIS A 105      14.211  11.440  93.953  1.00  0.79           O
ATOM    819  CB  HIS A 105      13.269   9.469  92.148  1.00  0.79           C
ATOM    820  CG  HIS A 105      13.138   8.032  91.756  1.00  0.79           C
ATOM    821  ND1 HIS A 105      12.348   7.678  90.684  1.00  0.79           N
ATOM    822  CD2 HIS A 105      13.688   6.925  92.319  1.00  0.79           C
ATOM    823  CE1 HIS A 105      12.420   6.366  90.619  1.00  0.79           C
ATOM    824  NE2 HIS A 105      13.222   5.856  91.585  1.00  0.79           N
ATOM    825  N   LYS A 106      12.117  12.130  94.360  1.00  0.82           N
ATOM    826  CA  LYS A 106      12.554  13.302  95.108  1.00  0.82           C
ATOM    827  C   LYS A 106      13.236  12.896  96.408  1.00  0.82           C
ATOM    828  O   LYS A 106      12.685  12.143  97.210  1.00  0.82           O
ATOM    829  CB  LYS A 106      11.383  14.267  95.451  1.00  0.82           C
ATOM    830  CG  LYS A 106      10.899  15.145  94.283  1.00  0.82           C
ATOM    831  CD  LYS A 106       9.816  16.152  94.711  1.00  0.82           C
ATOM    832  CE  LYS A 106       9.321  17.044  93.567  1.00  0.82           C
ATOM    833  NZ  LYS A 106       8.418  18.081  94.117  1.00  0.82           N
ATOM    834  N   ASP A 107      14.462  13.401  96.642  1.00  0.87           N
ATOM    835  CA  ASP A 107      15.235  13.043  97.803  1.00  0.87           C
ATOM    836  C   ASP A 107      15.124  14.107  98.891  1.00  0.87           C
ATOM    837  O   ASP A 107      15.668  15.208  98.797  1.00  0.87           O
ATOM    838  CB  ASP A 107      16.713  12.763  97.439  1.00  0.87           C
ATOM    839  CG  ASP A 107      17.393  11.950  98.541  1.00  0.87           C
ATOM    840  OD1 ASP A 107      16.885  11.920  99.707  1.00  0.87           O
ATOM    841  OD2 ASP A 107      18.431  11.330  98.228  1.00  0.87           O
ATOM    842  N   ASP A 108      14.451  13.743 100.004  1.00  0.85           N
ATOM    843  CA  ASP A 108      14.340  14.541 101.204  1.00  0.85           C
ATOM    844  C   ASP A 108      15.672  14.662 101.981  1.00  0.85           C
ATOM    845  O   ASP A 108      15.794  15.492 102.881  1.00  0.85           O
ATOM    846  CB  ASP A 108      13.167  14.015 102.101  1.00  0.85           C
ATOM    847  CG  ASP A 108      11.796  14.531 101.657  1.00  0.85           C
```

ATOM	848	OD1	ASP	A	108	11.634	15.777	101.646	1.00	0.85	O
ATOM	849	OD2	ASP	A	108	10.845	13.739	101.439	1.00	0.85	O
ATOM	850	N	ASN	A	109	16.736	13.877	101.675	1.00	0.80	N
ATOM	851	CA	ASN	A	109	18.009	14.015	102.380	1.00	0.80	C
ATOM	852	C	ASN	A	109	19.183	14.007	101.384	1.00	0.80	C
ATOM	853	O	ASN	A	109	19.934	13.034	101.387	1.00	0.80	O
ATOM	854	CB	ASN	A	109	18.144	12.921	103.484	1.00	0.80	C
ATOM	855	CG	ASN	A	109	19.364	13.186	104.363	1.00	0.80	C
ATOM	856	OD1	ASN	A	109	19.799	14.328	104.501	1.00	0.80	O
ATOM	857	ND2	ASN	A	109	19.936	12.120	104.969	1.00	0.80	N
ATOM	858	N	PRO	A	110	19.390	15.029	100.535	1.00	0.85	N
ATOM	859	CA	PRO	A	110	20.142	14.887	99.283	1.00	0.85	C
ATOM	860	C	PRO	A	110	21.658	14.710	99.329	1.00	0.85	C
ATOM	861	O	PRO	A	110	22.237	14.688	98.249	1.00	0.85	O
ATOM	862	CB	PRO	A	110	19.836	16.150	98.462	1.00	0.85	C
ATOM	863	CG	PRO	A	110	18.631	16.805	99.121	1.00	0.85	C
ATOM	864	CD	PRO	A	110	18.582	16.256	100.539	1.00	0.85	C
ATOM	865	N	ASP	A	111	22.310	14.725	100.515	1.00	0.77	N
ATOM	866	CA	ASP	A	111	23.757	14.582	100.705	1.00	0.77	C
ATOM	867	C	ASP	A	111	24.528	15.910	100.599	1.00	0.77	C
ATOM	868	O	ASP	A	111	25.719	16.016	100.889	1.00	0.77	O
ATOM	869	CB	ASP	A	111	24.343	13.310	99.982	1.00	0.77	C
ATOM	870	CG	ASP	A	111	25.550	13.444	99.043	1.00	0.77	C
ATOM	871	OD1	ASP	A	111	26.539	12.700	99.285	1.00	0.77	O
ATOM	872	OD2	ASP	A	111	25.486	14.231	98.065	1.00	0.77	O
ATOM	873	N	LEU	A	112	23.829	17.010	100.240	1.00	0.83	N
ATOM	874	CA	LEU	A	112	24.508	18.100	99.553	1.00	0.83	C
ATOM	875	C	LEU	A	112	25.544	18.967	100.262	1.00	0.83	C
ATOM	876	O	LEU	A	112	25.273	19.400	101.391	1.00	0.83	O
ATOM	877	CB	LEU	A	112	23.528	19.001	98.777	1.00	0.83	C
ATOM	878	CG	LEU	A	112	22.974	18.343	97.495	1.00	0.83	C
ATOM	879	CD1	LEU	A	112	21.863	19.181	96.855	1.00	0.83	C
ATOM	880	CD2	LEU	A	112	24.033	18.069	96.417	1.00	0.83	C
ATOM	881	N	PRO	A	113	26.690	19.331	99.626	1.00	0.76	N
ATOM	882	CA	PRO	A	113	27.688	20.253	100.149	1.00	0.76	C
ATOM	883	C	PRO	A	113	27.161	21.468	100.861	1.00	0.76	C
ATOM	884	O	PRO	A	113	26.218	22.124	100.399	1.00	0.76	O
ATOM	885	CB	PRO	A	113	28.544	20.636	98.935	1.00	0.76	C
ATOM	886	CG	PRO	A	113	28.504	19.400	98.033	1.00	0.76	C
ATOM	887	CD	PRO	A	113	27.175	18.719	98.378	1.00	0.76	C
ATOM	888	N	LYS	A	114	27.783	21.800	101.998	1.00	0.58	N
ATOM	889	CA	LYS	A	114	27.357	22.923	102.771	1.00	0.58	C
ATOM	890	C	LYS	A	114	27.941	24.172	102.171	1.00	0.58	C
ATOM	891	O	LYS	A	114	29.153	24.372	102.124	1.00	0.58	O
ATOM	892	CB	LYS	A	114	27.715	22.739	104.262	1.00	0.58	C
ATOM	893	CG	LYS	A	114	26.554	23.083	105.212	1.00	0.58	C
ATOM	894	CD	LYS	A	114	25.367	22.103	105.035	1.00	0.58	C
ATOM	895	CE	LYS	A	114	24.205	22.249	106.011	1.00	0.58	C
ATOM	896	NZ	LYS	A	114	24.780	22.171	107.359	1.00	0.58	N
ATOM	897	N	LEU	A	115	27.062	25.059	101.673	1.00	0.67	N
ATOM	898	CA	LEU	A	115	27.450	26.395	101.321	1.00	0.67	C
ATOM	899	C	LEU	A	115	27.978	27.109	102.547	1.00	0.67	C
ATOM	900	O	LEU	A	115	27.282	27.279	103.546	1.00	0.67	O
ATOM	901	CB	LEU	A	115	26.261	27.210	100.806	1.00	0.67	C
ATOM	902	CG	LEU	A	115	25.591	26.694	99.526	1.00	0.67	C
ATOM	903	CD1	LEU	A	115	24.284	27.485	99.398	1.00	0.67	C
ATOM	904	CD2	LEU	A	115	26.498	26.844	98.293	1.00	0.67	C
ATOM	905	N	LYS	A	116	29.247	27.508	102.483	1.00	0.52	N
ATOM	906	CA	LYS	A	116	29.851	28.385	103.442	1.00	0.52	C
ATOM	907	C	LYS	A	116	29.985	29.722	102.711	1.00	0.52	C
ATOM	908	O	LYS	A	116	30.971	29.876	101.984	1.00	0.52	O
ATOM	909	CB	LYS	A	116	31.245	27.802	103.785	1.00	0.52	C
ATOM	910	CG	LYS	A	116	32.098	28.677	104.715	1.00	0.52	C
ATOM	911	CD	LYS	A	116	33.624	28.532	104.527	1.00	0.52	C
ATOM	912	CE	LYS	A	116	34.102	28.928	103.121	1.00	0.52	C
ATOM	913	NZ	LYS	A	116	35.582	29.013	103.044	1.00	0.52	N
ATOM	914	N	PRO	A	117	29.082	30.701	102.799	1.00	0.64	N
ATOM	915	CA	PRO	A	117	29.075	31.815	101.855	1.00	0.64	C
ATOM	916	C	PRO	A	117	30.173	32.799	102.242	1.00	0.64	C
ATOM	917	O	PRO	A	117	30.251	33.158	103.415	1.00	0.64	O
ATOM	918	CB	PRO	A	117	27.655	32.423	102.030	1.00	0.64	C
ATOM	919	CG	PRO	A	117	26.838	31.308	102.688	1.00	0.64	C
ATOM	920	CD	PRO	A	117	27.867	30.679	103.612	1.00	0.64	C
ATOM	921	N	ASP	A	118	31.009	33.287	101.301	1.00	0.79	N
ATOM	922	CA	ASP	A	118	32.091	34.206	101.618	1.00	0.79	C
ATOM	923	C	ASP	A	118	31.663	35.553	101.029	1.00	0.79	C

ATOM	924	O	ASP	A	118	31.330	35.603	99.843	1.00	0.79	O
ATOM	925	CB	ASP	A	118	33.472	33.658	101.127	1.00	0.79	C
ATOM	926	CG	ASP	A	118	34.425	34.761	100.692	1.00	0.79	C
ATOM	927	OD1	ASP	A	118	34.552	34.987	99.464	1.00	0.79	O
ATOM	928	OD2	ASP	A	118	34.957	35.427	101.611	1.00	0.79	O
ATOM	929	N	PRO	A	119	31.589	36.670	101.747	1.00	0.83	N
ATOM	930	CA	PRO	A	119	30.976	37.877	101.227	1.00	0.83	C
ATOM	931	C	PRO	A	119	31.734	38.540	100.097	1.00	0.83	C
ATOM	932	O	PRO	A	119	31.105	39.290	99.353	1.00	0.83	O
ATOM	933	CB	PRO	A	119	30.866	38.831	102.430	1.00	0.83	C
ATOM	934	CG	PRO	A	119	31.386	38.035	103.633	1.00	0.83	C
ATOM	935	CD	PRO	A	119	32.241	36.922	103.026	1.00	0.83	C
ATOM	936	N	GLU	A	120	33.069	38.367	99.966	1.00	0.77	N
ATOM	937	CA	GLU	A	120	33.784	39.026	98.896	1.00	0.77	C
ATOM	938	C	GLU	A	120	33.485	38.395	97.548	1.00	0.77	C
ATOM	939	O	GLU	A	120	32.996	39.071	96.640	1.00	0.77	O
ATOM	940	CB	GLU	A	120	35.299	39.040	99.164	1.00	0.77	C
ATOM	941	CG	GLU	A	120	36.128	39.681	98.024	1.00	0.77	C
ATOM	942	CD	GLU	A	120	37.616	39.780	98.366	1.00	0.77	C
ATOM	943	OE1	GLU	A	120	37.983	39.463	99.524	1.00	0.77	O
ATOM	944	OE2	GLU	A	120	38.378	40.239	97.478	1.00	0.77	O
ATOM	945	N	THR	A	121	33.665	37.060	97.417	1.00	0.82	N
ATOM	946	CA	THR	A	121	33.390	36.338	96.178	1.00	0.82	C
ATOM	947	C	THR	A	121	31.880	36.401	95.867	1.00	0.82	C
ATOM	948	O	THR	A	121	31.457	36.630	94.743	1.00	0.82	O
ATOM	949	CB	THR	A	121	34.085	34.953	96.135	1.00	0.82	C
ATOM	950	OG1	THR	A	121	34.556	34.577	94.846	1.00	0.82	O
ATOM	951	CG2	THR	A	121	33.211	33.794	96.608	1.00	0.82	C
ATOM	952	N	PHE	A	122	31.003	36.351	96.905	1.00	0.83	N
ATOM	953	CA	PHE	A	122	29.548	36.444	96.744	1.00	0.83	C
ATOM	954	C	PHE	A	122	29.041	37.815	96.267	1.00	0.83	C
ATOM	955	O	PHE	A	122	28.195	37.909	95.376	1.00	0.83	O
ATOM	956	CB	PHE	A	122	28.769	35.971	98.020	1.00	0.83	C
ATOM	957	CG	PHE	A	122	28.744	34.455	98.178	1.00	0.83	C
ATOM	958	CD1	PHE	A	122	29.903	33.682	97.989	1.00	0.83	C
ATOM	959	CD2	PHE	A	122	27.562	33.757	98.520	1.00	0.83	C
ATOM	960	CE1	PHE	A	122	29.884	32.294	98.029	1.00	0.83	C
ATOM	961	CE2	PHE	A	122	27.536	32.354	98.555	1.00	0.83	C
ATOM	962	CZ	PHE	A	122	28.697	31.628	98.297	1.00	0.83	C
ATOM	963	N	CYS	A	123	29.562	38.942	96.811	1.00	0.87	N
ATOM	964	CA	CYS	A	123	29.291	40.284	96.285	1.00	0.87	C
ATOM	965	C	CYS	A	123	29.856	40.490	94.889	1.00	0.87	C
ATOM	966	O	CYS	A	123	29.262	41.208	94.083	1.00	0.87	O
ATOM	967	CB	CYS	A	123	29.736	41.442	97.221	1.00	0.87	C
ATOM	968	SG	CYS	A	123	28.461	42.025	98.392	1.00	0.87	S
ATOM	969	N	THR	A	124	30.993	39.831	94.569	1.00	0.84	N
ATOM	970	CA	THR	A	124	31.570	39.790	93.219	1.00	0.84	C
ATOM	971	C	THR	A	124	30.586	39.242	92.185	1.00	0.84	C
ATOM	972	O	THR	A	124	30.332	39.883	91.162	1.00	0.84	O
ATOM	973	CB	THR	A	124	32.902	39.017	93.170	1.00	0.84	C
ATOM	974	OG1	THR	A	124	33.940	39.709	93.852	1.00	0.84	O
ATOM	975	CG2	THR	A	124	33.428	38.733	91.754	1.00	0.84	C
ATOM	976	N	GLU	A	125	29.930	38.097	92.468	1.00	0.80	N
ATOM	977	CA	GLU	A	125	29.023	37.420	91.560	1.00	0.80	C
ATOM	978	C	GLU	A	125	27.691	38.122	91.370	1.00	0.80	C
ATOM	979	O	GLU	A	125	27.088	38.054	90.296	1.00	0.80	O
ATOM	980	CB	GLU	A	125	28.750	35.992	92.081	1.00	0.80	C
ATOM	981	CG	GLU	A	125	29.999	35.070	92.077	1.00	0.80	C
ATOM	982	CD	GLU	A	125	29.908	33.896	93.059	1.00	0.80	C
ATOM	983	OE1	GLU	A	125	30.987	33.424	93.507	1.00	0.80	O
ATOM	984	OE2	GLU	A	125	28.771	33.436	93.339	1.00	0.80	O
ATOM	985	N	PHE	A	126	27.183	38.837	92.404	1.00	0.86	N
ATOM	986	CA	PHE	A	126	25.965	39.627	92.264	1.00	0.86	C
ATOM	987	C	PHE	A	126	26.183	40.770	91.256	1.00	0.86	C
ATOM	988	O	PHE	A	126	25.436	40.903	90.294	1.00	0.86	O
ATOM	989	CB	PHE	A	126	25.347	39.981	93.666	1.00	0.86	C
ATOM	990	CG	PHE	A	126	24.513	41.237	93.736	1.00	0.86	C
ATOM	991	CD1	PHE	A	126	23.140	41.263	93.440	1.00	0.86	C
ATOM	992	CD2	PHE	A	126	25.133	42.438	94.097	1.00	0.86	C
ATOM	993	CE1	PHE	A	126	22.431	42.474	93.465	1.00	0.86	C
ATOM	994	CE2	PHE	A	126	24.416	43.635	94.165	1.00	0.86	C
ATOM	995	CZ	PHE	A	126	23.061	43.662	93.836	1.00	0.86	C
ATOM	996	N	LYS	A	127	27.287	41.542	91.377	1.00	0.81	N
ATOM	997	CA	LYS	A	127	27.597	42.638	90.469	1.00	0.81	C
ATOM	998	C	LYS	A	127	27.876	42.231	89.026	1.00	0.81	C
ATOM	999	O	LYS	A	127	27.581	42.974	88.091	1.00	0.81	O

ATOM	1000	CB	LYS	A	127	28.762	43.521	90.992	1.00	0.81	C
ATOM	1001	CG	LYS	A	127	28.379	45.004	91.182	1.00	0.81	C
ATOM	1002	CD	LYS	A	127	28.205	45.764	89.849	1.00	0.81	C
ATOM	1003	CE	LYS	A	127	27.748	47.221	89.961	1.00	0.81	C
ATOM	1004	NZ	LYS	A	127	28.696	47.956	90.817	1.00	0.81	N
ATOM	1005	N	GLU	A	128	28.505	41.054	88.836	1.00	0.80	N
ATOM	1006	CA	GLU	A	128	28.737	40.451	87.536	1.00	0.80	C
ATOM	1007	C	GLU	A	128	27.470	40.110	86.776	1.00	0.80	C
ATOM	1008	O	GLU	A	128	27.375	40.374	85.577	1.00	0.80	O
ATOM	1009	CB	GLU	A	128	29.570	39.160	87.680	1.00	0.80	C
ATOM	1010	CG	GLU	A	128	29.900	38.466	86.332	1.00	0.80	C
ATOM	1011	CD	GLU	A	128	30.598	37.112	86.482	1.00	0.80	C
ATOM	1012	OE1	GLU	A	128	30.730	36.425	85.433	1.00	0.80	O
ATOM	1013	OE2	GLU	A	128	30.947	36.720	87.622	1.00	0.80	O
ATOM	1014	N	ASN	A	129	26.450	39.532	87.446	1.00	0.83	N
ATOM	1015	CA	ASN	A	129	25.192	39.275	86.785	1.00	0.83	C
ATOM	1016	C	ASN	A	129	24.121	38.974	87.824	1.00	0.83	C
ATOM	1017	O	ASN	A	129	23.949	37.848	88.300	1.00	0.83	O
ATOM	1018	CB	ASN	A	129	25.312	38.115	85.749	1.00	0.83	C
ATOM	1019	CG	ASN	A	129	24.034	37.828	84.963	1.00	0.83	C
ATOM	1020	OD1	ASN	A	129	22.904	38.148	85.337	1.00	0.83	O
ATOM	1021	ND2	ASN	A	129	24.219	37.128	83.816	1.00	0.83	N
ATOM	1022	N	GLU	A	130	23.308	39.996	88.129	1.00	0.80	N
ATOM	1023	CA	GLU	A	130	22.219	39.941	89.079	1.00	0.80	C
ATOM	1024	C	GLU	A	130	21.102	38.924	88.841	1.00	0.80	C
ATOM	1025	O	GLU	A	130	20.520	38.411	89.797	1.00	0.80	O
ATOM	1026	CB	GLU	A	130	21.619	41.344	89.194	1.00	0.80	C
ATOM	1027	CG	GLU	A	130	22.588	42.345	89.864	1.00	0.80	C
ATOM	1028	CD	GLU	A	130	22.048	43.774	89.756	1.00	0.80	C
ATOM	1029	OE1	GLU	A	130	22.863	44.727	89.724	1.00	0.80	O
ATOM	1030	OE2	GLU	A	130	20.784	43.899	89.694	1.00	0.80	O
ATOM	1031	N	GLN	A	131	20.741	38.628	87.573	1.00	0.79	N
ATOM	1032	CA	GLN	A	131	19.682	37.685	87.238	1.00	0.79	C
ATOM	1033	C	GLN	A	131	20.146	36.241	87.224	1.00	0.79	C
ATOM	1034	O	GLN	A	131	19.358	35.320	87.417	1.00	0.79	O
ATOM	1035	CB	GLN	A	131	19.037	38.028	85.871	1.00	0.79	C
ATOM	1036	CG	GLN	A	131	18.373	39.419	85.806	1.00	0.79	C
ATOM	1037	CD	GLN	A	131	17.339	39.550	86.915	1.00	0.79	C
ATOM	1038	OE1	GLN	A	131	16.530	38.670	87.190	1.00	0.79	O
ATOM	1039	NE2	GLN	A	131	17.344	40.719	87.604	1.00	0.79	N
ATOM	1040	N	LYS	A	132	21.461	35.992	87.055	1.00	0.79	N
ATOM	1041	CA	LYS	A	132	22.010	34.662	87.244	1.00	0.79	C
ATOM	1042	C	LYS	A	132	22.274	34.331	88.708	1.00	0.79	C
ATOM	1043	O	LYS	A	132	22.044	33.208	89.157	1.00	0.79	O
ATOM	1044	CB	LYS	A	132	23.257	34.441	86.365	1.00	0.79	C
ATOM	1045	CG	LYS	A	132	24.256	33.399	86.896	1.00	0.79	C
ATOM	1046	CD	LYS	A	132	25.264	32.952	85.832	1.00	0.79	C
ATOM	1047	CE	LYS	A	132	26.711	32.974	86.332	1.00	0.79	C
ATOM	1048	NZ	LYS	A	132	27.611	32.519	85.252	1.00	0.79	N
ATOM	1049	N	PHE	A	133	22.727	35.318	89.506	1.00	0.85	N
ATOM	1050	CA	PHE	A	133	22.888	35.220	90.950	1.00	0.85	C
ATOM	1051	C	PHE	A	133	21.564	34.846	91.621	1.00	0.85	C
ATOM	1052	O	PHE	A	133	21.496	34.021	92.534	1.00	0.85	O
ATOM	1053	CB	PHE	A	133	23.421	36.592	91.431	1.00	0.85	C
ATOM	1054	CG	PHE	A	133	23.650	36.673	92.910	1.00	0.85	C
ATOM	1055	CD1	PHE	A	133	24.912	36.410	93.455	1.00	0.85	C
ATOM	1056	CD2	PHE	A	133	22.605	37.056	93.766	1.00	0.85	C
ATOM	1057	CE1	PHE	A	133	25.124	36.509	94.833	1.00	0.85	C
ATOM	1058	CE2	PHE	A	133	22.822	37.194	95.140	1.00	0.85	C
ATOM	1059	CZ	PHE	A	133	24.080	36.902	95.674	1.00	0.85	C
ATOM	1060	N	TRP	A	134	20.471	35.437	91.102	1.00	0.82	N
ATOM	1061	CA	TRP	A	134	19.100	35.102	91.418	1.00	0.82	C
ATOM	1062	C	TRP	A	134	18.706	33.656	91.103	1.00	0.82	C
ATOM	1063	O	TRP	A	134	18.161	32.944	91.948	1.00	0.82	O
ATOM	1064	CB	TRP	A	134	18.228	36.089	90.611	1.00	0.82	C
ATOM	1065	CG	TRP	A	134	16.766	36.148	90.988	1.00	0.82	C
ATOM	1066	CD1	TRP	A	134	16.173	36.787	92.039	1.00	0.82	C
ATOM	1067	CD2	TRP	A	134	15.713	35.495	90.265	1.00	0.82	C
ATOM	1068	NE1	TRP	A	134	14.818	36.552	92.035	1.00	0.82	N
ATOM	1069	CE2	TRP	A	134	14.517	35.756	90.960	1.00	0.82	C
ATOM	1070	CE3	TRP	A	134	15.719	34.717	89.113	1.00	0.82	C
ATOM	1071	CZ2	TRP	A	134	13.311	35.232	90.523	1.00	0.82	C
ATOM	1072	CZ3	TRP	A	134	14.492	34.226	88.649	1.00	0.82	C
ATOM	1073	CH2	TRP	A	134	13.303	34.483	89.341	1.00	0.82	C
ATOM	1074	N	GLY	A	135	19.034	33.157	89.889	1.00	0.91	N
ATOM	1075	CA	GLY	A	135	18.729	31.787	89.477	1.00	0.91	C

ATOM	1076	C	GLY	A	135	19.550	30.731	90.184	1.00	0.91	C
ATOM	1077	O	GLY	A	135	19.079	29.638	90.483	1.00	0.91	O
ATOM	1078	N	LYS	A	136	20.812	31.040	90.525	1.00	0.83	N
ATOM	1079	CA	LYS	A	136	21.648	30.152	91.316	1.00	0.83	C
ATOM	1080	C	LYS	A	136	21.071	29.921	92.701	1.00	0.83	C
ATOM	1081	O	LYS	A	136	20.986	28.780	93.147	1.00	0.83	O
ATOM	1082	CB	LYS	A	136	23.120	30.628	91.396	1.00	0.83	C
ATOM	1083	CG	LYS	A	136	24.114	29.478	91.641	1.00	0.83	C
ATOM	1084	CD	LYS	A	136	25.096	29.292	90.476	1.00	0.83	C
ATOM	1085	CE	LYS	A	136	25.818	27.947	90.540	1.00	0.83	C
ATOM	1086	NZ	LYS	A	136	26.904	27.936	89.538	1.00	0.83	N
ATOM	1087	N	TYR	A	137	20.573	30.994	93.365	1.00	0.83	N
ATOM	1088	CA	TYR	A	137	19.894	30.910	94.651	1.00	0.83	C
ATOM	1089	C	TYR	A	137	18.651	29.993	94.596	1.00	0.83	C
ATOM	1090	O	TYR	A	137	18.491	29.093	95.425	1.00	0.83	O
ATOM	1091	CB	TYR	A	137	19.566	32.343	95.179	1.00	0.83	C
ATOM	1092	CG	TYR	A	137	18.843	32.296	96.497	1.00	0.83	C
ATOM	1093	CD1	TYR	A	137	19.518	32.210	97.723	1.00	0.83	C
ATOM	1094	CD2	TYR	A	137	17.446	32.237	96.497	1.00	0.83	C
ATOM	1095	CE1	TYR	A	137	18.804	32.098	98.925	1.00	0.83	C
ATOM	1096	CE2	TYR	A	137	16.733	32.102	97.693	1.00	0.83	C
ATOM	1097	CZ	TYR	A	137	17.410	32.060	98.913	1.00	0.83	C
ATOM	1098	OH	TYR	A	137	16.706	31.939	100.127	1.00	0.83	O
ATOM	1099	N	LEU	A	138	17.776	30.152	93.576	1.00	0.91	N
ATOM	1100	CA	LEU	A	138	16.571	29.352	93.403	1.00	0.91	C
ATOM	1101	C	LEU	A	138	16.849	27.869	93.215	1.00	0.91	C
ATOM	1102	O	LEU	A	138	16.180	27.011	93.789	1.00	0.91	O
ATOM	1103	CB	LEU	A	138	15.803	29.781	92.129	1.00	0.91	C
ATOM	1104	CG	LEU	A	138	15.061	31.128	92.118	1.00	0.91	C
ATOM	1105	CD1	LEU	A	138	14.414	31.341	90.737	1.00	0.91	C
ATOM	1106	CD2	LEU	A	138	13.976	31.174	93.197	1.00	0.91	C
ATOM	1107	N	TYR	A	139	17.867	27.555	92.386	1.00	0.89	N
ATOM	1108	CA	TYR	A	139	18.400	26.220	92.173	1.00	0.89	C
ATOM	1109	C	TYR	A	139	18.898	25.638	93.502	1.00	0.89	C
ATOM	1110	O	TYR	A	139	18.478	24.551	93.903	1.00	0.89	O
ATOM	1111	CB	TYR	A	139	19.472	26.259	91.019	1.00	0.89	C
ATOM	1112	CG	TYR	A	139	20.512	25.156	90.978	1.00	0.89	C
ATOM	1113	CD1	TYR	A	139	20.378	24.000	90.186	1.00	0.89	C
ATOM	1114	CD2	TYR	A	139	21.706	25.331	91.690	1.00	0.89	C
ATOM	1115	CE1	TYR	A	139	21.402	23.034	90.142	1.00	0.89	C
ATOM	1116	CE2	TYR	A	139	22.729	24.384	91.624	1.00	0.89	C
ATOM	1117	CZ	TYR	A	139	22.598	23.250	90.836	1.00	0.89	C
ATOM	1118	OH	TYR	A	139	23.711	22.388	90.766	1.00	0.89	O
ATOM	1119	N	GLU	A	140	19.723	26.406	94.246	1.00	0.85	N
ATOM	1120	CA	GLU	A	140	20.438	25.969	95.432	1.00	0.85	C
ATOM	1121	C	GLU	A	140	19.571	25.582	96.632	1.00	0.85	C
ATOM	1122	O	GLU	A	140	19.819	24.563	97.290	1.00	0.85	O
ATOM	1123	CB	GLU	A	140	21.560	26.969	95.805	1.00	0.85	C
ATOM	1124	CG	GLU	A	140	22.934	26.276	96.112	1.00	0.85	C
ATOM	1125	CD	GLU	A	140	23.505	25.328	95.019	1.00	0.85	C
ATOM	1126	OE1	GLU	A	140	24.092	25.806	94.022	1.00	0.85	O
ATOM	1127	OE2	GLU	A	140	23.373	24.081	95.200	1.00	0.85	O
ATOM	1128	N	ILE	A	141	18.510	26.382	96.909	1.00	0.88	N
ATOM	1129	CA	ILE	A	141	17.417	26.058	97.838	1.00	0.88	C
ATOM	1130	C	ILE	A	141	16.538	24.929	97.324	1.00	0.88	C
ATOM	1131	O	ILE	A	141	16.307	23.941	98.019	1.00	0.88	O
ATOM	1132	CB	ILE	A	141	16.511	27.255	98.157	1.00	0.88	C
ATOM	1133	CG1	ILE	A	141	17.313	28.498	98.599	1.00	0.88	C
ATOM	1134	CG2	ILE	A	141	15.413	26.917	99.201	1.00	0.88	C
ATOM	1135	CD1	ILE	A	141	18.208	28.315	99.826	1.00	0.88	C
ATOM	1136	N	ALA	A	142	16.047	24.995	96.068	1.00	0.93	N
ATOM	1137	CA	ALA	A	142	15.109	24.019	95.565	1.00	0.93	C
ATOM	1138	C	ALA	A	142	15.639	22.590	95.448	1.00	0.93	C
ATOM	1139	O	ALA	A	142	14.920	21.628	95.701	1.00	0.93	O
ATOM	1140	CB	ALA	A	142	14.521	24.541	94.249	1.00	0.93	C
ATOM	1141	N	ARG	A	143	16.922	22.395	95.100	1.00	0.86	N
ATOM	1142	CA	ARG	A	143	17.500	21.067	95.020	1.00	0.86	C
ATOM	1143	C	ARG	A	143	17.813	20.426	96.372	1.00	0.86	C
ATOM	1144	O	ARG	A	143	17.942	19.206	96.475	1.00	0.86	O
ATOM	1145	CB	ARG	A	143	18.768	21.113	94.143	1.00	0.86	C
ATOM	1146	CG	ARG	A	143	19.968	21.827	94.792	1.00	0.86	C
ATOM	1147	CD	ARG	A	143	21.010	22.301	93.780	1.00	0.86	C
ATOM	1148	NE	ARG	A	143	21.791	21.110	93.336	1.00	0.86	N
ATOM	1149	CZ	ARG	A	143	23.089	20.859	93.549	1.00	0.86	C
ATOM	1150	NH1	ARG	A	143	23.922	21.683	94.172	1.00	0.86	N
ATOM	1151	NH2	ARG	A	143	23.584	19.718	93.063	1.00	0.86	N

ATOM	1152	N	ARG	A	144	17.915	21.236	97.455	1.00	0.83	N
ATOM	1153	CA	ARG	A	144	18.062	20.721	98.809	1.00	0.83	C
ATOM	1154	C	ARG	A	144	16.735	20.530	99.523	1.00	0.83	C
ATOM	1155	O	ARG	A	144	16.649	19.768	100.481	1.00	0.83	O
ATOM	1156	CB	ARG	A	144	18.932	21.630	99.711	1.00	0.83	C
ATOM	1157	CG	ARG	A	144	20.421	21.608	99.330	1.00	0.83	C
ATOM	1158	CD	ARG	A	144	21.253	22.709	99.996	1.00	0.83	C
ATOM	1159	NE	ARG	A	144	22.704	22.538	99.636	1.00	0.83	N
ATOM	1160	CZ	ARG	A	144	23.222	22.788	98.424	1.00	0.83	C
ATOM	1161	NH1	ARG	A	144	22.466	23.078	97.370	1.00	0.83	N
ATOM	1162	NH2	ARG	A	144	24.540	22.758	98.239	1.00	0.83	N
ATOM	1163	N	HIS	A	145	15.669	21.199	99.052	1.00	0.86	N
ATOM	1164	CA	HIS	A	145	14.352	21.119	99.632	1.00	0.86	C
ATOM	1165	C	HIS	A	145	13.366	20.868	98.479	1.00	0.86	C
ATOM	1166	O	HIS	A	145	12.850	21.828	97.897	1.00	0.86	O
ATOM	1167	CB	HIS	A	145	14.005	22.449	100.346	1.00	0.86	C
ATOM	1168	CG	HIS	A	145	15.024	22.889	101.363	1.00	0.86	C
ATOM	1169	ND1	HIS	A	145	14.796	22.581	102.687	1.00	0.86	N
ATOM	1170	CD2	HIS	A	145	16.204	23.558	101.236	1.00	0.86	C
ATOM	1171	CE1	HIS	A	145	15.836	23.054	103.338	1.00	0.86	C
ATOM	1172	NE2	HIS	A	145	16.723	23.658	102.510	1.00	0.86	N
ATOM	1173	N	PRO	A	146	13.017	19.638	98.087	1.00	0.92	N
ATOM	1174	CA	PRO	A	146	12.490	19.368	96.747	1.00	0.92	C
ATOM	1175	C	PRO	A	146	10.965	19.454	96.740	1.00	0.92	C
ATOM	1176	O	PRO	A	146	10.319	19.253	95.710	1.00	0.92	O
ATOM	1177	CB	PRO	A	146	13.026	17.969	96.408	1.00	0.92	C
ATOM	1178	CG	PRO	A	146	13.211	17.292	97.761	1.00	0.92	C
ATOM	1179	CD	PRO	A	146	13.583	18.439	98.692	1.00	0.92	C
ATOM	1180	N	TYR	A	147	10.374	19.789	97.897	1.00	0.88	N
ATOM	1181	CA	TYR	A	147	8.954	20.055	98.093	1.00	0.88	C
ATOM	1182	C	TYR	A	147	8.723	21.505	98.523	1.00	0.88	C
ATOM	1183	O	TYR	A	147	7.649	21.853	99.010	1.00	0.88	O
ATOM	1184	CB	TYR	A	147	8.279	19.122	99.140	1.00	0.88	C
ATOM	1185	CG	TYR	A	147	8.289	17.687	98.722	1.00	0.88	C
ATOM	1186	CD1	TYR	A	147	9.381	16.864	99.015	1.00	0.88	C
ATOM	1187	CD2	TYR	A	147	7.163	17.124	98.104	1.00	0.88	C
ATOM	1188	CE1	TYR	A	147	9.331	15.495	98.734	1.00	0.88	C
ATOM	1189	CE2	TYR	A	147	7.119	15.758	97.800	1.00	0.88	C
ATOM	1190	CZ	TYR	A	147	8.198	14.937	98.145	1.00	0.88	C
ATOM	1191	OH	TYR	A	147	8.143	13.545	97.973	1.00	0.88	O
ATOM	1192	N	PHE	A	148	9.734	22.391	98.350	1.00	0.87	N
ATOM	1193	CA	PHE	A	148	9.652	23.809	98.699	1.00	0.87	C
ATOM	1194	C	PHE	A	148	8.501	24.531	98.011	1.00	0.87	C
ATOM	1195	O	PHE	A	148	8.249	24.346	96.822	1.00	0.87	O
ATOM	1196	CB	PHE	A	148	10.975	24.567	98.365	1.00	0.87	C
ATOM	1197	CG	PHE	A	148	11.181	25.811	99.200	1.00	0.87	C
ATOM	1198	CD1	PHE	A	148	10.500	27.016	98.945	1.00	0.87	C
ATOM	1199	CD2	PHE	A	148	12.063	25.778	100.288	1.00	0.87	C
ATOM	1200	CE1	PHE	A	148	10.626	28.113	99.802	1.00	0.87	C
ATOM	1201	CE2	PHE	A	148	12.184	26.870	101.149	1.00	0.87	C
ATOM	1202	CZ	PHE	A	148	11.458	28.032	100.915	1.00	0.87	C
ATOM	1203	N	TYR	A	149	7.775	25.381	98.765	1.00	0.86	N
ATOM	1204	CA	TYR	A	149	6.673	26.173	98.247	1.00	0.86	C
ATOM	1205	C	TYR	A	149	7.095	27.201	97.196	1.00	0.86	C
ATOM	1206	O	TYR	A	149	7.580	28.285	97.511	1.00	0.86	O
ATOM	1207	CB	TYR	A	149	5.919	26.828	99.429	1.00	0.86	C
ATOM	1208	CG	TYR	A	149	4.563	27.395	99.102	1.00	0.86	C
ATOM	1209	CD1	TYR	A	149	3.772	27.019	98.004	1.00	0.86	C
ATOM	1210	CD2	TYR	A	149	4.057	28.355	99.979	1.00	0.86	C
ATOM	1211	CE1	TYR	A	149	2.519	27.615	97.789	1.00	0.86	C
ATOM	1212	CE2	TYR	A	149	2.818	28.963	99.764	1.00	0.86	C
ATOM	1213	CZ	TYR	A	149	2.037	28.574	98.678	1.00	0.86	C
ATOM	1214	OH	TYR	A	149	0.754	29.117	98.509	1.00	0.86	O
ATOM	1215	N	ALA	A	150	6.892	26.875	95.899	1.00	0.90	N
ATOM	1216	CA	ALA	A	150	7.552	27.551	94.793	1.00	0.90	C
ATOM	1217	C	ALA	A	150	7.309	29.061	94.614	1.00	0.90	C
ATOM	1218	O	ALA	A	150	8.290	29.793	94.443	1.00	0.90	O
ATOM	1219	CB	ALA	A	150	7.388	26.690	93.518	1.00	0.90	C
ATOM	1220	N	PRO	A	151	6.113	29.629	94.724	1.00	0.88	N
ATOM	1221	CA	PRO	A	151	5.925	31.077	94.704	1.00	0.88	C
ATOM	1222	C	PRO	A	151	6.613	31.839	95.830	1.00	0.88	C
ATOM	1223	O	PRO	A	151	6.962	32.999	95.629	1.00	0.88	O
ATOM	1224	CB	PRO	A	151	4.401	31.254	94.743	1.00	0.88	C
ATOM	1225	CG	PRO	A	151	3.846	29.948	94.170	1.00	0.88	C
ATOM	1226	CD	PRO	A	151	4.839	28.908	94.665	1.00	0.88	C
ATOM	1227	N	GLU	A	152	6.798	31.224	97.019	1.00	0.84	N

ATOM	1228	CA	GLU	A	152	7.524	31.797	98.139	1.00	0.84	C
ATOM	1229	C	GLU	A	152	9.014	31.726	97.902	1.00	0.84	C
ATOM	1230	O	GLU	A	152	9.791	32.551	98.377	1.00	0.84	O
ATOM	1231	CB	GLU	A	152	7.198	31.041	99.441	1.00	0.84	C
ATOM	1232	CG	GLU	A	152	5.748	31.307	99.920	1.00	0.84	C
ATOM	1233	CD	GLU	A	152	5.676	32.647	100.680	1.00	0.84	C
ATOM	1234	OE1	GLU	A	152	6.340	32.727	101.724	1.00	0.84	O
ATOM	1235	OE2	GLU	A	152	4.961	33.574	100.190	1.00	0.84	O
ATOM	1236	N	LEU	A	153	9.462	30.751	97.089	1.00	0.89	N
ATOM	1237	CA	LEU	A	153	10.852	30.691	96.693	1.00	0.89	C
ATOM	1238	C	LEU	A	153	11.277	31.882	95.866	1.00	0.89	C
ATOM	1239	O	LEU	A	153	12.316	32.473	96.154	1.00	0.89	O
ATOM	1240	CB	LEU	A	153	11.240	29.366	96.021	1.00	0.89	C
ATOM	1241	CG	LEU	A	153	12.737	28.957	96.084	1.00	0.89	C
ATOM	1242	CD1	LEU	A	153	13.584	29.527	97.239	1.00	0.89	C
ATOM	1243	CD2	LEU	A	153	12.826	27.427	96.128	1.00	0.89	C
ATOM	1244	N	LEU	A	154	10.443	32.323	94.888	1.00	0.87	N
ATOM	1245	CA	LEU	A	154	10.667	33.560	94.157	1.00	0.87	C
ATOM	1246	C	LEU	A	154	10.715	34.736	95.119	1.00	0.87	C
ATOM	1247	O	LEU	A	154	11.681	35.487	95.121	1.00	0.87	O
ATOM	1248	CB	LEU	A	154	9.586	33.786	93.074	1.00	0.87	C
ATOM	1249	CG	LEU	A	154	9.380	32.610	92.103	1.00	0.87	C
ATOM	1250	CD1	LEU	A	154	8.030	32.798	91.401	1.00	0.87	C
ATOM	1251	CD2	LEU	A	154	10.519	32.450	91.083	1.00	0.87	C
ATOM	1252	N	TYR	A	155	9.731	34.830	96.054	1.00	0.86	N
ATOM	1253	CA	TYR	A	155	9.702	35.852	97.093	1.00	0.86	C
ATOM	1254	C	TYR	A	155	11.036	35.968	97.848	1.00	0.86	C
ATOM	1255	O	TYR	A	155	11.679	37.019	97.827	1.00	0.86	O
ATOM	1256	CB	TYR	A	155	8.502	35.595	98.065	1.00	0.86	C
ATOM	1257	CG	TYR	A	155	8.556	36.436	99.316	1.00	0.86	C
ATOM	1258	CD1	TYR	A	155	8.313	37.813	99.270	1.00	0.86	C
ATOM	1259	CD2	TYR	A	155	8.969	35.863	100.530	1.00	0.86	C
ATOM	1260	CE1	TYR	A	155	8.487	38.600	100.415	1.00	0.86	C
ATOM	1261	CE2	TYR	A	155	9.197	36.662	101.659	1.00	0.86	C
ATOM	1262	CZ	TYR	A	155	8.953	38.036	101.602	1.00	0.86	C
ATOM	1263	OH	TYR	A	155	9.183	38.880	102.707	1.00	0.86	O
ATOM	1264	N	PHE	A	156	11.515	34.864	98.462	1.00	0.89	N
ATOM	1265	CA	PHE	A	156	12.775	34.838	99.184	1.00	0.89	C
ATOM	1266	C	PHE	A	156	13.997	35.175	98.335	1.00	0.89	C
ATOM	1267	O	PHE	A	156	14.885	35.896	98.780	1.00	0.89	O
ATOM	1268	CB	PHE	A	156	13.023	33.482	99.884	1.00	0.89	C
ATOM	1269	CG	PHE	A	156	12.097	33.268	101.042	1.00	0.89	C
ATOM	1270	CD1	PHE	A	156	12.180	34.105	102.162	1.00	0.89	C
ATOM	1271	CD2	PHE	A	156	11.196	32.193	101.071	1.00	0.89	C
ATOM	1272	CE1	PHE	A	156	11.435	33.841	103.313	1.00	0.89	C
ATOM	1273	CE2	PHE	A	156	10.374	31.984	102.183	1.00	0.89	C
ATOM	1274	CZ	PHE	A	156	10.519	32.786	103.319	1.00	0.89	C
ATOM	1275	N	ALA	A	157	14.068	34.675	97.089	1.00	0.92	N
ATOM	1276	CA	ALA	A	157	15.103	34.976	96.109	1.00	0.92	C
ATOM	1277	C	ALA	A	157	15.172	36.449	95.669	1.00	0.92	C
ATOM	1278	O	ALA	A	157	16.251	37.013	95.475	1.00	0.92	O
ATOM	1279	CB	ALA	A	157	14.937	34.028	94.910	1.00	0.92	C
ATOM	1280	N	HIS	A	158	14.012	37.130	95.538	1.00	0.81	N
ATOM	1281	CA	HIS	A	158	13.929	38.588	95.456	1.00	0.81	C
ATOM	1282	C	HIS	A	158	14.381	39.327	96.717	1.00	0.81	C
ATOM	1283	O	HIS	A	158	15.113	40.316	96.624	1.00	0.81	O
ATOM	1284	CB	HIS	A	158	12.546	39.110	95.009	1.00	0.81	C
ATOM	1285	CG	HIS	A	158	12.279	38.869	93.559	1.00	0.81	C
ATOM	1286	ND1	HIS	A	158	11.720	37.680	93.158	1.00	0.81	N
ATOM	1287	CD2	HIS	A	158	12.501	39.669	92.484	1.00	0.81	C
ATOM	1288	CE1	HIS	A	158	11.604	37.773	91.853	1.00	0.81	C
ATOM	1289	NE2	HIS	A	158	12.063	38.957	91.390	1.00	0.81	N
ATOM	1290	N	GLN	A	159	14.005	38.841	97.928	1.00	0.81	N
ATOM	1291	CA	GLN	A	159	14.482	39.364	99.211	1.00	0.81	C
ATOM	1292	C	GLN	A	159	15.995	39.276	99.324	1.00	0.81	C
ATOM	1293	O	GLN	A	159	16.659	40.179	99.827	1.00	0.81	O
ATOM	1294	CB	GLN	A	159	13.875	38.653	100.462	1.00	0.81	C
ATOM	1295	CG	GLN	A	159	12.341	38.712	100.633	1.00	0.81	C
ATOM	1296	CD	GLN	A	159	11.869	40.157	100.755	1.00	0.81	C
ATOM	1297	OE1	GLN	A	159	12.319	40.912	101.615	1.00	0.81	O
ATOM	1298	NE2	GLN	A	159	10.944	40.562	99.857	1.00	0.81	N
ATOM	1299	N	TYR	A	160	16.565	38.172	98.810	1.00	0.85	N
ATOM	1300	CA	TYR	A	160	17.976	37.874	98.794	1.00	0.85	C
ATOM	1301	C	TYR	A	160	18.818	38.851	97.976	1.00	0.85	C
ATOM	1302	O	TYR	A	160	19.767	39.448	98.479	1.00	0.85	O
ATOM	1303	CB	TYR	A	160	18.070	36.437	98.244	1.00	0.85	C

ATOM	1304	CG	TYR	A	160	19.422	35.846	98.381	1.00	0.85	C
ATOM	1305	CD1	TYR	A	160	19.886	35.468	99.643	1.00	0.85	C
ATOM	1306	CD2	TYR	A	160	20.227	35.636	97.258	1.00	0.85	C
ATOM	1307	CE1	TYR	A	160	21.152	34.893	99.786	1.00	0.85	C
ATOM	1308	CE2	TYR	A	160	21.491	35.053	97.397	1.00	0.85	C
ATOM	1309	CZ	TYR	A	160	21.956	34.686	98.663	1.00	0.85	C
ATOM	1310	OH	TYR	A	160	23.234	34.118	98.794	1.00	0.85	O
ATOM	1311	N	LYS	A	161	18.418	39.111	96.711	1.00	0.81	N
ATOM	1312	CA	LYS	A	161	18.990	40.128	95.845	1.00	0.81	C
ATOM	1313	C	LYS	A	161	18.822	41.508	96.451	1.00	0.81	C
ATOM	1314	O	LYS	A	161	19.704	42.356	96.345	1.00	0.81	O
ATOM	1315	CB	LYS	A	161	18.380	40.045	94.417	1.00	0.81	C
ATOM	1316	CG	LYS	A	161	18.731	41.281	93.568	1.00	0.81	C
ATOM	1317	CD	LYS	A	161	18.327	41.280	92.086	1.00	0.81	C
ATOM	1318	CE	LYS	A	161	18.686	42.623	91.429	1.00	0.81	C
ATOM	1319	NZ	LYS	A	161	18.327	42.635	89.996	1.00	0.81	N
ATOM	1320	N	GLY	A	162	17.678	41.734	97.127	1.00	0.88	N
ATOM	1321	CA	GLY	A	162	17.446	42.877	98.010	1.00	0.88	C
ATOM	1322	C	GLY	A	162	18.571	43.167	99.002	1.00	0.88	C
ATOM	1323	O	GLY	A	162	19.097	44.281	99.044	1.00	0.88	O
ATOM	1324	N	VAL	A	163	19.007	42.148	99.780	1.00	0.88	N
ATOM	1325	CA	VAL	A	163	20.112	42.250	100.739	1.00	0.88	C
ATOM	1326	C	VAL	A	163	21.427	42.639	100.077	1.00	0.88	C
ATOM	1327	O	VAL	A	163	22.169	43.489	100.558	1.00	0.88	O
ATOM	1328	CB	VAL	A	163	20.388	40.969	101.545	1.00	0.88	C
ATOM	1329	CG1	VAL	A	163	21.457	41.237	102.627	1.00	0.88	C
ATOM	1330	CG2	VAL	A	163	19.121	40.453	102.244	1.00	0.88	C
ATOM	1331	N	PHE	A	164	21.771	42.030	98.928	1.00	0.86	N
ATOM	1332	CA	PHE	A	164	22.980	42.381	98.205	1.00	0.86	C
ATOM	1333	C	PHE	A	164	22.939	43.784	97.605	1.00	0.86	C
ATOM	1334	O	PHE	A	164	23.935	44.512	97.631	1.00	0.86	O
ATOM	1335	CB	PHE	A	164	23.362	41.311	97.164	1.00	0.86	C
ATOM	1336	CG	PHE	A	164	23.860	40.072	97.867	1.00	0.86	C
ATOM	1337	CD1	PHE	A	164	25.212	39.944	98.209	1.00	0.86	C
ATOM	1338	CD2	PHE	A	164	22.986	39.048	98.253	1.00	0.86	C
ATOM	1339	CE1	PHE	A	164	25.674	38.836	98.926	1.00	0.86	C
ATOM	1340	CE2	PHE	A	164	23.427	37.962	99.017	1.00	0.86	C
ATOM	1341	CZ	PHE	A	164	24.780	37.845	99.338	1.00	0.86	C
ATOM	1342	N	ALA	A	165	21.761	44.213	97.111	1.00	0.88	N
ATOM	1343	CA	ALA	A	165	21.498	45.552	96.640	1.00	0.88	C
ATOM	1344	C	ALA	A	165	21.686	46.618	97.715	1.00	0.88	C
ATOM	1345	O	ALA	A	165	22.305	47.644	97.450	1.00	0.88	O
ATOM	1346	CB	ALA	A	165	20.077	45.598	96.037	1.00	0.88	C
ATOM	1347	N	GLU	A	166	21.194	46.395	98.956	1.00	0.79	N
ATOM	1348	CA	GLU	A	166	21.496	47.286	100.071	1.00	0.79	C
ATOM	1349	C	GLU	A	166	22.946	47.178	100.617	1.00	0.79	C
ATOM	1350	O	GLU	A	166	23.627	48.189	100.812	1.00	0.79	O
ATOM	1351	CB	GLU	A	166	20.404	47.211	101.191	1.00	0.79	C
ATOM	1352	CG	GLU	A	166	20.492	45.979	102.135	1.00	0.79	C
ATOM	1353	CD	GLU	A	166	19.376	45.782	103.179	1.00	0.79	C
ATOM	1354	OE1	GLU	A	166	18.741	44.695	103.182	1.00	0.79	O
ATOM	1355	OE2	GLU	A	166	19.229	46.653	104.079	1.00	0.79	O
ATOM	1356	N	CYS	A	167	23.503	45.951	100.823	1.00	0.88	N
ATOM	1357	CA	CYS	A	167	24.753	45.727	101.551	1.00	0.88	C
ATOM	1358	C	CYS	A	167	26.064	45.662	100.752	1.00	0.88	C
ATOM	1359	O	CYS	A	167	27.137	45.808	101.343	1.00	0.88	O
ATOM	1360	CB	CYS	A	167	24.723	44.390	102.346	1.00	0.88	C
ATOM	1361	SG	CYS	A	167	23.713	44.384	103.849	1.00	0.88	S
ATOM	1362	N	CYS	A	168	26.075	45.501	99.403	1.00	0.88	N
ATOM	1363	CA	CYS	A	168	27.342	45.476	98.659	1.00	0.88	C
ATOM	1364	C	CYS	A	168	27.845	46.888	98.371	1.00	0.88	C
ATOM	1365	O	CYS	A	168	28.910	47.055	97.777	1.00	0.88	O
ATOM	1366	CB	CYS	A	168	27.335	44.732	97.278	1.00	0.88	C
ATOM	1367	SG	CYS	A	168	27.023	42.935	97.262	1.00	0.88	S
ATOM	1368	N	GLN	A	169	27.112	47.935	98.816	1.00	0.81	N
ATOM	1369	CA	GLN	A	169	27.554	49.306	98.705	1.00	0.81	C
ATOM	1370	C	GLN	A	169	28.158	49.829	100.014	1.00	0.81	C
ATOM	1371	O	GLN	A	169	28.688	50.937	100.079	1.00	0.81	O
ATOM	1372	CB	GLN	A	169	26.365	50.188	98.257	1.00	0.81	C
ATOM	1373	CG	GLN	A	169	26.815	51.518	97.613	1.00	0.81	C
ATOM	1374	CD	GLN	A	169	25.626	52.390	97.218	1.00	0.81	C
ATOM	1375	OE1	GLN	A	169	24.455	52.039	97.342	1.00	0.81	O
ATOM	1376	NE2	GLN	A	169	25.941	53.602	96.705	1.00	0.81	N
ATOM	1377	N	ALA	A	170	28.124	49.028	101.101	1.00	0.86	N
ATOM	1378	CA	ALA	A	170	28.661	49.420	102.390	1.00	0.86	C
ATOM	1379	C	ALA	A	170	30.156	49.128	102.543	1.00	0.86	C

ATOM	1380	O	ALA	A	170	30.729	48.298	101.838	1.00	0.86	O
ATOM	1381	CB	ALA	A	170	27.851	48.757	103.523	1.00	0.86	C
ATOM	1382	N	ALA	A	171	30.838	49.812	103.499	1.00	0.81	N
ATOM	1383	CA	ALA	A	171	32.276	49.686	103.682	1.00	0.81	C
ATOM	1384	C	ALA	A	171	32.702	48.387	104.370	1.00	0.81	C
ATOM	1385	O	ALA	A	171	33.823	47.913	104.198	1.00	0.81	O
ATOM	1386	CB	ALA	A	171	32.812	50.936	104.415	1.00	0.81	C
ATOM	1387	N	ASP	A	172	31.768	47.737	105.092	1.00	0.85	N
ATOM	1388	CA	ASP	A	172	31.966	46.419	105.631	1.00	0.85	C
ATOM	1389	C	ASP	A	172	30.804	45.590	105.086	1.00	0.85	C
ATOM	1390	O	ASP	A	172	29.687	45.562	105.609	1.00	0.85	O
ATOM	1391	CB	ASP	A	172	32.059	46.423	107.178	1.00	0.85	C
ATOM	1392	CG	ASP	A	172	32.636	45.097	107.665	1.00	0.85	C
ATOM	1393	OD1	ASP	A	172	32.242	44.043	107.090	1.00	0.85	O
ATOM	1394	OD2	ASP	A	172	33.458	45.118	108.609	1.00	0.85	O
ATOM	1395	N	LYS	A	173	31.056	44.907	103.951	1.00	0.82	N
ATOM	1396	CA	LYS	A	173	30.109	44.000	103.335	1.00	0.82	C
ATOM	1397	C	LYS	A	173	29.753	42.804	104.214	1.00	0.82	C
ATOM	1398	O	LYS	A	173	28.610	42.365	104.282	1.00	0.82	O
ATOM	1399	CB	LYS	A	173	30.661	43.443	102.000	1.00	0.82	C
ATOM	1400	CG	LYS	A	173	31.099	44.498	100.978	1.00	0.82	C
ATOM	1401	CD	LYS	A	173	31.501	43.847	99.636	1.00	0.82	C
ATOM	1402	CE	LYS	A	173	32.825	43.068	99.545	1.00	0.82	C
ATOM	1403	NZ	LYS	A	173	33.972	43.981	99.736	1.00	0.82	N
ATOM	1404	N	GLY	A	174	30.769	42.239	104.891	1.00	0.87	N
ATOM	1405	CA	GLY	A	174	30.671	41.133	105.841	1.00	0.87	C
ATOM	1406	C	GLY	A	174	29.756	41.377	107.020	1.00	0.87	C
ATOM	1407	O	GLY	A	174	28.859	40.579	107.296	1.00	0.87	O
ATOM	1408	N	ALA	A	175	29.969	42.504	107.728	1.00	0.89	N
ATOM	1409	CA	ALA	A	175	29.170	42.962	108.852	1.00	0.89	C
ATOM	1410	C	ALA	A	175	27.709	43.281	108.496	1.00	0.89	C
ATOM	1411	O	ALA	A	175	26.800	43.097	109.308	1.00	0.89	O
ATOM	1412	CB	ALA	A	175	29.876	44.158	109.531	1.00	0.89	C
ATOM	1413	N	CYS	A	176	27.448	43.779	107.267	1.00	0.91	N
ATOM	1414	CA	CYS	A	176	26.110	44.011	106.743	1.00	0.91	C
ATOM	1415	C	CYS	A	176	25.363	42.734	106.333	1.00	0.91	C
ATOM	1416	O	CYS	A	176	24.184	42.552	106.648	1.00	0.91	O
ATOM	1417	CB	CYS	A	176	26.212	45.018	105.566	1.00	0.91	C
ATOM	1418	SG	CYS	A	176	24.641	45.775	105.007	1.00	0.91	S
ATOM	1419	N	LEU	A	177	26.047	41.813	105.617	1.00	0.87	N
ATOM	1420	CA	LEU	A	177	25.428	40.701	104.906	1.00	0.87	C
ATOM	1421	C	LEU	A	177	25.059	39.471	105.692	1.00	0.87	C
ATOM	1422	O	LEU	A	177	23.926	38.988	105.624	1.00	0.87	O
ATOM	1423	CB	LEU	A	177	26.406	40.179	103.834	1.00	0.87	C
ATOM	1424	CG	LEU	A	177	26.388	40.997	102.546	1.00	0.87	C
ATOM	1425	CD1	LEU	A	177	27.559	40.586	101.660	1.00	0.87	C
ATOM	1426	CD2	LEU	A	177	25.064	40.764	101.822	1.00	0.87	C
ATOM	1427	N	ILE	A	178	26.035	38.928	106.444	1.00	0.83	N
ATOM	1428	CA	ILE	A	178	25.871	37.722	107.247	1.00	0.83	C
ATOM	1429	C	ILE	A	178	24.708	37.779	108.228	1.00	0.83	C
ATOM	1430	O	ILE	A	178	23.881	36.864	108.136	1.00	0.83	O
ATOM	1431	CB	ILE	A	178	27.181	37.286	107.901	1.00	0.83	C
ATOM	1432	CG1	ILE	A	178	28.225	36.958	106.803	1.00	0.83	C
ATOM	1433	CG2	ILE	A	178	26.951	36.061	108.822	1.00	0.83	C
ATOM	1434	CD1	ILE	A	178	29.642	36.756	107.351	1.00	0.83	C
ATOM	1435	N	PRO	A	179	24.478	38.772	109.109	1.00	0.86	N
ATOM	1436	CA	PRO	A	179	23.279	38.809	109.935	1.00	0.86	C
ATOM	1437	C	PRO	A	179	21.978	38.717	109.144	1.00	0.86	C
ATOM	1438	O	PRO	A	179	21.083	37.993	109.565	1.00	0.86	O
ATOM	1439	CB	PRO	A	179	23.375	40.112	110.761	1.00	0.86	C
ATOM	1440	CG	PRO	A	179	24.746	40.722	110.445	1.00	0.86	C
ATOM	1441	CD	PRO	A	179	25.134	40.085	109.115	1.00	0.86	C
ATOM	1442	N	LYS	A	180	21.842	39.430	108.004	1.00	0.81	N
ATOM	1443	CA	LYS	A	180	20.641	39.387	107.188	1.00	0.81	C
ATOM	1444	C	LYS	A	180	20.390	38.061	106.477	1.00	0.81	C
ATOM	1445	O	LYS	A	180	19.260	37.570	106.474	1.00	0.81	O
ATOM	1446	CB	LYS	A	180	20.566	40.590	106.220	1.00	0.81	C
ATOM	1447	CG	LYS	A	180	20.337	41.931	106.946	1.00	0.81	C
ATOM	1448	CD	LYS	A	180	20.182	43.090	105.942	1.00	0.81	C
ATOM	1449	CE	LYS	A	180	20.346	44.508	106.494	1.00	0.81	C
ATOM	1450	NZ	LYS	A	180	19.238	44.806	107.416	1.00	0.81	N
ATOM	1451	N	ILE	A	181	21.421	37.422	105.878	1.00	0.83	N
ATOM	1452	CA	ILE	A	181	21.267	36.088	105.313	1.00	0.83	C
ATOM	1453	C	ILE	A	181	21.043	34.995	106.349	1.00	0.83	C
ATOM	1454	O	ILE	A	181	20.300	34.059	106.084	1.00	0.83	O
ATOM	1455	CB	ILE	A	181	22.287	35.657	104.259	1.00	0.83	C

```
ATOM   1456  CG1 ILE A 181      23.666  35.308 104.869  1.00  0.83           C
ATOM   1457  CG2 ILE A 181      22.251  36.665 103.080  1.00  0.83           C
ATOM   1458  CD1 ILE A 181      24.876  35.602 103.982  1.00  0.83           C
ATOM   1459  N   GLU A 182      21.635  35.089 107.562  1.00  0.79           N
ATOM   1460  CA  GLU A 182      21.305  34.193 108.661  1.00  0.79           C
ATOM   1461  C   GLU A 182      19.880  34.317 109.179  1.00  0.79           C
ATOM   1462  O   GLU A 182      19.185  33.307 109.320  1.00  0.79           O
ATOM   1463  CB  GLU A 182      22.321  34.308 109.820  1.00  0.79           C
ATOM   1464  CG  GLU A 182      23.689  33.647 109.506  1.00  0.79           C
ATOM   1465  CD  GLU A 182      23.582  32.255 108.873  1.00  0.79           C
ATOM   1466  OE1 GLU A 182      22.918  31.350 109.456  1.00  0.79           O
ATOM   1467  OE2 GLU A 182      24.156  32.086 107.767  1.00  0.79           O
ATOM   1468  N   THR A 183      19.357  35.550 109.393  1.00  0.81           N
ATOM   1469  CA  THR A 183      17.938  35.752 109.737  1.00  0.81           C
ATOM   1470  C   THR A 183      17.025  35.167 108.654  1.00  0.81           C
ATOM   1471  O   THR A 183      16.071  34.435 108.926  1.00  0.81           O
ATOM   1472  CB  THR A 183      17.555  37.219 109.989  1.00  0.81           C
ATOM   1473  OG1 THR A 183      18.428  37.839 110.923  1.00  0.81           O
ATOM   1474  CG2 THR A 183      16.157  37.325 110.614  1.00  0.81           C
ATOM   1475  N   LEU A 184      17.369  35.417 107.375  1.00  0.85           N
ATOM   1476  CA  LEU A 184      16.666  34.915 106.213  1.00  0.85           C
ATOM   1477  C   LEU A 184      16.674  33.399 106.022  1.00  0.85           C
ATOM   1478  O   LEU A 184      15.676  32.786 105.653  1.00  0.85           O
ATOM   1479  CB  LEU A 184      17.283  35.555 104.957  1.00  0.85           C
ATOM   1480  CG  LEU A 184      16.558  35.226 103.644  1.00  0.85           C
ATOM   1481  CD1 LEU A 184      15.106  35.708 103.682  1.00  0.85           C
ATOM   1482  CD2 LEU A 184      17.275  35.841 102.442  1.00  0.85           C
ATOM   1483  N   ARG A 185      17.830  32.749 106.252  1.00  0.77           N
ATOM   1484  CA  ARG A 185      18.001  31.309 106.164  1.00  0.77           C
ATOM   1485  C   ARG A 185      17.111  30.536 107.148  1.00  0.77           C
ATOM   1486  O   ARG A 185      16.487  29.541 106.773  1.00  0.77           O
ATOM   1487  CB  ARG A 185      19.502  30.986 106.382  1.00  0.77           C
ATOM   1488  CG  ARG A 185      19.920  29.508 106.471  1.00  0.77           C
ATOM   1489  CD  ARG A 185      21.028  29.324 107.511  1.00  0.77           C
ATOM   1490  NE  ARG A 185      21.317  27.854 107.529  1.00  0.77           N
ATOM   1491  CZ  ARG A 185      21.635  27.181 108.639  1.00  0.77           C
ATOM   1492  NH1 ARG A 185      21.642  27.776 109.823  1.00  0.77           N
ATOM   1493  NH2 ARG A 185      21.938  25.883 108.538  1.00  0.77           N
ATOM   1494  N   GLU A 186      16.990  30.983 108.418  1.00  0.79           N
ATOM   1495  CA  GLU A 186      16.091  30.394 109.410  1.00  0.79           C
ATOM   1496  C   GLU A 186      14.610  30.460 109.000  1.00  0.79           C
ATOM   1497  O   GLU A 186      13.859  29.488 109.145  1.00  0.79           O
ATOM   1498  CB  GLU A 186      16.362  30.991 110.819  1.00  0.79           C
ATOM   1499  CG  GLU A 186      17.662  30.426 111.485  1.00  0.79           C
ATOM   1500  CD  GLU A 186      17.468  29.320 112.549  1.00  0.79           C
ATOM   1501  OE1 GLU A 186      16.369  28.709 112.607  1.00  0.79           O
ATOM   1502  OE2 GLU A 186      18.456  29.009 113.274  1.00  0.79           O
ATOM   1503  N   GLU A 187      14.161  31.591 108.405  1.00  0.81           N
ATOM   1504  CA  GLU A 187      12.828  31.725 107.828  1.00  0.81           C
ATOM   1505  C   GLU A 187      12.580  30.761 106.676  1.00  0.81           C
ATOM   1506  O   GLU A 187      11.520  30.127 106.584  1.00  0.81           O
ATOM   1507  CB  GLU A 187      12.531  33.167 107.362  1.00  0.81           C
ATOM   1508  CG  GLU A 187      12.528  34.204 108.510  1.00  0.81           C
ATOM   1509  CD  GLU A 187      12.277  35.626 108.003  1.00  0.81           C
ATOM   1510  OE1 GLU A 187      12.199  35.804 106.760  1.00  0.81           O
ATOM   1511  OE2 GLU A 187      12.186  36.541 108.862  1.00  0.81           O
ATOM   1512  N   VAL A 188      13.581  30.599 105.786  1.00  0.89           N
ATOM   1513  CA  VAL A 188      13.539  29.667 104.662  1.00  0.89           C
ATOM   1514  C   VAL A 188      13.360  28.204 105.065  1.00  0.89           C
ATOM   1515  O   VAL A 188      12.492  27.500 104.543  1.00  0.89           O
ATOM   1516  CB  VAL A 188      14.789  29.836 103.799  1.00  0.89           C
ATOM   1517  CG1 VAL A 188      15.095  28.641 102.867  1.00  0.89           C
ATOM   1518  CG2 VAL A 188      14.593  31.114 102.969  1.00  0.89           C
ATOM   1519  N   LEU A 189      14.154  27.717 106.041  1.00  0.85           N
ATOM   1520  CA  LEU A 189      14.070  26.357 106.559  1.00  0.85           C
ATOM   1521  C   LEU A 189      12.743  26.065 107.264  1.00  0.85           C
ATOM   1522  O   LEU A 189      12.144  25.001 107.104  1.00  0.85           O
ATOM   1523  CB  LEU A 189      15.229  26.043 107.539  1.00  0.85           C
ATOM   1524  CG  LEU A 189      16.674  26.162 107.010  1.00  0.85           C
ATOM   1525  CD1 LEU A 189      17.645  25.883 108.172  1.00  0.85           C
ATOM   1526  CD2 LEU A 189      16.951  25.219 105.830  1.00  0.85           C
ATOM   1527  N   ALA A 190      12.242  27.037 108.055  1.00  0.88           N
ATOM   1528  CA  ALA A 190      10.939  26.995 108.697  1.00  0.88           C
ATOM   1529  C   ALA A 190       9.755  26.950 107.708  1.00  0.88           C
ATOM   1530  O   ALA A 190       8.779  26.228 107.928  1.00  0.88           O
ATOM   1531  CB  ALA A 190      10.793  28.192 109.666  1.00  0.88           C
```

ATOM	1532	N	SER	A	191	9.828	27.728	106.597	1.00	0.87	N
ATOM	1533	CA	SER	A	191	8.899	27.718	105.454	1.00	0.87	C
ATOM	1534	C	SER	A	191	8.894	26.375	104.707	1.00	0.87	C
ATOM	1535	O	SER	A	191	7.830	25.800	104.470	1.00	0.87	O
ATOM	1536	CB	SER	A	191	9.134	28.957	104.510	1.00	0.87	C
ATOM	1537	OG	SER	A	191	8.473	28.888	103.241	1.00	0.87	O
ATOM	1538	N	SER	A	192	10.084	25.792	104.408	1.00	0.87	N
ATOM	1539	CA	SER	A	192	10.257	24.451	103.821	1.00	0.87	C
ATOM	1540	C	SER	A	192	9.641	23.357	104.684	1.00	0.87	C
ATOM	1541	O	SER	A	192	8.836	22.558	104.208	1.00	0.87	O
ATOM	1542	CB	SER	A	192	11.764	24.154	103.510	1.00	0.87	C
ATOM	1543	OG	SER	A	192	12.078	22.768	103.366	1.00	0.87	O
ATOM	1544	N	ALA	A	193	9.930	23.353	106.003	1.00	0.91	N
ATOM	1545	CA	ALA	A	193	9.381	22.390	106.941	1.00	0.91	C
ATOM	1546	C	ALA	A	193	7.854	22.437	107.037	1.00	0.91	C
ATOM	1547	O	ALA	A	193	7.177	21.403	107.058	1.00	0.91	O
ATOM	1548	CB	ALA	A	193	10.041	22.592	108.319	1.00	0.91	C
ATOM	1549	N	ARG	A	194	7.277	23.660	107.022	1.00	0.77	N
ATOM	1550	CA	ARG	A	194	5.845	23.892	106.915	1.00	0.77	C
ATOM	1551	C	ARG	A	194	5.226	23.350	105.639	1.00	0.77	C
ATOM	1552	O	ARG	A	194	4.168	22.719	105.657	1.00	0.77	O
ATOM	1553	CB	ARG	A	194	5.538	25.408	106.981	1.00	0.77	C
ATOM	1554	CG	ARG	A	194	5.455	25.960	108.416	1.00	0.77	C
ATOM	1555	CD	ARG	A	194	4.862	27.370	108.485	1.00	0.77	C
ATOM	1556	NE	ARG	A	194	5.333	28.055	109.742	1.00	0.77	N
ATOM	1557	CZ	ARG	A	194	4.989	27.758	111.005	1.00	0.77	C
ATOM	1558	NH1	ARG	A	194	4.086	26.844	111.318	1.00	0.77	N
ATOM	1559	NH2	ARG	A	194	5.579	28.385	112.029	1.00	0.77	N
ATOM	1560	N	GLN	A	195	5.868	23.594	104.485	1.00	0.83	N
ATOM	1561	CA	GLN	A	195	5.384	23.093	103.210	1.00	0.83	C
ATOM	1562	C	GLN	A	195	5.459	21.578	103.067	1.00	0.83	C
ATOM	1563	O	GLN	A	195	4.555	20.934	102.537	1.00	0.83	O
ATOM	1564	CB	GLN	A	195	6.081	23.732	101.989	1.00	0.83	C
ATOM	1565	CG	GLN	A	195	5.367	23.410	100.650	1.00	0.83	C
ATOM	1566	CD	GLN	A	195	3.970	24.035	100.560	1.00	0.83	C
ATOM	1567	OE1	GLN	A	195	3.572	24.943	101.295	1.00	0.83	O
ATOM	1568	NE2	GLN	A	195	3.167	23.500	99.608	1.00	0.83	N
ATOM	1569	N	ARG	A	196	6.552	20.969	103.567	1.00	0.80	N
ATOM	1570	CA	ARG	A	196	6.769	19.537	103.558	1.00	0.80	C
ATOM	1571	C	ARG	A	196	5.702	18.800	104.354	1.00	0.80	C
ATOM	1572	O	ARG	A	196	5.273	17.716	103.955	1.00	0.80	O
ATOM	1573	CB	ARG	A	196	8.174	19.190	104.110	1.00	0.80	C
ATOM	1574	CG	ARG	A	196	8.578	17.701	103.969	1.00	0.80	C
ATOM	1575	CD	ARG	A	196	8.958	17.327	102.526	1.00	0.80	C
ATOM	1576	NE	ARG	A	196	8.928	15.857	102.309	1.00	0.80	N
ATOM	1577	CZ	ARG	A	196	7.835	15.115	102.096	1.00	0.80	C
ATOM	1578	NH1	ARG	A	196	6.598	15.589	102.236	1.00	0.80	N
ATOM	1579	NH2	ARG	A	196	8.021	13.838	101.768	1.00	0.80	N
ATOM	1580	N	LEU	A	197	5.262	19.389	105.494	1.00	0.85	N
ATOM	1581	CA	LEU	A	197	4.088	18.944	106.247	1.00	0.85	C
ATOM	1582	C	LEU	A	197	2.792	19.019	105.447	1.00	0.85	C
ATOM	1583	O	LEU	A	197	2.066	18.030	105.371	1.00	0.85	O
ATOM	1584	CB	LEU	A	197	3.953	19.670	107.621	1.00	0.85	C
ATOM	1585	CG	LEU	A	197	2.666	19.385	108.433	1.00	0.85	C
ATOM	1586	CD1	LEU	A	197	2.509	17.911	108.840	1.00	0.85	C
ATOM	1587	CD2	LEU	A	197	2.604	20.308	109.662	1.00	0.85	C
ATOM	1588	N	LYS	A	198	2.496	20.145	104.753	1.00	0.81	N
ATOM	1589	CA	LYS	A	198	1.292	20.276	103.948	1.00	0.81	C
ATOM	1590	C	LYS	A	198	1.189	19.230	102.872	1.00	0.81	C
ATOM	1591	O	LYS	A	198	0.122	18.661	102.657	1.00	0.81	O
ATOM	1592	CB	LYS	A	198	1.214	21.650	103.270	1.00	0.81	C
ATOM	1593	CG	LYS	A	198	0.952	22.789	104.250	1.00	0.81	C
ATOM	1594	CD	LYS	A	198	0.948	24.124	103.510	1.00	0.81	C
ATOM	1595	CE	LYS	A	198	1.024	25.286	104.479	1.00	0.81	C
ATOM	1596	NZ	LYS	A	198	1.052	26.535	103.707	1.00	0.81	N
ATOM	1597	N	CYS	A	199	2.329	18.935	102.214	1.00	0.89	N
ATOM	1598	CA	CYS	A	199	2.434	17.844	101.264	1.00	0.89	C
ATOM	1599	C	CYS	A	199	2.220	16.474	101.900	1.00	0.89	C
ATOM	1600	O	CYS	A	199	1.421	15.687	101.399	1.00	0.89	O
ATOM	1601	CB	CYS	A	199	3.756	17.856	100.449	1.00	0.89	C
ATOM	1602	SG	CYS	A	199	4.084	19.432	99.603	1.00	0.89	S
ATOM	1603	N	THR	A	200	2.855	16.190	103.069	1.00	0.84	N
ATOM	1604	CA	THR	A	200	2.673	14.932	103.820	1.00	0.84	C
ATOM	1605	C	THR	A	200	1.214	14.717	104.173	1.00	0.84	C
ATOM	1606	O	THR	A	200	0.672	13.629	103.991	1.00	0.84	O
ATOM	1607	CB	THR	A	200	3.515	14.759	105.097	1.00	0.84	C

ATOM	1608	OG1	THR	A	200	4.912	14.726	104.827	1.00	0.84	O
ATOM	1609	CG2	THR	A	200	3.218	13.417	105.796	1.00	0.84	C
ATOM	1610	N	SER	A	201	0.498	15.765	104.613	1.00	0.85	N
ATOM	1611	CA	SER	A	201	−0.923	15.660	104.903	1.00	0.85	C
ATOM	1612	C	SER	A	201	−1.770	15.151	103.746	1.00	0.85	C
ATOM	1613	O	SER	A	201	−2.584	14.256	103.934	1.00	0.85	O
ATOM	1614	CB	SER	A	201	−1.571	17.007	105.318	1.00	0.85	C
ATOM	1615	OG	SER	A	201	−0.855	17.670	106.355	1.00	0.85	O
ATOM	1616	N	ILE	A	202	−1.581	15.673	102.511	1.00	0.85	N
ATOM	1617	CA	ILE	A	202	−2.302	15.179	101.341	1.00	0.85	C
ATOM	1618	C	ILE	A	202	−1.935	13.765	100.919	1.00	0.85	C
ATOM	1619	O	ILE	A	202	−2.798	12.924	100.670	1.00	0.85	O
ATOM	1620	CB	ILE	A	202	−2.155	16.039	100.092	1.00	0.85	C
ATOM	1621	CG1	ILE	A	202	−1.997	17.533	100.414	1.00	0.85	C
ATOM	1622	CG2	ILE	A	202	−3.400	15.759	99.220	1.00	0.85	C
ATOM	1623	CD1	ILE	A	202	−1.959	18.399	99.154	1.00	0.85	C
ATOM	1624	N	GLN	A	203	−0.621	13.469	100.847	1.00	0.80	N
ATOM	1625	CA	GLN	A	203	−0.088	12.269	100.219	1.00	0.80	C
ATOM	1626	C	GLN	A	203	−0.262	11.021	101.076	1.00	0.80	C
ATOM	1627	O	GLN	A	203	−0.090	9.897	100.610	1.00	0.80	O
ATOM	1628	CB	GLN	A	203	1.439	12.423	100.010	1.00	0.80	C
ATOM	1629	CG	GLN	A	203	1.907	13.571	99.087	1.00	0.80	C
ATOM	1630	CD	GLN	A	203	3.421	13.767	99.232	1.00	0.80	C
ATOM	1631	OE1	GLN	A	203	3.968	13.960	100.321	1.00	0.80	O
ATOM	1632	NE2	GLN	A	203	4.146	13.729	98.090	1.00	0.80	N
ATOM	1633	N	LYS	A	204	−0.551	11.232	102.377	1.00	0.81	N
ATOM	1634	CA	LYS	A	204	−0.608	10.192	103.390	1.00	0.81	C
ATOM	1635	C	LYS	A	204	−1.910	10.172	104.225	1.00	0.81	C
ATOM	1636	O	LYS	A	204	−2.271	9.125	104.762	1.00	0.81	O
ATOM	1637	CB	LYS	A	204	0.620	10.403	104.325	1.00	0.81	C
ATOM	1638	CG	LYS	A	204	1.848	9.479	104.159	1.00	0.81	C
ATOM	1639	CD	LYS	A	204	2.378	9.421	102.714	1.00	0.81	C
ATOM	1640	CE	LYS	A	204	3.898	9.376	102.522	1.00	0.81	C
ATOM	1641	NZ	LYS	A	204	4.492	8.334	103.366	1.00	0.81	N
ATOM	1642	N	PHE	A	205	−2.654	11.301	104.342	1.00	0.86	N
ATOM	1643	CA	PHE	A	205	−3.892	11.405	105.113	1.00	0.86	C
ATOM	1644	C	PHE	A	205	−5.034	12.034	104.304	1.00	0.86	C
ATOM	1645	O	PHE	A	205	−6.137	12.241	104.814	1.00	0.86	O
ATOM	1646	CB	PHE	A	205	−3.675	12.275	106.376	1.00	0.86	C
ATOM	1647	CG	PHE	A	205	−2.735	11.600	107.320	1.00	0.86	C
ATOM	1648	CD1	PHE	A	205	−3.223	10.630	108.203	1.00	0.86	C
ATOM	1649	CD2	PHE	A	205	−1.365	11.898	107.325	1.00	0.86	C
ATOM	1650	CE1	PHE	A	205	−2.364	9.977	109.090	1.00	0.86	C
ATOM	1651	CE2	PHE	A	205	−0.502	11.236	108.203	1.00	0.86	C
ATOM	1652	CZ	PHE	A	205	−1.000	10.282	109.092	1.00	0.86	C
ATOM	1653	N	GLY	A	206	−4.822	12.315	102.997	1.00	0.90	N
ATOM	1654	CA	GLY	A	206	−5.856	12.805	102.090	1.00	0.90	C
ATOM	1655	C	GLY	A	206	−6.060	14.309	102.016	1.00	0.90	C
ATOM	1656	O	GLY	A	206	−5.667	15.092	102.881	1.00	0.90	O
ATOM	1657	N	GLU	A	207	−6.760	14.763	100.946	1.00	0.83	N
ATOM	1658	CA	GLU	A	207	−7.078	16.164	100.679	1.00	0.83	C
ATOM	1659	C	GLU	A	207	−7.873	16.831	101.798	1.00	0.83	C
ATOM	1660	O	GLU	A	207	−7.676	17.998	102.130	1.00	0.83	O
ATOM	1661	CB	GLU	A	207	−7.808	16.336	99.321	1.00	0.83	C
ATOM	1662	CG	GLU	A	207	−7.495	17.702	98.652	1.00	0.83	C
ATOM	1663	CD	GLU	A	207	−8.158	17.954	97.287	1.00	0.83	C
ATOM	1664	OE1	GLU	A	207	−8.217	17.018	96.454	1.00	0.83	O
ATOM	1665	OE2	GLU	A	207	−8.526	19.129	97.011	1.00	0.83	O
ATOM	1666	N	ARG	A	208	−8.745	16.022	102.442	1.00	0.75	N
ATOM	1667	CA	ARG	A	208	−9.537	16.294	103.637	1.00	0.75	C
ATOM	1668	C	ARG	A	208	−8.708	16.856	104.788	1.00	0.75	C
ATOM	1669	O	ARG	A	208	−9.102	17.844	105.409	1.00	0.75	O
ATOM	1670	CB	ARG	A	208	−10.265	14.990	104.106	1.00	0.75	C
ATOM	1671	CG	ARG	A	208	−11.258	15.211	105.280	1.00	0.75	C
ATOM	1672	CD	ARG	A	208	−12.187	14.043	105.682	1.00	0.75	C
ATOM	1673	NE	ARG	A	208	−11.583	13.315	106.860	1.00	0.75	N
ATOM	1674	CZ	ARG	A	208	−12.068	13.349	108.121	1.00	0.75	C
ATOM	1675	NH1	ARG	A	208	−13.134	14.048	108.475	1.00	0.75	N
ATOM	1676	NH2	ARG	A	208	−11.528	12.584	109.072	1.00	0.75	N
ATOM	1677	N	ALA	A	209	−7.519	16.266	105.066	1.00	0.87	N
ATOM	1678	CA	ALA	A	209	−6.639	16.668	106.148	1.00	0.87	C
ATOM	1679	C	ALA	A	209	−6.040	18.067	105.950	1.00	0.87	C
ATOM	1680	O	ALA	A	209	−6.013	18.905	106.859	1.00	0.87	O
ATOM	1681	CB	ALA	A	209	−5.548	15.581	106.339	1.00	0.87	C
ATOM	1682	N	LEU	A	210	−5.574	18.370	104.723	1.00	0.84	N
ATOM	1683	CA	LEU	A	210	−5.060	19.675	104.352	1.00	0.84	C

```
ATOM   1684  C   LEU A 210      -6.127  20.738 104.282  1.00  0.84           C
ATOM   1685  O   LEU A 210      -5.941  21.882 104.659  1.00  0.84           O
ATOM   1686  CB  LEU A 210      -4.370  19.633 102.980  1.00  0.84           C
ATOM   1687  CG  LEU A 210      -3.799  20.976 102.497  1.00  0.84           C
ATOM   1688  CD1 LEU A 210      -2.587  21.405 103.330  1.00  0.84           C
ATOM   1689  CD2 LEU A 210      -3.389  20.825 101.040  1.00  0.84           C
ATOM   1690  N   LYS A 211      -7.292  20.354 103.750  1.00  0.81           N
ATOM   1691  CA  LYS A 211      -8.437  21.205 103.659  1.00  0.81           C
ATOM   1692  C   LYS A 211      -8.962  21.633 105.019  1.00  0.81           C
ATOM   1693  O   LYS A 211      -9.319  22.795 105.188  1.00  0.81           O
ATOM   1694  CB  LYS A 211      -9.516  20.425 102.899  1.00  0.81           C
ATOM   1695  CG  LYS A 211     -10.705  21.265 102.461  1.00  0.81           C
ATOM   1696  CD  LYS A 211     -11.786  20.405 101.808  1.00  0.81           C
ATOM   1697  CE  LYS A 211     -13.121  21.138 101.762  1.00  0.81           C
ATOM   1698  NZ  LYS A 211     -14.082  20.328 100.994  1.00  0.81           N
ATOM   1699  N   ALA A 212      -8.991  20.690 105.999  1.00  0.90           N
ATOM   1700  CA  ALA A 212      -9.291  20.871 107.418  1.00  0.90           C
ATOM   1701  C   ALA A 212      -8.295  21.806 108.134  1.00  0.90           C
ATOM   1702  O   ALA A 212      -8.676  22.665 108.937  1.00  0.90           O
ATOM   1703  CB  ALA A 212      -9.354  19.488 108.113  1.00  0.90           C
ATOM   1704  N   TRP A 213      -6.985  21.681 107.802  1.00  0.81           N
ATOM   1705  CA  TRP A 213      -5.892  22.554 108.227  1.00  0.81           C
ATOM   1706  C   TRP A 213      -6.147  23.994 107.812  1.00  0.81           C
ATOM   1707  O   TRP A 213      -6.006  24.913 108.624  1.00  0.81           O
ATOM   1708  CB  TRP A 213      -4.513  22.071 107.665  1.00  0.81           C
ATOM   1709  CG  TRP A 213      -3.286  22.972 107.936  1.00  0.81           C
ATOM   1710  CD1 TRP A 213      -2.443  23.040 109.018  1.00  0.81           C
ATOM   1711  CD2 TRP A 213      -2.906  24.066 107.077  1.00  0.81           C
ATOM   1712  NE1 TRP A 213      -1.593  24.127 108.901  1.00  0.81           N
ATOM   1713  CE2 TRP A 213      -1.886  24.785 107.732  1.00  0.81           C
ATOM   1714  CE3 TRP A 213      -3.394  24.493 105.848  1.00  0.81           C
ATOM   1715  CZ2 TRP A 213      -1.374  25.958 107.179  1.00  0.81           C
ATOM   1716  CZ3 TRP A 213      -2.940  25.711 105.337  1.00  0.81           C
ATOM   1717  CH2 TRP A 213      -1.956  26.445 105.998  1.00  0.81           C
ATOM   1718  N   SER A 214      -6.586  24.216 106.550  1.00  0.87           N
ATOM   1719  CA  SER A 214      -6.900  25.553 106.048  1.00  0.87           C
ATOM   1720  C   SER A 214      -8.089  26.173 106.743  1.00  0.87           C
ATOM   1721  O   SER A 214      -8.090  27.370 107.011  1.00  0.87           O
ATOM   1722  CB  SER A 214      -7.097  25.723 104.515  1.00  0.87           C
ATOM   1723  OG  SER A 214      -5.996  25.213 103.767  1.00  0.87           O
ATOM   1724  N   VAL A 215      -9.130  25.386 107.096  1.00  0.91           N
ATOM   1725  CA  VAL A 215     -10.256  25.896 107.883  1.00  0.91           C
ATOM   1726  C   VAL A 215      -9.798  26.417 109.240  1.00  0.91           C
ATOM   1727  O   VAL A 215     -10.152  27.510 109.679  1.00  0.91           O
ATOM   1728  CB  VAL A 215     -11.351  24.871 108.184  1.00  0.91           C
ATOM   1729  CG1 VAL A 215     -12.608  25.613 108.671  1.00  0.91           C
ATOM   1730  CG2 VAL A 215     -11.717  24.018 106.964  1.00  0.91           C
ATOM   1731  N   ALA A 216      -8.946  25.637 109.931  1.00  0.93           N
ATOM   1732  CA  ALA A 216      -8.387  26.043 111.195  1.00  0.93           C
ATOM   1733  C   ALA A 216      -7.493  27.269 111.105  1.00  0.93           C
ATOM   1734  O   ALA A 216      -7.647  28.193 111.890  1.00  0.93           O
ATOM   1735  CB  ALA A 216      -7.562  24.900 111.801  1.00  0.93           C
ATOM   1736  N   ARG A 217      -6.560  27.323 110.128  1.00  0.79           N
ATOM   1737  CA  ARG A 217      -5.710  28.486 109.929  1.00  0.79           C
ATOM   1738  C   ARG A 217      -6.429  29.758 109.497  1.00  0.79           C
ATOM   1739  O   ARG A 217      -6.140  30.845 109.995  1.00  0.79           O
ATOM   1740  CB  ARG A 217      -4.540  28.229 108.938  1.00  0.79           C
ATOM   1741  CG  ARG A 217      -3.477  29.355 109.029  1.00  0.79           C
ATOM   1742  CD  ARG A 217      -2.219  29.165 108.179  1.00  0.79           C
ATOM   1743  NE  ARG A 217      -1.296  30.362 108.302  1.00  0.79           N
ATOM   1744  CZ  ARG A 217      -0.562  30.668 109.387  1.00  0.79           C
ATOM   1745  NH1 ARG A 217      -0.697  30.007 110.532  1.00  0.79           N
ATOM   1746  NH2 ARG A 217       0.340  31.648 109.314  1.00  0.79           N
ATOM   1747  N   LEU A 218      -7.357  29.677 108.535  1.00  0.90           N
ATOM   1748  CA  LEU A 218      -8.073  30.834 108.030  1.00  0.90           C
ATOM   1749  C   LEU A 218      -9.110  31.418 108.942  1.00  0.90           C
ATOM   1750  O   LEU A 218      -9.218  32.630 109.074  1.00  0.90           O
ATOM   1751  CB  LEU A 218      -8.803  30.469 106.759  1.00  0.90           C
ATOM   1752  CG  LEU A 218      -7.873  30.180 105.582  1.00  0.90           C
ATOM   1753  CD1 LEU A 218      -8.817  29.656 104.503  1.00  0.90           C
ATOM   1754  CD2 LEU A 218      -7.080  31.410 105.117  1.00  0.90           C
ATOM   1755  N   SER A 219      -9.906  30.569 109.603  1.00  0.91           N
ATOM   1756  CA  SER A 219     -10.934  31.020 110.523  1.00  0.91           C
ATOM   1757  C   SER A 219     -10.364  31.720 111.733  1.00  0.91           C
ATOM   1758  O   SER A 219     -10.954  32.638 112.288  1.00  0.91           O
ATOM   1759  CB  SER A 219     -11.774  29.842 111.036  1.00  0.91           C
```

ATOM	1760	OG	SER	A	219	-12.479	29.219	109.970	1.00	0.91	O
ATOM	1761	N	GLN	A	220	-9.173	31.267	112.161	1.00	0.86	N
ATOM	1762	CA	GLN	A	220	-8.358	31.910	113.159	1.00	0.86	C
ATOM	1763	C	GLN	A	220	-7.865	33.306	112.794	1.00	0.86	C
ATOM	1764	O	GLN	A	220	-7.847	34.217	113.619	1.00	0.86	O
ATOM	1765	CB	GLN	A	220	-7.120	31.034	113.412	1.00	0.86	C
ATOM	1766	CG	GLN	A	220	-6.990	30.615	114.881	1.00	0.86	C
ATOM	1767	CD	GLN	A	220	-5.590	30.087	115.189	1.00	0.86	C
ATOM	1768	OE1	GLN	A	220	-4.692	30.046	114.341	1.00	0.86	O
ATOM	1769	NE2	GLN	A	220	-5.388	29.727	116.475	1.00	0.86	N
ATOM	1770	N	LYS	A	221	-7.387	33.477	111.539	1.00	0.87	N
ATOM	1771	CA	LYS	A	221	-6.941	34.760	111.010	1.00	0.87	C
ATOM	1772	C	LYS	A	221	-8.074	35.714	110.748	1.00	0.87	C
ATOM	1773	O	LYS	A	221	-7.996	36.914	111.014	1.00	0.87	O
ATOM	1774	CB	LYS	A	221	-6.231	34.691	109.637	1.00	0.87	C
ATOM	1775	CG	LYS	A	221	-4.914	33.927	109.562	1.00	0.87	C
ATOM	1776	CD	LYS	A	221	-4.317	34.185	108.166	1.00	0.87	C
ATOM	1777	CE	LYS	A	221	-2.810	34.018	108.012	1.00	0.87	C
ATOM	1778	NZ	LYS	A	221	-2.123	34.950	108.922	1.00	0.87	N
ATOM	1779	N	PHE	A	222	-9.130	35.160	110.143	1.00	0.91	N
ATOM	1780	CA	PHE	A	222	-10.256	35.875	109.605	1.00	0.91	C
ATOM	1781	C	PHE	A	222	-11.531	35.537	110.375	1.00	0.91	C
ATOM	1782	O	PHE	A	222	-12.458	34.961	109.794	1.00	0.91	O
ATOM	1783	CB	PHE	A	222	-10.388	35.538	108.088	1.00	0.91	C
ATOM	1784	CG	PHE	A	222	-9.139	35.845	107.283	1.00	0.91	C
ATOM	1785	CD1	PHE	A	222	-8.288	36.921	107.581	1.00	0.91	C
ATOM	1786	CD2	PHE	A	222	-8.816	35.050	106.169	1.00	0.91	C
ATOM	1787	CE1	PHE	A	222	-7.167	37.200	106.793	1.00	0.91	C
ATOM	1788	CE2	PHE	A	222	-7.690	35.322	105.381	1.00	0.91	C
ATOM	1789	CZ	PHE	A	222	-6.866	36.405	105.689	1.00	0.91	C
ATOM	1790	N	PRO	A	223	-11.683	35.883	111.669	1.00	0.92	N
ATOM	1791	CA	PRO	A	223	-12.786	35.402	112.476	1.00	0.92	C
ATOM	1792	C	PRO	A	223	-14.070	36.080	112.139	1.00	0.92	C
ATOM	1793	O	PRO	A	223	-15.107	35.616	112.598	1.00	0.92	O
ATOM	1794	CB	PRO	A	223	-12.382	35.708	113.928	1.00	0.92	C
ATOM	1795	CG	PRO	A	223	-11.426	36.875	113.785	1.00	0.92	C
ATOM	1796	CD	PRO	A	223	-10.658	36.451	112.545	1.00	0.92	C
ATOM	1797	N	LYS	A	224	-14.052	37.158	111.352	1.00	0.86	N
ATOM	1798	CA	LYS	A	224	-15.255	37.846	110.997	1.00	0.86	C
ATOM	1799	C	LYS	A	224	-15.695	37.491	109.586	1.00	0.86	C
ATOM	1800	O	LYS	A	224	-16.824	37.791	109.213	1.00	0.86	O
ATOM	1801	CB	LYS	A	224	-15.059	39.354	111.265	1.00	0.86	C
ATOM	1802	CG	LYS	A	224	-15.254	40.251	110.051	1.00	0.86	C
ATOM	1803	CD	LYS	A	224	-14.902	41.698	110.353	1.00	0.86	C
ATOM	1804	CE	LYS	A	224	-14.721	42.463	109.054	1.00	0.86	C
ATOM	1805	NZ	LYS	A	224	-13.887	43.629	109.358	1.00	0.86	N
ATOM	1806	N	ALA	A	225	-14.863	36.752	108.806	1.00	0.93	N
ATOM	1807	CA	ALA	A	225	-15.304	36.109	107.584	1.00	0.93	C
ATOM	1808	C	ALA	A	225	-16.378	35.108	107.953	1.00	0.93	C
ATOM	1809	O	ALA	A	225	-16.246	34.426	108.970	1.00	0.93	O
ATOM	1810	CB	ALA	A	225	-14.131	35.383	106.884	1.00	0.93	C
ATOM	1811	N	ASP	A	226	-17.483	35.036	107.187	1.00	0.88	N
ATOM	1812	CA	ASP	A	226	-18.522	34.070	107.450	1.00	0.88	C
ATOM	1813	C	ASP	A	226	-18.100	32.693	106.949	1.00	0.88	C
ATOM	1814	O	ASP	A	226	-17.075	32.539	106.283	1.00	0.88	O
ATOM	1815	CB	ASP	A	226	-19.928	34.565	107.000	1.00	0.88	C
ATOM	1816	CG	ASP	A	226	-20.197	34.647	105.502	1.00	0.88	C
ATOM	1817	OD1	ASP	A	226	-21.105	35.439	105.148	1.00	0.88	O
ATOM	1818	OD2	ASP	A	226	-19.578	33.873	104.725	1.00	0.88	O
ATOM	1819	N	PHE	A	227	-18.849	31.621	107.276	1.00	0.89	N
ATOM	1820	CA	PHE	A	227	-18.454	30.292	106.856	1.00	0.89	C
ATOM	1821	C	PHE	A	227	-18.369	30.105	105.333	1.00	0.89	C
ATOM	1822	O	PHE	A	227	-17.445	29.464	104.844	1.00	0.89	O
ATOM	1823	CB	PHE	A	227	-19.320	29.199	107.536	1.00	0.89	C
ATOM	1824	CG	PHE	A	227	-18.770	27.825	107.260	1.00	0.89	C
ATOM	1825	CD1	PHE	A	227	-17.451	27.507	107.616	1.00	0.89	C
ATOM	1826	CD2	PHE	A	227	-19.538	26.859	106.600	1.00	0.89	C
ATOM	1827	CE1	PHE	A	227	-16.924	26.235	107.383	1.00	0.89	C
ATOM	1828	CE2	PHE	A	227	-19.007	25.591	106.337	1.00	0.89	C
ATOM	1829	CZ	PHE	A	227	-17.709	25.270	106.747	1.00	0.89	C
ATOM	1830	N	ALA	A	228	-19.305	30.667	104.534	1.00	0.90	N
ATOM	1831	CA	ALA	A	228	-19.292	30.480	103.094	1.00	0.90	C
ATOM	1832	C	ALA	A	228	-18.068	31.116	102.435	1.00	0.90	C
ATOM	1833	O	ALA	A	228	-17.422	30.497	101.585	1.00	0.90	O
ATOM	1834	CB	ALA	A	228	-20.602	30.974	102.449	1.00	0.90	C
ATOM	1835	N	GLU	A	229	-17.686	32.334	102.872	1.00	0.87	N

```
ATOM   1836  CA  GLU A 229     -16.449  32.989 102.468  1.00  0.87           C
ATOM   1837  C   GLU A 229     -15.174  32.218 102.857  1.00  0.87           C
ATOM   1838  O   GLU A 229     -14.252  32.032 102.057  1.00  0.87           O
ATOM   1839  CB  GLU A 229     -16.391  34.404 103.083  1.00  0.87           C
ATOM   1840  CG  GLU A 229     -15.195  35.273 102.620  1.00  0.87           C
ATOM   1841  CD  GLU A 229     -15.351  35.776 101.188  1.00  0.87           C
ATOM   1842  OE1 GLU A 229     -16.409  36.360 100.850  1.00  0.87           O
ATOM   1843  OE2 GLU A 229     -14.381  35.599 100.407  1.00  0.87           O
ATOM   1844  N   VAL A 230     -15.105  31.684 104.103  1.00  0.92           N
ATOM   1845  CA  VAL A 230     -14.015  30.816 104.556  1.00  0.92           C
ATOM   1846  C   VAL A 230     -13.937  29.526 103.756  1.00  0.92           C
ATOM   1847  O   VAL A 230     -12.870  29.130 103.296  1.00  0.92           O
ATOM   1848  CB  VAL A 230     -14.101  30.455 106.038  1.00  0.92           C
ATOM   1849  CG1 VAL A 230     -12.954  29.505 106.453  1.00  0.92           C
ATOM   1850  CG2 VAL A 230     -14.000  31.720 106.905  1.00  0.92           C
ATOM   1851  N   SER A 231     -15.085  28.866 103.503  1.00  0.88           N
ATOM   1852  CA  SER A 231     -15.189  27.660 102.683  1.00  0.88           C
ATOM   1853  C   SER A 231     -14.667  27.881 101.283  1.00  0.88           C
ATOM   1854  O   SER A 231     -13.995  27.033 100.700  1.00  0.88           O
ATOM   1855  CB  SER A 231     -16.653  27.168 102.527  1.00  0.88           C
ATOM   1856  OG  SER A 231     -17.144  26.509 103.693  1.00  0.88           O
ATOM   1857  N   LYS A 232     -14.975  29.054 100.712  1.00  0.85           N
ATOM   1858  CA  LYS A 232     -14.527  29.480  99.407  1.00  0.85           C
ATOM   1859  C   LYS A 232     -13.025  29.713  99.280  1.00  0.85           C
ATOM   1860  O   LYS A 232     -12.402  29.249  98.323  1.00  0.85           O
ATOM   1861  CB  LYS A 232     -15.279  30.772  99.040  1.00  0.85           C
ATOM   1862  CG  LYS A 232     -15.219  31.166  97.555  1.00  0.85           C
ATOM   1863  CD  LYS A 232     -13.990  32.007  97.148  1.00  0.85           C
ATOM   1864  CE  LYS A 232     -13.937  32.371  95.658  1.00  0.85           C
ATOM   1865  NZ  LYS A 232     -13.471  31.241  94.840  1.00  0.85           N
ATOM   1866  N   ILE A 233     -12.410  30.424 100.259  1.00  0.90           N
ATOM   1867  CA  ILE A 233     -10.962  30.617 100.351  1.00  0.90           C
ATOM   1868  C   ILE A 233     -10.271  29.268 100.550  1.00  0.90           C
ATOM   1869  O   ILE A 233      -9.254  28.993  99.918  1.00  0.90           O
ATOM   1870  CB  ILE A 233     -10.534  31.625 101.435  1.00  0.90           C
ATOM   1871  CG1 ILE A 233     -10.942  33.090 101.109  1.00  0.90           C
ATOM   1872  CG2 ILE A 233      -8.991  31.636 101.647  1.00  0.90           C
ATOM   1873  CD1 ILE A 233     -10.826  34.019 102.328  1.00  0.90           C
ATOM   1874  N   VAL A 234     -10.836  28.363 101.390  1.00  0.90           N
ATOM   1875  CA  VAL A 234     -10.322  27.009 101.613  1.00  0.90           C
ATOM   1876  C   VAL A 234     -10.253  26.180 100.348  1.00  0.90           C
ATOM   1877  O   VAL A 234      -9.235  25.551 100.063  1.00  0.90           O
ATOM   1878  CB  VAL A 234     -11.130  26.247 102.666  1.00  0.90           C
ATOM   1879  CG1 VAL A 234     -10.848  24.722 102.708  1.00  0.90           C
ATOM   1880  CG2 VAL A 234     -10.795  26.855 104.036  1.00  0.90           C
ATOM   1881  N   THR A 235     -11.317  26.205  99.522  1.00  0.88           N
ATOM   1882  CA  THR A 235     -11.369  25.462  98.258  1.00  0.88           C
ATOM   1883  C   THR A 235     -10.270  25.849  97.291  1.00  0.88           C
ATOM   1884  O   THR A 235      -9.583  24.989  96.734  1.00  0.88           O
ATOM   1885  CB  THR A 235     -12.699  25.658  97.538  1.00  0.88           C
ATOM   1886  OG1 THR A 235     -13.764  25.129  98.315  1.00  0.88           O
ATOM   1887  CG2 THR A 235     -12.804  24.956  96.171  1.00  0.88           C
ATOM   1888  N   ASP A 236     -10.050  27.164  97.104  1.00  0.89           N
ATOM   1889  CA  ASP A 236      -8.961  27.666  96.306  1.00  0.89           C
ATOM   1890  C   ASP A 236      -7.577  27.521  96.959  1.00  0.89           C
ATOM   1891  O   ASP A 236      -6.596  27.218  96.280  1.00  0.89           O
ATOM   1892  CB  ASP A 236      -9.285  29.087  95.805  1.00  0.89           C
ATOM   1893  CG  ASP A 236     -10.446  29.048  94.809  1.00  0.89           C
ATOM   1894  OD1 ASP A 236     -10.489  28.187  93.897  1.00  0.89           O
ATOM   1895  OD2 ASP A 236     -11.333  29.926  94.963  1.00  0.89           O
ATOM   1896  N   LEU A 237      -7.431  27.693  98.296  1.00  0.89           N
ATOM   1897  CA  LEU A 237      -6.148  27.494  98.967  1.00  0.89           C
ATOM   1898  C   LEU A 237      -5.658  26.047  98.959  1.00  0.89           C
ATOM   1899  O   LEU A 237      -4.496  25.766  98.661  1.00  0.89           O
ATOM   1900  CB  LEU A 237      -6.111  28.064 100.414  1.00  0.89           C
ATOM   1901  CG  LEU A 237      -4.741  27.996 101.142  1.00  0.89           C
ATOM   1902  CD1 LEU A 237      -3.602  28.599 100.298  1.00  0.89           C
ATOM   1903  CD2 LEU A 237      -4.804  28.678 102.523  1.00  0.89           C
ATOM   1904  N   THR A 238      -6.557  25.073  99.229  1.00  0.86           N
ATOM   1905  CA  THR A 238      -6.254  23.634  99.177  1.00  0.86           C
ATOM   1906  C   THR A 238      -5.837  23.234  97.796  1.00  0.86           C
ATOM   1907  O   THR A 238      -4.916  22.438  97.593  1.00  0.86           O
ATOM   1908  CB  THR A 238      -7.423  22.734  99.529  1.00  0.86           C
ATOM   1909  OG1 THR A 238      -7.834  22.945 100.864  1.00  0.86           O
ATOM   1910  CG2 THR A 238      -7.079  21.237  99.456  1.00  0.86           C
ATOM   1911  N   LYS A 239      -6.501  23.829  96.783  1.00  0.84           N
```

ATOM	1912	CA	LYS	A	239	-6.087	23.673	95.415	1.00	0.84	C
ATOM	1913	C	LYS	A	239	-4.646	24.093	95.183	1.00	0.84	C
ATOM	1914	O	LYS	A	239	-3.853	23.259	94.784	1.00	0.84	O
ATOM	1915	CB	LYS	A	239	-7.020	24.377	94.403	1.00	0.84	C
ATOM	1916	CG	LYS	A	239	-6.573	24.158	92.943	1.00	0.84	C
ATOM	1917	CD	LYS	A	239	-7.545	24.702	91.873	1.00	0.84	C
ATOM	1918	CE	LYS	A	239	-7.066	24.578	90.414	1.00	0.84	C
ATOM	1919	NZ	LYS	A	239	-6.404	23.286	90.174	1.00	0.84	N
ATOM	1920	N	VAL	A	240	-4.273	25.337	95.561	1.00	0.90	N
ATOM	1921	CA	VAL	A	240	-2.926	25.876	95.401	1.00	0.90	C
ATOM	1922	C	VAL	A	240	-1.846	24.971	96.002	1.00	0.90	C
ATOM	1923	O	VAL	A	240	-0.821	24.654	95.386	1.00	0.90	O
ATOM	1924	CB	VAL	A	240	-2.893	27.278	96.020	1.00	0.90	C
ATOM	1925	CG1	VAL	A	240	-1.466	27.824	96.212	1.00	0.90	C
ATOM	1926	CG2	VAL	A	240	-3.675	28.244	95.108	1.00	0.90	C
ATOM	1927	N	HIS	A	241	-2.069	24.468	97.230	1.00	0.83	N
ATOM	1928	CA	HIS	A	241	-1.184	23.512	97.864	1.00	0.83	C
ATOM	1929	C	HIS	A	241	-1.132	22.120	97.253	1.00	0.83	C
ATOM	1930	O	HIS	A	241	-0.055	21.535	97.176	1.00	0.83	O
ATOM	1931	CB	HIS	A	241	-1.463	23.406	99.363	1.00	0.83	C
ATOM	1932	CG	HIS	A	241	-0.996	24.608	100.096	1.00	0.83	C
ATOM	1933	ND1	HIS	A	241	-1.816	25.192	101.032	1.00	0.83	N
ATOM	1934	CD2	HIS	A	241	0.108	25.370	99.896	1.00	0.83	C
ATOM	1935	CE1	HIS	A	241	-1.222	26.310	101.362	1.00	0.83	C
ATOM	1936	NE2	HIS	A	241	-0.043	26.470	100.710	1.00	0.83	N
ATOM	1937	N	LYS	A	242	-2.273	21.557	96.805	1.00	0.84	N
ATOM	1938	CA	LYS	A	242	-2.355	20.272	96.112	1.00	0.84	C
ATOM	1939	C	LYS	A	242	-1.512	20.243	94.836	1.00	0.84	C
ATOM	1940	O	LYS	A	242	-0.748	19.307	94.609	1.00	0.84	O
ATOM	1941	CB	LYS	A	242	-3.848	19.991	95.779	1.00	0.84	C
ATOM	1942	CG	LYS	A	242	-4.164	18.823	94.819	1.00	0.84	C
ATOM	1943	CD	LYS	A	242	-5.644	18.727	94.420	1.00	0.84	C
ATOM	1944	CE	LYS	A	242	-6.139	19.987	93.736	1.00	0.84	C
ATOM	1945	NZ	LYS	A	242	-7.561	19.776	93.430	1.00	0.84	N
ATOM	1946	N	GLU	A	243	-1.596	21.295	94.004	1.00	0.87	N
ATOM	1947	CA	GLU	A	243	-0.770	21.482	92.813	1.00	0.87	C
ATOM	1948	C	GLU	A	243	0.711	21.711	93.082	1.00	0.87	C
ATOM	1949	O	GLU	A	243	1.582	21.061	92.503	1.00	0.87	O
ATOM	1950	CB	GLU	A	243	-1.345	22.644	91.999	1.00	0.87	C
ATOM	1951	CG	GLU	A	243	-2.805	22.358	91.639	1.00	0.87	C
ATOM	1952	CD	GLU	A	243	-3.381	23.561	90.929	1.00	0.87	C
ATOM	1953	OE1	GLU	A	243	-3.568	24.611	91.581	1.00	0.87	O
ATOM	1954	OE2	GLU	A	243	-3.865	23.317	89.792	1.00	0.87	O
ATOM	1955	N	CYS	A	244	1.071	22.589	94.039	1.00	0.91	N
ATOM	1956	CA	CYS	A	244	2.461	22.801	94.424	1.00	0.91	C
ATOM	1957	C	CYS	A	244	3.153	21.575	94.991	1.00	0.91	C
ATOM	1958	O	CYS	A	244	4.323	21.329	94.721	1.00	0.91	O
ATOM	1959	CB	CYS	A	244	2.617	23.939	95.452	1.00	0.91	C
ATOM	1960	SG	CYS	A	244	3.205	25.499	94.718	1.00	0.91	S
ATOM	1961	N	CYS	A	245	2.426	20.772	95.795	1.00	0.89	N
ATOM	1962	CA	CYS	A	245	2.912	19.511	96.319	1.00	0.89	C
ATOM	1963	C	CYS	A	245	3.001	18.411	95.273	1.00	0.89	C
ATOM	1964	O	CYS	A	245	3.724	17.431	95.464	1.00	0.89	O
ATOM	1965	CB	CYS	A	245	2.022	19.029	97.484	1.00	0.89	C
ATOM	1966	SG	CYS	A	245	2.256	20.041	98.975	1.00	0.89	S
ATOM	1967	N	HIS	A	246	2.307	18.561	94.129	1.00	0.84	N
ATOM	1968	CA	HIS	A	246	2.321	17.585	93.050	1.00	0.84	C
ATOM	1969	C	HIS	A	246	3.328	17.945	91.962	1.00	0.84	C
ATOM	1970	O	HIS	A	246	3.646	17.144	91.090	1.00	0.84	O
ATOM	1971	CB	HIS	A	246	0.891	17.425	92.490	1.00	0.84	C
ATOM	1972	CG	HIS	A	246	0.686	16.264	91.568	1.00	0.84	C
ATOM	1973	ND1	HIS	A	246	0.168	16.544	90.335	1.00	0.84	N
ATOM	1974	CD2	HIS	A	246	0.897	14.926	91.691	1.00	0.84	C
ATOM	1975	CE1	HIS	A	246	0.082	15.398	89.712	1.00	0.84	C
ATOM	1976	NE2	HIS	A	246	0.506	14.373	90.488	1.00	0.84	N
ATOM	1977	N	GLY	A	247	3.972	19.131	92.060	1.00	0.88	N
ATOM	1978	CA	GLY	A	247	4.935	19.579	91.060	1.00	0.88	C
ATOM	1979	C	GLY	A	247	4.356	20.413	89.946	1.00	0.88	C
ATOM	1980	O	GLY	A	247	5.106	20.887	89.095	1.00	0.88	O
ATOM	1981	N	ASP	A	248	3.036	20.671	89.953	1.00	0.86	N
ATOM	1982	CA	ASP	A	248	2.344	21.532	89.014	1.00	0.86	C
ATOM	1983	C	ASP	A	248	2.585	23.003	89.384	1.00	0.86	C
ATOM	1984	O	ASP	A	248	1.707	23.774	89.779	1.00	0.86	O
ATOM	1985	CB	ASP	A	248	0.829	21.224	89.013	1.00	0.86	C
ATOM	1986	CG	ASP	A	248	0.477	19.775	88.689	1.00	0.86	C
ATOM	1987	OD1	ASP	A	248	1.322	19.047	88.103	1.00	0.86	O

```
ATOM   1988  OD2 ASP A 248      -0.663  19.381  89.051  1.00  0.86           O
ATOM   1989  N   LEU A 249       3.868  23.413  89.327  1.00  0.87           N
ATOM   1990  CA  LEU A 249       4.363  24.672  89.846  1.00  0.87           C
ATOM   1991  C   LEU A 249       3.832  25.908  89.147  1.00  0.87           C
ATOM   1992  O   LEU A 249       3.577  26.929  89.789  1.00  0.87           O
ATOM   1993  CB  LEU A 249       5.911  24.719  89.895  1.00  0.87           C
ATOM   1994  CG  LEU A 249       6.608  23.595  90.697  1.00  0.87           C
ATOM   1995  CD1 LEU A 249       8.079  23.968  90.917  1.00  0.87           C
ATOM   1996  CD2 LEU A 249       5.960  23.280  92.056  1.00  0.87           C
ATOM   1997  N   LEU A 250       3.667  25.843  87.810  1.00  0.86           N
ATOM   1998  CA  LEU A 250       3.137  26.930  87.015  1.00  0.86           C
ATOM   1999  C   LEU A 250       1.682  27.223  87.345  1.00  0.86           C
ATOM   2000  O   LEU A 250       1.346  28.379  87.610  1.00  0.86           O
ATOM   2001  CB  LEU A 250       3.284  26.657  85.501  1.00  0.86           C
ATOM   2002  CG  LEU A 250       4.713  26.403  84.971  1.00  0.86           C
ATOM   2003  CD1 LEU A 250       4.668  26.106  83.464  1.00  0.86           C
ATOM   2004  CD2 LEU A 250       5.678  27.567  85.237  1.00  0.86           C
ATOM   2005  N   GLU A 251       0.822  26.180  87.424  1.00  0.86           N
ATOM   2006  CA  GLU A 251      -0.550  26.265  87.907  1.00  0.86           C
ATOM   2007  C   GLU A 251      -0.613  26.877  89.301  1.00  0.86           C
ATOM   2008  O   GLU A 251      -1.199  27.941  89.505  1.00  0.86           O
ATOM   2009  CB  GLU A 251      -1.244  24.865  87.915  1.00  0.86           C
ATOM   2010  CG  GLU A 251      -1.367  24.144  86.534  1.00  0.86           C
ATOM   2011  CD  GLU A 251      -0.159  23.364  85.985  1.00  0.86           C
ATOM   2012  OE1 GLU A 251       0.998  23.857  86.103  1.00  0.86           O
ATOM   2013  OE2 GLU A 251      -0.398  22.303  85.351  1.00  0.86           O
ATOM   2014  N   CYS A 252       0.147  26.305  90.260  1.00  0.93           N
ATOM   2015  CA  CYS A 252       0.162  26.727  91.653  1.00  0.93           C
ATOM   2016  C   CYS A 252       0.462  28.205  91.854  1.00  0.93           C
ATOM   2017  O   CYS A 252      -0.140  28.878  92.693  1.00  0.93           O
ATOM   2018  CB  CYS A 252       1.258  25.929  92.409  1.00  0.93           C
ATOM   2019  SG  CYS A 252       1.494  26.421  94.157  1.00  0.93           S
ATOM   2020  N   ALA A 253       1.437  28.738  91.104  1.00  0.90           N
ATOM   2021  CA  ALA A 253       1.820  30.126  91.163  1.00  0.90           C
ATOM   2022  C   ALA A 253       0.794  31.126  90.686  1.00  0.90           C
ATOM   2023  O   ALA A 253       0.594  32.165  91.320  1.00  0.90           O
ATOM   2024  CB  ALA A 253       3.088  30.301  90.328  1.00  0.90           C
ATOM   2025  N   ASP A 254       0.147  30.815  89.556  1.00  0.88           N
ATOM   2026  CA  ASP A 254      -0.927  31.559  88.954  1.00  0.88           C
ATOM   2027  C   ASP A 254      -2.142  31.541  89.895  1.00  0.88           C
ATOM   2028  O   ASP A 254      -2.530  32.586  90.412  1.00  0.88           O
ATOM   2029  CB  ASP A 254      -1.063  30.834  87.584  1.00  0.88           C
ATOM   2030  CG  ASP A 254      -1.777  31.582  86.462  1.00  0.88           C
ATOM   2031  OD1 ASP A 254      -2.148  30.897  85.480  1.00  0.88           O
ATOM   2032  OD2 ASP A 254      -1.784  32.831  86.527  1.00  0.88           O
ATOM   2033  N   ASP A 255      -2.613  30.343  90.334  1.00  0.89           N
ATOM   2034  CA  ASP A 255      -3.755  30.208  91.231  1.00  0.89           C
ATOM   2035  C   ASP A 255      -3.551  30.940  92.568  1.00  0.89           C
ATOM   2036  O   ASP A 255      -4.462  31.547  93.142  1.00  0.89           O
ATOM   2037  CB  ASP A 255      -4.155  28.710  91.389  1.00  0.89           C
ATOM   2038  CG  ASP A 255      -4.838  28.174  90.124  1.00  0.89           C
ATOM   2039  OD1 ASP A 255      -5.440  28.962  89.358  1.00  0.89           O
ATOM   2040  OD2 ASP A 255      -4.807  26.939  89.917  1.00  0.89           O
ATOM   2041  N   ARG A 256      -2.296  30.967  93.069  1.00  0.82           N
ATOM   2042  CA  ARG A 256      -1.910  31.745  94.236  1.00  0.82           C
ATOM   2043  C   ARG A 256      -2.092  33.264  94.068  1.00  0.82           C
ATOM   2044  O   ARG A 256      -2.531  33.965  94.984  1.00  0.82           O
ATOM   2045  CB  ARG A 256      -0.442  31.493  94.653  1.00  0.82           C
ATOM   2046  CG  ARG A 256      -0.195  31.737  96.155  1.00  0.82           C
ATOM   2047  CD  ARG A 256       1.284  31.941  96.454  1.00  0.82           C
ATOM   2048  NE  ARG A 256       1.422  32.216  97.916  1.00  0.82           N
ATOM   2049  CZ  ARG A 256       2.514  32.701  98.492  1.00  0.82           C
ATOM   2050  NH1 ARG A 256       3.597  33.115  97.829  1.00  0.82           N
ATOM   2051  NH2 ARG A 256       2.588  32.804  99.816  1.00  0.82           N
ATOM   2052  N   ALA A 257      -1.723  33.792  92.879  1.00  0.93           N
ATOM   2053  CA  ALA A 257      -1.896  35.164  92.428  1.00  0.93           C
ATOM   2054  C   ALA A 257      -3.358  35.575  92.275  1.00  0.93           C
ATOM   2055  O   ALA A 257      -3.752  36.650  92.729  1.00  0.93           O
ATOM   2056  CB  ALA A 257      -1.194  35.400  91.071  1.00  0.93           C
ATOM   2057  N   ASP A 258      -4.190  34.698  91.673  1.00  0.90           N
ATOM   2058  CA  ASP A 258      -5.639  34.839  91.606  1.00  0.90           C
ATOM   2059  C   ASP A 258      -6.298  34.902  92.968  1.00  0.90           C
ATOM   2060  O   ASP A 258      -7.145  35.759  93.228  1.00  0.90           O
ATOM   2061  CB  ASP A 258      -6.293  33.712  90.768  1.00  0.90           C
ATOM   2062  CG  ASP A 258      -5.973  33.919  89.287  1.00  0.90           C
ATOM   2063  OD1 ASP A 258      -5.487  35.029  88.935  1.00  0.90           O
```

ATOM	2064	OD2	ASP	A	258	-6.318	33.014	88.495	1.00	0.90	O
ATOM	2065	N	LEU	A	259	-5.890	34.037	93.909	1.00	0.92	N
ATOM	2066	CA	LEU	A	259	-6.335	34.111	95.291	1.00	0.92	C
ATOM	2067	C	LEU	A	259	-5.899	35.375	96.060	1.00	0.92	C
ATOM	2068	O	LEU	A	259	-6.676	35.983	96.803	1.00	0.92	O
ATOM	2069	CB	LEU	A	259	-5.914	32.851	96.067	1.00	0.92	C
ATOM	2070	CG	LEU	A	259	-6.556	32.749	97.462	1.00	0.92	C
ATOM	2071	CD1	LEU	A	259	-8.067	32.481	97.354	1.00	0.92	C
ATOM	2072	CD2	LEU	A	259	-5.836	31.690	98.305	1.00	0.92	C
ATOM	2073	N	ALA	A	260	-4.636	35.827	95.890	1.00	0.93	N
ATOM	2074	CA	ALA	A	260	-4.127	37.056	96.473	1.00	0.93	C
ATOM	2075	C	ALA	A	260	-4.871	38.281	95.970	1.00	0.93	C
ATOM	2076	O	ALA	A	260	-5.162	39.218	96.709	1.00	0.93	O
ATOM	2077	CB	ALA	A	260	-2.631	37.237	96.156	1.00	0.93	C
ATOM	2078	N	LYS	A	261	-5.194	38.267	94.665	1.00	0.87	N
ATOM	2079	CA	LYS	A	261	-6.092	39.196	94.020	1.00	0.87	C
ATOM	2080	C	LYS	A	261	-7.518	39.152	94.575	1.00	0.87	C
ATOM	2081	O	LYS	A	261	-8.037	40.187	94.994	1.00	0.87	O
ATOM	2082	CB	LYS	A	261	-6.058	38.937	92.498	1.00	0.87	C
ATOM	2083	CG	LYS	A	261	-6.997	39.846	91.707	1.00	0.87	C
ATOM	2084	CD	LYS	A	261	-6.819	39.740	90.191	1.00	0.87	C
ATOM	2085	CE	LYS	A	261	-7.612	40.838	89.488	1.00	0.87	C
ATOM	2086	NZ	LYS	A	261	-7.511	40.651	88.031	1.00	0.87	N
ATOM	2087	N	TYR	A	262	-8.141	37.951	94.694	1.00	0.91	N
ATOM	2088	CA	TYR	A	262	-9.474	37.782	95.266	1.00	0.91	C
ATOM	2089	C	TYR	A	262	-9.558	38.328	96.698	1.00	0.91	C
ATOM	2090	O	TYR	A	262	-10.448	39.120	97.003	1.00	0.91	O
ATOM	2091	CB	TYR	A	262	-9.932	36.285	95.195	1.00	0.91	C
ATOM	2092	CG	TYR	A	262	-11.342	36.084	95.712	1.00	0.91	C
ATOM	2093	CD1	TYR	A	262	-12.480	36.107	94.884	1.00	0.91	C
ATOM	2094	CD2	TYR	A	262	-11.528	35.913	97.088	1.00	0.91	C
ATOM	2095	CE1	TYR	A	262	-13.770	35.966	95.431	1.00	0.91	C
ATOM	2096	CE2	TYR	A	262	-12.806	35.809	97.633	1.00	0.91	C
ATOM	2097	CZ	TYR	A	262	-13.934	35.817	96.816	1.00	0.91	C
ATOM	2098	OH	TYR	A	262	-15.211	35.675	97.399	1.00	0.91	O
ATOM	2099	N	ILE	A	263	-8.599	37.971	97.591	1.00	0.91	N
ATOM	2100	CA	ILE	A	263	-8.600	38.384	98.999	1.00	0.91	C
ATOM	2101	C	ILE	A	263	-8.577	39.886	99.166	1.00	0.91	C
ATOM	2102	O	ILE	A	263	-9.308	40.478	99.963	1.00	0.91	O
ATOM	2103	CB	ILE	A	263	-7.439	37.743	99.773	1.00	0.91	C
ATOM	2104	CG1	ILE	A	263	-7.872	36.320	100.191	1.00	0.91	C
ATOM	2105	CG2	ILE	A	263	-6.958	38.577	100.994	1.00	0.91	C
ATOM	2106	CD1	ILE	A	263	-6.738	35.476	100.780	1.00	0.91	C
ATOM	2107	N	CYS	A	264	-7.732	40.548	98.371	1.00	0.93	N
ATOM	2108	CA	CYS	A	264	-7.604	41.984	98.362	1.00	0.93	C
ATOM	2109	C	CYS	A	264	-8.819	42.736	97.868	1.00	0.93	C
ATOM	2110	O	CYS	A	264	-9.154	43.791	98.407	1.00	0.93	O
ATOM	2111	CB	CYS	A	264	-6.328	42.376	97.611	1.00	0.93	C
ATOM	2112	SG	CYS	A	264	-4.879	41.828	98.557	1.00	0.93	S
ATOM	2113	N	GLU	A	265	-9.541	42.184	96.882	1.00	0.87	N
ATOM	2114	CA	GLU	A	265	-10.721	42.794	96.311	1.00	0.87	C
ATOM	2115	C	GLU	A	265	-11.980	42.507	97.156	1.00	0.87	C
ATOM	2116	O	GLU	A	265	-13.066	42.998	96.850	1.00	0.87	O
ATOM	2117	CB	GLU	A	265	-10.885	42.291	94.848	1.00	0.87	C
ATOM	2118	CG	GLU	A	265	-9.768	42.787	93.874	1.00	0.87	C
ATOM	2119	CD	GLU	A	265	-9.873	42.272	92.428	1.00	0.87	C
ATOM	2120	OE1	GLU	A	265	-10.841	41.541	92.107	1.00	0.87	O
ATOM	2121	OE2	GLU	A	265	-8.952	42.590	91.620	1.00	0.87	O
ATOM	2122	N	ASN	A	266	-11.851	41.753	98.285	1.00	0.91	N
ATOM	2123	CA	ASN	A	266	-12.951	41.384	99.170	1.00	0.91	C
ATOM	2124	C	ASN	A	266	-12.648	41.651	100.654	1.00	0.91	C
ATOM	2125	O	ASN	A	266	-13.275	41.116	101.562	1.00	0.91	O
ATOM	2126	CB	ASN	A	266	-13.375	39.913	98.954	1.00	0.91	C
ATOM	2127	CG	ASN	A	266	-14.063	39.796	97.598	1.00	0.91	C
ATOM	2128	OD1	ASN	A	266	-15.257	40.069	97.471	1.00	0.91	O
ATOM	2129	ND2	ASN	A	266	-13.303	39.426	96.544	1.00	0.91	N
ATOM	2130	N	GLN	A	267	-11.701	42.555	100.967	1.00	0.86	N
ATOM	2131	CA	GLN	A	267	-11.287	42.891	102.325	1.00	0.86	C
ATOM	2132	C	GLN	A	267	-12.360	43.140	103.373	1.00	0.86	C
ATOM	2133	O	GLN	A	267	-12.309	42.606	104.476	1.00	0.86	O
ATOM	2134	CB	GLN	A	267	-10.426	44.154	102.244	1.00	0.86	C
ATOM	2135	CG	GLN	A	267	-8.969	43.777	101.979	1.00	0.86	C
ATOM	2136	CD	GLN	A	267	-8.155	45.033	101.734	1.00	0.86	C
ATOM	2137	OE1	GLN	A	267	-7.834	45.746	102.682	1.00	0.86	O
ATOM	2138	NE2	GLN	A	267	-7.815	45.322	100.458	1.00	0.86	N
ATOM	2139	N	ALA	A	268	-13.393	43.925	103.041	1.00	0.91	N

ATOM	2140	CA	ALA	A	268	-14.460	44.275	103.955	1.00	0.91	C
ATOM	2141	C	ALA	A	268	-15.235	43.073	104.545	1.00	0.91	C
ATOM	2142	O	ALA	A	268	-15.652	43.111	105.706	1.00	0.91	O
ATOM	2143	CB	ALA	A	268	-15.385	45.300	103.268	1.00	0.91	C
ATOM	2144	N	THR	A	269	-15.394	41.974	103.764	1.00	0.89	N
ATOM	2145	CA	THR	A	269	-16.095	40.746	104.129	1.00	0.89	C
ATOM	2146	C	THR	A	269	-15.144	39.691	104.687	1.00	0.89	C
ATOM	2147	O	THR	A	269	-15.579	38.702	105.275	1.00	0.89	O
ATOM	2148	CB	THR	A	269	-16.847	40.140	102.930	1.00	0.89	C
ATOM	2149	OG1	THR	A	269	-16.036	40.041	101.768	1.00	0.89	O
ATOM	2150	CG2	THR	A	269	-18.021	41.068	102.563	1.00	0.89	C
ATOM	2151	N	ILE	A	270	-13.813	39.922	104.594	1.00	0.89	N
ATOM	2152	CA	ILE	A	270	-12.779	39.003	105.058	1.00	0.89	C
ATOM	2153	C	ILE	A	270	-12.141	39.464	106.381	1.00	0.89	C
ATOM	2154	O	ILE	A	270	-12.250	38.795	107.408	1.00	0.89	O
ATOM	2155	CB	ILE	A	270	-11.718	38.734	103.974	1.00	0.89	C
ATOM	2156	CG1	ILE	A	270	-12.394	38.121	102.716	1.00	0.89	C
ATOM	2157	CG2	ILE	A	270	-10.595	37.815	104.519	1.00	0.89	C
ATOM	2158	CD1	ILE	A	270	-11.452	37.827	101.540	1.00	0.89	C
ATOM	2159	N	SER	A	271	-11.429	40.617	106.384	1.00	0.90	N
ATOM	2160	CA	SER	A	271	-10.601	41.085	107.493	1.00	0.90	C
ATOM	2161	C	SER	A	271	-10.207	42.543	107.253	1.00	0.90	C
ATOM	2162	O	SER	A	271	-10.142	43.017	106.123	1.00	0.90	O
ATOM	2163	CB	SER	A	271	-9.366	40.157	107.748	1.00	0.90	C
ATOM	2164	OG	SER	A	271	-8.234	40.768	108.381	1.00	0.90	O
ATOM	2165	N	SER	A	272	-9.934	43.305	108.336	1.00	0.86	N
ATOM	2166	CA	SER	A	272	-9.601	44.728	108.318	1.00	0.86	C
ATOM	2167	C	SER	A	272	-8.103	45.005	108.363	1.00	0.86	C
ATOM	2168	O	SER	A	272	-7.680	46.155	108.477	1.00	0.86	O
ATOM	2169	CB	SER	A	272	-10.239	45.465	109.532	1.00	0.86	C
ATOM	2170	OG	SER	A	272	-9.764	44.930	110.768	1.00	0.86	O
ATOM	2171	N	LYS	A	273	-7.246	43.964	108.264	1.00	0.82	N
ATOM	2172	CA	LYS	A	273	-5.821	44.099	108.506	1.00	0.82	C
ATOM	2173	C	LYS	A	273	-5.002	44.016	107.234	1.00	0.82	C
ATOM	2174	O	LYS	A	273	-3.772	44.025	107.226	1.00	0.82	O
ATOM	2175	CB	LYS	A	273	-5.358	42.964	109.431	1.00	0.82	C
ATOM	2176	CG	LYS	A	273	-6.255	42.730	110.654	1.00	0.82	C
ATOM	2177	CD	LYS	A	273	-6.277	43.902	111.641	1.00	0.82	C
ATOM	2178	CE	LYS	A	273	-6.754	43.432	113.013	1.00	0.82	C
ATOM	2179	NZ	LYS	A	273	-6.454	44.447	114.039	1.00	0.82	N
ATOM	2180	N	LEU	A	274	-5.704	43.938	106.101	1.00	0.88	N
ATOM	2181	CA	LEU	A	274	-5.140	43.560	104.837	1.00	0.88	C
ATOM	2182	C	LEU	A	274	-4.894	44.737	103.911	1.00	0.88	C
ATOM	2183	O	LEU	A	274	-4.307	44.593	102.846	1.00	0.88	O
ATOM	2184	CB	LEU	A	274	-6.171	42.623	104.184	1.00	0.88	C
ATOM	2185	CG	LEU	A	274	-6.505	41.330	104.963	1.00	0.88	C
ATOM	2186	CD1	LEU	A	274	-7.682	40.583	104.315	1.00	0.88	C
ATOM	2187	CD2	LEU	A	274	-5.283	40.419	105.104	1.00	0.88	C
ATOM	2188	N	GLN	A	275	-5.260	45.958	104.322	1.00	0.81	N
ATOM	2189	CA	GLN	A	275	-5.077	47.162	103.539	1.00	0.81	C
ATOM	2190	C	GLN	A	275	-3.606	47.460	103.192	1.00	0.81	C
ATOM	2191	O	GLN	A	275	-3.274	47.840	102.072	1.00	0.81	O
ATOM	2192	CB	GLN	A	275	-5.784	48.298	104.314	1.00	0.81	C
ATOM	2193	CG	GLN	A	275	-5.756	49.681	103.637	1.00	0.81	C
ATOM	2194	CD	GLN	A	275	-6.560	49.671	102.337	1.00	0.81	C
ATOM	2195	OE1	GLN	A	275	-7.733	49.301	102.309	1.00	0.81	O
ATOM	2196	NE2	GLN	A	275	-5.931	50.106	101.221	1.00	0.81	N
ATOM	2197	N	LYS	A	276	-2.672	47.247	104.149	1.00	0.80	N
ATOM	2198	CA	LYS	A	276	-1.230	47.327	103.929	1.00	0.80	C
ATOM	2199	C	LYS	A	276	-0.678	46.197	103.072	1.00	0.80	C
ATOM	2200	O	LYS	A	276	0.251	46.366	102.278	1.00	0.80	O
ATOM	2201	CB	LYS	A	276	-0.447	47.361	105.261	1.00	0.80	C
ATOM	2202	CG	LYS	A	276	-0.637	48.671	106.038	1.00	0.80	C
ATOM	2203	CD	LYS	A	276	0.268	48.726	107.282	1.00	0.80	C
ATOM	2204	CE	LYS	A	276	0.144	50.038	108.063	1.00	0.80	C
ATOM	2205	NZ	LYS	A	276	0.995	50.010	109.276	1.00	0.80	N
ATOM	2206	N	CYS	A	277	-1.236	44.987	103.256	1.00	0.90	N
ATOM	2207	CA	CYS	A	277	-0.939	43.793	102.480	1.00	0.90	C
ATOM	2208	C	CYS	A	277	-1.264	43.925	101.002	1.00	0.90	C
ATOM	2209	O	CYS	A	277	-0.473	43.551	100.134	1.00	0.90	O
ATOM	2210	CB	CYS	A	277	-1.623	42.547	103.101	1.00	0.90	C
ATOM	2211	SG	CYS	A	277	-0.722	41.852	104.536	1.00	0.90	S
ATOM	2212	N	CYS	A	278	-2.413	44.540	100.691	1.00	0.90	N
ATOM	2213	CA	CYS	A	278	-3.062	44.502	99.405	1.00	0.90	C
ATOM	2214	C	CYS	A	278	-2.670	45.612	98.458	1.00	0.90	C
ATOM	2215	O	CYS	A	278	-3.410	45.979	97.549	1.00	0.90	O

ATOM	2216	CB	CYS	A	278	-4.565	44.506	99.687	1.00	0.90	C
ATOM	2217	SG	CYS	A	278	-5.095	42.874	100.268	1.00	0.90	S
ATOM	2218	N	HIS	A	279	-1.428	46.079	98.651	1.00	0.81	N
ATOM	2219	CA	HIS	A	279	-0.845	47.259	98.066	1.00	0.81	C
ATOM	2220	C	HIS	A	279	0.628	47.057	97.681	1.00	0.81	C
ATOM	2221	O	HIS	A	279	1.315	47.980	97.254	1.00	0.81	O
ATOM	2222	CB	HIS	A	279	-0.940	48.305	99.185	1.00	0.81	C
ATOM	2223	CG	HIS	A	279	-0.623	49.683	98.764	1.00	0.81	C
ATOM	2224	ND1	HIS	A	279	-1.494	50.312	97.907	1.00	0.81	N
ATOM	2225	CD2	HIS	A	279	0.442	50.474	99.040	1.00	0.81	C
ATOM	2226	CE1	HIS	A	279	-0.942	51.478	97.667	1.00	0.81	C
ATOM	2227	NE2	HIS	A	279	0.231	51.633	98.329	1.00	0.81	N
ATOM	2228	N	LYS	A	280	1.162	45.818	97.810	1.00	0.82	N
ATOM	2229	CA	LYS	A	280	2.548	45.497	97.485	1.00	0.82	C
ATOM	2230	C	LYS	A	280	2.646	44.949	96.053	1.00	0.82	C
ATOM	2231	O	LYS	A	280	1.606	44.544	95.513	1.00	0.82	O
ATOM	2232	CB	LYS	A	280	3.133	44.452	98.475	1.00	0.82	C
ATOM	2233	CG	LYS	A	280	2.922	44.742	99.970	1.00	0.82	C
ATOM	2234	CD	LYS	A	280	4.279	44.951	100.666	1.00	0.82	C
ATOM	2235	CE	LYS	A	280	4.205	45.266	102.152	1.00	0.82	C
ATOM	2236	NZ	LYS	A	280	3.596	44.095	102.792	1.00	0.82	N
ATOM	2237	N	PRO	A	281	3.797	44.866	95.360	1.00	0.84	N
ATOM	2238	CA	PRO	A	281	3.947	43.978	94.207	1.00	0.84	C
ATOM	2239	C	PRO	A	281	3.552	42.522	94.503	1.00	0.84	C
ATOM	2240	O	PRO	A	281	3.404	42.117	95.657	1.00	0.84	O
ATOM	2241	CB	PRO	A	281	5.421	44.147	93.759	1.00	0.84	C
ATOM	2242	CG	PRO	A	281	6.081	45.106	94.762	1.00	0.84	C
ATOM	2243	CD	PRO	A	281	5.114	45.123	95.939	1.00	0.84	C
ATOM	2244	N	LEU	A	282	3.334	41.711	93.451	1.00	0.86	N
ATOM	2245	CA	LEU	A	282	2.613	40.446	93.537	1.00	0.86	C
ATOM	2246	C	LEU	A	282	3.123	39.388	94.538	1.00	0.86	C
ATOM	2247	O	LEU	A	282	2.352	38.791	95.288	1.00	0.86	O
ATOM	2248	CB	LEU	A	282	2.543	39.832	92.124	1.00	0.86	C
ATOM	2249	CG	LEU	A	282	1.510	38.701	91.996	1.00	0.86	C
ATOM	2250	CD1	LEU	A	282	0.581	38.987	90.812	1.00	0.86	C
ATOM	2251	CD2	LEU	A	282	2.171	37.322	91.858	1.00	0.86	C
ATOM	2252	N	LEU	A	283	4.449	39.154	94.580	1.00	0.84	N
ATOM	2253	CA	LEU	A	283	5.105	38.237	95.502	1.00	0.84	C
ATOM	2254	C	LEU	A	283	5.035	38.653	96.967	1.00	0.84	C
ATOM	2255	O	LEU	A	283	4.777	37.838	97.852	1.00	0.84	O
ATOM	2256	CB	LEU	A	283	6.588	38.023	95.093	1.00	0.84	C
ATOM	2257	CG	LEU	A	283	6.811	37.247	93.777	1.00	0.84	C
ATOM	2258	CD1	LEU	A	283	8.301	37.215	93.399	1.00	0.84	C
ATOM	2259	CD2	LEU	A	283	6.291	35.809	93.905	1.00	0.84	C
ATOM	2260	N	GLU	A	284	5.246	39.947	97.265	1.00	0.83	N
ATOM	2261	CA	GLU	A	284	5.237	40.440	98.621	1.00	0.83	C
ATOM	2262	C	GLU	A	284	3.853	40.589	99.206	1.00	0.83	C
ATOM	2263	O	GLU	A	284	3.663	40.520	100.419	1.00	0.83	O
ATOM	2264	CB	GLU	A	284	5.865	41.825	98.660	1.00	0.83	C
ATOM	2265	CG	GLU	A	284	7.275	41.935	98.059	1.00	0.83	C
ATOM	2266	CD	GLU	A	284	7.693	43.400	98.148	1.00	0.83	C
ATOM	2267	OE1	GLU	A	284	7.244	44.085	99.108	1.00	0.83	O
ATOM	2268	OE2	GLU	A	284	8.407	43.851	97.222	1.00	0.83	O
ATOM	2269	N	LYS	A	285	2.834	40.800	98.349	1.00	0.85	N
ATOM	2270	CA	LYS	A	285	1.445	40.785	98.758	1.00	0.85	C
ATOM	2271	C	LYS	A	285	1.028	39.415	99.265	1.00	0.85	C
ATOM	2272	O	LYS	A	285	0.526	39.276	100.375	1.00	0.85	O
ATOM	2273	CB	LYS	A	285	0.572	41.239	97.568	1.00	0.85	C
ATOM	2274	CG	LYS	A	285	-0.948	41.123	97.745	1.00	0.85	C
ATOM	2275	CD	LYS	A	285	-1.675	41.939	96.671	1.00	0.85	C
ATOM	2276	CE	LYS	A	285	-1.655	41.281	95.297	1.00	0.85	C
ATOM	2277	NZ	LYS	A	285	-2.097	42.284	94.311	1.00	0.85	N
ATOM	2278	N	SER	A	286	1.325	38.354	98.497	1.00	0.89	N
ATOM	2279	CA	SER	A	286	1.020	36.979	98.857	1.00	0.89	C
ATOM	2280	C	SER	A	286	1.715	36.457	100.113	1.00	0.89	C
ATOM	2281	O	SER	A	286	1.103	35.737	100.903	1.00	0.89	O
ATOM	2282	CB	SER	A	286	1.337	36.037	97.682	1.00	0.89	C
ATOM	2283	OG	SER	A	286	0.565	36.385	96.533	1.00	0.89	O
ATOM	2284	N	HIS	A	287	3.006	36.823	100.315	1.00	0.84	N
ATOM	2285	CA	HIS	A	287	3.758	36.631	101.557	1.00	0.84	C
ATOM	2286	C	HIS	A	287	3.149	37.415	102.714	1.00	0.84	C
ATOM	2287	O	HIS	A	287	2.947	36.910	103.812	1.00	0.84	O
ATOM	2288	CB	HIS	A	287	5.248	37.034	101.397	1.00	0.84	C
ATOM	2289	CG	HIS	A	287	6.074	36.869	102.631	1.00	0.84	C
ATOM	2290	ND1	HIS	A	287	6.527	35.605	102.910	1.00	0.84	N
ATOM	2291	CD2	HIS	A	287	6.405	37.719	103.636	1.00	0.84	C

```
ATOM   2292  CE1 HIS A 287       7.125  35.696 104.074  1.00  0.84           C
ATOM   2293  NE2 HIS A 287       7.073  36.953 104.563  1.00  0.84           N
ATOM   2294  N   CYS A 288       2.763  38.692 102.492  1.00  0.91           N
ATOM   2295  CA  CYS A 288       2.125  39.499 103.528  1.00  0.91           C
ATOM   2296  C   CYS A 288       0.810  38.928 104.027  1.00  0.91           C
ATOM   2297  O   CYS A 288       0.556  38.886 105.226  1.00  0.91           O
ATOM   2298  CB  CYS A 288       1.853  40.940 103.027  1.00  0.91           C
ATOM   2299  SG  CYS A 288       1.289  42.169 104.268  1.00  0.91           S
ATOM   2300  N   ILE A 289      -0.061  38.458 103.111  1.00  0.89           N
ATOM   2301  CA  ILE A 289      -1.344  37.850 103.450  1.00  0.89           C
ATOM   2302  C   ILE A 289      -1.160  36.554 104.268  1.00  0.89           C
ATOM   2303  O   ILE A 289      -1.875  36.291 105.239  1.00  0.89           O
ATOM   2304  CB  ILE A 289      -2.232  37.629 102.210  1.00  0.89           C
ATOM   2305  CG1 ILE A 289      -2.463  38.889 101.329  1.00  0.89           C
ATOM   2306  CG2 ILE A 289      -3.623  37.121 102.642  1.00  0.89           C
ATOM   2307  CD1 ILE A 289      -2.769  38.553  99.858  1.00  0.89           C
ATOM   2308  N   SER A 290      -0.160  35.706 103.937  1.00  0.86           N
ATOM   2309  CA  SER A 290       0.176  34.501 104.696  1.00  0.86           C
ATOM   2310  C   SER A 290       0.737  34.780 106.104  1.00  0.86           C
ATOM   2311  O   SER A 290       0.382  34.073 107.066  1.00  0.86           O
ATOM   2312  CB  SER A 290       1.084  33.536 103.879  1.00  0.86           C
ATOM   2313  OG  SER A 290       2.325  34.152 103.568  1.00  0.86           O
ATOM   2314  N   GLU A 291       1.539  35.869 106.249  1.00  0.82           N
ATOM   2315  CA  GLU A 291       2.118  36.408 107.479  1.00  0.82           C
ATOM   2316  C   GLU A 291       1.104  37.113 108.376  1.00  0.82           C
ATOM   2317  O   GLU A 291       1.292  37.213 109.589  1.00  0.82           O
ATOM   2318  CB  GLU A 291       3.244  37.438 107.173  1.00  0.82           C
ATOM   2319  CG  GLU A 291       4.685  36.872 107.288  1.00  0.82           C
ATOM   2320  CD  GLU A 291       5.777  37.955 107.275  1.00  0.82           C
ATOM   2321  OE1 GLU A 291       6.962  37.562 107.122  1.00  0.82           O
ATOM   2322  OE2 GLU A 291       5.452  39.162 107.402  1.00  0.82           O
ATOM   2323  N   VAL A 292      -0.038  37.568 107.819  1.00  0.89           N
ATOM   2324  CA  VAL A 292      -0.852  38.613 108.442  1.00  0.89           C
ATOM   2325  C   VAL A 292      -1.336  38.419 109.908  1.00  0.89           C
ATOM   2326  O   VAL A 292      -1.594  37.292 110.359  1.00  0.89           O
ATOM   2327  CB  VAL A 292      -1.976  39.061 107.498  1.00  0.89           C
ATOM   2328  CG1 VAL A 292      -3.284  38.258 107.656  1.00  0.89           C
ATOM   2329  CG2 VAL A 292      -2.175  40.589 107.591  1.00  0.89           C
ATOM   2330  N   GLU A 293      -1.468  39.529 110.689  1.00  0.81           N
ATOM   2331  CA  GLU A 293      -2.153  39.592 111.990  1.00  0.81           C
ATOM   2332  C   GLU A 293      -3.614  39.152 111.892  1.00  0.81           C
ATOM   2333  O   GLU A 293      -4.291  39.328 110.880  1.00  0.81           O
ATOM   2334  CB  GLU A 293      -1.974  40.964 112.739  1.00  0.81           C
ATOM   2335  CG  GLU A 293      -2.885  41.244 113.976  1.00  0.81           C
ATOM   2336  CD  GLU A 293      -2.209  41.918 115.179  1.00  0.81           C
ATOM   2337  OE1 GLU A 293      -2.004  41.222 116.206  1.00  0.81           O
ATOM   2338  OE2 GLU A 293      -1.977  43.150 115.084  1.00  0.81           O
ATOM   2339  N   LYS A 294      -4.088  38.472 112.954  1.00  0.83           N
ATOM   2340  CA  LYS A 294      -5.427  37.968 113.148  1.00  0.83           C
ATOM   2341  C   LYS A 294      -6.405  39.091 113.474  1.00  0.83           C
ATOM   2342  O   LYS A 294      -6.041  40.088 114.095  1.00  0.83           O
ATOM   2343  CB  LYS A 294      -5.350  36.856 114.224  1.00  0.83           C
ATOM   2344  CG  LYS A 294      -4.162  35.910 113.941  1.00  0.83           C
ATOM   2345  CD  LYS A 294      -4.321  34.525 114.589  1.00  0.83           C
ATOM   2346  CE  LYS A 294      -3.017  33.824 114.979  1.00  0.83           C
ATOM   2347  NZ  LYS A 294      -2.233  33.537 113.764  1.00  0.83           N
ATOM   2348  N   ASP A 295      -7.670  38.980 113.020  1.00  0.85           N
ATOM   2349  CA  ASP A 295      -8.678  40.004 113.229  1.00  0.85           C
ATOM   2350  C   ASP A 295      -9.334  39.790 114.589  1.00  0.85           C
ATOM   2351  O   ASP A 295      -9.164  38.752 115.231  1.00  0.85           O
ATOM   2352  CB  ASP A 295      -9.672  40.047 112.016  1.00  0.85           C
ATOM   2353  CG  ASP A 295     -10.375  41.393 111.815  1.00  0.85           C
ATOM   2354  OD1 ASP A 295     -10.146  42.298 112.658  1.00  0.85           O
ATOM   2355  OD2 ASP A 295     -11.137  41.549 110.816  1.00  0.85           O
ATOM   2356  N   GLU A 296     -10.062  40.799 115.095  1.00  0.80           N
ATOM   2357  CA  GLU A 296     -10.770  40.672 116.353  1.00  0.80           C
ATOM   2358  C   GLU A 296     -11.912  39.672 116.243  1.00  0.80           C
ATOM   2359  O   GLU A 296     -12.570  39.535 115.210  1.00  0.80           O
ATOM   2360  CB  GLU A 296     -11.219  42.037 116.926  1.00  0.80           C
ATOM   2361  CG  GLU A 296     -11.831  41.986 118.352  1.00  0.80           C
ATOM   2362  CD  GLU A 296     -12.037  43.367 118.980  1.00  0.80           C
ATOM   2363  OE1 GLU A 296     -11.682  44.379 118.320  1.00  0.80           O
ATOM   2364  OE2 GLU A 296     -12.507  43.412 120.146  1.00  0.80           O
ATOM   2365  N   LEU A 297     -12.150  38.910 117.325  1.00  0.82           N
ATOM   2366  CA  LEU A 297     -13.326  38.100 117.510  1.00  0.82           C
ATOM   2367  C   LEU A 297     -14.567  39.000 117.474  1.00  0.82           C
```

ATOM	2368	O	LEU	A	297	-14.556	39.989	118.204	1.00	0.82	O
ATOM	2369	CB	LEU	A	297	-13.203	37.430	118.906	1.00	0.82	C
ATOM	2370	CG	LEU	A	297	-14.333	36.454	119.253	1.00	0.82	C
ATOM	2371	CD1	LEU	A	297	-14.336	35.325	118.226	1.00	0.82	C
ATOM	2372	CD2	LEU	A	297	-14.247	35.890	120.682	1.00	0.82	C
ATOM	2373	N	PRO	A	298	-15.636	38.785	116.701	1.00	0.82	N
ATOM	2374	CA	PRO	A	298	-16.913	39.492	116.842	1.00	0.82	C
ATOM	2375	C	PRO	A	298	-17.396	39.548	118.284	1.00	0.82	C
ATOM	2376	O	PRO	A	298	-16.972	38.747	119.101	1.00	0.82	O
ATOM	2377	CB	PRO	A	298	-17.835	38.786	115.827	1.00	0.82	C
ATOM	2378	CG	PRO	A	298	-16.864	38.336	114.729	1.00	0.82	C
ATOM	2379	CD	PRO	A	298	-15.598	37.965	115.499	1.00	0.82	C
ATOM	2380	N	GLU	A	299	-18.216	40.513	118.680	1.00	0.70	N
ATOM	2381	CA	GLU	A	299	-18.440	40.770	120.089	1.00	0.70	C
ATOM	2382	C	GLU	A	299	-19.480	39.911	120.828	1.00	0.70	C
ATOM	2383	O	GLU	A	299	-19.335	39.577	122.004	1.00	0.70	O
ATOM	2384	CB	GLU	A	299	-18.768	42.266	120.184	1.00	0.70	C
ATOM	2385	CG	GLU	A	299	-19.868	42.772	119.203	1.00	0.70	C
ATOM	2386	CD	GLU	A	299	-19.406	43.323	117.842	1.00	0.70	C
ATOM	2387	OE1	GLU	A	299	-18.950	42.499	117.004	1.00	0.70	O
ATOM	2388	OE2	GLU	A	299	-19.602	44.541	117.615	1.00	0.70	O
ATOM	2389	N	ASN	A	300	-20.558	39.513	120.130	1.00	0.67	N
ATOM	2390	CA	ASN	A	300	-21.744	38.879	120.684	1.00	0.67	C
ATOM	2391	C	ASN	A	300	-21.887	37.511	120.069	1.00	0.67	C
ATOM	2392	O	ASN	A	300	-22.924	37.137	119.521	1.00	0.67	O
ATOM	2393	CB	ASN	A	300	-23.025	39.692	120.378	1.00	0.67	C
ATOM	2394	CG	ASN	A	300	-23.001	40.974	121.194	1.00	0.67	C
ATOM	2395	OD1	ASN	A	300	-22.615	40.977	122.362	1.00	0.67	O
ATOM	2396	ND2	ASN	A	300	-23.462	42.100	120.601	1.00	0.67	N
ATOM	2397	N	LEU	A	301	-20.807	36.718	120.113	1.00	0.77	N
ATOM	2398	CA	LEU	A	301	-20.846	35.386	119.558	1.00	0.77	C
ATOM	2399	C	LEU	A	301	-21.608	34.379	120.376	1.00	0.77	C
ATOM	2400	O	LEU	A	301	-21.293	34.067	121.522	1.00	0.77	O
ATOM	2401	CB	LEU	A	301	-19.473	34.777	119.266	1.00	0.77	C
ATOM	2402	CG	LEU	A	301	-18.788	35.304	118.007	1.00	0.77	C
ATOM	2403	CD1	LEU	A	301	-17.577	36.032	118.521	1.00	0.77	C
ATOM	2404	CD2	LEU	A	301	-18.319	34.182	117.073	1.00	0.77	C
ATOM	2405	N	SER	A	302	-22.599	33.781	119.698	1.00	0.73	N
ATOM	2406	CA	SER	A	302	-23.459	32.716	120.189	1.00	0.73	C
ATOM	2407	C	SER	A	302	-22.672	31.506	120.664	1.00	0.73	C
ATOM	2408	O	SER	A	302	-21.955	30.911	119.863	1.00	0.73	O
ATOM	2409	CB	SER	A	302	-24.404	32.259	119.050	1.00	0.73	C
ATOM	2410	OG	SER	A	302	-25.386	31.333	119.509	1.00	0.73	O
ATOM	2411	N	LEU	A	303	-22.728	31.165	121.976	1.00	0.74	N
ATOM	2412	CA	LEU	A	303	-21.931	30.137	122.662	1.00	0.74	C
ATOM	2413	C	LEU	A	303	-21.829	28.788	121.944	1.00	0.74	C
ATOM	2414	O	LEU	A	303	-22.834	28.229	121.526	1.00	0.74	O
ATOM	2415	CB	LEU	A	303	-22.491	29.902	124.093	1.00	0.74	C
ATOM	2416	CG	LEU	A	303	-21.686	28.976	125.035	1.00	0.74	C
ATOM	2417	CD1	LEU	A	303	-20.317	29.559	125.424	1.00	0.74	C
ATOM	2418	CD2	LEU	A	303	-22.516	28.681	126.297	1.00	0.74	C
ATOM	2419	N	LEU	A	304	-20.610	28.199	121.813	1.00	0.79	N
ATOM	2420	CA	LEU	A	304	-20.359	26.996	121.011	1.00	0.79	C
ATOM	2421	C	LEU	A	304	-21.257	25.780	121.264	1.00	0.79	C
ATOM	2422	O	LEU	A	304	-21.579	25.012	120.364	1.00	0.79	O
ATOM	2423	CB	LEU	A	304	-18.904	26.512	121.212	1.00	0.79	C
ATOM	2424	CG	LEU	A	304	-17.820	27.381	120.563	1.00	0.79	C
ATOM	2425	CD1	LEU	A	304	-16.428	26.853	120.926	1.00	0.79	C
ATOM	2426	CD2	LEU	A	304	-17.954	27.340	119.041	1.00	0.79	C
ATOM	2427	N	ALA	A	305	-21.708	25.574	122.505	1.00	0.83	N
ATOM	2428	CA	ALA	A	305	-22.674	24.554	122.848	1.00	0.83	C
ATOM	2429	C	ALA	A	305	-23.983	24.559	122.007	1.00	0.83	C
ATOM	2430	O	ALA	A	305	-24.496	23.484	121.681	1.00	0.83	O
ATOM	2431	CB	ALA	A	305	-22.948	24.702	124.353	1.00	0.83	C
ATOM	2432	N	ALA	A	306	-24.468	25.759	121.575	1.00	0.81	N
ATOM	2433	CA	ALA	A	306	-25.716	26.022	120.860	1.00	0.81	C
ATOM	2434	C	ALA	A	306	-26.013	25.051	119.707	1.00	0.81	C
ATOM	2435	O	ALA	A	306	-26.963	24.266	119.755	1.00	0.81	O
ATOM	2436	CB	ALA	A	306	-25.698	27.493	120.344	1.00	0.81	C
ATOM	2437	N	ASP	A	307	-25.130	25.020	118.685	1.00	0.83	N
ATOM	2438	CA	ASP	A	307	-25.349	24.251	117.478	1.00	0.83	C
ATOM	2439	C	ASP	A	307	-24.775	22.841	117.556	1.00	0.83	C
ATOM	2440	O	ASP	A	307	-25.154	21.942	116.804	1.00	0.83	O
ATOM	2441	CB	ASP	A	307	-24.619	24.952	116.305	1.00	0.83	C
ATOM	2442	CG	ASP	A	307	-25.332	26.178	115.736	1.00	0.83	C
ATOM	2443	OD1	ASP	A	307	-25.279	26.314	114.486	1.00	0.83	O

ATOM	2444	OD2	ASP	A	307	-25.843	27.020	116.521	1.00	0.83	O
ATOM	2445	N	PHE	A	308	-23.826	22.605	118.485	1.00	0.83	N
ATOM	2446	CA	PHE	A	308	-23.012	21.399	118.453	1.00	0.83	C
ATOM	2447	C	PHE	A	308	-23.238	20.420	119.601	1.00	0.83	C
ATOM	2448	O	PHE	A	308	-22.709	19.301	119.558	1.00	0.83	O
ATOM	2449	CB	PHE	A	308	-21.513	21.770	118.416	1.00	0.83	C
ATOM	2450	CG	PHE	A	308	-21.207	22.592	117.197	1.00	0.83	C
ATOM	2451	CD1	PHE	A	308	-21.228	22.038	115.906	1.00	0.83	C
ATOM	2452	CD2	PHE	A	308	-20.918	23.954	117.334	1.00	0.83	C
ATOM	2453	CE1	PHE	A	308	-20.972	22.836	114.782	1.00	0.83	C
ATOM	2454	CE2	PHE	A	308	-20.695	24.760	116.221	1.00	0.83	C
ATOM	2455	CZ	PHE	A	308	-20.706	24.200	114.947	1.00	0.83	C
ATOM	2456	N	ALA	A	309	-24.030	20.785	120.638	1.00	0.83	N
ATOM	2457	CA	ALA	A	309	-24.280	19.894	121.759	1.00	0.83	C
ATOM	2458	C	ALA	A	309	-25.678	19.913	122.362	1.00	0.83	C
ATOM	2459	O	ALA	A	309	-26.097	18.897	122.900	1.00	0.83	O
ATOM	2460	CB	ALA	A	309	-23.323	20.199	122.927	1.00	0.83	C
ATOM	2461	N	GLU	A	310	-26.459	21.010	122.273	1.00	0.74	N
ATOM	2462	CA	GLU	A	310	-27.835	20.980	122.756	1.00	0.74	C
ATOM	2463	C	GLU	A	310	-28.786	20.796	121.602	1.00	0.74	C
ATOM	2464	O	GLU	A	310	-29.937	20.394	121.777	1.00	0.74	O
ATOM	2465	CB	GLU	A	310	-28.253	22.306	123.427	1.00	0.74	C
ATOM	2466	CG	GLU	A	310	-27.208	22.888	124.402	1.00	0.74	C
ATOM	2467	CD	GLU	A	310	-27.432	24.389	124.610	1.00	0.74	C
ATOM	2468	OE1	GLU	A	310	-26.558	25.178	124.162	1.00	0.74	O
ATOM	2469	OE2	GLU	A	310	-28.469	24.743	125.223	1.00	0.74	O
ATOM	2470	N	ASP	A	311	-28.307	21.060	120.366	1.00	0.77	N
ATOM	2471	CA	ASP	A	311	-29.102	20.908	119.183	1.00	0.77	C
ATOM	2472	C	ASP	A	311	-29.646	19.487	119.020	1.00	0.77	C
ATOM	2473	O	ASP	A	311	-28.934	18.490	118.900	1.00	0.77	O
ATOM	2474	CB	ASP	A	311	-28.377	21.418	117.912	1.00	0.77	C
ATOM	2475	CG	ASP	A	311	-29.401	22.133	117.043	1.00	0.77	C
ATOM	2476	OD1	ASP	A	311	-30.563	21.639	117.043	1.00	0.77	O
ATOM	2477	OD2	ASP	A	311	-29.046	23.137	116.391	1.00	0.77	O
ATOM	2478	N	LYS	A	312	-30.976	19.365	119.038	1.00	0.72	N
ATOM	2479	CA	LYS	A	312	-31.729	18.149	118.905	1.00	0.72	C
ATOM	2480	C	LYS	A	312	-31.731	17.586	117.488	1.00	0.72	C
ATOM	2481	O	LYS	A	312	-32.361	16.563	117.214	1.00	0.72	O
ATOM	2482	CB	LYS	A	312	-33.138	18.420	119.500	1.00	0.72	C
ATOM	2483	CG	LYS	A	312	-33.773	19.811	119.243	1.00	0.72	C
ATOM	2484	CD	LYS	A	312	-34.558	19.949	117.928	1.00	0.72	C
ATOM	2485	CE	LYS	A	312	-35.814	20.821	118.052	1.00	0.72	C
ATOM	2486	NZ	LYS	A	312	-36.984	20.052	117.579	1.00	0.72	N
ATOM	2487	N	GLU	A	313	-30.961	18.233	116.586	1.00	0.77	N
ATOM	2488	CA	GLU	A	313	-30.719	17.852	115.216	1.00	0.77	C
ATOM	2489	C	GLU	A	313	-29.242	17.537	114.905	1.00	0.77	C
ATOM	2490	O	GLU	A	313	-28.926	17.230	113.758	1.00	0.77	O
ATOM	2491	CB	GLU	A	313	-31.260	18.990	114.313	1.00	0.77	C
ATOM	2492	CG	GLU	A	313	-32.797	19.170	114.510	1.00	0.77	C
ATOM	2493	CD	GLU	A	313	-33.509	20.228	113.659	1.00	0.77	C
ATOM	2494	OE1	GLU	A	313	-32.895	20.784	112.721	1.00	0.77	O
ATOM	2495	OE2	GLU	A	313	-34.727	20.429	113.946	1.00	0.77	O
ATOM	2496	N	VAL	A	314	-28.278	17.509	115.881	1.00	0.85	N
ATOM	2497	CA	VAL	A	314	-26.835	17.365	115.571	1.00	0.85	C
ATOM	2498	C	VAL	A	314	-26.501	16.153	114.708	1.00	0.85	C
ATOM	2499	O	VAL	A	314	-25.829	16.274	113.684	1.00	0.85	O
ATOM	2500	CB	VAL	A	314	-25.890	17.275	116.787	1.00	0.85	C
ATOM	2501	CG1	VAL	A	314	-24.411	17.039	116.384	1.00	0.85	C
ATOM	2502	CG2	VAL	A	314	-25.880	18.562	117.623	1.00	0.85	C
ATOM	2503	N	CYS	A	315	-27.008	14.950	115.067	1.00	0.87	N
ATOM	2504	CA	CYS	A	315	-26.779	13.730	114.300	1.00	0.87	C
ATOM	2505	C	CYS	A	315	-27.353	13.815	112.902	1.00	0.87	C
ATOM	2506	O	CYS	A	315	-26.726	13.407	111.926	1.00	0.87	O
ATOM	2507	CB	CYS	A	315	-27.318	12.465	115.013	1.00	0.87	C
ATOM	2508	SG	CYS	A	315	-26.313	12.005	116.450	1.00	0.87	S
ATOM	2509	N	LYS	A	316	-28.557	14.405	112.786	1.00	0.82	N
ATOM	2510	CA	LYS	A	316	-29.214	14.663	111.524	1.00	0.82	C
ATOM	2511	C	LYS	A	316	-28.413	15.614	110.619	1.00	0.82	C
ATOM	2512	O	LYS	A	316	-28.128	15.302	109.468	1.00	0.82	O
ATOM	2513	CB	LYS	A	316	-30.618	15.235	111.837	1.00	0.82	C
ATOM	2514	CG	LYS	A	316	-31.622	15.126	110.680	1.00	0.82	C
ATOM	2515	CD	LYS	A	316	-32.167	16.503	110.238	1.00	0.82	C
ATOM	2516	CE	LYS	A	316	-33.035	16.502	108.975	1.00	0.82	C
ATOM	2517	NZ	LYS	A	316	-34.038	15.433	109.102	1.00	0.82	N
ATOM	2518	N	ASN	A	317	-27.934	16.747	111.176	1.00	0.85	N
ATOM	2519	CA	ASN	A	317	-27.092	17.731	110.504	1.00	0.85	C

ATOM	2520	C	ASN	A	317	-25.772	17.168	110.008	1.00	0.85	C
ATOM	2521	O	ASN	A	317	-25.305	17.458	108.909	1.00	0.85	O
ATOM	2522	CB	ASN	A	317	-26.656	18.863	111.479	1.00	0.85	C
ATOM	2523	CG	ASN	A	317	-27.837	19.674	111.997	1.00	0.85	C
ATOM	2524	OD1	ASN	A	317	-28.917	19.654	111.413	1.00	0.85	O
ATOM	2525	ND2	ASN	A	317	-27.625	20.429	113.104	1.00	0.85	N
ATOM	2526	N	TYR	A	318	-25.125	16.374	110.877	1.00	0.84	N
ATOM	2527	CA	TYR	A	318	-23.884	15.683	110.608	1.00	0.84	C
ATOM	2528	C	TYR	A	318	-23.960	14.609	109.526	1.00	0.84	C
ATOM	2529	O	TYR	A	318	-23.110	14.553	108.648	1.00	0.84	O
ATOM	2530	CB	TYR	A	318	-23.400	15.079	111.947	1.00	0.84	C
ATOM	2531	CG	TYR	A	318	-22.191	14.184	111.850	1.00	0.84	C
ATOM	2532	CD1	TYR	A	318	-20.997	14.660	111.307	1.00	0.84	C
ATOM	2533	CD2	TYR	A	318	-22.235	12.866	112.328	1.00	0.84	C
ATOM	2534	CE1	TYR	A	318	-19.848	13.869	111.314	1.00	0.84	C
ATOM	2535	CE2	TYR	A	318	-21.090	12.052	112.313	1.00	0.84	C
ATOM	2536	CZ	TYR	A	318	-19.880	12.569	111.817	1.00	0.84	C
ATOM	2537	OH	TYR	A	318	-18.663	11.855	111.807	1.00	0.84	O
ATOM	2538	N	ASN	A	319	-24.974	13.726	109.538	1.00	0.84	N
ATOM	2539	CA	ASN	A	319	-25.114	12.689	108.527	1.00	0.84	C
ATOM	2540	C	ASN	A	319	-25.377	13.210	107.121	1.00	0.84	C
ATOM	2541	O	ASN	A	319	-24.912	12.610	106.151	1.00	0.84	O
ATOM	2542	CB	ASN	A	319	-26.191	11.659	108.932	1.00	0.84	C
ATOM	2543	CG	ASN	A	319	-25.647	10.713	110.000	1.00	0.84	C
ATOM	2544	OD1	ASN	A	319	-24.442	10.489	110.145	1.00	0.84	O
ATOM	2545	ND2	ASN	A	319	-26.573	10.056	110.738	1.00	0.84	N
ATOM	2546	N	GLU	A	320	-26.125	14.322	106.978	1.00	0.81	N
ATOM	2547	CA	GLU	A	320	-26.419	14.916	105.686	1.00	0.81	C
ATOM	2548	C	GLU	A	320	-25.199	15.517	104.985	1.00	0.81	C
ATOM	2549	O	GLU	A	320	-25.007	15.301	103.791	1.00	0.81	O
ATOM	2550	CB	GLU	A	320	-27.697	15.799	105.733	1.00	0.81	C
ATOM	2551	CG	GLU	A	320	-28.918	14.970	106.253	1.00	0.81	C
ATOM	2552	CD	GLU	A	320	-30.312	15.621	106.237	1.00	0.81	C
ATOM	2553	OE1	GLU	A	320	-30.461	16.768	105.755	1.00	0.81	O
ATOM	2554	OE2	GLU	A	320	-31.263	14.949	106.740	1.00	0.81	O
ATOM	2555	N	ALA	A	321	-24.281	16.215	105.696	1.00	0.90	N
ATOM	2556	CA	ALA	A	321	-22.953	16.431	105.142	1.00	0.90	C
ATOM	2557	C	ALA	A	321	-21.904	16.423	106.249	1.00	0.90	C
ATOM	2558	O	ALA	A	321	-21.684	17.428	106.927	1.00	0.90	O
ATOM	2559	CB	ALA	A	321	-22.819	17.729	104.303	1.00	0.90	C
ATOM	2560	N	LYS	A	322	-21.207	15.274	106.444	1.00	0.83	N
ATOM	2561	CA	LYS	A	322	-20.280	15.082	107.558	1.00	0.83	C
ATOM	2562	C	LYS	A	322	-19.109	16.055	107.603	1.00	0.83	C
ATOM	2563	O	LYS	A	322	-18.908	16.762	108.589	1.00	0.83	O
ATOM	2564	CB	LYS	A	322	-19.730	13.627	107.589	1.00	0.83	C
ATOM	2565	CG	LYS	A	322	-20.793	12.542	107.863	1.00	0.83	C
ATOM	2566	CD	LYS	A	322	-20.196	11.123	107.991	1.00	0.83	C
ATOM	2567	CE	LYS	A	322	-21.208	10.052	108.433	1.00	0.83	C
ATOM	2568	NZ	LYS	A	322	-20.616	9.119	109.419	1.00	0.83	N
ATOM	2569	N	ASP	A	323	-18.370	16.174	106.485	1.00	0.84	N
ATOM	2570	CA	ASP	A	323	-17.242	17.072	106.332	1.00	0.84	C
ATOM	2571	C	ASP	A	323	-17.608	18.559	106.377	1.00	0.84	C
ATOM	2572	O	ASP	A	323	-16.823	19.390	106.839	1.00	0.84	O
ATOM	2573	CB	ASP	A	323	-16.454	16.740	105.038	1.00	0.84	C
ATOM	2574	CG	ASP	A	323	-15.546	15.516	105.184	1.00	0.84	C
ATOM	2575	OD1	ASP	A	323	-15.523	14.868	106.261	1.00	0.84	O
ATOM	2576	OD2	ASP	A	323	-14.794	15.264	104.207	1.00	0.84	O
ATOM	2577	N	VAL	A	324	-18.807	18.943	105.876	1.00	0.86	N
ATOM	2578	CA	VAL	A	324	-19.289	20.323	105.961	1.00	0.86	C
ATOM	2579	C	VAL	A	324	-19.589	20.703	107.399	1.00	0.86	C
ATOM	2580	O	VAL	A	324	-19.075	21.694	107.906	1.00	0.86	O
ATOM	2581	CB	VAL	A	324	-20.496	20.636	105.069	1.00	0.86	C
ATOM	2582	CG1	VAL	A	324	-20.939	22.111	105.230	1.00	0.86	C
ATOM	2583	CG2	VAL	A	324	-20.110	20.374	103.600	1.00	0.86	C
ATOM	2584	N	PHE	A	325	-20.357	19.858	108.121	1.00	0.88	N
ATOM	2585	CA	PHE	A	325	-20.719	20.052	109.512	1.00	0.88	C
ATOM	2586	C	PHE	A	325	-19.492	20.099	110.430	1.00	0.88	C
ATOM	2587	O	PHE	A	325	-19.401	20.922	111.341	1.00	0.88	O
ATOM	2588	CB	PHE	A	325	-21.741	18.959	109.917	1.00	0.88	C
ATOM	2589	CG	PHE	A	325	-22.319	19.189	111.288	1.00	0.88	C
ATOM	2590	CD1	PHE	A	325	-23.248	20.219	111.508	1.00	0.88	C
ATOM	2591	CD2	PHE	A	325	-21.887	18.426	112.385	1.00	0.88	C
ATOM	2592	CE1	PHE	A	325	-23.723	20.492	112.796	1.00	0.88	C
ATOM	2593	CE2	PHE	A	325	-22.367	18.694	113.672	1.00	0.88	C
ATOM	2594	CZ	PHE	A	325	-23.291	19.723	113.877	1.00	0.88	C
ATOM	2595	N	LEU	A	326	-18.491	19.233	110.178	1.00	0.89	N

ATOM	2596	CA	LEU	A	326	-17.201	19.285	110.847	1.00	0.89	C
ATOM	2597	C	LEU	A	326	-16.336	20.488	110.519	1.00	0.89	C
ATOM	2598	O	LEU	A	326	-15.735	21.098	111.404	1.00	0.89	O
ATOM	2599	CB	LEU	A	326	-16.413	18.002	110.570	1.00	0.89	C
ATOM	2600	CG	LEU	A	326	-16.997	16.776	111.286	1.00	0.89	C
ATOM	2601	CD1	LEU	A	326	-16.267	15.539	110.765	1.00	0.89	C
ATOM	2602	CD2	LEU	A	326	-16.886	16.877	112.816	1.00	0.89	C
ATOM	2603	N	GLY	A	327	-16.267	20.886	109.233	1.00	0.91	N
ATOM	2604	CA	GLY	A	327	-15.621	22.124	108.810	1.00	0.91	C
ATOM	2605	C	GLY	A	327	-16.246	23.370	109.430	1.00	0.91	C
ATOM	2606	O	GLY	A	327	-15.550	24.302	109.818	1.00	0.91	O
ATOM	2607	N	THR	A	328	-17.587	23.352	109.597	1.00	0.88	N
ATOM	2608	CA	THR	A	328	-18.405	24.305	110.364	1.00	0.88	C
ATOM	2609	C	THR	A	328	-18.005	24.366	111.834	1.00	0.88	C
ATOM	2610	O	THR	A	328	-17.701	25.438	112.353	1.00	0.88	O
ATOM	2611	CB	THR	A	328	-19.910	24.024	110.230	1.00	0.88	C
ATOM	2612	OG1	THR	A	328	-20.355	24.183	108.890	1.00	0.88	O
ATOM	2613	CG2	THR	A	328	-20.782	24.968	111.062	1.00	0.88	C
ATOM	2614	N	PHE	A	329	-17.875	23.211	112.530	1.00	0.89	N
ATOM	2615	CA	PHE	A	329	-17.375	23.133	113.901	1.00	0.89	C
ATOM	2616	C	PHE	A	329	-15.967	23.713	114.017	1.00	0.89	C
ATOM	2617	O	PHE	A	329	-15.674	24.506	114.912	1.00	0.89	O
ATOM	2618	CB	PHE	A	329	-17.436	21.660	114.419	1.00	0.89	C
ATOM	2619	CG	PHE	A	329	-16.793	21.472	115.769	1.00	0.89	C
ATOM	2620	CD1	PHE	A	329	-17.442	21.846	116.954	1.00	0.89	C
ATOM	2621	CD2	PHE	A	329	-15.476	20.999	115.844	1.00	0.89	C
ATOM	2622	CE1	PHE	A	329	-16.791	21.734	118.188	1.00	0.89	C
ATOM	2623	CE2	PHE	A	329	-14.819	20.903	117.073	1.00	0.89	C
ATOM	2624	CZ	PHE	A	329	-15.480	21.252	118.251	1.00	0.89	C
ATOM	2625	N	LEU	A	330	-15.074	23.357	113.076	1.00	0.91	N
ATOM	2626	CA	LEU	A	330	-13.703	23.825	113.031	1.00	0.91	C
ATOM	2627	C	LEU	A	330	-13.577	25.336	112.846	1.00	0.91	C
ATOM	2628	O	LEU	A	330	-12.781	25.997	113.506	1.00	0.91	O
ATOM	2629	CB	LEU	A	330	-12.959	23.035	111.939	1.00	0.91	C
ATOM	2630	CG	LEU	A	330	-11.421	23.000	112.001	1.00	0.91	C
ATOM	2631	CD1	LEU	A	330	-10.846	22.849	113.420	1.00	0.91	C
ATOM	2632	CD2	LEU	A	330	-10.963	21.836	111.110	1.00	0.91	C
ATOM	2633	N	TYR	A	331	-14.420	25.916	111.972	1.00	0.91	N
ATOM	2634	CA	TYR	A	331	-14.630	27.344	111.807	1.00	0.91	C
ATOM	2635	C	TYR	A	331	-15.122	28.034	113.070	1.00	0.91	C
ATOM	2636	O	TYR	A	331	-14.545	29.033	113.500	1.00	0.91	O
ATOM	2637	CB	TYR	A	331	-15.637	27.537	110.636	1.00	0.91	C
ATOM	2638	CG	TYR	A	331	-16.311	28.881	110.576	1.00	0.91	C
ATOM	2639	CD1	TYR	A	331	-15.684	29.994	110.007	1.00	0.91	C
ATOM	2640	CD2	TYR	A	331	-17.584	29.040	111.145	1.00	0.91	C
ATOM	2641	CE1	TYR	A	331	-16.322	31.238	109.985	1.00	0.91	C
ATOM	2642	CE2	TYR	A	331	-18.217	30.283	111.134	1.00	0.91	C
ATOM	2643	CZ	TYR	A	331	-17.600	31.374	110.530	1.00	0.91	C
ATOM	2644	OH	TYR	A	331	-18.303	32.584	110.488	1.00	0.91	O
ATOM	2645	N	ASP	A	332	-16.180	27.491	113.702	1.00	0.90	N
ATOM	2646	CA	ASP	A	332	-16.752	28.023	114.913	1.00	0.90	C
ATOM	2647	C	ASP	A	332	-15.803	28.017	116.073	1.00	0.90	C
ATOM	2648	O	ASP	A	332	-15.736	28.980	116.835	1.00	0.90	O
ATOM	2649	CB	ASP	A	332	-18.039	27.258	115.270	1.00	0.90	C
ATOM	2650	CG	ASP	A	332	-19.170	28.128	114.752	1.00	0.90	C
ATOM	2651	OD1	ASP	A	332	-19.812	27.765	113.745	1.00	0.90	O
ATOM	2652	OD2	ASP	A	332	-19.321	29.236	115.360	1.00	0.90	O
ATOM	2653	N	TYR	A	333	-15.055	26.922	116.230	1.00	0.90	N
ATOM	2654	CA	TYR	A	333	-14.068	26.787	117.268	1.00	0.90	C
ATOM	2655	C	TYR	A	333	-12.836	27.661	117.068	1.00	0.90	C
ATOM	2656	O	TYR	A	333	-12.445	28.404	117.967	1.00	0.90	O
ATOM	2657	CB	TYR	A	333	-13.659	25.302	117.375	1.00	0.90	C
ATOM	2658	CG	TYR	A	333	-13.378	24.934	118.799	1.00	0.90	C
ATOM	2659	CD1	TYR	A	333	-12.233	25.390	119.462	1.00	0.90	C
ATOM	2660	CD2	TYR	A	333	-14.321	24.188	119.517	1.00	0.90	C
ATOM	2661	CE1	TYR	A	333	-12.061	25.137	120.827	1.00	0.90	C
ATOM	2662	CE2	TYR	A	333	-14.122	23.886	120.871	1.00	0.90	C
ATOM	2663	CZ	TYR	A	333	-12.996	24.381	121.533	1.00	0.90	C
ATOM	2664	OH	TYR	A	333	-12.802	24.155	122.909	1.00	0.90	O
ATOM	2665	N	ALA	A	334	-12.230	27.623	115.860	1.00	0.95	N
ATOM	2666	CA	ALA	A	334	-10.999	28.308	115.506	1.00	0.95	C
ATOM	2667	C	ALA	A	334	-11.085	29.819	115.585	1.00	0.95	C
ATOM	2668	O	ALA	A	334	-10.165	30.488	116.053	1.00	0.95	O
ATOM	2669	CB	ALA	A	334	-10.569	27.879	114.088	1.00	0.95	C
ATOM	2670	N	ARG	A	335	-12.225	30.400	115.163	1.00	0.86	N
ATOM	2671	CA	ARG	A	335	-12.419	31.831	115.192	1.00	0.86	C

ATOM	2672	C	ARG	A	335	-12.547	32.403	116.586	1.00	0.86	C
ATOM	2673	O	ARG	A	335	-12.241	33.571	116.785	1.00	0.86	O
ATOM	2674	CB	ARG	A	335	-13.623	32.237	114.303	1.00	0.86	C
ATOM	2675	CG	ARG	A	335	-15.025	31.949	114.870	1.00	0.86	C
ATOM	2676	CD	ARG	A	335	-16.108	31.797	113.793	1.00	0.86	C
ATOM	2677	NE	ARG	A	335	-16.407	33.155	113.231	1.00	0.86	N
ATOM	2678	CZ	ARG	A	335	-17.598	33.773	113.202	1.00	0.86	C
ATOM	2679	NH1	ARG	A	335	-18.673	33.289	113.819	1.00	0.86	N
ATOM	2680	NH2	ARG	A	335	-17.700	34.912	112.516	1.00	0.86	N
ATOM	2681	N	ARG	A	336	-12.947	31.586	117.588	1.00	0.84	N
ATOM	2682	CA	ARG	A	336	-13.071	32.027	118.967	1.00	0.84	C
ATOM	2683	C	ARG	A	336	-11.860	31.732	119.794	1.00	0.84	C
ATOM	2684	O	ARG	A	336	-11.747	32.242	120.903	1.00	0.84	O
ATOM	2685	CB	ARG	A	336	-14.221	31.311	119.687	1.00	0.84	C
ATOM	2686	CG	ARG	A	336	-15.521	31.431	118.911	1.00	0.84	C
ATOM	2687	CD	ARG	A	336	-16.646	30.722	119.615	1.00	0.84	C
ATOM	2688	NE	ARG	A	336	-17.656	30.509	118.529	1.00	0.84	N
ATOM	2689	CZ	ARG	A	336	-18.958	30.801	118.654	1.00	0.84	C
ATOM	2690	NH1	ARG	A	336	-19.378	31.420	119.753	1.00	0.84	N
ATOM	2691	NH2	ARG	A	336	-19.853	30.474	117.728	1.00	0.84	N
ATOM	2692	N	HIS	A	337	-10.928	30.930	119.263	1.00	0.82	N
ATOM	2693	CA	HIS	A	337	-9.739	30.594	120.004	1.00	0.82	C
ATOM	2694	C	HIS	A	337	-8.489	30.869	119.177	1.00	0.82	C
ATOM	2695	O	HIS	A	337	-7.803	29.930	118.753	1.00	0.82	O
ATOM	2696	CB	HIS	A	337	-9.796	29.153	120.558	1.00	0.82	C
ATOM	2697	CG	HIS	A	337	-10.879	28.968	121.583	1.00	0.82	C
ATOM	2698	ND1	HIS	A	337	-10.689	29.515	122.828	1.00	0.82	N
ATOM	2699	CD2	HIS	A	337	-12.066	28.304	121.550	1.00	0.82	C
ATOM	2700	CE1	HIS	A	337	-11.742	29.191	123.529	1.00	0.82	C
ATOM	2701	NE2	HIS	A	337	-12.614	28.445	122.810	1.00	0.82	N
ATOM	2702	N	PRO	A	338	-8.122	32.124	118.878	1.00	0.84	N
ATOM	2703	CA	PRO	A	338	-6.895	32.438	118.168	1.00	0.84	C
ATOM	2704	C	PRO	A	338	-5.628	32.242	119.000	1.00	0.84	C
ATOM	2705	O	PRO	A	338	-4.546	32.356	118.431	1.00	0.84	O
ATOM	2706	CB	PRO	A	338	-7.121	33.886	117.707	1.00	0.84	C
ATOM	2707	CG	PRO	A	338	-7.934	34.500	118.843	1.00	0.84	C
ATOM	2708	CD	PRO	A	338	-8.840	33.344	119.272	1.00	0.84	C
ATOM	2709	N	GLU	A	339	-5.718	31.954	120.319	1.00	0.74	N
ATOM	2710	CA	GLU	A	339	-4.604	31.627	121.188	1.00	0.74	C
ATOM	2711	C	GLU	A	339	-4.044	30.253	120.849	1.00	0.74	C
ATOM	2712	O	GLU	A	339	-2.843	29.991	120.938	1.00	0.74	O
ATOM	2713	CB	GLU	A	339	-5.035	31.624	122.687	1.00	0.74	C
ATOM	2714	CG	GLU	A	339	-6.001	32.766	123.107	1.00	0.74	C
ATOM	2715	CD	GLU	A	339	-7.467	32.355	122.932	1.00	0.74	C
ATOM	2716	OE1	GLU	A	339	-7.713	31.492	122.051	1.00	0.74	O
ATOM	2717	OE2	GLU	A	339	-8.325	32.926	123.641	1.00	0.74	O
ATOM	2718	N	TYR	A	340	-4.948	29.321	120.476	1.00	0.79	N
ATOM	2719	CA	TYR	A	340	-4.646	27.909	120.297	1.00	0.79	C
ATOM	2720	C	TYR	A	340	-3.710	27.597	119.130	1.00	0.79	C
ATOM	2721	O	TYR	A	340	-3.676	28.259	118.095	1.00	0.79	O
ATOM	2722	CB	TYR	A	340	-5.903	26.999	120.165	1.00	0.79	C
ATOM	2723	CG	TYR	A	340	-6.806	26.907	121.377	1.00	0.79	C
ATOM	2724	CD1	TYR	A	340	-6.783	27.777	122.483	1.00	0.79	C
ATOM	2725	CD2	TYR	A	340	-7.770	25.886	121.371	1.00	0.79	C
ATOM	2726	CE1	TYR	A	340	-7.729	27.661	123.512	1.00	0.79	C
ATOM	2727	CE2	TYR	A	340	-8.678	25.737	122.428	1.00	0.79	C
ATOM	2728	CZ	TYR	A	340	-8.666	26.631	123.500	1.00	0.79	C
ATOM	2729	OH	TYR	A	340	-9.582	26.501	124.566	1.00	0.79	O
ATOM	2730	N	SER	A	341	-2.897	26.529	119.247	1.00	0.81	N
ATOM	2731	CA	SER	A	341	-2.176	26.025	118.089	1.00	0.81	C
ATOM	2732	C	SER	A	341	-3.137	25.346	117.113	1.00	0.81	C
ATOM	2733	O	SER	A	341	-4.172	24.805	117.512	1.00	0.81	O
ATOM	2734	CB	SER	A	341	-0.974	25.100	118.440	1.00	0.81	C
ATOM	2735	OG	SER	A	341	-1.367	23.875	119.068	1.00	0.81	O
ATOM	2736	N	VAL	A	342	-2.837	25.335	115.792	1.00	0.86	N
ATOM	2737	CA	VAL	A	342	-3.651	24.628	114.794	1.00	0.86	C
ATOM	2738	C	VAL	A	342	-3.723	23.137	115.078	1.00	0.86	C
ATOM	2739	O	VAL	A	342	-4.773	22.509	115.042	1.00	0.86	O
ATOM	2740	CB	VAL	A	342	-3.125	24.791	113.370	1.00	0.86	C
ATOM	2741	CG1	VAL	A	342	-3.877	23.908	112.341	1.00	0.86	C
ATOM	2742	CG2	VAL	A	342	-3.242	26.269	112.976	1.00	0.86	C
ATOM	2743	N	SER	A	343	-2.563	22.558	115.448	1.00	0.82	N
ATOM	2744	CA	SER	A	343	-2.390	21.155	115.769	1.00	0.82	C
ATOM	2745	C	SER	A	343	-3.331	20.743	116.878	1.00	0.82	C
ATOM	2746	O	SER	A	343	-3.941	19.686	116.831	1.00	0.82	O
ATOM	2747	CB	SER	A	343	-0.934	20.833	116.217	1.00	0.82	C

```
ATOM   2748  OG  SER A 343       0.050  21.526 115.441  1.00  0.82           O
ATOM   2749  N   LEU A 344      -3.519  21.614 117.887  1.00  0.84           N
ATOM   2750  CA  LEU A 344      -4.561  21.460 118.877  1.00  0.84           C
ATOM   2751  C   LEU A 344      -6.021  21.543 118.371  1.00  0.84           C
ATOM   2752  O   LEU A 344      -6.841  20.681 118.693  1.00  0.84           O
ATOM   2753  CB  LEU A 344      -4.292  22.484 119.993  1.00  0.84           C
ATOM   2754  CG  LEU A 344      -5.190  22.371 121.230  1.00  0.84           C
ATOM   2755  CD1 LEU A 344      -5.075  20.999 121.921  1.00  0.84           C
ATOM   2756  CD2 LEU A 344      -4.855  23.519 122.188  1.00  0.84           C
ATOM   2757  N   LEU A 345      -6.405  22.535 117.536  1.00  0.90           N
ATOM   2758  CA  LEU A 345      -7.762  22.668 116.994  1.00  0.90           C
ATOM   2759  C   LEU A 345      -8.244  21.533 116.086  1.00  0.90           C
ATOM   2760  O   LEU A 345      -9.410  21.141 116.099  1.00  0.90           O
ATOM   2761  CB  LEU A 345      -7.982  23.979 116.203  1.00  0.90           C
ATOM   2762  CG  LEU A 345      -7.756  25.309 116.941  1.00  0.90           C
ATOM   2763  CD1 LEU A 345      -7.596  26.428 115.901  1.00  0.90           C
ATOM   2764  CD2 LEU A 345      -8.924  25.649 117.877  1.00  0.90           C
ATOM   2765  N   LEU A 346      -7.336  20.983 115.258  1.00  0.87           N
ATOM   2766  CA  LEU A 346      -7.584  19.812 114.434  1.00  0.87           C
ATOM   2767  C   LEU A 346      -7.816  18.578 115.300  1.00  0.87           C
ATOM   2768  O   LEU A 346      -8.617  17.697 114.980  1.00  0.87           O
ATOM   2769  CB  LEU A 346      -6.429  19.553 113.430  1.00  0.87           C
ATOM   2770  CG  LEU A 346      -6.040  20.726 112.499  1.00  0.87           C
ATOM   2771  CD1 LEU A 346      -4.816  20.347 111.641  1.00  0.87           C
ATOM   2772  CD2 LEU A 346      -7.205  21.237 111.644  1.00  0.87           C
ATOM   2773  N   ARG A 347      -7.107  18.516 116.447  1.00  0.80           N
ATOM   2774  CA  ARG A 347      -7.182  17.477 117.460  1.00  0.80           C
ATOM   2775  C   ARG A 347      -8.535  17.492 118.163  1.00  0.80           C
ATOM   2776  O   ARG A 347      -9.180  16.458 118.349  1.00  0.80           O
ATOM   2777  CB  ARG A 347      -6.013  17.636 118.467  1.00  0.80           C
ATOM   2778  CG  ARG A 347      -5.452  16.326 119.045  1.00  0.80           C
ATOM   2779  CD  ARG A 347      -4.387  16.561 120.130  1.00  0.80           C
ATOM   2780  NE  ARG A 347      -3.071  16.831 119.448  1.00  0.80           N
ATOM   2781  CZ  ARG A 347      -2.059  17.504 119.999  1.00  0.80           C
ATOM   2782  NH1 ARG A 347      -2.198  18.218 121.112  1.00  0.80           N
ATOM   2783  NH2 ARG A 347      -0.862  17.510 119.416  1.00  0.80           N
ATOM   2784  N   ILE A 348      -9.003  18.722 118.488  1.00  0.87           N
ATOM   2785  CA  ILE A 348     -10.312  19.062 119.039  1.00  0.87           C
ATOM   2786  C   ILE A 348     -11.407  18.563 118.093  1.00  0.87           C
ATOM   2787  O   ILE A 348     -12.305  17.822 118.494  1.00  0.87           O
ATOM   2788  CB  ILE A 348     -10.398  20.580 119.313  1.00  0.87           C
ATOM   2789  CG1 ILE A 348      -9.525  21.000 120.525  1.00  0.87           C
ATOM   2790  CG2 ILE A 348     -11.843  21.095 119.502  1.00  0.87           C
ATOM   2791  CD1 ILE A 348      -9.424  22.522 120.725  1.00  0.87           C
ATOM   2792  N   ALA A 349     -11.303  18.890 116.783  1.00  0.94           N
ATOM   2793  CA  ALA A 349     -12.236  18.464 115.749  1.00  0.94           C
ATOM   2794  C   ALA A 349     -12.278  16.973 115.448  1.00  0.94           C
ATOM   2795  O   ALA A 349     -13.354  16.394 115.303  1.00  0.94           O
ATOM   2796  CB  ALA A 349     -12.027  19.264 114.453  1.00  0.94           C
ATOM   2797  N   LYS A 350     -11.109  16.307 115.399  1.00  0.82           N
ATOM   2798  CA  LYS A 350     -10.971  14.866 115.263  1.00  0.82           C
ATOM   2799  C   LYS A 350     -11.619  14.111 116.416  1.00  0.82           C
ATOM   2800  O   LYS A 350     -12.253  13.069 116.244  1.00  0.82           O
ATOM   2801  CB  LYS A 350      -9.456  14.543 115.251  1.00  0.82           C
ATOM   2802  CG  LYS A 350      -9.069  13.065 115.080  1.00  0.82           C
ATOM   2803  CD  LYS A 350      -8.994  12.647 113.603  1.00  0.82           C
ATOM   2804  CE  LYS A 350      -8.734  11.153 113.389  1.00  0.82           C
ATOM   2805  NZ  LYS A 350      -7.293  10.870 113.556  1.00  0.82           N
ATOM   2806  N   GLY A 351     -11.464  14.633 117.649  1.00  0.89           N
ATOM   2807  CA  GLY A 351     -12.117  14.067 118.821  1.00  0.89           C
ATOM   2808  C   GLY A 351     -13.608  14.306 118.872  1.00  0.89           C
ATOM   2809  O   GLY A 351     -14.363  13.470 119.363  1.00  0.89           O
ATOM   2810  N   TYR A 352     -14.081  15.453 118.353  1.00  0.86           N
ATOM   2811  CA  TYR A 352     -15.485  15.742 118.143  1.00  0.86           C
ATOM   2812  C   TYR A 352     -16.157  14.811 117.106  1.00  0.86           C
ATOM   2813  O   TYR A 352     -17.271  14.337 117.330  1.00  0.86           O
ATOM   2814  CB  TYR A 352     -15.623  17.248 117.797  1.00  0.86           C
ATOM   2815  CG  TYR A 352     -17.052  17.671 117.649  1.00  0.86           C
ATOM   2816  CD1 TYR A 352     -17.619  17.791 116.376  1.00  0.86           C
ATOM   2817  CD2 TYR A 352     -17.865  17.864 118.771  1.00  0.86           C
ATOM   2818  CE1 TYR A 352     -18.986  18.051 116.230  1.00  0.86           C
ATOM   2819  CE2 TYR A 352     -19.236  18.122 118.624  1.00  0.86           C
ATOM   2820  CZ  TYR A 352     -19.802  18.212 117.350  1.00  0.86           C
ATOM   2821  OH  TYR A 352     -21.187  18.441 117.192  1.00  0.86           O
ATOM   2822  N   GLU A 353     -15.459  14.502 115.979  1.00  0.84           N
ATOM   2823  CA  GLU A 353     -15.868  13.556 114.926  1.00  0.84           C
```

ATOM	2824	C	GLU	A	353	-16.103	12.150	115.475	1.00	0.84	C
ATOM	2825	O	GLU	A	353	-17.161	11.549	115.279	1.00	0.84	O
ATOM	2826	CB	GLU	A	353	-14.781	13.485	113.795	1.00	0.84	C
ATOM	2827	CG	GLU	A	353	-15.060	12.544	112.575	1.00	0.84	C
ATOM	2828	CD	GLU	A	353	-14.020	12.617	111.438	1.00	0.84	C
ATOM	2829	OE1	GLU	A	353	-12.793	12.438	111.675	1.00	0.84	O
ATOM	2830	OE2	GLU	A	353	-14.412	12.839	110.267	1.00	0.84	O
ATOM	2831	N	ALA	A	354	-15.136	11.649	116.279	1.00	0.92	N
ATOM	2832	CA	ALA	A	354	-15.192	10.371	116.973	1.00	0.92	C
ATOM	2833	C	ALA	A	354	-16.373	10.296	117.922	1.00	0.92	C
ATOM	2834	O	ALA	A	354	-17.102	9.308	117.977	1.00	0.92	O
ATOM	2835	CB	ALA	A	354	-13.877	10.143	117.760	1.00	0.92	C
ATOM	2836	N	THR	A	355	-16.639	11.391	118.661	1.00	0.87	N
ATOM	2837	CA	THR	A	355	-17.801	11.446	119.539	1.00	0.87	C
ATOM	2838	C	THR	A	355	-19.119	11.305	118.776	1.00	0.87	C
ATOM	2839	O	THR	A	355	-20.002	10.557	119.181	1.00	0.87	O
ATOM	2840	CB	THR	A	355	-17.818	12.696	120.411	1.00	0.87	C
ATOM	2841	OG1	THR	A	355	-16.673	12.778	121.255	1.00	0.87	O
ATOM	2842	CG2	THR	A	355	-19.012	12.723	121.373	1.00	0.87	C
ATOM	2843	N	LEU	A	356	-19.310	11.981	117.620	1.00	0.88	N
ATOM	2844	CA	LEU	A	356	-20.550	11.811	116.865	1.00	0.88	C
ATOM	2845	C	LEU	A	356	-20.740	10.431	116.248	1.00	0.88	C
ATOM	2846	O	LEU	A	356	-21.844	9.899	116.255	1.00	0.88	O
ATOM	2847	CB	LEU	A	356	-20.833	12.837	115.754	1.00	0.88	C
ATOM	2848	CG	LEU	A	356	-20.682	14.318	116.125	1.00	0.88	C
ATOM	2849	CD1	LEU	A	356	-21.272	15.169	114.995	1.00	0.88	C
ATOM	2850	CD2	LEU	A	356	-21.295	14.718	117.478	1.00	0.88	C
ATOM	2851	N	GLU	A	357	-19.667	9.813	115.714	1.00	0.82	N
ATOM	2852	CA	GLU	A	357	-19.713	8.487	115.108	1.00	0.82	C
ATOM	2853	C	GLU	A	357	-20.246	7.420	116.057	1.00	0.82	C
ATOM	2854	O	GLU	A	357	-21.161	6.664	115.720	1.00	0.82	O
ATOM	2855	CB	GLU	A	357	-18.296	8.067	114.640	1.00	0.82	C
ATOM	2856	CG	GLU	A	357	-17.795	8.788	113.357	1.00	0.82	C
ATOM	2857	CD	GLU	A	357	-18.602	8.465	112.104	1.00	0.82	C
ATOM	2858	OE1	GLU	A	357	-18.413	7.398	111.474	1.00	0.82	O
ATOM	2859	OE2	GLU	A	357	-19.405	9.362	111.710	1.00	0.82	O
ATOM	2860	N	ASP	A	358	-19.721	7.403	117.296	1.00	0.84	N
ATOM	2861	CA	ASP	A	358	-20.120	6.477	118.329	1.00	0.84	C
ATOM	2862	C	ASP	A	358	-21.361	6.894	119.131	1.00	0.84	C
ATOM	2863	O	ASP	A	358	-22.163	6.059	119.560	1.00	0.84	O
ATOM	2864	CB	ASP	A	358	-18.896	6.251	119.234	1.00	0.84	C
ATOM	2865	CG	ASP	A	358	-17.777	5.610	118.419	1.00	0.84	C
ATOM	2866	OD1	ASP	A	358	-18.033	4.535	117.817	1.00	0.84	O
ATOM	2867	OD2	ASP	A	358	-16.656	6.173	118.418	1.00	0.84	O
ATOM	2868	N	CYS	A	359	-21.587	8.209	119.359	1.00	0.86	N
ATOM	2869	CA	CYS	A	359	-22.814	8.707	119.967	1.00	0.86	C
ATOM	2870	C	CYS	A	359	-24.056	8.564	119.118	1.00	0.86	C
ATOM	2871	O	CYS	A	359	-25.114	8.239	119.648	1.00	0.86	O
ATOM	2872	CB	CYS	A	359	-22.768	10.181	120.429	1.00	0.86	C
ATOM	2873	SG	CYS	A	359	-21.658	10.497	121.833	1.00	0.86	S
ATOM	2874	N	CYS	A	360	-23.959	8.813	117.796	1.00	0.86	N
ATOM	2875	CA	CYS	A	360	-25.076	8.719	116.866	1.00	0.86	C
ATOM	2876	C	CYS	A	360	-25.471	7.276	116.562	1.00	0.86	C
ATOM	2877	O	CYS	A	360	-26.550	7.036	116.028	1.00	0.86	O
ATOM	2878	CB	CYS	A	360	-24.814	9.462	115.525	1.00	0.86	C
ATOM	2879	SG	CYS	A	360	-24.602	11.282	115.631	1.00	0.86	S
ATOM	2880	N	ALA	A	361	-24.604	6.292	116.888	1.00	0.84	N
ATOM	2881	CA	ALA	A	361	-24.934	4.881	116.959	1.00	0.84	C
ATOM	2882	C	ALA	A	361	-25.877	4.437	118.096	1.00	0.84	C
ATOM	2883	O	ALA	A	361	-26.723	3.567	117.905	1.00	0.84	O
ATOM	2884	CB	ALA	A	361	-23.640	4.064	117.091	1.00	0.84	C
ATOM	2885	N	LYS	A	362	-25.712	4.988	119.326	1.00	0.80	N
ATOM	2886	CA	LYS	A	362	-26.599	4.795	120.478	1.00	0.80	C
ATOM	2887	C	LYS	A	362	-28.051	5.235	120.194	1.00	0.80	C
ATOM	2888	O	LYS	A	362	-28.281	6.102	119.363	1.00	0.80	O
ATOM	2889	CB	LYS	A	362	-26.096	5.574	121.736	1.00	0.80	C
ATOM	2890	CG	LYS	A	362	-24.751	5.105	122.317	1.00	0.80	C
ATOM	2891	CD	LYS	A	362	-24.179	6.084	123.364	1.00	0.80	C
ATOM	2892	CE	LYS	A	362	-24.779	6.002	124.780	1.00	0.80	C
ATOM	2893	NZ	LYS	A	362	-25.800	7.050	125.063	1.00	0.80	N
ATOM	2894	N	ASP	A	363	-29.063	4.660	120.901	1.00	0.82	N
ATOM	2895	CA	ASP	A	363	-30.460	5.089	120.837	1.00	0.82	C
ATOM	2896	C	ASP	A	363	-30.733	6.492	121.404	1.00	0.82	C
ATOM	2897	O	ASP	A	363	-31.519	7.265	120.863	1.00	0.82	O
ATOM	2898	CB	ASP	A	363	-31.387	4.039	121.502	1.00	0.82	C
ATOM	2899	CG	ASP	A	363	-32.800	4.205	120.950	1.00	0.82	C

ATOM	2900	OD1	ASP	A	363	-32.977	3.940	119.735	1.00	0.82	O
ATOM	2901	OD2	ASP	A	363	-33.699	4.576	121.743	1.00	0.82	O
ATOM	2902	N	ASP	A	364	-30.035	6.865	122.502	1.00	0.85	N
ATOM	2903	CA	ASP	A	364	-30.013	8.219	123.029	1.00	0.85	C
ATOM	2904	C	ASP	A	364	-28.657	8.864	122.644	1.00	0.85	C
ATOM	2905	O	ASP	A	364	-27.668	8.739	123.405	1.00	0.85	O
ATOM	2906	CB	ASP	A	364	-30.375	8.196	124.553	1.00	0.85	C
ATOM	2907	CG	ASP	A	364	-30.674	9.577	125.128	1.00	0.85	C
ATOM	2908	OD1	ASP	A	364	-30.433	10.591	124.423	1.00	0.85	O
ATOM	2909	OD2	ASP	A	364	-31.073	9.618	126.319	1.00	0.85	O
ATOM	2910	N	PRO	A	365	-28.508	9.471	121.441	1.00	0.84	N
ATOM	2911	CA	PRO	A	365	-27.416	10.375	121.095	1.00	0.84	C
ATOM	2912	C	PRO	A	365	-27.343	11.650	121.928	1.00	0.84	C
ATOM	2913	O	PRO	A	365	-26.218	11.885	122.383	1.00	0.84	O
ATOM	2914	CB	PRO	A	365	-27.473	10.529	119.557	1.00	0.84	C
ATOM	2915	CG	PRO	A	365	-28.860	10.063	119.119	1.00	0.84	C
ATOM	2916	CD	PRO	A	365	-29.322	9.151	120.251	1.00	0.84	C
ATOM	2917	N	PRO	A	366	-28.361	12.470	122.203	1.00	0.83	N
ATOM	2918	CA	PRO	A	366	-28.246	13.657	123.048	1.00	0.83	C
ATOM	2919	C	PRO	A	366	-27.654	13.395	124.414	1.00	0.83	C
ATOM	2920	O	PRO	A	366	-26.793	14.158	124.837	1.00	0.83	O
ATOM	2921	CB	PRO	A	366	-29.650	14.271	123.091	1.00	0.83	C
ATOM	2922	CG	PRO	A	366	-30.348	13.766	121.822	1.00	0.83	C
ATOM	2923	CD	PRO	A	366	-29.599	12.492	121.428	1.00	0.83	C
ATOM	2924	N	ALA	A	367	-28.029	12.303	125.107	1.00	0.87	N
ATOM	2925	CA	ALA	A	367	-27.388	11.965	126.368	1.00	0.87	C
ATOM	2926	C	ALA	A	367	-25.864	11.746	126.282	1.00	0.87	C
ATOM	2927	O	ALA	A	367	-25.112	12.086	127.193	1.00	0.87	O
ATOM	2928	CB	ALA	A	367	-28.063	10.734	126.995	1.00	0.87	C
ATOM	2929	N	CYS	A	368	-25.388	11.152	125.165	1.00	0.84	N
ATOM	2930	CA	CYS	A	368	-23.985	10.920	124.864	1.00	0.84	C
ATOM	2931	C	CYS	A	368	-23.226	12.152	124.413	1.00	0.84	C
ATOM	2932	O	CYS	A	368	-22.135	12.420	124.907	1.00	0.84	O
ATOM	2933	CB	CYS	A	368	-23.921	9.844	123.753	1.00	0.84	C
ATOM	2934	SG	CYS	A	368	-22.288	9.205	123.258	1.00	0.84	S
ATOM	2935	N	TYR	A	369	-23.779	12.939	123.462	1.00	0.80	N
ATOM	2936	CA	TYR	A	369	-23.019	14.026	122.877	1.00	0.80	C
ATOM	2937	C	TYR	A	369	-23.192	15.366	123.581	1.00	0.80	C
ATOM	2938	O	TYR	A	369	-22.356	16.250	123.412	1.00	0.80	O
ATOM	2939	CB	TYR	A	369	-23.165	14.120	121.324	1.00	0.80	C
ATOM	2940	CG	TYR	A	369	-24.479	14.632	120.802	1.00	0.80	C
ATOM	2941	CD1	TYR	A	369	-25.381	13.783	120.152	1.00	0.80	C
ATOM	2942	CD2	TYR	A	369	-24.808	15.990	120.897	1.00	0.80	C
ATOM	2943	CE1	TYR	A	369	-26.625	14.252	119.714	1.00	0.80	C
ATOM	2944	CE2	TYR	A	369	-26.063	16.454	120.478	1.00	0.80	C
ATOM	2945	CZ	TYR	A	369	-26.990	15.578	119.911	1.00	0.80	C
ATOM	2946	OH	TYR	A	369	-28.287	15.985	119.538	1.00	0.80	O
ATOM	2947	N	ALA	A	370	-24.209	15.546	124.456	1.00	0.80	N
ATOM	2948	CA	ALA	A	370	-24.431	16.780	125.200	1.00	0.80	C
ATOM	2949	C	ALA	A	370	-23.242	17.196	126.073	1.00	0.80	C
ATOM	2950	O	ALA	A	370	-22.968	18.376	126.294	1.00	0.80	O
ATOM	2951	CB	ALA	A	370	-25.690	16.637	126.080	1.00	0.80	C
ATOM	2952	N	THR	A	371	-22.478	16.206	126.564	1.00	0.77	N
ATOM	2953	CA	THR	A	371	-21.352	16.361	127.466	1.00	0.77	C
ATOM	2954	C	THR	A	371	-19.990	16.394	126.774	1.00	0.77	C
ATOM	2955	O	THR	A	371	-18.950	16.446	127.432	1.00	0.77	O
ATOM	2956	CB	THR	A	371	-21.326	15.217	128.465	1.00	0.77	C
ATOM	2957	OG1	THR	A	371	-21.535	13.981	127.793	1.00	0.77	O
ATOM	2958	CG2	THR	A	371	-22.484	15.421	129.451	1.00	0.77	C
ATOM	2959	N	VAL	A	372	-19.938	16.420	125.418	1.00	0.81	N
ATOM	2960	CA	VAL	A	372	-18.691	16.427	124.645	1.00	0.81	C
ATOM	2961	C	VAL	A	372	-17.768	17.596	124.925	1.00	0.81	C
ATOM	2962	O	VAL	A	372	-16.548	17.446	124.922	1.00	0.81	O
ATOM	2963	CB	VAL	A	372	-18.905	16.335	123.130	1.00	0.81	C
ATOM	2964	CG1	VAL	A	372	-19.608	17.588	122.574	1.00	0.81	C
ATOM	2965	CG2	VAL	A	372	-17.578	16.079	122.369	1.00	0.81	C
ATOM	2966	N	PHE	A	373	-18.313	18.804	125.191	1.00	0.78	N
ATOM	2967	CA	PHE	A	373	-17.500	19.983	125.424	1.00	0.78	C
ATOM	2968	C	PHE	A	373	-16.804	19.997	126.762	1.00	0.78	C
ATOM	2969	O	PHE	A	373	-15.800	20.670	126.900	1.00	0.78	O
ATOM	2970	CB	PHE	A	373	-18.264	21.312	125.441	1.00	0.78	C
ATOM	2971	CG	PHE	A	373	-18.493	21.744	124.057	1.00	0.78	C
ATOM	2972	CD1	PHE	A	373	-19.664	21.374	123.406	1.00	0.78	C
ATOM	2973	CD2	PHE	A	373	-17.527	22.512	123.395	1.00	0.78	C
ATOM	2974	CE1	PHE	A	373	-19.899	21.810	122.103	1.00	0.78	C
ATOM	2975	CE2	PHE	A	373	-17.768	22.965	122.098	1.00	0.78	C

ATOM	2976	CZ	PHE	A	373	-18.962	22.623	121.455	1.00	0.78	C
ATOM	2977	N	GLU	A	374	-17.329	19.208	127.764	1.00	0.73	N
ATOM	2978	CA	GLU	A	374	-16.455	18.846	128.869	1.00	0.73	C
ATOM	2979	C	GLU	A	374	-15.311	18.029	128.293	1.00	0.73	C
ATOM	2980	O	GLU	A	374	-14.255	18.576	128.157	1.00	0.73	O
ATOM	2981	CB	GLU	A	374	-17.072	18.342	130.209	1.00	0.73	C
ATOM	2982	CG	GLU	A	374	-16.063	18.277	131.424	1.00	0.73	C
ATOM	2983	CD	GLU	A	374	-15.367	19.553	131.969	1.00	0.73	C
ATOM	2984	OE1	GLU	A	374	-15.410	20.616	131.306	1.00	0.73	O
ATOM	2985	OE2	GLU	A	374	-14.718	19.490	133.053	1.00	0.73	O
ATOM	2986	N	LYS	A	375	-15.438	16.723	127.905	1.00	0.74	N
ATOM	2987	CA	LYS	A	375	-14.358	16.016	127.142	1.00	0.74	C
ATOM	2988	C	LYS	A	375	-13.251	16.735	126.309	1.00	0.74	C
ATOM	2989	O	LYS	A	375	-12.095	16.348	126.351	1.00	0.74	O
ATOM	2990	CB	LYS	A	375	-14.918	14.897	126.217	1.00	0.74	C
ATOM	2991	CG	LYS	A	375	-15.137	13.515	126.837	1.00	0.74	C
ATOM	2992	CD	LYS	A	375	-15.408	12.480	125.719	1.00	0.74	C
ATOM	2993	CE	LYS	A	375	-14.325	12.301	124.637	1.00	0.74	C
ATOM	2994	NZ	LYS	A	375	-13.007	12.105	125.274	1.00	0.74	N
ATOM	2995	N	LEU	A	376	-13.641	17.720	125.479	1.00	0.75	N
ATOM	2996	CA	LEU	A	376	-12.804	18.661	124.748	1.00	0.75	C
ATOM	2997	C	LEU	A	376	-11.951	19.633	125.588	1.00	0.75	C
ATOM	2998	O	LEU	A	376	-10.811	19.924	125.242	1.00	0.75	O
ATOM	2999	CB	LEU	A	376	-13.705	19.522	123.831	1.00	0.75	C
ATOM	3000	CG	LEU	A	376	-14.445	18.778	122.705	1.00	0.75	C
ATOM	3001	CD1	LEU	A	376	-15.393	19.752	121.990	1.00	0.75	C
ATOM	3002	CD2	LEU	A	376	-13.450	18.161	121.714	1.00	0.75	C
ATOM	3003	N	ARG	A	377	-12.490	20.196	126.688	1.00	0.66	N
ATOM	3004	CA	ARG	A	377	-11.809	21.118	127.591	1.00	0.66	C
ATOM	3005	C	ARG	A	377	-10.594	20.550	128.391	1.00	0.66	C
ATOM	3006	O	ARG	A	377	-9.552	21.216	128.363	1.00	0.66	O
ATOM	3007	CB	ARG	A	377	-12.901	21.814	128.450	1.00	0.66	C
ATOM	3008	CG	ARG	A	377	-12.371	22.892	129.409	1.00	0.66	C
ATOM	3009	CD	ARG	A	377	-13.429	23.479	130.354	1.00	0.66	C
ATOM	3010	NE	ARG	A	377	-13.621	22.530	131.496	1.00	0.66	N
ATOM	3011	CZ	ARG	A	377	-12.845	22.457	132.584	1.00	0.66	C
ATOM	3012	NH1	ARG	A	377	-13.109	21.547	133.517	1.00	0.66	N
ATOM	3013	NH2	ARG	A	377	-11.787	23.257	132.717	1.00	0.66	N
ATOM	3014	N	PRO	A	378	-10.544	19.377	129.046	1.00	0.74	N
ATOM	3015	CA	PRO	A	378	-9.334	18.621	129.411	1.00	0.74	C
ATOM	3016	C	PRO	A	378	-8.291	18.378	128.301	1.00	0.74	C
ATOM	3017	O	PRO	A	378	-7.120	18.219	128.651	1.00	0.74	O
ATOM	3018	CB	PRO	A	378	-9.900	17.277	129.944	1.00	0.74	C
ATOM	3019	CG	PRO	A	378	-11.328	17.555	130.406	1.00	0.74	C
ATOM	3020	CD	PRO	A	378	-11.725	18.626	129.425	1.00	0.74	C
ATOM	3021	N	LEU	A	379	-8.638	18.317	126.986	1.00	0.76	N
ATOM	3022	CA	LEU	A	379	-7.672	18.050	125.901	1.00	0.76	C
ATOM	3023	C	LEU	A	379	-6.630	19.160	125.743	1.00	0.76	C
ATOM	3024	O	LEU	A	379	-5.480	18.920	125.367	1.00	0.76	O
ATOM	3025	CB	LEU	A	379	-8.270	17.765	124.484	1.00	0.76	C
ATOM	3026	CG	LEU	A	379	-8.763	16.334	124.126	1.00	0.76	C
ATOM	3027	CD1	LEU	A	379	-8.405	16.014	122.657	1.00	0.76	C
ATOM	3028	CD2	LEU	A	379	-8.214	15.215	125.023	1.00	0.76	C
ATOM	3029	N	VAL	A	380	-7.012	20.417	126.049	1.00	0.77	N
ATOM	3030	CA	VAL	A	380	-6.129	21.570	126.074	1.00	0.77	C
ATOM	3031	C	VAL	A	380	-4.972	21.378	127.058	1.00	0.77	C
ATOM	3032	O	VAL	A	380	-3.824	21.755	126.812	1.00	0.77	O
ATOM	3033	CB	VAL	A	380	-6.896	22.829	126.475	1.00	0.77	C
ATOM	3034	CG1	VAL	A	380	-6.096	24.065	126.028	1.00	0.77	C
ATOM	3035	CG2	VAL	A	380	-8.316	22.866	125.867	1.00	0.77	C
ATOM	3036	N	GLU	A	381	-5.276	20.749	128.210	1.00	0.68	N
ATOM	3037	CA	GLU	A	381	-4.448	20.813	129.389	1.00	0.68	C
ATOM	3038	C	GLU	A	381	-3.519	19.609	129.562	1.00	0.68	C
ATOM	3039	O	GLU	A	381	-2.586	19.658	130.363	1.00	0.68	O
ATOM	3040	CB	GLU	A	381	-5.381	20.963	130.619	1.00	0.68	C
ATOM	3041	CG	GLU	A	381	-6.383	22.150	130.565	1.00	0.68	C
ATOM	3042	CD	GLU	A	381	-5.724	23.505	130.663	1.00	0.68	C
ATOM	3043	OE1	GLU	A	381	-5.797	24.285	129.679	1.00	0.68	O
ATOM	3044	OE2	GLU	A	381	-5.121	23.846	131.713	1.00	0.68	O
ATOM	3045	N	GLU	A	382	-3.688	18.533	128.760	1.00	0.67	N
ATOM	3046	CA	GLU	A	382	-2.807	17.366	128.724	1.00	0.67	C
ATOM	3047	C	GLU	A	382	-1.368	17.690	128.276	1.00	0.67	C
ATOM	3048	O	GLU	A	382	-0.405	17.164	128.841	1.00	0.67	O
ATOM	3049	CB	GLU	A	382	-3.461	16.224	127.904	1.00	0.67	C
ATOM	3050	CG	GLU	A	382	-3.337	14.786	128.468	1.00	0.67	C
ATOM	3051	CD	GLU	A	382	-3.735	13.778	127.381	1.00	0.67	C

ATOM	3052	OE1	GLU	A	382	-4.705	14.061	126.629	1.00	0.67	O
ATOM	3053	OE2	GLU	A	382	-3.035	12.740	127.265	1.00	0.67	O
ATOM	3054	N	PRO	A	383	-1.144	18.569	127.294	1.00	0.72	N
ATOM	3055	CA	PRO	A	383	0.159	19.181	127.057	1.00	0.72	C
ATOM	3056	C	PRO	A	383	0.861	19.981	128.148	1.00	0.72	C
ATOM	3057	O	PRO	A	383	2.087	19.929	128.192	1.00	0.72	O
ATOM	3058	CB	PRO	A	383	-0.140	20.137	125.903	1.00	0.72	C
ATOM	3059	CG	PRO	A	383	-1.171	19.408	125.043	1.00	0.72	C
ATOM	3060	CD	PRO	A	383	-1.948	18.561	126.054	1.00	0.72	C
ATOM	3061	N	LYS	A	384	0.172	20.781	128.989	1.00	0.67	N
ATOM	3062	CA	LYS	A	384	0.769	21.887	129.736	1.00	0.67	C
ATOM	3063	C	LYS	A	384	1.931	21.561	130.677	1.00	0.67	C
ATOM	3064	O	LYS	A	384	2.862	22.345	130.864	1.00	0.67	O
ATOM	3065	CB	LYS	A	384	-0.306	22.619	130.544	1.00	0.67	C
ATOM	3066	CG	LYS	A	384	-0.980	23.717	129.719	1.00	0.67	C
ATOM	3067	CD	LYS	A	384	-2.068	24.388	130.555	1.00	0.67	C
ATOM	3068	CE	LYS	A	384	-2.822	25.488	129.817	1.00	0.67	C
ATOM	3069	NZ	LYS	A	384	-3.942	25.948	130.652	1.00	0.67	N
ATOM	3070	N	ASN	A	385	1.884	20.382	131.309	1.00	0.69	N
ATOM	3071	CA	ASN	A	385	2.893	19.887	132.235	1.00	0.69	C
ATOM	3072	C	ASN	A	385	4.156	19.372	131.537	1.00	0.69	C
ATOM	3073	O	ASN	A	385	5.222	19.357	132.152	1.00	0.69	O
ATOM	3074	CB	ASN	A	385	2.362	18.855	133.295	1.00	0.69	C
ATOM	3075	CG	ASN	A	385	0.927	18.393	133.038	1.00	0.69	C
ATOM	3076	OD1	ASN	A	385	0.624	17.965	131.928	1.00	0.69	O
ATOM	3077	ND2	ASN	A	385	0.050	18.450	134.071	1.00	0.69	N
ATOM	3078	N	LEU	A	386	4.092	18.989	130.239	1.00	0.78	N
ATOM	3079	CA	LEU	A	386	5.245	18.664	129.403	1.00	0.78	C
ATOM	3080	C	LEU	A	386	6.128	19.875	129.206	1.00	0.78	C
ATOM	3081	O	LEU	A	386	7.354	19.804	129.208	1.00	0.78	O
ATOM	3082	CB	LEU	A	386	4.779	18.193	127.994	1.00	0.78	C
ATOM	3083	CG	LEU	A	386	5.839	17.875	126.903	1.00	0.78	C
ATOM	3084	CD1	LEU	A	386	5.215	16.991	125.816	1.00	0.78	C
ATOM	3085	CD2	LEU	A	386	6.378	19.113	126.157	1.00	0.78	C
ATOM	3086	N	ILE	A	387	5.458	21.027	128.992	1.00	0.79	N
ATOM	3087	CA	ILE	A	387	6.043	22.316	128.659	1.00	0.79	C
ATOM	3088	C	ILE	A	387	6.988	22.836	129.721	1.00	0.79	C
ATOM	3089	O	ILE	A	387	8.123	23.224	129.444	1.00	0.79	O
ATOM	3090	CB	ILE	A	387	4.961	23.380	128.503	1.00	0.79	C
ATOM	3091	CG1	ILE	A	387	3.753	22.901	127.666	1.00	0.79	C
ATOM	3092	CG2	ILE	A	387	5.582	24.669	127.927	1.00	0.79	C
ATOM	3093	CD1	ILE	A	387	4.077	22.498	126.228	1.00	0.79	C
ATOM	3094	N	LYS	A	388	6.525	22.823	130.987	1.00	0.75	N
ATOM	3095	CA	LYS	A	388	7.257	23.342	132.128	1.00	0.75	C
ATOM	3096	C	LYS	A	388	8.579	22.631	132.341	1.00	0.75	C
ATOM	3097	O	LYS	A	388	9.627	23.262	132.443	1.00	0.75	O
ATOM	3098	CB	LYS	A	388	6.368	23.215	133.397	1.00	0.75	C
ATOM	3099	CG	LYS	A	388	7.112	23.294	134.743	1.00	0.75	C
ATOM	3100	CD	LYS	A	388	6.188	23.271	135.969	1.00	0.75	C
ATOM	3101	CE	LYS	A	388	5.945	24.673	136.521	1.00	0.75	C
ATOM	3102	NZ	LYS	A	388	4.789	24.648	137.439	1.00	0.75	N
ATOM	3103	N	GLN	A	389	8.548	21.287	132.318	1.00	0.77	N
ATOM	3104	CA	GLN	A	389	9.685	20.430	132.557	1.00	0.77	C
ATOM	3105	C	GLN	A	389	10.768	20.618	131.520	1.00	0.77	C
ATOM	3106	O	GLN	A	389	11.954	20.718	131.819	1.00	0.77	O
ATOM	3107	CB	GLN	A	389	9.185	18.973	132.478	1.00	0.77	C
ATOM	3108	CG	GLN	A	389	8.165	18.607	133.577	1.00	0.77	C
ATOM	3109	CD	GLN	A	389	7.600	17.205	133.345	1.00	0.77	C
ATOM	3110	OE1	GLN	A	389	8.266	16.291	132.862	1.00	0.77	O
ATOM	3111	NE2	GLN	A	389	6.300	17.028	133.684	1.00	0.77	N
ATOM	3112	N	ASN	A	390	10.347	20.692	130.243	1.00	0.83	N
ATOM	3113	CA	ASN	A	390	11.233	20.925	129.121	1.00	0.83	C
ATOM	3114	C	ASN	A	390	11.859	22.265	129.081	1.00	0.83	C
ATOM	3115	O	ASN	A	390	13.050	22.379	128.794	1.00	0.83	O
ATOM	3116	CB	ASN	A	390	10.531	20.692	127.781	1.00	0.83	C
ATOM	3117	CG	ASN	A	390	10.458	19.187	127.676	1.00	0.83	C
ATOM	3118	OD1	ASN	A	390	11.474	18.495	127.797	1.00	0.83	O
ATOM	3119	ND2	ASN	A	390	9.270	18.600	127.507	1.00	0.83	N
ATOM	3120	N	CYS	A	391	11.080	23.314	129.370	1.00	0.88	N
ATOM	3121	CA	CYS	A	391	11.660	24.623	129.493	1.00	0.88	C
ATOM	3122	C	CYS	A	391	12.629	24.685	130.675	1.00	0.88	C
ATOM	3123	O	CYS	A	391	13.757	25.106	130.504	1.00	0.88	O
ATOM	3124	CB	CYS	A	391	10.625	25.772	129.521	1.00	0.88	C
ATOM	3125	SG	CYS	A	391	9.755	26.010	127.938	1.00	0.88	S
ATOM	3126	N	GLU	A	392	12.278	24.157	131.874	1.00	0.80	N
ATOM	3127	CA	GLU	A	392	13.210	24.155	133.006	1.00	0.80	C

ATOM	3128	C	GLU	A	392	14.537	23.455	132.720	1.00	0.80	C
ATOM	3129	O	GLU	A	392	15.615	23.983	132.989	1.00	0.80	O
ATOM	3130	CB	GLU	A	392	12.588	23.506	134.262	1.00	0.80	C
ATOM	3131	CG	GLU	A	392	11.456	24.354	134.898	1.00	0.80	C
ATOM	3132	CD	GLU	A	392	10.675	23.634	136.003	1.00	0.80	C
ATOM	3133	OE1	GLU	A	392	11.052	22.490	136.358	1.00	0.80	O
ATOM	3134	OE2	GLU	A	392	9.664	24.221	136.482	1.00	0.80	O
ATOM	3135	N	LEU	A	393	14.483	22.270	132.083	1.00	0.85	N
ATOM	3136	CA	LEU	A	393	15.642	21.566	131.575	1.00	0.85	C
ATOM	3137	C	LEU	A	393	16.446	22.350	130.532	1.00	0.85	C
ATOM	3138	O	LEU	A	393	17.675	22.330	130.561	1.00	0.85	O
ATOM	3139	CB	LEU	A	393	15.246	20.203	130.958	1.00	0.85	C
ATOM	3140	CG	LEU	A	393	14.748	19.106	131.925	1.00	0.85	C
ATOM	3141	CD1	LEU	A	393	14.063	17.985	131.120	1.00	0.85	C
ATOM	3142	CD2	LEU	A	393	15.858	18.522	132.815	1.00	0.85	C
ATOM	3143	N	PHE	A	394	15.779	23.063	129.599	1.00	0.88	N
ATOM	3144	CA	PHE	A	394	16.396	24.007	128.682	1.00	0.88	C
ATOM	3145	C	PHE	A	394	17.072	25.209	129.366	1.00	0.88	C
ATOM	3146	O	PHE	A	394	18.238	25.474	129.071	1.00	0.88	O
ATOM	3147	CB	PHE	A	394	15.342	24.463	127.628	1.00	0.88	C
ATOM	3148	CG	PHE	A	394	15.843	25.567	126.744	1.00	0.88	C
ATOM	3149	CD1	PHE	A	394	16.706	25.304	125.675	1.00	0.88	C
ATOM	3150	CD2	PHE	A	394	15.543	26.899	127.066	1.00	0.88	C
ATOM	3151	CE1	PHE	A	394	17.251	26.353	124.927	1.00	0.88	C
ATOM	3152	CE2	PHE	A	394	16.109	27.948	126.338	1.00	0.88	C
ATOM	3153	CZ	PHE	A	394	16.957	27.679	125.261	1.00	0.88	C
ATOM	3154	N	GLU	A	395	16.418	25.938	130.309	1.00	0.82	N
ATOM	3155	CA	GLU	A	395	17.029	27.100	130.970	1.00	0.82	C
ATOM	3156	C	GLU	A	395	18.270	26.688	131.748	1.00	0.82	C
ATOM	3157	O	GLU	A	395	19.297	27.364	131.794	1.00	0.82	O
ATOM	3158	CB	GLU	A	395	16.146	27.887	131.997	1.00	0.82	C
ATOM	3159	CG	GLU	A	395	14.614	28.061	131.789	1.00	0.82	C
ATOM	3160	CD	GLU	A	395	14.126	28.756	130.521	1.00	0.82	C
ATOM	3161	OE1	GLU	A	395	14.963	29.300	129.758	1.00	0.82	O
ATOM	3162	OE2	GLU	A	395	12.878	28.742	130.323	1.00	0.82	O
ATOM	3163	N	LYS	A	396	18.153	25.520	132.397	1.00	0.79	N
ATOM	3164	CA	LYS	A	396	19.165	24.882	133.206	1.00	0.79	C
ATOM	3165	C	LYS	A	396	20.439	24.449	132.500	1.00	0.79	C
ATOM	3166	O	LYS	A	396	21.528	24.501	133.065	1.00	0.79	O
ATOM	3167	CB	LYS	A	396	18.580	23.592	133.813	1.00	0.79	C
ATOM	3168	CG	LYS	A	396	19.339	23.099	135.045	1.00	0.79	C
ATOM	3169	CD	LYS	A	396	18.882	21.695	135.475	1.00	0.79	C
ATOM	3170	CE	LYS	A	396	19.811	21.003	136.469	1.00	0.79	C
ATOM	3171	NZ	LYS	A	396	21.110	20.810	135.797	1.00	0.79	N
ATOM	3172	N	LEU	A	397	20.279	23.896	131.284	1.00	0.85	N
ATOM	3173	CA	LEU	A	397	21.361	23.354	130.495	1.00	0.85	C
ATOM	3174	C	LEU	A	397	21.812	24.291	129.361	1.00	0.85	C
ATOM	3175	O	LEU	A	397	22.995	24.323	129.022	1.00	0.85	O
ATOM	3176	CB	LEU	A	397	20.886	21.985	129.942	1.00	0.85	C
ATOM	3177	CG	LEU	A	397	20.784	20.837	130.974	1.00	0.85	C
ATOM	3178	CD1	LEU	A	397	19.955	19.696	130.370	1.00	0.85	C
ATOM	3179	CD2	LEU	A	397	22.166	20.297	131.356	1.00	0.85	C
ATOM	3180	N	GLY	A	398	20.929	25.123	128.760	1.00	0.89	N
ATOM	3181	CA	GLY	A	398	21.197	25.855	127.520	1.00	0.89	C
ATOM	3182	C	GLY	A	398	21.055	24.982	126.295	1.00	0.89	C
ATOM	3183	O	GLY	A	398	20.996	23.757	126.374	1.00	0.89	O
ATOM	3184	N	GLU	A	399	21.009	25.599	125.094	1.00	0.85	N
ATOM	3185	CA	GLU	A	399	20.634	24.925	123.857	1.00	0.85	C
ATOM	3186	C	GLU	A	399	21.430	23.665	123.489	1.00	0.85	C
ATOM	3187	O	GLU	A	399	20.854	22.599	123.287	1.00	0.85	O
ATOM	3188	CB	GLU	A	399	20.632	25.968	122.704	1.00	0.85	C
ATOM	3189	CG	GLU	A	399	20.302	25.397	121.294	1.00	0.85	C
ATOM	3190	CD	GLU	A	399	20.066	26.469	120.210	1.00	0.85	C
ATOM	3191	OE1	GLU	A	399	19.309	27.431	120.504	1.00	0.85	O
ATOM	3192	OE2	GLU	A	399	20.586	26.297	119.071	1.00	0.85	O
ATOM	3193	N	TYR	A	400	22.777	23.715	123.469	1.00	0.85	N
ATOM	3194	CA	TYR	A	400	23.619	22.580	123.110	1.00	0.85	C
ATOM	3195	C	TYR	A	400	23.477	21.375	124.035	1.00	0.85	C
ATOM	3196	O	TYR	A	400	23.403	20.223	123.608	1.00	0.85	O
ATOM	3197	CB	TYR	A	400	25.098	23.045	123.092	1.00	0.85	C
ATOM	3198	CG	TYR	A	400	26.082	21.979	122.681	1.00	0.85	C
ATOM	3199	CD1	TYR	A	400	25.836	21.128	121.592	1.00	0.85	C
ATOM	3200	CD2	TYR	A	400	27.282	21.829	123.394	1.00	0.85	C
ATOM	3201	CE1	TYR	A	400	26.783	20.169	121.212	1.00	0.85	C
ATOM	3202	CE2	TYR	A	400	28.236	20.884	122.995	1.00	0.85	C
ATOM	3203	CZ	TYR	A	400	27.992	20.064	121.892	1.00	0.85	C

```
ATOM   3204  OH  TYR A 400      28.970  19.149 121.460  1.00  0.85           O
ATOM   3205  N   GLN A 401      23.426  21.644 125.349  1.00  0.81           N
ATOM   3206  CA  GLN A 401      23.298  20.654 126.382  1.00  0.81           C
ATOM   3207  C   GLN A 401      21.887  20.065 126.431  1.00  0.81           C
ATOM   3208  O   GLN A 401      21.693  18.903 126.794  1.00  0.81           O
ATOM   3209  CB  GLN A 401      23.614  21.340 127.729  1.00  0.81           C
ATOM   3210  CG  GLN A 401      25.010  21.955 127.978  1.00  0.81           C
ATOM   3211  CD  GLN A 401      26.062  20.863 128.006  1.00  0.81           C
ATOM   3212  OE1 GLN A 401      25.940  19.868 128.720  1.00  0.81           O
ATOM   3213  NE2 GLN A 401      27.136  21.038 127.205  1.00  0.81           N
ATOM   3214  N   PHE A 402      20.868  20.863 126.035  1.00  0.88           N
ATOM   3215  CA  PHE A 402      19.501  20.428 125.810  1.00  0.88           C
ATOM   3216  C   PHE A 402      19.331  19.537 124.591  1.00  0.88           C
ATOM   3217  O   PHE A 402      18.558  18.581 124.619  1.00  0.88           O
ATOM   3218  CB  PHE A 402      18.539  21.639 125.755  1.00  0.88           C
ATOM   3219  CG  PHE A 402      17.091  21.243 125.873  1.00  0.88           C
ATOM   3220  CD1 PHE A 402      16.629  20.500 126.969  1.00  0.88           C
ATOM   3221  CD2 PHE A 402      16.170  21.624 124.888  1.00  0.88           C
ATOM   3222  CE1 PHE A 402      15.283  20.131 127.069  1.00  0.88           C
ATOM   3223  CE2 PHE A 402      14.823  21.251 124.982  1.00  0.88           C
ATOM   3224  CZ  PHE A 402      14.376  20.500 126.073  1.00  0.88           C
ATOM   3225  N   GLN A 403      20.069  19.788 123.488  1.00  0.86           N
ATOM   3226  CA  GLN A 403      20.092  18.883 122.352  1.00  0.86           C
ATOM   3227  C   GLN A 403      20.644  17.531 122.747  1.00  0.86           C
ATOM   3228  O   GLN A 403      20.042  16.497 122.482  1.00  0.86           O
ATOM   3229  CB  GLN A 403      20.946  19.466 121.209  1.00  0.86           C
ATOM   3230  CG  GLN A 403      20.324  20.734 120.595  1.00  0.86           C
ATOM   3231  CD  GLN A 403      21.242  21.379 119.560  1.00  0.86           C
ATOM   3232  OE1 GLN A 403      22.444  21.109 119.487  1.00  0.86           O
ATOM   3233  NE2 GLN A 403      20.651  22.253 118.710  1.00  0.86           N
ATOM   3234  N   ASN A 404      21.768  17.522 123.486  1.00  0.88           N
ATOM   3235  CA  ASN A 404      22.374  16.304 124.001  1.00  0.88           C
ATOM   3236  C   ASN A 404      21.444  15.525 124.962  1.00  0.88           C
ATOM   3237  O   ASN A 404      21.417  14.294 124.973  1.00  0.88           O
ATOM   3238  CB  ASN A 404      23.741  16.575 124.674  1.00  0.88           C
ATOM   3239  CG  ASN A 404      24.712  17.269 123.719  1.00  0.88           C
ATOM   3240  OD1 ASN A 404      24.564  17.288 122.495  1.00  0.88           O
ATOM   3241  ND2 ASN A 404      25.775  17.868 124.305  1.00  0.88           N
ATOM   3242  N   ALA A 405      20.641  16.260 125.773  1.00  0.91           N
ATOM   3243  CA  ALA A 405      19.574  15.754 126.626  1.00  0.91           C
ATOM   3244  C   ALA A 405      18.454  15.082 125.838  1.00  0.91           C
ATOM   3245  O   ALA A 405      17.979  14.001 126.196  1.00  0.91           O
ATOM   3246  CB  ALA A 405      18.960  16.894 127.485  1.00  0.91           C
ATOM   3247  N   LEU A 406      18.010  15.709 124.729  1.00  0.89           N
ATOM   3248  CA  LEU A 406      17.071  15.131 123.778  1.00  0.89           C
ATOM   3249  C   LEU A 406      17.593  13.953 122.977  1.00  0.89           C
ATOM   3250  O   LEU A 406      16.872  12.974 122.807  1.00  0.89           O
ATOM   3251  CB  LEU A 406      16.483  16.202 122.851  1.00  0.89           C
ATOM   3252  CG  LEU A 406      15.498  17.122 123.588  1.00  0.89           C
ATOM   3253  CD1 LEU A 406      15.384  18.464 122.866  1.00  0.89           C
ATOM   3254  CD2 LEU A 406      14.127  16.443 123.732  1.00  0.89           C
ATOM   3255  N   ILE A 407      18.871  13.978 122.532  1.00  0.90           N
ATOM   3256  CA  ILE A 407      19.591  12.852 121.938  1.00  0.90           C
ATOM   3257  C   ILE A 407      19.475  11.638 122.826  1.00  0.90           C
ATOM   3258  O   ILE A 407      19.015  10.590 122.399  1.00  0.90           O
ATOM   3259  CB  ILE A 407      21.071  13.177 121.656  1.00  0.90           C
ATOM   3260  CG1 ILE A 407      21.147  14.146 120.457  1.00  0.90           C
ATOM   3261  CG2 ILE A 407      21.927  11.909 121.426  1.00  0.90           C
ATOM   3262  CD1 ILE A 407      22.464  14.214 119.690  1.00  0.90           C
ATOM   3263  N   VAL A 408      19.796  11.785 124.126  1.00  0.89           N
ATOM   3264  CA  VAL A 408      19.707  10.676 125.063  1.00  0.89           C
ATOM   3265  C   VAL A 408      18.291  10.161 125.228  1.00  0.89           C
ATOM   3266  O   VAL A 408      18.025   8.956 125.164  1.00  0.89           O
ATOM   3267  CB  VAL A 408      20.283  11.088 126.410  1.00  0.89           C
ATOM   3268  CG1 VAL A 408      19.904  10.121 127.555  1.00  0.89           C
ATOM   3269  CG2 VAL A 408      21.809  11.147 126.237  1.00  0.89           C
ATOM   3270  N   ARG A 409      17.336  11.085 125.416  1.00  0.79           N
ATOM   3271  CA  ARG A 409      15.935  10.773 125.604  1.00  0.79           C
ATOM   3272  C   ARG A 409      15.263  10.121 124.398  1.00  0.79           C
ATOM   3273  O   ARG A 409      14.480   9.184 124.548  1.00  0.79           O
ATOM   3274  CB  ARG A 409      15.193  12.051 126.078  1.00  0.79           C
ATOM   3275  CG  ARG A 409      13.672  12.107 125.819  1.00  0.79           C
ATOM   3276  CD  ARG A 409      12.825  12.616 126.988  1.00  0.79           C
ATOM   3277  NE  ARG A 409      12.396  14.020 126.713  1.00  0.79           N
ATOM   3278  CZ  ARG A 409      13.082  15.124 127.044  1.00  0.79           C
ATOM   3279  NH1 ARG A 409      14.306  15.109 127.558  1.00  0.79           N
```

ATOM	3280	NH2	ARG	A	409	12.498	16.284	126.795	1.00	0.79	N
ATOM	3281	N	TYR	A	410	15.543	10.616	123.182	1.00	0.86	N
ATOM	3282	CA	TYR	A	410	15.043	10.078	121.936	1.00	0.86	C
ATOM	3283	C	TYR	A	410	15.751	8.810	121.445	1.00	0.86	C
ATOM	3284	O	TYR	A	410	15.109	7.950	120.841	1.00	0.86	O
ATOM	3285	CB	TYR	A	410	14.991	11.180	120.846	1.00	0.86	C
ATOM	3286	CG	TYR	A	410	13.606	11.735	120.793	1.00	0.86	C
ATOM	3287	CD1	TYR	A	410	13.163	12.725	121.684	1.00	0.86	C
ATOM	3288	CD2	TYR	A	410	12.701	11.169	119.890	1.00	0.86	C
ATOM	3289	CE1	TYR	A	410	11.823	13.134	121.661	1.00	0.86	C
ATOM	3290	CE2	TYR	A	410	11.365	11.586	119.863	1.00	0.86	C
ATOM	3291	CZ	TYR	A	410	10.924	12.570	120.755	1.00	0.86	C
ATOM	3292	OH	TYR	A	410	9.593	13.032	120.758	1.00	0.86	O
ATOM	3293	N	THR	A	411	17.066	8.624	121.710	1.00	0.89	N
ATOM	3294	CA	THR	A	411	17.796	7.390	121.354	1.00	0.89	C
ATOM	3295	C	THR	A	411	17.339	6.200	122.173	1.00	0.89	C
ATOM	3296	O	THR	A	411	17.170	5.108	121.643	1.00	0.89	O
ATOM	3297	CB	THR	A	411	19.340	7.473	121.337	1.00	0.89	C
ATOM	3298	OG1	THR	A	411	19.796	8.394	120.357	1.00	0.89	O
ATOM	3299	CG2	THR	A	411	20.021	6.135	120.964	1.00	0.89	C
ATOM	3300	N	LYS	A	412	17.075	6.365	123.491	1.00	0.84	N
ATOM	3301	CA	LYS	A	412	16.457	5.317	124.287	1.00	0.84	C
ATOM	3302	C	LYS	A	412	15.070	4.938	123.764	1.00	0.84	C
ATOM	3303	O	LYS	A	412	14.721	3.763	123.683	1.00	0.84	O
ATOM	3304	CB	LYS	A	412	16.368	5.737	125.776	1.00	0.84	C
ATOM	3305	CG	LYS	A	412	17.719	5.840	126.501	1.00	0.84	C
ATOM	3306	CD	LYS	A	412	17.523	6.206	127.983	1.00	0.84	C
ATOM	3307	CE	LYS	A	412	18.849	6.358	128.735	1.00	0.84	C
ATOM	3308	NZ	LYS	A	412	18.636	6.780	130.140	1.00	0.84	N
ATOM	3309	N	LYS	A	413	14.278	5.955	123.378	1.00	0.82	N
ATOM	3310	CA	LYS	A	413	12.933	5.844	122.837	1.00	0.82	C
ATOM	3311	C	LYS	A	413	12.801	5.088	121.531	1.00	0.82	C
ATOM	3312	O	LYS	A	413	11.911	4.252	121.360	1.00	0.82	O
ATOM	3313	CB	LYS	A	413	12.415	7.279	122.588	1.00	0.82	C
ATOM	3314	CG	LYS	A	413	11.452	7.847	123.635	1.00	0.82	C
ATOM	3315	CD	LYS	A	413	10.935	9.216	123.155	1.00	0.82	C
ATOM	3316	CE	LYS	A	413	10.265	10.080	124.224	1.00	0.82	C
ATOM	3317	NZ	LYS	A	413	8.917	9.574	124.526	1.00	0.82	N
ATOM	3318	N	VAL	A	414	13.672	5.385	120.552	1.00	0.88	N
ATOM	3319	CA	VAL	A	414	13.571	4.774	119.246	1.00	0.88	C
ATOM	3320	C	VAL	A	414	14.951	4.355	118.734	1.00	0.88	C
ATOM	3321	O	VAL	A	414	15.419	4.889	117.727	1.00	0.88	O
ATOM	3322	CB	VAL	A	414	12.775	5.647	118.256	1.00	0.88	C
ATOM	3323	CG1	VAL	A	414	11.269	5.528	118.589	1.00	0.88	C
ATOM	3324	CG2	VAL	A	414	13.240	7.119	118.278	1.00	0.88	C
ATOM	3325	N	PRO	A	415	15.653	3.387	119.344	1.00	0.88	N
ATOM	3326	CA	PRO	A	415	17.066	3.119	119.061	1.00	0.88	C
ATOM	3327	C	PRO	A	415	17.397	2.737	117.617	1.00	0.88	C
ATOM	3328	O	PRO	A	415	18.521	2.978	117.166	1.00	0.88	O
ATOM	3329	CB	PRO	A	415	17.430	1.958	120.003	1.00	0.88	C
ATOM	3330	CG	PRO	A	415	16.366	1.943	121.100	1.00	0.88	C
ATOM	3331	CD	PRO	A	415	15.133	2.507	120.401	1.00	0.88	C
ATOM	3332	N	GLN	A	416	16.443	2.090	116.900	1.00	0.83	N
ATOM	3333	CA	GLN	A	416	16.586	1.585	115.538	1.00	0.83	C
ATOM	3334	C	GLN	A	416	16.778	2.653	114.473	1.00	0.83	C
ATOM	3335	O	GLN	A	416	17.517	2.446	113.510	1.00	0.83	O
ATOM	3336	CB	GLN	A	416	15.427	0.661	115.084	1.00	0.83	C
ATOM	3337	CG	GLN	A	416	15.180	-0.553	116.001	1.00	0.83	C
ATOM	3338	CD	GLN	A	416	14.192	-0.286	117.141	1.00	0.83	C
ATOM	3339	OE1	GLN	A	416	13.607	0.788	117.323	1.00	0.83	O
ATOM	3340	NE2	GLN	A	416	14.023	-1.341	117.981	1.00	0.83	N
ATOM	3341	N	VAL	A	417	16.105	3.825	114.627	1.00	0.87	N
ATOM	3342	CA	VAL	A	417	16.218	5.013	113.772	1.00	0.87	C
ATOM	3343	C	VAL	A	417	17.672	5.297	113.456	1.00	0.87	C
ATOM	3344	O	VAL	A	417	18.534	5.238	114.331	1.00	0.87	O
ATOM	3345	CB	VAL	A	417	15.600	6.264	114.399	1.00	0.87	C
ATOM	3346	CG1	VAL	A	417	15.788	7.529	113.532	1.00	0.87	C
ATOM	3347	CG2	VAL	A	417	14.102	6.041	114.642	1.00	0.87	C
ATOM	3348	N	SER	A	418	18.023	5.564	112.184	1.00	0.87	N
ATOM	3349	CA	SER	A	418	19.414	5.771	111.811	1.00	0.87	C
ATOM	3350	C	SER	A	418	20.057	6.929	112.585	1.00	0.87	C
ATOM	3351	O	SER	A	418	19.398	7.874	113.018	1.00	0.87	O
ATOM	3352	CB	SER	A	418	19.650	5.933	110.278	1.00	0.87	C
ATOM	3353	OG	SER	A	418	19.096	7.147	109.771	1.00	0.87	O
ATOM	3354	N	THR	A	419	21.378	6.848	112.836	1.00	0.89	N
ATOM	3355	CA	THR	A	419	22.133	7.877	113.563	1.00	0.89	C

ATOM	3356	C	THR	A	419	22.048	9.274	112.966	1.00	0.89	C
ATOM	3357	O	THR	A	419	21.780	10.191	113.743	1.00	0.89	O
ATOM	3358	CB	THR	A	419	23.580	7.463	113.796	1.00	0.89	C
ATOM	3359	OG1	THR	A	419	23.557	6.249	114.537	1.00	0.89	O
ATOM	3360	CG2	THR	A	419	24.410	8.519	114.559	1.00	0.89	C
ATOM	3361	N	PRO	A	420	22.171	9.544	111.654	1.00	0.88	N
ATOM	3362	CA	PRO	A	420	22.017	10.895	111.138	1.00	0.88	C
ATOM	3363	C	PRO	A	420	20.640	11.475	111.392	1.00	0.88	C
ATOM	3364	O	PRO	A	420	20.556	12.660	111.713	1.00	0.88	O
ATOM	3365	CB	PRO	A	420	22.387	10.847	109.644	1.00	0.88	C
ATOM	3366	CG	PRO	A	420	22.849	9.416	109.340	1.00	0.88	C
ATOM	3367	CD	PRO	A	420	22.622	8.611	110.622	1.00	0.88	C
ATOM	3368	N	THR	A	421	19.563	10.662	111.302	1.00	0.89	N
ATOM	3369	CA	THR	A	421	18.210	11.109	111.624	1.00	0.89	C
ATOM	3370	C	THR	A	421	18.075	11.494	113.084	1.00	0.89	C
ATOM	3371	O	THR	A	421	17.616	12.587	113.402	1.00	0.89	O
ATOM	3372	CB	THR	A	421	17.138	10.079	111.274	1.00	0.89	C
ATOM	3373	OG1	THR	A	421	17.121	9.838	109.875	1.00	0.89	O
ATOM	3374	CG2	THR	A	421	15.718	10.547	111.622	1.00	0.89	C
ATOM	3375	N	LEU	A	422	18.549	10.643	114.026	1.00	0.91	N
ATOM	3376	CA	LEU	A	422	18.474	10.931	115.451	1.00	0.91	C
ATOM	3377	C	LEU	A	422	19.230	12.189	115.846	1.00	0.91	C
ATOM	3378	O	LEU	A	422	18.759	12.985	116.662	1.00	0.91	O
ATOM	3379	CB	LEU	A	422	18.931	9.719	116.297	1.00	0.91	C
ATOM	3380	CG	LEU	A	422	17.839	8.652	116.498	1.00	0.91	C
ATOM	3381	CD1	LEU	A	422	18.459	7.344	116.998	1.00	0.91	C
ATOM	3382	CD2	LEU	A	422	16.762	9.102	117.498	1.00	0.91	C
ATOM	3383	N	VAL	A	423	20.411	12.432	115.244	1.00	0.93	N
ATOM	3384	CA	VAL	A	423	21.114	13.687	115.461	1.00	0.93	C
ATOM	3385	C	VAL	A	423	20.365	14.921	114.947	1.00	0.93	C
ATOM	3386	O	VAL	A	423	20.169	15.888	115.682	1.00	0.93	O
ATOM	3387	CB	VAL	A	423	22.488	13.697	114.811	1.00	0.93	C
ATOM	3388	CG1	VAL	A	423	23.202	15.051	115.018	1.00	0.93	C
ATOM	3389	CG2	VAL	A	423	23.353	12.596	115.435	1.00	0.93	C
ATOM	3390	N	GLU	A	424	19.903	14.920	113.673	1.00	0.85	N
ATOM	3391	CA	GLU	A	424	19.240	16.074	113.069	1.00	0.85	C
ATOM	3392	C	GLU	A	424	17.927	16.456	113.749	1.00	0.85	C
ATOM	3393	O	GLU	A	424	17.682	17.635	114.033	1.00	0.85	O
ATOM	3394	CB	GLU	A	424	19.070	15.913	111.527	1.00	0.85	C
ATOM	3395	CG	GLU	A	424	17.774	16.517	110.924	1.00	0.85	C
ATOM	3396	CD	GLU	A	424	17.765	16.633	109.401	1.00	0.85	C
ATOM	3397	OE1	GLU	A	424	18.246	15.735	108.674	1.00	0.85	O
ATOM	3398	OE2	GLU	A	424	17.149	17.625	108.923	1.00	0.85	O
ATOM	3399	N	VAL	A	425	17.066	15.466	114.083	1.00	0.91	N
ATOM	3400	CA	VAL	A	425	15.804	15.689	114.790	1.00	0.91	C
ATOM	3401	C	VAL	A	425	16.052	16.305	116.164	1.00	0.91	C
ATOM	3402	O	VAL	A	425	15.515	17.358	116.492	1.00	0.91	O
ATOM	3403	CB	VAL	A	425	14.976	14.403	114.905	1.00	0.91	C
ATOM	3404	CG1	VAL	A	425	13.659	14.624	115.683	1.00	0.91	C
ATOM	3405	CG2	VAL	A	425	14.623	13.903	113.492	1.00	0.91	C
ATOM	3406	N	SER	A	426	16.963	15.723	116.972	1.00	0.91	N
ATOM	3407	CA	SER	A	426	17.247	16.201	118.324	1.00	0.91	C
ATOM	3408	C	SER	A	426	17.795	17.602	118.401	1.00	0.91	C
ATOM	3409	O	SER	A	426	17.479	18.387	119.298	1.00	0.91	O
ATOM	3410	CB	SER	A	426	18.221	15.274	119.055	1.00	0.91	C
ATOM	3411	OG	SER	A	426	17.556	14.058	119.398	1.00	0.91	O
ATOM	3412	N	ARG	A	427	18.633	17.966	117.426	1.00	0.82	N
ATOM	3413	CA	ARG	A	427	19.121	19.313	117.271	1.00	0.82	C
ATOM	3414	C	ARG	A	427	18.029	20.334	116.972	1.00	0.82	C
ATOM	3415	O	ARG	A	427	17.986	21.419	117.553	1.00	0.82	O
ATOM	3416	CB	ARG	A	427	20.176	19.351	116.154	1.00	0.82	C
ATOM	3417	CG	ARG	A	427	21.505	18.671	116.553	1.00	0.82	C
ATOM	3418	CD	ARG	A	427	22.618	18.704	115.494	1.00	0.82	C
ATOM	3419	NE	ARG	A	427	22.815	20.125	115.052	1.00	0.82	N
ATOM	3420	CZ	ARG	A	427	23.599	21.039	115.639	1.00	0.82	C
ATOM	3421	NH1	ARG	A	427	24.371	20.775	116.680	1.00	0.82	N
ATOM	3422	NH2	ARG	A	427	23.636	22.279	115.136	1.00	0.82	N
ATOM	3423	N	ASN	A	428	17.107	19.978	116.060	1.00	0.87	N
ATOM	3424	CA	ASN	A	428	15.925	20.752	115.734	1.00	0.87	C
ATOM	3425	C	ASN	A	428	14.915	20.905	116.874	1.00	0.87	C
ATOM	3426	O	ASN	A	428	14.368	21.989	117.077	1.00	0.87	O
ATOM	3427	CB	ASN	A	428	15.208	20.121	114.528	1.00	0.87	C
ATOM	3428	CG	ASN	A	428	15.966	20.454	113.248	1.00	0.87	C
ATOM	3429	OD1	ASN	A	428	16.750	21.402	113.170	1.00	0.87	O
ATOM	3430	ND2	ASN	A	428	15.691	19.664	112.186	1.00	0.87	N
ATOM	3431	N	LEU	A	429	14.668	19.812	117.636	1.00	0.88	N

```
ATOM   3432  CA  LEU A 429      13.881  19.758 118.870  1.00  0.88           C
ATOM   3433  C   LEU A 429      14.497  20.623 119.960  1.00  0.88           C
ATOM   3434  O   LEU A 429      13.798  21.324 120.688  1.00  0.88           O
ATOM   3435  CB  LEU A 429      13.729  18.314 119.452  1.00  0.88           C
ATOM   3436  CG  LEU A 429      12.905  17.267 118.672  1.00  0.88           C
ATOM   3437  CD1 LEU A 429      12.938  15.911 119.405  1.00  0.88           C
ATOM   3438  CD2 LEU A 429      11.444  17.696 118.531  1.00  0.88           C
ATOM   3439  N   GLY A 430      15.840  20.624 120.103  1.00  0.91           N
ATOM   3440  CA  GLY A 430      16.499  21.479 121.087  1.00  0.91           C
ATOM   3441  C   GLY A 430      16.398  22.966 120.823  1.00  0.91           C
ATOM   3442  O   GLY A 430      16.282  23.764 121.752  1.00  0.91           O
ATOM   3443  N   ARG A 431      16.345  23.367 119.532  1.00  0.79           N
ATOM   3444  CA  ARG A 431      16.089  24.742 119.123  1.00  0.79           C
ATOM   3445  C   ARG A 431      14.657  25.165 119.388  1.00  0.79           C
ATOM   3446  O   ARG A 431      14.355  26.348 119.418  1.00  0.79           O
ATOM   3447  CB  ARG A 431      16.429  25.021 117.638  1.00  0.79           C
ATOM   3448  CG  ARG A 431      17.942  25.108 117.374  1.00  0.79           C
ATOM   3449  CD  ARG A 431      18.308  25.409 115.924  1.00  0.79           C
ATOM   3450  NE  ARG A 431      19.804  25.411 115.894  1.00  0.79           N
ATOM   3451  CZ  ARG A 431      20.489  25.446 114.746  1.00  0.79           C
ATOM   3452  NH1 ARG A 431      21.819  25.534 114.775  1.00  0.79           N
ATOM   3453  NH2 ARG A 431      19.849  25.513 113.581  1.00  0.79           N
ATOM   3454  N   VAL A 432      13.717  24.232 119.659  1.00  0.88           N
ATOM   3455  CA  VAL A 432      12.387  24.589 120.141  1.00  0.88           C
ATOM   3456  C   VAL A 432      12.458  25.252 121.520  1.00  0.88           C
ATOM   3457  O   VAL A 432      11.624  26.094 121.853  1.00  0.88           O
ATOM   3458  CB  VAL A 432      11.385  23.429 120.082  1.00  0.88           C
ATOM   3459  CG1 VAL A 432       9.964  23.831 120.541  1.00  0.88           C
ATOM   3460  CG2 VAL A 432      11.320  22.879 118.639  1.00  0.88           C
ATOM   3461  N   GLY A 433      13.509  24.964 122.332  1.00  0.90           N
ATOM   3462  CA  GLY A 433      13.756  25.683 123.577  1.00  0.90           C
ATOM   3463  C   GLY A 433      14.032  27.149 123.370  1.00  0.90           C
ATOM   3464  O   GLY A 433      13.409  27.997 124.004  1.00  0.90           O
ATOM   3465  N   SER A 434      14.941  27.488 122.432  1.00  0.87           N
ATOM   3466  CA  SER A 434      15.235  28.869 122.063  1.00  0.87           C
ATOM   3467  C   SER A 434      14.047  29.602 121.453  1.00  0.87           C
ATOM   3468  O   SER A 434      13.771  30.751 121.796  1.00  0.87           O
ATOM   3469  CB  SER A 434      16.498  29.007 121.159  1.00  0.87           C
ATOM   3470  OG  SER A 434      16.322  28.541 119.821  1.00  0.87           O
ATOM   3471  N   LYS A 435      13.298  28.918 120.561  1.00  0.86           N
ATOM   3472  CA  LYS A 435      12.088  29.421 119.929  1.00  0.86           C
ATOM   3473  C   LYS A 435      10.945  29.728 120.862  1.00  0.86           C
ATOM   3474  O   LYS A 435      10.291  30.762 120.740  1.00  0.86           O
ATOM   3475  CB  LYS A 435      11.529  28.390 118.923  1.00  0.86           C
ATOM   3476  CG  LYS A 435      12.445  28.185 117.715  1.00  0.86           C
ATOM   3477  CD  LYS A 435      11.910  27.115 116.755  1.00  0.86           C
ATOM   3478  CE  LYS A 435      12.811  26.916 115.536  1.00  0.86           C
ATOM   3479  NZ  LYS A 435      12.230  25.892 114.643  1.00  0.86           N
ATOM   3480  N   CYS A 436      10.655  28.811 121.803  1.00  0.91           N
ATOM   3481  CA  CYS A 436       9.440  28.904 122.578  1.00  0.91           C
ATOM   3482  C   CYS A 436       9.632  29.266 124.048  1.00  0.91           C
ATOM   3483  O   CYS A 436       8.787  29.929 124.638  1.00  0.91           O
ATOM   3484  CB  CYS A 436       8.624  27.604 122.433  1.00  0.91           C
ATOM   3485  SG  CYS A 436       8.223  27.304 120.685  1.00  0.91           S
ATOM   3486  N   CYS A 437      10.747  28.909 124.715  1.00  0.90           N
ATOM   3487  CA  CYS A 437      10.787  29.005 126.175  1.00  0.90           C
ATOM   3488  C   CYS A 437      10.950  30.396 126.766  1.00  0.90           C
ATOM   3489  O   CYS A 437      10.639  30.600 127.935  1.00  0.90           O
ATOM   3490  CB  CYS A 437      11.771  28.014 126.839  1.00  0.90           C
ATOM   3491  SG  CYS A 437      11.261  26.283 126.593  1.00  0.90           S
ATOM   3492  N   LYS A 438      11.355  31.410 125.969  1.00  0.83           N
ATOM   3493  CA  LYS A 438      11.412  32.778 126.444  1.00  0.83           C
ATOM   3494  C   LYS A 438      10.110  33.526 126.229  1.00  0.83           C
ATOM   3495  O   LYS A 438       9.892  34.579 126.824  1.00  0.83           O
ATOM   3496  CB  LYS A 438      12.538  33.555 125.739  1.00  0.83           C
ATOM   3497  CG  LYS A 438      13.934  33.028 126.083  1.00  0.83           C
ATOM   3498  CD  LYS A 438      14.992  33.916 125.423  1.00  0.83           C
ATOM   3499  CE  LYS A 438      16.416  33.472 125.734  1.00  0.83           C
ATOM   3500  NZ  LYS A 438      17.382  34.358 125.051  1.00  0.83           N
ATOM   3501  N   ASN A 439       9.192  32.980 125.402  1.00  0.84           N
ATOM   3502  CA  ASN A 439       7.870  33.542 125.178  1.00  0.84           C
ATOM   3503  C   ASN A 439       7.074  33.668 126.482  1.00  0.84           C
ATOM   3504  O   ASN A 439       7.347  32.914 127.424  1.00  0.84           O
ATOM   3505  CB  ASN A 439       7.049  32.688 124.183  1.00  0.84           C
ATOM   3506  CG  ASN A 439       7.655  32.831 122.793  1.00  0.84           C
ATOM   3507  OD1 ASN A 439       8.422  33.751 122.505  1.00  0.84           O
```

ATOM	3508	ND2	ASN	A	439	7.315	31.884	121.892	1.00	0.84	N
ATOM	3509	N	PRO	A	440	6.113	34.574	126.619	1.00	0.79	N
ATOM	3510	CA	PRO	A	440	5.076	34.517	127.653	1.00	0.79	C
ATOM	3511	C	PRO	A	440	4.527	33.138	128.025	1.00	0.79	C
ATOM	3512	O	PRO	A	440	4.319	32.303	127.142	1.00	0.79	O
ATOM	3513	CB	PRO	A	440	3.971	35.408	127.067	1.00	0.79	C
ATOM	3514	CG	PRO	A	440	4.717	36.469	126.254	1.00	0.79	C
ATOM	3515	CD	PRO	A	440	5.890	35.681	125.674	1.00	0.79	C
ATOM	3516	N	GLU	A	441	4.241	32.869	129.319	1.00	0.72	N
ATOM	3517	CA	GLU	A	441	3.837	31.548	129.794	1.00	0.72	C
ATOM	3518	C	GLU	A	441	2.636	30.885	129.122	1.00	0.72	C
ATOM	3519	O	GLU	A	441	2.641	29.682	128.854	1.00	0.72	O
ATOM	3520	CB	GLU	A	441	3.557	31.556	131.316	1.00	0.72	C
ATOM	3521	CG	GLU	A	441	4.823	31.317	132.167	1.00	0.72	C
ATOM	3522	CD	GLU	A	441	5.575	32.598	132.515	1.00	0.72	C
ATOM	3523	OE1	GLU	A	441	5.569	32.981	133.708	1.00	0.72	O
ATOM	3524	OE2	GLU	A	441	6.200	33.174	131.585	1.00	0.72	O
ATOM	3525	N	SER	A	442	1.575	31.654	128.816	1.00	0.68	N
ATOM	3526	CA	SER	A	442	0.354	31.144	128.207	1.00	0.68	C
ATOM	3527	C	SER	A	442	0.495	30.930	126.702	1.00	0.68	C
ATOM	3528	O	SER	A	442	−0.340	30.274	126.080	1.00	0.68	O
ATOM	3529	CB	SER	A	442	−0.912	31.981	128.574	1.00	0.68	C
ATOM	3530	OG	SER	A	442	−0.936	33.276	127.978	1.00	0.68	O
ATOM	3531	N	GLU	A	443	1.607	31.418	126.104	1.00	0.77	N
ATOM	3532	CA	GLU	A	443	1.930	31.281	124.698	1.00	0.77	C
ATOM	3533	C	GLU	A	443	2.955	30.169	124.446	1.00	0.77	C
ATOM	3534	O	GLU	A	443	3.055	29.618	123.345	1.00	0.77	O
ATOM	3535	CB	GLU	A	443	2.488	32.639	124.224	1.00	0.77	C
ATOM	3536	CG	GLU	A	443	2.909	32.716	122.743	1.00	0.77	C
ATOM	3537	CD	GLU	A	443	3.426	34.111	122.383	1.00	0.77	C
ATOM	3538	OE1	GLU	A	443	4.109	34.192	121.334	1.00	0.77	O
ATOM	3539	OE2	GLU	A	443	3.173	35.080	123.145	1.00	0.77	O
ATOM	3540	N	ARG	A	444	3.700	29.729	125.491	1.00	0.74	N
ATOM	3541	CA	ARG	A	444	4.758	28.723	125.377	1.00	0.74	C
ATOM	3542	C	ARG	A	444	4.338	27.385	124.781	1.00	0.74	C
ATOM	3543	O	ARG	A	444	5.039	26.799	123.956	1.00	0.74	O
ATOM	3544	CB	ARG	A	444	5.406	28.432	126.754	1.00	0.74	C
ATOM	3545	CG	ARG	A	444	6.565	29.398	127.047	1.00	0.74	C
ATOM	3546	CD	ARG	A	444	7.310	29.152	128.359	1.00	0.74	C
ATOM	3547	NE	ARG	A	444	7.763	30.485	128.847	1.00	0.74	N
ATOM	3548	CZ	ARG	A	444	8.161	30.748	130.104	1.00	0.74	C
ATOM	3549	NH1	ARG	A	444	8.239	29.795	131.033	1.00	0.74	N
ATOM	3550	NH2	ARG	A	444	8.486	31.993	130.427	1.00	0.74	N
ATOM	3551	N	MET	A	445	3.167	26.881	125.209	1.00	0.74	N
ATOM	3552	CA	MET	A	445	2.578	25.639	124.745	1.00	0.74	C
ATOM	3553	C	MET	A	445	2.141	25.663	123.290	1.00	0.74	C
ATOM	3554	O	MET	A	445	2.423	24.734	122.533	1.00	0.74	O
ATOM	3555	CB	MET	A	445	1.383	25.241	125.643	1.00	0.74	C
ATOM	3556	CG	MET	A	445	0.773	23.861	125.298	1.00	0.74	C
ATOM	3557	SD	MET	A	445	−0.598	23.809	124.092	1.00	0.74	S
ATOM	3558	CE	MET	A	445	−1.815	24.676	125.109	1.00	0.74	C
ATOM	3559	N	SER	A	446	1.471	26.763	122.867	1.00	0.77	N
ATOM	3560	CA	SER	A	446	0.998	26.977	121.490	1.00	0.77	C
ATOM	3561	C	SER	A	446	2.163	26.985	120.490	1.00	0.77	C
ATOM	3562	O	SER	A	446	2.146	26.296	119.468	1.00	0.77	O
ATOM	3563	CB	SER	A	446	0.138	28.278	121.345	1.00	0.77	C
ATOM	3564	OG	SER	A	446	−0.354	28.469	120.015	1.00	0.77	O
ATOM	3565	N	CYS	A	447	3.254	27.702	120.845	1.00	0.89	N
ATOM	3566	CA	CYS	A	447	4.526	27.727	120.137	1.00	0.89	C
ATOM	3567	C	CYS	A	447	5.191	26.365	120.047	1.00	0.89	C
ATOM	3568	O	CYS	A	447	5.653	25.951	118.985	1.00	0.89	O
ATOM	3569	CB	CYS	A	447	5.485	28.709	120.872	1.00	0.89	C
ATOM	3570	SG	CYS	A	447	7.139	28.943	120.127	1.00	0.89	S
ATOM	3571	N	ALA	A	448	5.261	25.617	121.167	1.00	0.90	N
ATOM	3572	CA	ALA	A	448	5.880	24.310	121.174	1.00	0.90	C
ATOM	3573	C	ALA	A	448	5.149	23.269	120.317	1.00	0.90	C
ATOM	3574	O	ALA	A	448	5.761	22.540	119.544	1.00	0.90	O
ATOM	3575	CB	ALA	A	448	6.002	23.812	122.623	1.00	0.90	C
ATOM	3576	N	GLU	A	449	3.807	23.199	120.391	1.00	0.78	N
ATOM	3577	CA	GLU	A	449	3.004	22.282	119.577	1.00	0.78	C
ATOM	3578	C	GLU	A	449	3.087	22.546	118.052	1.00	0.78	C
ATOM	3579	O	GLU	A	449	3.103	21.601	117.261	1.00	0.78	O
ATOM	3580	CB	GLU	A	449	1.507	22.287	120.010	1.00	0.78	C
ATOM	3581	CG	GLU	A	449	0.990	21.227	121.043	1.00	0.78	C
ATOM	3582	CD	GLU	A	449	0.940	19.756	120.626	1.00	0.78	C
ATOM	3583	OE1	GLU	A	449	0.449	18.944	121.471	1.00	0.78	O

ATOM	3584	OE2	GLU	A	449	1.319	19.345	119.509	1.00	0.78	O
ATOM	3585	N	ASP	A	450	3.110	23.832	117.591	1.00	0.80	N
ATOM	3586	CA	ASP	A	450	3.278	24.213	116.183	1.00	0.80	C
ATOM	3587	C	ASP	A	450	4.615	23.757	115.642	1.00	0.80	C
ATOM	3588	O	ASP	A	450	4.659	23.003	114.673	1.00	0.80	O
ATOM	3589	CB	ASP	A	450	3.046	25.767	116.025	1.00	0.80	C
ATOM	3590	CG	ASP	A	450	3.627	26.548	114.834	1.00	0.80	C
ATOM	3591	OD1	ASP	A	450	3.329	26.185	113.669	1.00	0.80	O
ATOM	3592	OD2	ASP	A	450	4.335	27.562	115.052	1.00	0.80	O
ATOM	3593	N	TYR	A	451	5.745	24.098	116.302	1.00	0.84	N
ATOM	3594	CA	TYR	A	451	7.027	23.677	115.756	1.00	0.84	C
ATOM	3595	C	TYR	A	451	7.255	22.176	115.782	1.00	0.84	C
ATOM	3596	O	TYR	A	451	7.837	21.594	114.868	1.00	0.84	O
ATOM	3597	CB	TYR	A	451	8.270	24.369	116.343	1.00	0.84	C
ATOM	3598	CG	TYR	A	451	8.378	25.770	115.836	1.00	0.84	C
ATOM	3599	CD1	TYR	A	451	8.002	26.820	116.670	1.00	0.84	C
ATOM	3600	CD2	TYR	A	451	8.923	26.064	114.575	1.00	0.84	C
ATOM	3601	CE1	TYR	A	451	8.224	28.147	116.296	1.00	0.84	C
ATOM	3602	CE2	TYR	A	451	9.145	27.395	114.192	1.00	0.84	C
ATOM	3603	CZ	TYR	A	451	8.818	28.438	115.069	1.00	0.84	C
ATOM	3604	OH	TYR	A	451	9.150	29.770	114.767	1.00	0.84	O
ATOM	3605	N	LEU	A	452	6.797	21.495	116.843	1.00	0.87	N
ATOM	3606	CA	LEU	A	452	7.003	20.076	116.997	1.00	0.87	C
ATOM	3607	C	LEU	A	452	6.377	19.224	115.897	1.00	0.87	C
ATOM	3608	O	LEU	A	452	6.933	18.199	115.499	1.00	0.87	O
ATOM	3609	CB	LEU	A	452	6.529	19.573	118.373	1.00	0.87	C
ATOM	3610	CG	LEU	A	452	7.432	19.940	119.570	1.00	0.87	C
ATOM	3611	CD1	LEU	A	452	6.882	19.311	120.854	1.00	0.87	C
ATOM	3612	CD2	LEU	A	452	8.869	19.477	119.380	1.00	0.87	C
ATOM	3613	N	SER	A	453	5.214	19.636	115.349	1.00	0.85	N
ATOM	3614	CA	SER	A	453	4.561	18.967	114.238	1.00	0.85	C
ATOM	3615	C	SER	A	453	5.405	18.994	112.956	1.00	0.85	C
ATOM	3616	O	SER	A	453	5.458	18.005	112.229	1.00	0.85	O
ATOM	3617	CB	SER	A	453	3.070	19.410	114.101	1.00	0.85	C
ATOM	3618	OG	SER	A	453	2.886	20.570	113.297	1.00	0.85	O
ATOM	3619	N	LEU	A	454	6.153	20.101	112.709	1.00	0.86	N
ATOM	3620	CA	LEU	A	454	7.129	20.283	111.634	1.00	0.86	C
ATOM	3621	C	LEU	A	454	8.328	19.350	111.775	1.00	0.86	C
ATOM	3622	O	LEU	A	454	8.785	18.711	110.820	1.00	0.86	O
ATOM	3623	CB	LEU	A	454	7.663	21.753	111.616	1.00	0.86	C
ATOM	3624	CG	LEU	A	454	6.591	22.858	111.616	1.00	0.86	C
ATOM	3625	CD1	LEU	A	454	7.240	24.247	111.733	1.00	0.86	C
ATOM	3626	CD2	LEU	A	454	5.763	22.773	110.338	1.00	0.86	C
ATOM	3627	N	VAL	A	455	8.860	19.260	113.009	1.00	0.90	N
ATOM	3628	CA	VAL	A	455	10.040	18.479	113.359	1.00	0.90	C
ATOM	3629	C	VAL	A	455	9.782	16.967	113.361	1.00	0.90	C
ATOM	3630	O	VAL	A	455	10.551	16.172	112.821	1.00	0.90	O
ATOM	3631	CB	VAL	A	455	10.639	18.978	114.671	1.00	0.90	C
ATOM	3632	CG1	VAL	A	455	11.919	18.199	115.018	1.00	0.90	C
ATOM	3633	CG2	VAL	A	455	11.008	20.467	114.508	1.00	0.90	C
ATOM	3634	N	LEU	A	456	8.645	16.534	113.938	1.00	0.89	N
ATOM	3635	CA	LEU	A	456	8.176	15.155	113.951	1.00	0.89	C
ATOM	3636	C	LEU	A	456	7.653	14.692	112.593	1.00	0.89	C
ATOM	3637	O	LEU	A	456	7.806	13.530	112.220	1.00	0.89	O
ATOM	3638	CB	LEU	A	456	7.152	14.948	115.100	1.00	0.89	C
ATOM	3639	CG	LEU	A	456	7.755	15.105	116.520	1.00	0.89	C
ATOM	3640	CD1	LEU	A	456	6.677	15.117	117.624	1.00	0.89	C
ATOM	3641	CD2	LEU	A	456	8.814	14.027	116.804	1.00	0.89	C
ATOM	3642	N	ASN	A	457	7.084	15.597	111.762	1.00	0.87	N
ATOM	3643	CA	ASN	A	457	6.879	15.336	110.349	1.00	0.87	C
ATOM	3644	C	ASN	A	457	8.202	15.065	109.625	1.00	0.87	C
ATOM	3645	O	ASN	A	457	8.269	14.130	108.831	1.00	0.87	O
ATOM	3646	CB	ASN	A	457	6.069	16.469	109.666	1.00	0.87	C
ATOM	3647	CG	ASN	A	457	5.741	16.082	108.235	1.00	0.87	C
ATOM	3648	OD1	ASN	A	457	4.994	15.117	108.023	1.00	0.87	O
ATOM	3649	ND2	ASN	A	457	6.329	16.800	107.253	1.00	0.87	N
ATOM	3650	N	ARG	A	458	9.289	15.830	109.901	1.00	0.81	N
ATOM	3651	CA	ARG	A	458	10.592	15.547	109.302	1.00	0.81	C
ATOM	3652	C	ARG	A	458	11.115	14.171	109.660	1.00	0.81	C
ATOM	3653	O	ARG	A	458	11.542	13.442	108.769	1.00	0.81	O
ATOM	3654	CB	ARG	A	458	11.691	16.604	109.621	1.00	0.81	C
ATOM	3655	CG	ARG	A	458	13.150	16.167	109.347	1.00	0.81	C
ATOM	3656	CD	ARG	A	458	14.065	17.193	108.675	1.00	0.81	C
ATOM	3657	NE	ARG	A	458	13.929	17.025	107.185	1.00	0.81	N
ATOM	3658	CZ	ARG	A	458	14.901	16.602	106.353	1.00	0.81	C
ATOM	3659	NH1	ARG	A	458	16.152	16.417	106.733	1.00	0.81	N

ATOM	3660	NH2	ARG	A	458	14.566	16.354	105.087	1.00	0.81	N
ATOM	3661	N	LEU	A	459	11.039	13.761	110.944	1.00	0.88	N
ATOM	3662	CA	LEU	A	459	11.448	12.439	111.391	1.00	0.88	C
ATOM	3663	C	LEU	A	459	10.694	11.341	110.664	1.00	0.88	C
ATOM	3664	O	LEU	A	459	11.291	10.375	110.200	1.00	0.88	O
ATOM	3665	CB	LEU	A	459	11.158	12.308	112.907	1.00	0.88	C
ATOM	3666	CG	LEU	A	459	11.265	10.903	113.547	1.00	0.88	C
ATOM	3667	CD1	LEU	A	459	12.705	10.510	113.899	1.00	0.88	C
ATOM	3668	CD2	LEU	A	459	10.328	10.817	114.755	1.00	0.88	C
ATOM	3669	N	CYS	A	460	9.365	11.503	110.513	1.00	0.91	N
ATOM	3670	CA	CYS	A	460	8.529	10.617	109.717	1.00	0.91	C
ATOM	3671	C	CYS	A	460	8.889	10.541	108.236	1.00	0.91	C
ATOM	3672	O	CYS	A	460	8.926	9.464	107.662	1.00	0.91	O
ATOM	3673	CB	CYS	A	460	7.029	11.001	109.786	1.00	0.91	C
ATOM	3674	SG	CYS	A	460	6.324	10.956	111.452	1.00	0.91	S
ATOM	3675	N	VAL	A	461	9.157	11.686	107.588	1.00	0.89	N
ATOM	3676	CA	VAL	A	461	9.619	11.801	106.211	1.00	0.89	C
ATOM	3677	C	VAL	A	461	11.000	11.208	105.996	1.00	0.89	C
ATOM	3678	O	VAL	A	461	11.267	10.502	105.032	1.00	0.89	O
ATOM	3679	CB	VAL	A	461	9.562	13.274	105.820	1.00	0.89	C
ATOM	3680	CG1	VAL	A	461	10.492	13.643	104.652	1.00	0.89	C
ATOM	3681	CG2	VAL	A	461	8.100	13.621	105.479	1.00	0.89	C
ATOM	3682	N	LEU	A	462	11.955	11.449	106.912	1.00	0.86	N
ATOM	3683	CA	LEU	A	462	13.264	10.824	106.840	1.00	0.86	C
ATOM	3684	C	LEU	A	462	13.221	9.315	107.003	1.00	0.86	C
ATOM	3685	O	LEU	A	462	13.960	8.579	106.342	1.00	0.86	O
ATOM	3686	CB	LEU	A	462	14.222	11.395	107.907	1.00	0.86	C
ATOM	3687	CG	LEU	A	462	14.841	12.767	107.585	1.00	0.86	C
ATOM	3688	CD1	LEU	A	462	15.574	13.286	108.834	1.00	0.86	C
ATOM	3689	CD2	LEU	A	462	15.788	12.700	106.375	1.00	0.86	C
ATOM	3690	N	HIS	A	463	12.365	8.857	107.927	1.00	0.83	N
ATOM	3691	CA	HIS	A	463	12.156	7.481	108.279	1.00	0.83	C
ATOM	3692	C	HIS	A	463	11.334	6.660	107.280	1.00	0.83	C
ATOM	3693	O	HIS	A	463	11.657	5.499	107.050	1.00	0.83	O
ATOM	3694	CB	HIS	A	463	11.556	7.438	109.688	1.00	0.83	C
ATOM	3695	CG	HIS	A	463	11.456	6.071	110.220	1.00	0.83	C
ATOM	3696	ND1	HIS	A	463	10.232	5.451	110.286	1.00	0.83	N
ATOM	3697	CD2	HIS	A	463	12.447	5.221	110.565	1.00	0.83	C
ATOM	3698	CE1	HIS	A	463	10.492	4.226	110.672	1.00	0.83	C
ATOM	3699	NE2	HIS	A	463	11.825	4.032	110.857	1.00	0.83	N
ATOM	3700	N	GLU	A	464	10.280	7.224	106.634	1.00	0.83	N
ATOM	3701	CA	GLU	A	464	9.380	6.494	105.718	1.00	0.83	C
ATOM	3702	C	GLU	A	464	10.096	5.886	104.529	1.00	0.83	C
ATOM	3703	O	GLU	A	464	9.798	4.779	104.085	1.00	0.83	O
ATOM	3704	CB	GLU	A	464	8.105	7.281	105.245	1.00	0.83	C
ATOM	3705	CG	GLU	A	464	7.918	7.667	103.745	1.00	0.83	C
ATOM	3706	CD	GLU	A	464	8.220	9.128	103.402	1.00	0.83	C
ATOM	3707	OE1	GLU	A	464	7.250	9.927	103.561	1.00	0.83	O
ATOM	3708	OE2	GLU	A	464	9.328	9.407	102.896	1.00	0.83	O
ATOM	3709	N	LYS	A	465	11.086	6.623	104.009	1.00	0.81	N
ATOM	3710	CA	LYS	A	465	11.992	6.245	102.948	1.00	0.81	C
ATOM	3711	C	LYS	A	465	12.792	4.973	103.208	1.00	0.81	C
ATOM	3712	O	LYS	A	465	13.052	4.173	102.314	1.00	0.81	O
ATOM	3713	CB	LYS	A	465	12.997	7.424	102.830	1.00	0.81	C
ATOM	3714	CG	LYS	A	465	14.230	7.169	101.940	1.00	0.81	C
ATOM	3715	CD	LYS	A	465	15.191	8.359	101.765	1.00	0.81	C
ATOM	3716	CE	LYS	A	465	15.690	9.021	103.048	1.00	0.81	C
ATOM	3717	NZ	LYS	A	465	15.809	10.471	102.778	1.00	0.81	N
ATOM	3718	N	THR	A	466	13.245	4.802	104.461	1.00	0.86	N
ATOM	3719	CA	THR	A	466	14.037	3.662	104.905	1.00	0.86	C
ATOM	3720	C	THR	A	466	13.413	3.134	106.197	1.00	0.86	C
ATOM	3721	O	THR	A	466	13.859	3.485	107.292	1.00	0.86	O
ATOM	3722	CB	THR	A	466	15.539	3.996	105.009	1.00	0.86	C
ATOM	3723	OG1	THR	A	466	16.320	2.884	105.429	1.00	0.86	O
ATOM	3724	CG2	THR	A	466	15.887	5.205	105.902	1.00	0.86	C
ATOM	3725	N	PRO	A	467	12.376	2.280	106.183	1.00	0.89	N
ATOM	3726	CA	PRO	A	467	11.569	2.077	107.380	1.00	0.89	C
ATOM	3727	C	PRO	A	467	12.224	0.985	108.215	1.00	0.89	C
ATOM	3728	O	PRO	A	467	12.441	−0.115	107.711	1.00	0.89	O
ATOM	3729	CB	PRO	A	467	10.180	1.599	106.881	1.00	0.89	C
ATOM	3730	CG	PRO	A	467	10.198	1.737	105.355	1.00	0.89	C
ATOM	3731	CD	PRO	A	467	11.682	1.798	104.985	1.00	0.89	C
ATOM	3732	N	VAL	A	468	12.561	1.309	109.493	1.00	0.89	N
ATOM	3733	CA	VAL	A	468	13.318	0.445	110.406	1.00	0.89	C
ATOM	3734	C	VAL	A	468	12.908	0.452	111.894	1.00	0.89	C
ATOM	3735	O	VAL	A	468	13.420	−0.337	112.684	1.00	0.89	O

ATOM	3736	CB	VAL	A	468	14.818	0.783	110.415	1.00	0.89	C
ATOM	3737	CG1	VAL	A	468	15.423	0.581	109.014	1.00	0.89	C
ATOM	3738	CG2	VAL	A	468	15.123	2.196	110.965	1.00	0.89	C
ATOM	3739	N	SER	A	469	11.984	1.334	112.341	1.00	0.86	N
ATOM	3740	CA	SER	A	469	11.501	1.347	113.722	1.00	0.86	C
ATOM	3741	C	SER	A	469	10.007	1.308	113.541	1.00	0.86	C
ATOM	3742	O	SER	A	469	9.409	2.218	112.970	1.00	0.86	O
ATOM	3743	CB	SER	A	469	11.937	2.578	114.607	1.00	0.86	C
ATOM	3744	OG	SER	A	469	11.033	2.937	115.663	1.00	0.86	O
ATOM	3745	N	GLU	A	470	9.387	0.223	114.018	1.00	0.85	N
ATOM	3746	CA	GLU	A	470	7.969	-0.074	114.083	1.00	0.85	C
ATOM	3747	C	GLU	A	470	7.229	0.940	114.949	1.00	0.85	C
ATOM	3748	O	GLU	A	470	6.093	1.335	114.679	1.00	0.85	O
ATOM	3749	CB	GLU	A	470	7.729	-1.535	114.582	1.00	0.85	C
ATOM	3750	CG	GLU	A	470	8.859	-2.535	114.204	1.00	0.85	C
ATOM	3751	CD	GLU	A	470	9.991	-2.418	115.240	1.00	0.85	C
ATOM	3752	OE1	GLU	A	470	10.964	-1.674	114.955	1.00	0.85	O
ATOM	3753	OE2	GLU	A	470	9.842	-2.926	116.377	1.00	0.85	O
ATOM	3754	N	ARG	A	471	7.903	1.419	116.018	1.00	0.79	N
ATOM	3755	CA	ARG	A	471	7.432	2.437	116.944	1.00	0.79	C
ATOM	3756	C	ARG	A	471	7.145	3.778	116.270	1.00	0.79	C
ATOM	3757	O	ARG	A	471	6.100	4.391	116.512	1.00	0.79	O
ATOM	3758	CB	ARG	A	471	8.504	2.687	118.035	1.00	0.79	C
ATOM	3759	CG	ARG	A	471	8.701	1.514	119.014	1.00	0.79	C
ATOM	3760	CD	ARG	A	471	10.006	1.603	119.825	1.00	0.79	C
ATOM	3761	NE	ARG	A	471	10.100	0.355	120.655	1.00	0.79	N
ATOM	3762	CZ	ARG	A	471	10.614	-0.815	120.218	1.00	0.79	C
ATOM	3763	NH1	ARG	A	471	10.477	-1.910	120.950	1.00	0.79	N
ATOM	3764	NH2	ARG	A	471	11.221	-0.979	119.056	1.00	0.79	N
ATOM	3765	N	VAL	A	472	8.096	4.213	115.408	1.00	0.91	N
ATOM	3766	CA	VAL	A	472	8.053	5.423	114.590	1.00	0.91	C
ATOM	3767	C	VAL	A	472	7.023	5.308	113.497	1.00	0.91	C
ATOM	3768	O	VAL	A	472	6.173	6.186	113.371	1.00	0.91	O
ATOM	3769	CB	VAL	A	472	9.421	5.783	114.003	1.00	0.91	C
ATOM	3770	CG1	VAL	A	472	9.362	6.969	113.015	1.00	0.91	C
ATOM	3771	CG2	VAL	A	472	10.310	6.174	115.188	1.00	0.91	C
ATOM	3772	N	THR	A	473	7.004	4.174	112.753	1.00	0.90	N
ATOM	3773	CA	THR	A	473	6.036	3.912	111.680	1.00	0.90	C
ATOM	3774	C	THR	A	473	4.616	4.035	112.170	1.00	0.90	C
ATOM	3775	O	THR	A	473	3.831	4.778	111.591	1.00	0.90	O
ATOM	3776	CB	THR	A	473	6.189	2.523	111.063	1.00	0.90	C
ATOM	3777	OG1	THR	A	473	7.457	2.388	110.439	1.00	0.90	O
ATOM	3778	CG2	THR	A	473	5.140	2.231	109.975	1.00	0.90	C
ATOM	3779	N	LYS	A	474	4.288	3.393	113.314	1.00	0.87	N
ATOM	3780	CA	LYS	A	474	3.022	3.580	114.001	1.00	0.87	C
ATOM	3781	C	LYS	A	474	2.734	5.045	114.315	1.00	0.87	C
ATOM	3782	O	LYS	A	474	1.729	5.579	113.871	1.00	0.87	O
ATOM	3783	CB	LYS	A	474	3.017	2.692	115.284	1.00	0.87	C
ATOM	3784	CG	LYS	A	474	1.953	2.998	116.357	1.00	0.87	C
ATOM	3785	CD	LYS	A	474	2.072	2.157	117.652	1.00	0.87	C
ATOM	3786	CE	LYS	A	474	3.416	2.183	118.391	1.00	0.87	C
ATOM	3787	NZ	LYS	A	474	4.017	3.529	118.307	1.00	0.87	N
ATOM	3788	N	CYS	A	475	3.632	5.793	115.005	1.00	0.93	N
ATOM	3789	CA	CYS	A	475	3.282	7.152	115.397	1.00	0.93	C
ATOM	3790	C	CYS	A	475	3.128	8.123	114.236	1.00	0.93	C
ATOM	3791	O	CYS	A	475	2.394	9.104	114.333	1.00	0.93	O
ATOM	3792	CB	CYS	A	475	4.242	7.762	116.449	1.00	0.93	C
ATOM	3793	SG	CYS	A	475	4.089	7.080	118.132	1.00	0.93	S
ATOM	3794	N	CYS	A	476	3.807	7.853	113.110	1.00	0.92	N
ATOM	3795	CA	CYS	A	476	3.702	8.621	111.893	1.00	0.92	C
ATOM	3796	C	CYS	A	476	2.487	8.335	111.009	1.00	0.92	C
ATOM	3797	O	CYS	A	476	2.045	9.229	110.283	1.00	0.92	O
ATOM	3798	CB	CYS	A	476	4.954	8.386	111.033	1.00	0.92	C
ATOM	3799	SG	CYS	A	476	6.450	8.992	111.854	1.00	0.92	S
ATOM	3800	N	THR	A	477	1.940	7.100	111.011	1.00	0.89	N
ATOM	3801	CA	THR	A	477	0.821	6.698	110.153	1.00	0.89	C
ATOM	3802	C	THR	A	477	-0.521	6.622	110.858	1.00	0.89	C
ATOM	3803	O	THR	A	477	-1.558	6.768	110.214	1.00	0.89	O
ATOM	3804	CB	THR	A	477	1.017	5.318	109.525	1.00	0.89	C
ATOM	3805	OG1	THR	A	477	1.261	4.317	110.503	1.00	0.89	O
ATOM	3806	CG2	THR	A	477	2.239	5.352	108.599	1.00	0.89	C
ATOM	3807	N	GLU	A	478	-0.556	6.434	112.193	1.00	0.84	N
ATOM	3808	CA	GLU	A	478	-1.780	6.324	112.977	1.00	0.84	C
ATOM	3809	C	GLU	A	478	-2.651	7.563	112.917	1.00	0.84	C
ATOM	3810	O	GLU	A	478	-3.881	7.545	112.849	1.00	0.84	O
ATOM	3811	CB	GLU	A	478	-1.390	6.074	114.453	1.00	0.84	C

ATOM	3812	CG	GLU	A	478	-2.411	5.260	115.284	1.00	0.84	C
ATOM	3813	CD	GLU	A	478	-2.191	3.745	115.206	1.00	0.84	C
ATOM	3814	OE1	GLU	A	478	-1.141	3.306	114.671	1.00	0.84	O
ATOM	3815	OE2	GLU	A	478	-3.067	3.020	115.741	1.00	0.84	O
ATOM	3816	N	SER	A	479	-1.989	8.727	112.942	1.00	0.85	N
ATOM	3817	CA	SER	A	479	-2.675	9.977	112.817	1.00	0.85	C
ATOM	3818	C	SER	A	479	-1.696	11.007	112.322	1.00	0.85	C
ATOM	3819	O	SER	A	479	-0.480	10.824	112.369	1.00	0.85	O
ATOM	3820	CB	SER	A	479	-3.395	10.369	114.150	1.00	0.85	C
ATOM	3821	OG	SER	A	479	-2.861	11.463	114.889	1.00	0.85	O
ATOM	3822	N	LEU	A	480	-2.228	12.112	111.774	1.00	0.82	N
ATOM	3823	CA	LEU	A	480	-1.479	13.333	111.599	1.00	0.82	C
ATOM	3824	C	LEU	A	480	-1.367	14.193	112.860	1.00	0.82	C
ATOM	3825	O	LEU	A	480	-0.301	14.695	113.205	1.00	0.82	O
ATOM	3826	CB	LEU	A	480	-2.100	14.156	110.455	1.00	0.82	C
ATOM	3827	CG	LEU	A	480	-1.268	15.375	110.036	1.00	0.82	C
ATOM	3828	CD1	LEU	A	480	0.173	14.977	109.682	1.00	0.82	C
ATOM	3829	CD2	LEU	A	480	-1.920	16.078	108.847	1.00	0.82	C
ATOM	3830	N	VAL	A	481	-2.478	14.378	113.603	1.00	0.83	N
ATOM	3831	CA	VAL	A	481	-2.598	15.394	114.626	1.00	0.83	C
ATOM	3832	C	VAL	A	481	-2.144	15.019	116.057	1.00	0.83	C
ATOM	3833	O	VAL	A	481	-1.849	15.869	116.904	1.00	0.83	O
ATOM	3834	CB	VAL	A	481	-4.044	15.855	114.560	1.00	0.83	C
ATOM	3835	CG1	VAL	A	481	-5.093	14.777	114.914	1.00	0.83	C
ATOM	3836	CG2	VAL	A	481	-4.195	17.118	115.390	1.00	0.83	C
ATOM	3837	N	ASN	A	482	-2.010	13.703	116.328	1.00	0.84	N
ATOM	3838	CA	ASN	A	482	-1.720	13.076	117.617	1.00	0.84	C
ATOM	3839	C	ASN	A	482	-0.327	12.479	117.611	1.00	0.84	C
ATOM	3840	O	ASN	A	482	0.025	11.673	118.469	1.00	0.84	O
ATOM	3841	CB	ASN	A	482	-2.753	11.997	118.080	1.00	0.84	C
ATOM	3842	CG	ASN	A	482	-4.032	12.701	118.509	1.00	0.84	C
ATOM	3843	OD1	ASN	A	482	-3.967	13.589	119.353	1.00	0.84	O
ATOM	3844	ND2	ASN	A	482	-5.216	12.315	117.978	1.00	0.84	N
ATOM	3845	N	ARG	A	483	0.535	12.905	116.667	1.00	0.81	N
ATOM	3846	CA	ARG	A	483	1.935	12.524	116.676	1.00	0.81	C
ATOM	3847	C	ARG	A	483	2.700	13.001	117.888	1.00	0.81	C
ATOM	3848	O	ARG	A	483	3.396	12.229	118.536	1.00	0.81	O
ATOM	3849	CB	ARG	A	483	2.656	13.102	115.452	1.00	0.81	C
ATOM	3850	CG	ARG	A	483	2.299	12.356	114.164	1.00	0.81	C
ATOM	3851	CD	ARG	A	483	2.628	13.178	112.929	1.00	0.81	C
ATOM	3852	NE	ARG	A	483	2.414	12.302	111.740	1.00	0.81	N
ATOM	3853	CZ	ARG	A	483	2.958	12.560	110.544	1.00	0.81	C
ATOM	3854	NH1	ARG	A	483	3.723	13.635	110.346	1.00	0.81	N
ATOM	3855	NH2	ARG	A	483	2.764	11.727	109.530	1.00	0.81	N
ATOM	3856	N	ARG	A	484	2.569	14.285	118.258	1.00	0.80	N
ATOM	3857	CA	ARG	A	484	3.261	14.821	119.416	1.00	0.80	C
ATOM	3858	C	ARG	A	484	2.952	14.126	120.765	1.00	0.80	C
ATOM	3859	O	ARG	A	484	3.900	13.848	121.504	1.00	0.80	O
ATOM	3860	CB	ARG	A	484	3.071	16.360	119.419	1.00	0.80	C
ATOM	3861	CG	ARG	A	484	4.093	17.205	120.213	1.00	0.80	C
ATOM	3862	CD	ARG	A	484	4.123	17.079	121.740	1.00	0.80	C
ATOM	3863	NE	ARG	A	484	2.731	17.315	122.205	1.00	0.80	N
ATOM	3864	CZ	ARG	A	484	2.100	16.652	123.180	1.00	0.80	C
ATOM	3865	NH1	ARG	A	484	2.689	15.718	123.923	1.00	0.80	N
ATOM	3866	NH2	ARG	A	484	0.802	16.922	123.338	1.00	0.80	N
ATOM	3867	N	PRO	A	485	1.729	13.770	121.159	1.00	0.86	N
ATOM	3868	CA	PRO	A	485	1.471	12.852	122.262	1.00	0.86	C
ATOM	3869	C	PRO	A	485	1.907	11.414	121.993	1.00	0.86	C
ATOM	3870	O	PRO	A	485	2.416	10.801	122.928	1.00	0.86	O
ATOM	3871	CB	PRO	A	485	-0.017	13.040	122.615	1.00	0.86	C
ATOM	3872	CG	PRO	A	485	-0.652	13.794	121.439	1.00	0.86	C
ATOM	3873	CD	PRO	A	485	0.513	14.370	120.638	1.00	0.86	C
ATOM	3874	N	CYS	A	486	1.777	10.846	120.766	1.00	0.92	N
ATOM	3875	CA	CYS	A	486	2.239	9.485	120.468	1.00	0.92	C
ATOM	3876	C	CYS	A	486	3.742	9.339	120.693	1.00	0.92	C
ATOM	3877	O	CYS	A	486	4.202	8.415	121.361	1.00	0.92	O
ATOM	3878	CB	CYS	A	486	1.850	9.051	119.018	1.00	0.92	C
ATOM	3879	SG	CYS	A	486	2.109	7.291	118.559	1.00	0.92	S
ATOM	3880	N	PHE	A	487	4.533	10.316	120.198	1.00	0.89	N
ATOM	3881	CA	PHE	A	487	5.966	10.394	120.416	1.00	0.89	C
ATOM	3882	C	PHE	A	487	6.362	10.626	121.868	1.00	0.89	C
ATOM	3883	O	PHE	A	487	7.273	9.975	122.393	1.00	0.89	O
ATOM	3884	CB	PHE	A	487	6.660	11.424	119.486	1.00	0.89	C
ATOM	3885	CG	PHE	A	487	6.930	10.798	118.143	1.00	0.89	C
ATOM	3886	CD1	PHE	A	487	8.041	9.953	117.999	1.00	0.89	C
ATOM	3887	CD2	PHE	A	487	6.086	10.988	117.036	1.00	0.89	C

ATOM	3888	CE1	PHE	A	487	8.298	9.306	116.790	1.00	0.89	C
ATOM	3889	CE2	PHE	A	487	6.339	10.338	115.821	1.00	0.89	C
ATOM	3890	CZ	PHE	A	487	7.448	9.500	115.698	1.00	0.89	C
ATOM	3891	N	SER	A	488	5.666	11.536	122.584	1.00	0.86	N
ATOM	3892	CA	SER	A	488	5.907	11.794	123.999	1.00	0.86	C
ATOM	3893	C	SER	A	488	5.611	10.599	124.909	1.00	0.86	C
ATOM	3894	O	SER	A	488	6.357	10.368	125.858	1.00	0.86	O
ATOM	3895	CB	SER	A	488	5.247	13.076	124.587	1.00	0.86	C
ATOM	3896	OG	SER	A	488	5.674	14.287	123.949	1.00	0.86	O
ATOM	3897	N	ALA	A	489	4.574	9.790	124.589	1.00	0.90	N
ATOM	3898	CA	ALA	A	489	4.137	8.609	125.323	1.00	0.90	C
ATOM	3899	C	ALA	A	489	4.985	7.358	125.053	1.00	0.90	C
ATOM	3900	O	ALA	A	489	4.807	6.326	125.698	1.00	0.90	O
ATOM	3901	CB	ALA	A	489	2.654	8.296	124.992	1.00	0.90	C
ATOM	3902	N	LEU	A	490	5.930	7.400	124.082	1.00	0.88	N
ATOM	3903	CA	LEU	A	490	6.914	6.339	123.891	1.00	0.88	C
ATOM	3904	C	LEU	A	490	7.907	6.187	125.022	1.00	0.88	C
ATOM	3905	O	LEU	A	490	8.450	7.153	125.565	1.00	0.88	O
ATOM	3906	CB	LEU	A	490	7.748	6.483	122.600	1.00	0.88	C
ATOM	3907	CG	LEU	A	490	6.952	6.286	121.309	1.00	0.88	C
ATOM	3908	CD1	LEU	A	490	7.687	7.011	120.177	1.00	0.88	C
ATOM	3909	CD2	LEU	A	490	6.773	4.797	120.981	1.00	0.88	C
ATOM	3910	N	THR	A	491	8.217	4.931	125.335	1.00	0.85	N
ATOM	3911	CA	THR	A	491	9.085	4.525	126.403	1.00	0.85	C
ATOM	3912	C	THR	A	491	10.311	3.895	125.788	1.00	0.85	C
ATOM	3913	O	THR	A	491	10.338	3.541	124.612	1.00	0.85	O
ATOM	3914	CB	THR	A	491	8.367	3.548	127.341	1.00	0.85	C
ATOM	3915	OG1	THR	A	491	7.791	2.462	126.635	1.00	0.85	O
ATOM	3916	CG2	THR	A	491	7.178	4.270	128.000	1.00	0.85	C
ATOM	3917	N	VAL	A	492	11.403	3.811	126.575	1.00	0.84	N
ATOM	3918	CA	VAL	A	492	12.525	2.914	126.320	1.00	0.84	C
ATOM	3919	C	VAL	A	492	12.044	1.474	126.482	1.00	0.84	C
ATOM	3920	O	VAL	A	492	11.571	1.077	127.545	1.00	0.84	O
ATOM	3921	CB	VAL	A	492	13.714	3.229	127.250	1.00	0.84	C
ATOM	3922	CG1	VAL	A	492	13.378	3.065	128.750	1.00	0.84	C
ATOM	3923	CG2	VAL	A	492	14.981	2.424	126.875	1.00	0.84	C
ATOM	3924	N	ASP	A	493	12.116	0.668	125.410	1.00	0.68	N
ATOM	3925	CA	ASP	A	493	11.717	−0.720	125.428	1.00	0.68	C
ATOM	3926	C	ASP	A	493	12.937	−1.616	125.655	1.00	0.68	C
ATOM	3927	O	ASP	A	493	14.016	−1.400	125.102	1.00	0.68	O
ATOM	3928	CB	ASP	A	493	11.119	−1.143	124.053	1.00	0.68	C
ATOM	3929	CG	ASP	A	493	9.594	−0.986	123.877	1.00	0.68	C
ATOM	3930	OD1	ASP	A	493	8.942	−0.189	124.557	1.00	0.68	O
ATOM	3931	OD2	ASP	A	493	9.055	−1.698	122.989	1.00	0.68	O
ATOM	3932	N	GLU	A	494	12.735	−2.740	126.378	1.00	0.68	N
ATOM	3933	CA	GLU	A	494	13.732	−3.767	126.656	1.00	0.68	C
ATOM	3934	C	GLU	A	494	13.562	−4.910	125.675	1.00	0.68	C
ATOM	3935	O	GLU	A	494	13.838	−6.075	125.929	1.00	0.68	O
ATOM	3936	CB	GLU	A	494	13.719	−4.206	128.139	1.00	0.68	C
ATOM	3937	CG	GLU	A	494	14.191	−3.006	128.999	1.00	0.68	C
ATOM	3938	CD	GLU	A	494	14.859	−3.307	130.338	1.00	0.68	C
ATOM	3939	OE1	GLU	A	494	14.229	−3.168	131.410	1.00	0.68	O
ATOM	3940	OE2	GLU	A	494	16.107	−3.448	130.283	1.00	0.68	O
ATOM	3941	N	THR	A	495	13.092	−4.520	124.480	1.00	0.70	N
ATOM	3942	CA	THR	A	495	12.778	−5.345	123.315	1.00	0.70	C
ATOM	3943	C	THR	A	495	13.826	−5.071	122.251	1.00	0.70	C
ATOM	3944	O	THR	A	495	13.750	−5.492	121.102	1.00	0.70	O
ATOM	3945	CB	THR	A	495	11.386	−4.959	122.813	1.00	0.70	C
ATOM	3946	OG1	THR	A	495	10.434	−5.177	123.842	1.00	0.70	O
ATOM	3947	CG2	THR	A	495	10.852	−5.708	121.583	1.00	0.70	C
ATOM	3948	N	TYR	A	496	14.879	−4.337	122.635	1.00	0.81	N
ATOM	3949	CA	TYR	A	496	15.944	−3.884	121.776	1.00	0.81	C
ATOM	3950	C	TYR	A	496	17.214	−4.573	122.247	1.00	0.81	C
ATOM	3951	O	TYR	A	496	17.635	−4.422	123.392	1.00	0.81	O
ATOM	3952	CB	TYR	A	496	16.021	−2.330	121.860	1.00	0.81	C
ATOM	3953	CG	TYR	A	496	17.264	−1.745	121.239	1.00	0.81	C
ATOM	3954	CD1	TYR	A	496	17.448	−1.729	119.848	1.00	0.81	C
ATOM	3955	CD2	TYR	A	496	18.293	−1.264	122.066	1.00	0.81	C
ATOM	3956	CE1	TYR	A	496	18.641	−1.246	119.294	1.00	0.81	C
ATOM	3957	CE2	TYR	A	496	19.484	−0.775	121.513	1.00	0.81	C
ATOM	3958	CZ	TYR	A	496	19.651	−0.758	120.125	1.00	0.81	C
ATOM	3959	OH	TYR	A	496	20.836	−0.259	119.558	1.00	0.81	O
ATOM	3960	N	GLU	A	497	17.841	−5.380	121.366	1.00	0.77	N
ATOM	3961	CA	GLU	A	497	19.180	−5.892	121.591	1.00	0.77	C
ATOM	3962	C	GLU	A	497	20.165	−4.733	121.439	1.00	0.77	C
ATOM	3963	O	GLU	A	497	20.070	−4.049	120.417	1.00	0.77	O

```
ATOM   3964  CB  GLU A 497      19.517  -7.032 120.608  1.00  0.77           C
ATOM   3965  CG  GLU A 497      20.858  -7.732 120.911  1.00  0.77           C
ATOM   3966  CD  GLU A 497      21.113  -8.909 119.968  1.00  0.77           C
ATOM   3967  OE1 GLU A 497      22.167  -9.567 120.157  1.00  0.77           O
ATOM   3968  OE2 GLU A 497      20.265  -9.172 119.074  1.00  0.77           O
ATOM   3969  N   PRO A 498      21.056  -4.385 122.377  1.00  0.85           N
ATOM   3970  CA  PRO A 498      22.137  -3.432 122.146  1.00  0.85           C
ATOM   3971  C   PRO A 498      22.951  -3.689 120.905  1.00  0.85           C
ATOM   3972  O   PRO A 498      23.213  -4.841 120.570  1.00  0.85           O
ATOM   3973  CB  PRO A 498      23.016  -3.507 123.404  1.00  0.85           C
ATOM   3974  CG  PRO A 498      22.081  -4.085 124.463  1.00  0.85           C
ATOM   3975  CD  PRO A 498      21.228  -5.066 123.657  1.00  0.85           C
ATOM   3976  N   LYS A 499      23.401  -2.624 120.222  1.00  0.80           N
ATOM   3977  CA  LYS A 499      24.300  -2.769 119.104  1.00  0.80           C
ATOM   3978  C   LYS A 499      25.612  -3.403 119.564  1.00  0.80           C
ATOM   3979  O   LYS A 499      26.119  -3.064 120.626  1.00  0.80           O
ATOM   3980  CB  LYS A 499      24.601  -1.376 118.506  1.00  0.80           C
ATOM   3981  CG  LYS A 499      25.108  -1.396 117.056  1.00  0.80           C
ATOM   3982  CD  LYS A 499      25.513   0.015 116.594  1.00  0.80           C
ATOM   3983  CE  LYS A 499      25.303   0.287 115.102  1.00  0.80           C
ATOM   3984  NZ  LYS A 499      25.212   1.739 114.897  1.00  0.80           N
ATOM   3985  N   ALA A 500      26.221  -4.330 118.801  1.00  0.83           N
ATOM   3986  CA  ALA A 500      27.595  -4.716 119.041  1.00  0.83           C
ATOM   3987  C   ALA A 500      28.560  -3.528 118.951  1.00  0.83           C
ATOM   3988  O   ALA A 500      28.342  -2.596 118.178  1.00  0.83           O
ATOM   3989  CB  ALA A 500      28.001  -5.793 118.018  1.00  0.83           C
ATOM   3990  N   PHE A 501      29.635  -3.526 119.773  1.00  0.75           N
ATOM   3991  CA  PHE A 501      30.636  -2.471 119.777  1.00  0.75           C
ATOM   3992  C   PHE A 501      31.326  -2.345 118.421  1.00  0.75           C
ATOM   3993  O   PHE A 501      31.630  -3.359 117.802  1.00  0.75           O
ATOM   3994  CB  PHE A 501      31.661  -2.766 120.907  1.00  0.75           C
ATOM   3995  CG  PHE A 501      32.748  -1.730 121.038  1.00  0.75           C
ATOM   3996  CD1 PHE A 501      32.496  -0.438 121.525  1.00  0.75           C
ATOM   3997  CD2 PHE A 501      34.048  -2.039 120.608  1.00  0.75           C
ATOM   3998  CE1 PHE A 501      33.522   0.514 121.590  1.00  0.75           C
ATOM   3999  CE2 PHE A 501      35.075  -1.094 120.687  1.00  0.75           C
ATOM   4000  CZ  PHE A 501      34.814   0.185 121.175  1.00  0.75           C
ATOM   4001  N   ASP A 502      31.557  -1.099 117.954  1.00  0.75           N
ATOM   4002  CA  ASP A 502      32.247  -0.827 116.724  1.00  0.75           C
ATOM   4003  C   ASP A 502      33.275   0.225 117.110  1.00  0.75           C
ATOM   4004  O   ASP A 502      32.943   1.304 117.605  1.00  0.75           O
ATOM   4005  CB  ASP A 502      31.199  -0.360 115.670  1.00  0.75           C
ATOM   4006  CG  ASP A 502      31.746  -0.052 114.273  1.00  0.75           C
ATOM   4007  OD1 ASP A 502      30.891   0.138 113.367  1.00  0.75           O
ATOM   4008  OD2 ASP A 502      32.989   0.063 114.094  1.00  0.75           O
ATOM   4009  N   GLU A 503      34.569  -0.103 116.945  1.00  0.67           N
ATOM   4010  CA  GLU A 503      35.718   0.729 117.225  1.00  0.67           C
ATOM   4011  C   GLU A 503      35.698   2.037 116.443  1.00  0.67           C
ATOM   4012  O   GLU A 503      36.012   3.110 116.959  1.00  0.67           O
ATOM   4013  CB  GLU A 503      37.031  -0.038 116.871  1.00  0.67           C
ATOM   4014  CG  GLU A 503      37.141  -1.498 117.394  1.00  0.67           C
ATOM   4015  CD  GLU A 503      36.300  -2.485 116.591  1.00  0.67           C
ATOM   4016  OE1 GLU A 503      36.634  -2.732 115.406  1.00  0.67           O
ATOM   4017  OE2 GLU A 503      35.288  -2.947 117.174  1.00  0.67           O
ATOM   4018  N   LYS A 504      35.283   1.980 115.161  1.00  0.70           N
ATOM   4019  CA  LYS A 504      35.365   3.086 114.224  1.00  0.70           C
ATOM   4020  C   LYS A 504      34.388   4.217 114.534  1.00  0.70           C
ATOM   4021  O   LYS A 504      34.594   5.351 114.098  1.00  0.70           O
ATOM   4022  CB  LYS A 504      35.196   2.583 112.768  1.00  0.70           C
ATOM   4023  CG  LYS A 504      36.358   1.684 112.301  1.00  0.70           C
ATOM   4024  CD  LYS A 504      36.164   1.144 110.872  1.00  0.70           C
ATOM   4025  CE  LYS A 504      36.697  -0.284 110.699  1.00  0.70           C
ATOM   4026  NZ  LYS A 504      37.697  -0.351 109.608  1.00  0.70           N
ATOM   4027  N   THR A 505      33.334   3.958 115.339  1.00  0.75           N
ATOM   4028  CA  THR A 505      32.357   4.969 115.754  1.00  0.75           C
ATOM   4029  C   THR A 505      32.901   5.896 116.850  1.00  0.75           C
ATOM   4030  O   THR A 505      32.415   7.019 117.032  1.00  0.75           O
ATOM   4031  CB  THR A 505      31.002   4.405 116.216  1.00  0.75           C
ATOM   4032  OG1 THR A 505      31.102   3.564 117.347  1.00  0.75           O
ATOM   4033  CG2 THR A 505      30.352   3.540 115.133  1.00  0.75           C
ATOM   4034  N   PHE A 506      33.968   5.457 117.568  1.00  0.76           N
ATOM   4035  CA  PHE A 506      34.513   6.119 118.746  1.00  0.76           C
ATOM   4036  C   PHE A 506      35.993   6.480 118.591  1.00  0.76           C
ATOM   4037  O   PHE A 506      36.679   6.825 119.554  1.00  0.76           O
ATOM   4038  CB  PHE A 506      34.311   5.251 120.017  1.00  0.76           C
ATOM   4039  CG  PHE A 506      32.855   4.938 120.237  1.00  0.76           C
```

ATOM	4040	CD1	PHE	A	506	32.398	3.616	120.154	1.00	0.76	C
ATOM	4041	CD2	PHE	A	506	31.923	5.949	120.514	1.00	0.76	C
ATOM	4042	CE1	PHE	A	506	31.053	3.299	120.376	1.00	0.76	C
ATOM	4043	CE2	PHE	A	506	30.570	5.639	120.701	1.00	0.76	C
ATOM	4044	CZ	PHE	A	506	30.135	4.312	120.645	1.00	0.76	C
ATOM	4045	N	THR	A	507	36.510	6.456	117.348	1.00	0.76	N
ATOM	4046	CA	THR	A	507	37.881	6.856	117.021	1.00	0.76	C
ATOM	4047	C	THR	A	507	37.833	8.290	116.530	1.00	0.76	C
ATOM	4048	O	THR	A	507	36.961	8.686	115.752	1.00	0.76	O
ATOM	4049	CB	THR	A	507	38.599	5.967	115.999	1.00	0.76	C
ATOM	4050	OG1	THR	A	507	38.554	4.613	116.411	1.00	0.76	O
ATOM	4051	CG2	THR	A	507	40.103	6.276	115.914	1.00	0.76	C
ATOM	4052	N	PHE	A	508	38.758	9.141	117.012	1.00	0.79	N
ATOM	4053	CA	PHE	A	508	38.765	10.557	116.714	1.00	0.79	C
ATOM	4054	C	PHE	A	508	40.168	10.933	116.309	1.00	0.79	C
ATOM	4055	O	PHE	A	508	41.135	10.335	116.771	1.00	0.79	O
ATOM	4056	CB	PHE	A	508	38.342	11.413	117.933	1.00	0.79	C
ATOM	4057	CG	PHE	A	508	36.968	11.034	118.387	1.00	0.79	C
ATOM	4058	CD1	PHE	A	508	35.834	11.646	117.842	1.00	0.79	C
ATOM	4059	CD2	PHE	A	508	36.801	10.054	119.372	1.00	0.79	C
ATOM	4060	CE1	PHE	A	508	34.554	11.305	118.295	1.00	0.79	C
ATOM	4061	CE2	PHE	A	508	35.523	9.685	119.804	1.00	0.79	C
ATOM	4062	CZ	PHE	A	508	34.395	10.323	119.280	1.00	0.79	C
ATOM	4063	N	HIS	A	509	40.289	11.931	115.414	1.00	0.77	N
ATOM	4064	CA	HIS	A	509	41.536	12.306	114.781	1.00	0.77	C
ATOM	4065	C	HIS	A	509	41.692	13.803	114.938	1.00	0.77	C
ATOM	4066	O	HIS	A	509	40.826	14.479	115.491	1.00	0.77	O
ATOM	4067	CB	HIS	A	509	41.588	11.935	113.273	1.00	0.77	C
ATOM	4068	CG	HIS	A	509	40.994	10.601	112.958	1.00	0.77	C
ATOM	4069	ND1	HIS	A	509	41.808	9.540	112.619	1.00	0.77	N
ATOM	4070	CD2	HIS	A	509	39.695	10.204	113.005	1.00	0.77	C
ATOM	4071	CE1	HIS	A	509	40.990	8.520	112.468	1.00	0.77	C
ATOM	4072	NE2	HIS	A	509	39.699	8.863	112.699	1.00	0.77	N
ATOM	4073	N	ALA	A	510	42.808	14.383	114.449	1.00	0.86	N
ATOM	4074	CA	ALA	A	510	43.076	15.805	114.567	1.00	0.86	C
ATOM	4075	C	ALA	A	510	42.103	16.730	113.798	1.00	0.86	C
ATOM	4076	O	ALA	A	510	42.048	17.934	114.062	1.00	0.86	O
ATOM	4077	CB	ALA	A	510	44.542	16.048	114.151	1.00	0.86	C
ATOM	4078	N	ASP	A	511	41.287	16.170	112.864	1.00	0.81	N
ATOM	4079	CA	ASP	A	511	40.220	16.818	112.113	1.00	0.81	C
ATOM	4080	C	ASP	A	511	39.115	17.395	112.985	1.00	0.81	C
ATOM	4081	O	ASP	A	511	38.525	18.413	112.659	1.00	0.81	O
ATOM	4082	CB	ASP	A	511	39.629	15.905	110.987	1.00	0.81	C
ATOM	4083	CG	ASP	A	511	38.952	14.635	111.500	1.00	0.81	C
ATOM	4084	OD1	ASP	A	511	37.912	14.745	112.194	1.00	0.81	O
ATOM	4085	OD2	ASP	A	511	39.495	13.538	111.218	1.00	0.81	O
ATOM	4086	N	LEU	A	512	38.854	16.800	114.169	1.00	0.84	N
ATOM	4087	CA	LEU	A	512	37.791	17.256	115.054	1.00	0.84	C
ATOM	4088	C	LEU	A	512	38.048	18.646	115.649	1.00	0.84	C
ATOM	4089	O	LEU	A	512	37.139	19.362	116.078	1.00	0.84	O
ATOM	4090	CB	LEU	A	512	37.573	16.220	116.181	1.00	0.84	C
ATOM	4091	CG	LEU	A	512	36.353	16.489	117.082	1.00	0.84	C
ATOM	4092	CD1	LEU	A	512	35.064	16.516	116.252	1.00	0.84	C
ATOM	4093	CD2	LEU	A	512	36.253	15.455	118.208	1.00	0.84	C
ATOM	4094	N	CYS	A	513	39.321	19.083	115.631	1.00	0.86	N
ATOM	4095	CA	CYS	A	513	39.763	20.400	116.046	1.00	0.86	C
ATOM	4096	C	CYS	A	513	39.784	21.405	114.884	1.00	0.86	C
ATOM	4097	O	CYS	A	513	40.165	22.557	115.086	1.00	0.86	O
ATOM	4098	CB	CYS	A	513	41.239	20.365	116.527	1.00	0.86	C
ATOM	4099	SG	CYS	A	513	41.675	19.057	117.713	1.00	0.86	S
ATOM	4100	N	THR	A	514	39.395	20.980	113.658	1.00	0.80	N
ATOM	4101	CA	THR	A	514	39.456	21.762	112.407	1.00	0.80	C
ATOM	4102	C	THR	A	514	38.081	22.185	111.969	1.00	0.80	C
ATOM	4103	O	THR	A	514	37.867	23.317	111.542	1.00	0.80	O
ATOM	4104	CB	THR	A	514	39.984	20.938	111.215	1.00	0.80	C
ATOM	4105	OG1	THR	A	514	41.371	20.682	111.355	1.00	0.80	O
ATOM	4106	CG2	THR	A	514	39.807	21.545	109.800	1.00	0.80	C
ATOM	4107	N	LEU	A	515	37.097	21.269	112.065	1.00	0.80	N
ATOM	4108	CA	LEU	A	515	35.762	21.421	111.506	1.00	0.80	C
ATOM	4109	C	LEU	A	515	35.035	22.736	111.795	1.00	0.80	C
ATOM	4110	O	LEU	A	515	35.172	23.231	112.922	1.00	0.80	O
ATOM	4111	CB	LEU	A	515	34.822	20.308	112.033	1.00	0.80	C
ATOM	4112	CG	LEU	A	515	35.349	18.866	111.957	1.00	0.80	C
ATOM	4113	CD1	LEU	A	515	34.423	17.894	112.705	1.00	0.80	C
ATOM	4114	CD2	LEU	A	515	35.573	18.402	110.513	1.00	0.80	C
ATOM	4115	N	PRO	A	516	34.231	23.323	110.877	1.00	0.71	N

ATOM	4116	CA	PRO	A	516	33.171	24.286	111.186	1.00	0.71	C
ATOM	4117	C	PRO	A	516	32.412	23.972	112.456	1.00	0.71	C
ATOM	4118	O	PRO	A	516	32.193	22.792	112.732	1.00	0.71	O
ATOM	4119	CB	PRO	A	516	32.243	24.248	109.954	1.00	0.71	C
ATOM	4120	CG	PRO	A	516	33.087	23.726	108.784	1.00	0.71	C
ATOM	4121	CD	PRO	A	516	34.307	23.061	109.432	1.00	0.71	C
ATOM	4122	N	GLU	A	517	31.982	24.981	113.243	1.00	0.72	N
ATOM	4123	CA	GLU	A	517	31.425	24.737	114.558	1.00	0.72	C
ATOM	4124	C	GLU	A	517	30.230	23.784	114.543	1.00	0.72	C
ATOM	4125	O	GLU	A	517	30.186	22.801	115.278	1.00	0.72	O
ATOM	4126	CB	GLU	A	517	31.086	26.069	115.266	1.00	0.72	C
ATOM	4127	CG	GLU	A	517	30.511	25.841	116.683	1.00	0.72	C
ATOM	4128	CD	GLU	A	517	31.411	24.991	117.591	1.00	0.72	C
ATOM	4129	OE1	GLU	A	517	30.830	24.410	118.545	1.00	0.72	O
ATOM	4130	OE2	GLU	A	517	32.639	24.857	117.328	1.00	0.72	O
ATOM	4131	N	ASN	A	518	29.287	23.972	113.592	1.00	0.77	N
ATOM	4132	CA	ASN	A	518	28.173	23.048	113.406	1.00	0.77	C
ATOM	4133	C	ASN	A	518	28.596	21.581	113.200	1.00	0.77	C
ATOM	4134	O	ASN	A	518	28.027	20.681	113.805	1.00	0.77	O
ATOM	4135	CB	ASN	A	518	27.283	23.426	112.187	1.00	0.77	C
ATOM	4136	CG	ASN	A	518	26.367	24.615	112.464	1.00	0.77	C
ATOM	4137	OD1	ASN	A	518	25.938	24.892	113.583	1.00	0.77	O
ATOM	4138	ND2	ASN	A	518	25.978	25.322	111.369	1.00	0.77	N
ATOM	4139	N	GLU	A	519	29.615	21.322	112.356	1.00	0.78	N
ATOM	4140	CA	GLU	A	519	30.198	20.008	112.109	1.00	0.78	C
ATOM	4141	C	GLU	A	519	30.880	19.383	113.321	1.00	0.78	C
ATOM	4142	O	GLU	A	519	30.777	18.180	113.568	1.00	0.78	O
ATOM	4143	CB	GLU	A	519	31.190	20.044	110.928	1.00	0.78	C
ATOM	4144	CG	GLU	A	519	30.534	19.835	109.543	1.00	0.78	C
ATOM	4145	CD	GLU	A	519	31.557	19.366	108.498	1.00	0.78	C
ATOM	4146	OE1	GLU	A	519	31.614	19.997	107.412	1.00	0.78	O
ATOM	4147	OE2	GLU	A	519	32.271	18.372	108.784	1.00	0.78	O
ATOM	4148	N	LYS	A	520	31.583	20.197	114.133	1.00	0.77	N
ATOM	4149	CA	LYS	A	520	32.181	19.759	115.383	1.00	0.77	C
ATOM	4150	C	LYS	A	520	31.184	19.160	116.357	1.00	0.77	C
ATOM	4151	O	LYS	A	520	31.401	18.086	116.923	1.00	0.77	O
ATOM	4152	CB	LYS	A	520	32.818	20.967	116.114	1.00	0.77	C
ATOM	4153	CG	LYS	A	520	34.343	20.966	116.259	1.00	0.77	C
ATOM	4154	CD	LYS	A	520	34.749	22.093	117.229	1.00	0.77	C
ATOM	4155	CE	LYS	A	520	36.077	22.804	116.961	1.00	0.77	C
ATOM	4156	NZ	LYS	A	520	36.037	23.439	115.631	1.00	0.77	N
ATOM	4157	N	GLN	A	521	30.064	19.877	116.569	1.00	0.80	N
ATOM	4158	CA	GLN	A	521	28.974	19.453	117.420	1.00	0.80	C
ATOM	4159	C	GLN	A	521	28.298	18.222	116.866	1.00	0.80	C
ATOM	4160	O	GLN	A	521	28.061	17.260	117.591	1.00	0.80	O
ATOM	4161	CB	GLN	A	521	27.919	20.561	117.595	1.00	0.80	C
ATOM	4162	CG	GLN	A	521	28.496	21.933	117.986	1.00	0.80	C
ATOM	4163	CD	GLN	A	521	27.416	23.014	117.904	1.00	0.80	C
ATOM	4164	OE1	GLN	A	521	26.264	22.798	117.505	1.00	0.80	O
ATOM	4165	NE2	GLN	A	521	27.844	24.246	118.261	1.00	0.80	N
ATOM	4166	N	ILE	A	522	28.036	18.188	115.540	1.00	0.86	N
ATOM	4167	CA	ILE	A	522	27.427	17.032	114.893	1.00	0.86	C
ATOM	4168	C	ILE	A	522	28.299	15.785	115.063	1.00	0.86	C
ATOM	4169	O	ILE	A	522	27.820	14.741	115.500	1.00	0.86	O
ATOM	4170	CB	ILE	A	522	27.053	17.311	113.432	1.00	0.86	C
ATOM	4171	CG1	ILE	A	522	25.912	18.361	113.391	1.00	0.86	C
ATOM	4172	CG2	ILE	A	522	26.636	16.014	112.697	1.00	0.86	C
ATOM	4173	CD1	ILE	A	522	25.472	18.787	111.984	1.00	0.86	C
ATOM	4174	N	LYS	A	523	29.633	15.884	114.840	1.00	0.82	N
ATOM	4175	CA	LYS	A	523	30.538	14.757	115.030	1.00	0.82	C
ATOM	4176	C	LYS	A	523	30.523	14.159	116.445	1.00	0.82	C
ATOM	4177	O	LYS	A	523	30.598	12.944	116.630	1.00	0.82	O
ATOM	4178	CB	LYS	A	523	32.008	15.060	114.629	1.00	0.82	C
ATOM	4179	CG	LYS	A	523	32.918	13.819	114.778	1.00	0.82	C
ATOM	4180	CD	LYS	A	523	33.777	13.490	113.548	1.00	0.82	C
ATOM	4181	CE	LYS	A	523	33.913	11.983	113.266	1.00	0.82	C
ATOM	4182	NZ	LYS	A	523	34.483	11.279	114.437	1.00	0.82	N
ATOM	4183	N	LYS	A	524	30.428	15.022	117.473	1.00	0.81	N
ATOM	4184	CA	LYS	A	524	30.187	14.651	118.861	1.00	0.81	C
ATOM	4185	C	LYS	A	524	28.855	13.996	119.126	1.00	0.81	C
ATOM	4186	O	LYS	A	524	28.775	12.941	119.754	1.00	0.81	O
ATOM	4187	CB	LYS	A	524	30.284	15.923	119.730	1.00	0.81	C
ATOM	4188	CG	LYS	A	524	31.766	16.248	119.934	1.00	0.81	C
ATOM	4189	CD	LYS	A	524	32.098	17.671	120.406	1.00	0.81	C
ATOM	4190	CE	LYS	A	524	33.331	18.241	119.688	1.00	0.81	C
ATOM	4191	NZ	LYS	A	524	33.675	19.581	120.211	1.00	0.81	N

ATOM	4192	N	GLN	A	525	27.774	14.599	118.623	1.00	0.85	N
ATOM	4193	CA	GLN	A	525	26.413	14.167	118.841	1.00	0.85	C
ATOM	4194	C	GLN	A	525	26.071	12.860	118.137	1.00	0.85	C
ATOM	4195	O	GLN	A	525	25.245	12.086	118.616	1.00	0.85	O
ATOM	4196	CB	GLN	A	525	25.487	15.330	118.427	1.00	0.85	C
ATOM	4197	CG	GLN	A	525	25.587	16.541	119.399	1.00	0.85	C
ATOM	4198	CD	GLN	A	525	24.919	17.834	118.883	1.00	0.85	C
ATOM	4199	OE1	GLN	A	525	25.071	18.308	117.757	1.00	0.85	O
ATOM	4200	NE2	GLN	A	525	24.143	18.499	119.771	1.00	0.85	N
ATOM	4201	N	ILE	A	526	26.757	12.554	117.016	1.00	0.88	N
ATOM	4202	CA	ILE	A	526	26.788	11.233	116.387	1.00	0.88	C
ATOM	4203	C	ILE	A	526	27.370	10.181	117.305	1.00	0.88	C
ATOM	4204	O	ILE	A	526	26.795	9.107	117.492	1.00	0.88	O
ATOM	4205	CB	ILE	A	526	27.626	11.253	115.107	1.00	0.88	C
ATOM	4206	CG1	ILE	A	526	26.874	12.007	113.995	1.00	0.88	C
ATOM	4207	CG2	ILE	A	526	28.028	9.833	114.622	1.00	0.88	C
ATOM	4208	CD1	ILE	A	526	27.779	12.369	112.816	1.00	0.88	C
ATOM	4209	N	ALA	A	527	28.530	10.482	117.922	1.00	0.88	N
ATOM	4210	CA	ALA	A	527	29.187	9.598	118.861	1.00	0.88	C
ATOM	4211	C	ALA	A	527	28.359	9.377	120.131	1.00	0.88	C
ATOM	4212	O	ALA	A	527	28.306	8.283	120.687	1.00	0.88	O
ATOM	4213	CB	ALA	A	527	30.607	10.101	119.183	1.00	0.88	C
ATOM	4214	N	LEU	A	528	27.644	10.420	120.601	1.00	0.86	N
ATOM	4215	CA	LEU	A	528	26.688	10.317	121.694	1.00	0.86	C
ATOM	4216	C	LEU	A	528	25.529	9.377	121.400	1.00	0.86	C
ATOM	4217	O	LEU	A	528	25.137	8.569	122.243	1.00	0.86	O
ATOM	4218	CB	LEU	A	528	26.111	11.703	122.066	1.00	0.86	C
ATOM	4219	CG	LEU	A	528	25.263	11.738	123.360	1.00	0.86	C
ATOM	4220	CD1	LEU	A	528	26.068	11.297	124.593	1.00	0.86	C
ATOM	4221	CD2	LEU	A	528	24.640	13.125	123.591	1.00	0.86	C
ATOM	4222	N	VAL	A	529	24.962	9.444	120.174	1.00	0.90	N
ATOM	4223	CA	VAL	A	529	23.949	8.497	119.720	1.00	0.90	C
ATOM	4224	C	VAL	A	529	24.466	7.066	119.664	1.00	0.90	C
ATOM	4225	O	VAL	A	529	23.864	6.151	120.229	1.00	0.90	O
ATOM	4226	CB	VAL	A	529	23.402	8.894	118.353	1.00	0.90	C
ATOM	4227	CG1	VAL	A	529	22.565	7.786	117.696	1.00	0.90	C
ATOM	4228	CG2	VAL	A	529	22.510	10.122	118.528	1.00	0.90	C
ATOM	4229	N	GLU	A	530	25.636	6.847	119.025	1.00	0.84	N
ATOM	4230	CA	GLU	A	530	26.243	5.532	118.874	1.00	0.84	C
ATOM	4231	C	GLU	A	530	26.584	4.878	120.208	1.00	0.84	C
ATOM	4232	O	GLU	A	530	26.443	3.668	120.393	1.00	0.84	O
ATOM	4233	CB	GLU	A	530	27.450	5.558	117.900	1.00	0.84	C
ATOM	4234	CG	GLU	A	530	27.048	5.845	116.419	1.00	0.84	C
ATOM	4235	CD	GLU	A	530	26.243	4.738	115.747	1.00	0.84	C
ATOM	4236	OE1	GLU	A	530	26.072	3.653	116.360	1.00	0.84	O
ATOM	4237	OE2	GLU	A	530	25.749	4.924	114.602	1.00	0.84	O
ATOM	4238	N	LEU	A	531	26.991	5.695	121.199	1.00	0.83	N
ATOM	4239	CA	LEU	A	531	27.177	5.291	122.581	1.00	0.83	C
ATOM	4240	C	LEU	A	531	25.909	4.750	123.249	1.00	0.83	C
ATOM	4241	O	LEU	A	531	25.935	3.673	123.847	1.00	0.83	O
ATOM	4242	CB	LEU	A	531	27.776	6.481	123.367	1.00	0.83	C
ATOM	4243	CG	LEU	A	531	28.064	6.229	124.859	1.00	0.83	C
ATOM	4244	CD1	LEU	A	531	29.105	5.123	125.101	1.00	0.83	C
ATOM	4245	CD2	LEU	A	531	28.490	7.535	125.547	1.00	0.83	C
ATOM	4246	N	VAL	A	532	24.750	5.437	123.113	1.00	0.87	N
ATOM	4247	CA	VAL	A	532	23.463	4.981	123.625	1.00	0.87	C
ATOM	4248	C	VAL	A	532	22.924	3.761	122.885	1.00	0.87	C
ATOM	4249	O	VAL	A	532	22.349	2.868	123.500	1.00	0.87	O
ATOM	4250	CB	VAL	A	532	22.460	6.115	123.853	1.00	0.87	C
ATOM	4251	CG1	VAL	A	532	21.125	5.567	124.400	1.00	0.87	C
ATOM	4252	CG2	VAL	A	532	23.079	7.051	124.914	1.00	0.87	C
ATOM	4253	N	LYS	A	533	23.138	3.622	121.557	1.00	0.83	N
ATOM	4254	CA	LYS	A	533	22.739	2.404	120.849	1.00	0.83	C
ATOM	4255	C	LYS	A	533	23.455	1.140	121.332	1.00	0.83	C
ATOM	4256	O	LYS	A	533	22.882	0.047	121.372	1.00	0.83	O
ATOM	4257	CB	LYS	A	533	22.914	2.481	119.312	1.00	0.83	C
ATOM	4258	CG	LYS	A	533	22.130	3.626	118.667	1.00	0.83	C
ATOM	4259	CD	LYS	A	533	22.085	3.525	117.140	1.00	0.83	C
ATOM	4260	CE	LYS	A	533	21.332	4.706	116.537	1.00	0.83	C
ATOM	4261	NZ	LYS	A	533	20.884	4.350	115.192	1.00	0.83	N
ATOM	4262	N	HIS	A	534	24.750	1.293	121.668	1.00	0.81	N
ATOM	4263	CA	HIS	A	534	25.617	0.285	122.250	1.00	0.81	C
ATOM	4264	C	HIS	A	534	25.306	0.022	123.711	1.00	0.81	C
ATOM	4265	O	HIS	A	534	25.338	−1.102	124.206	1.00	0.81	O
ATOM	4266	CB	HIS	A	534	27.098	0.739	122.122	1.00	0.81	C
ATOM	4267	CG	HIS	A	534	28.102	−0.197	122.718	1.00	0.81	C

```
ATOM   4268  ND1 HIS A 534      27.893  -1.530 122.494  1.00  0.81           N
ATOM   4269  CD2 HIS A 534      29.257  -0.020 123.414  1.00  0.81           C
ATOM   4270  CE1 HIS A 534      28.893  -2.153 123.041  1.00  0.81           C
ATOM   4271  NE2 HIS A 534      29.766  -1.290 123.613  1.00  0.81           N
ATOM   4272  N   LYS A 535      25.032   1.090 124.484  1.00  0.81           N
ATOM   4273  CA  LYS A 535      24.796   0.970 125.910  1.00  0.81           C
ATOM   4274  C   LYS A 535      23.505   1.627 126.402  1.00  0.81           C
ATOM   4275  O   LYS A 535      23.528   2.657 127.081  1.00  0.81           O
ATOM   4276  CB  LYS A 535      25.995   1.621 126.631  1.00  0.81           C
ATOM   4277  CG  LYS A 535      27.316   0.859 126.359  1.00  0.81           C
ATOM   4278  CD  LYS A 535      27.434  -0.282 127.377  1.00  0.81           C
ATOM   4279  CE  LYS A 535      28.538  -1.284 127.057  1.00  0.81           C
ATOM   4280  NZ  LYS A 535      28.184  -2.644 127.543  1.00  0.81           N
ATOM   4281  N   PRO A 536      22.352   1.032 126.139  1.00  0.86           N
ATOM   4282  CA  PRO A 536      21.075   1.733 126.188  1.00  0.86           C
ATOM   4283  C   PRO A 536      20.593   1.987 127.600  1.00  0.86           C
ATOM   4284  O   PRO A 536      19.672   2.780 127.795  1.00  0.86           O
ATOM   4285  CB  PRO A 536      20.136   0.834 125.356  1.00  0.86           C
ATOM   4286  CG  PRO A 536      20.813  -0.534 125.330  1.00  0.86           C
ATOM   4287  CD  PRO A 536      22.270  -0.140 125.282  1.00  0.86           C
ATOM   4288  N   LYS A 537      21.208   1.331 128.603  1.00  0.80           N
ATOM   4289  CA  LYS A 537      20.792   1.435 129.987  1.00  0.80           C
ATOM   4290  C   LYS A 537      21.639   2.395 130.802  1.00  0.80           C
ATOM   4291  O   LYS A 537      21.297   2.674 131.950  1.00  0.80           O
ATOM   4292  CB  LYS A 537      20.839   0.065 130.723  1.00  0.80           C
ATOM   4293  CG  LYS A 537      20.171  -1.118 129.997  1.00  0.80           C
ATOM   4294  CD  LYS A 537      18.639  -1.017 129.892  1.00  0.80           C
ATOM   4295  CE  LYS A 537      17.935  -1.298 131.227  1.00  0.80           C
ATOM   4296  NZ  LYS A 537      16.657  -0.554 131.361  1.00  0.80           N
ATOM   4297  N   VAL A 538      22.736   2.938 130.231  1.00  0.83           N
ATOM   4298  CA  VAL A 538      23.670   3.808 130.931  1.00  0.83           C
ATOM   4299  C   VAL A 538      22.994   4.985 131.627  1.00  0.83           C
ATOM   4300  O   VAL A 538      22.056   5.621 131.122  1.00  0.83           O
ATOM   4301  CB  VAL A 538      24.830   4.157 130.006  1.00  0.83           C
ATOM   4302  CG1 VAL A 538      25.730   5.283 130.528  1.00  0.83           C
ATOM   4303  CG2 VAL A 538      25.714   2.903 129.923  1.00  0.83           C
ATOM   4304  N   THR A 539      23.406   5.237 132.890  1.00  0.79           N
ATOM   4305  CA  THR A 539      22.732   6.222 133.724  1.00  0.79           C
ATOM   4306  C   THR A 539      23.048   7.632 133.305  1.00  0.79           C
ATOM   4307  O   THR A 539      24.006   7.918 132.589  1.00  0.79           O
ATOM   4308  CB  THR A 539      22.758   6.089 135.262  1.00  0.79           C
ATOM   4309  OG1 THR A 539      23.841   6.752 135.884  1.00  0.79           O
ATOM   4310  CG2 THR A 539      22.848   4.634 135.713  1.00  0.79           C
ATOM   4311  N   GLU A 540      22.201   8.577 133.742  1.00  0.72           N
ATOM   4312  CA  GLU A 540      22.449   9.980 133.547  1.00  0.72           C
ATOM   4313  C   GLU A 540      23.733  10.446 134.250  1.00  0.72           C
ATOM   4314  O   GLU A 540      24.536  11.183 133.683  1.00  0.72           O
ATOM   4315  CB  GLU A 540      21.211  10.758 133.998  1.00  0.72           C
ATOM   4316  CG  GLU A 540      21.082  12.104 133.263  1.00  0.72           C
ATOM   4317  CD  GLU A 540      20.838  13.228 134.261  1.00  0.72           C
ATOM   4318  OE1 GLU A 540      21.825  13.931 134.614  1.00  0.72           O
ATOM   4319  OE2 GLU A 540      19.676  13.357 134.720  1.00  0.72           O
ATOM   4320  N   GLU A 541      24.002   9.933 135.484  1.00  0.71           N
ATOM   4321  CA  GLU A 541      25.257  10.148 136.200  1.00  0.71           C
ATOM   4322  C   GLU A 541      26.457   9.622 135.420  1.00  0.71           C
ATOM   4323  O   GLU A 541      27.439  10.338 135.223  1.00  0.71           O
ATOM   4324  CB  GLU A 541      25.224   9.499 137.607  1.00  0.71           C
ATOM   4325  CG  GLU A 541      26.577   9.436 138.373  1.00  0.71           C
ATOM   4326  CD  GLU A 541      26.855   7.996 138.832  1.00  0.71           C
ATOM   4327  OE1 GLU A 541      26.556   7.061 138.038  1.00  0.71           O
ATOM   4328  OE2 GLU A 541      27.347   7.826 139.971  1.00  0.71           O
ATOM   4329  N   GLN A 542      26.394   8.388 134.883  1.00  0.75           N
ATOM   4330  CA  GLN A 542      27.489   7.846 134.098  1.00  0.75           C
ATOM   4331  C   GLN A 542      27.751   8.612 132.809  1.00  0.75           C
ATOM   4332  O   GLN A 542      28.896   8.895 132.478  1.00  0.75           O
ATOM   4333  CB  GLN A 542      27.288   6.351 133.747  1.00  0.75           C
ATOM   4334  CG  GLN A 542      27.002   5.443 134.970  1.00  0.75           C
ATOM   4335  CD  GLN A 542      26.582   4.017 134.587  1.00  0.75           C
ATOM   4336  OE1 GLN A 542      26.013   3.780 133.522  1.00  0.75           O
ATOM   4337  NE2 GLN A 542      26.791   3.059 135.530  1.00  0.75           N
ATOM   4338  N   LEU A 543      26.702   9.012 132.060  1.00  0.80           N
ATOM   4339  CA  LEU A 543      26.844   9.841 130.864  1.00  0.80           C
ATOM   4340  C   LEU A 543      27.446  11.204 131.144  1.00  0.80           C
ATOM   4341  O   LEU A 543      28.270  11.699 130.383  1.00  0.80           O
ATOM   4342  CB  LEU A 543      25.519  10.019 130.084  1.00  0.80           C
ATOM   4343  CG  LEU A 543      24.966   8.700 129.518  1.00  0.80           C
```

ATOM	4344	CD1	LEU	A	543	23.489	8.824	129.114	1.00	0.80	C
ATOM	4345	CD2	LEU	A	543	25.833	8.107	128.393	1.00	0.80	C
ATOM	4346	N	LYS	A	544	27.097	11.831	132.274	1.00	0.69	N
ATOM	4347	CA	LYS	A	544	27.700	13.062	132.744	1.00	0.69	C
ATOM	4348	C	LYS	A	544	29.208	12.967	132.951	1.00	0.69	C
ATOM	4349	O	LYS	A	544	29.970	13.850	132.560	1.00	0.69	O
ATOM	4350	CB	LYS	A	544	27.041	13.357	134.096	1.00	0.69	C
ATOM	4351	CG	LYS	A	544	27.090	14.801	134.593	1.00	0.69	C
ATOM	4352	CD	LYS	A	544	26.577	14.913	136.046	1.00	0.69	C
ATOM	4353	CE	LYS	A	544	25.195	14.271	136.265	1.00	0.69	C
ATOM	4354	NZ	LYS	A	544	24.756	14.370	137.677	1.00	0.69	N
ATOM	4355	N	THR	A	545	29.649	11.835	133.540	1.00	0.80	N
ATOM	4356	CA	THR	A	545	31.051	11.443	133.646	1.00	0.80	C
ATOM	4357	C	THR	A	545	31.691	11.287	132.273	1.00	0.80	C
ATOM	4358	O	THR	A	545	32.708	11.910	131.987	1.00	0.80	O
ATOM	4359	CB	THR	A	545	31.214	10.149	134.447	1.00	0.80	C
ATOM	4360	OG1	THR	A	545	30.675	10.290	135.754	1.00	0.80	O
ATOM	4361	CG2	THR	A	545	32.686	9.753	134.640	1.00	0.80	C
ATOM	4362	N	VAL	A	546	31.065	10.530	131.338	1.00	0.82	N
ATOM	4363	CA	VAL	A	546	31.602	10.312	129.991	1.00	0.82	C
ATOM	4364	C	VAL	A	546	31.707	11.598	129.176	1.00	0.82	C
ATOM	4365	O	VAL	A	546	32.702	11.862	128.502	1.00	0.82	O
ATOM	4366	CB	VAL	A	546	30.822	9.284	129.159	1.00	0.82	C
ATOM	4367	CG1	VAL	A	546	31.522	9.018	127.805	1.00	0.82	C
ATOM	4368	CG2	VAL	A	546	30.712	7.938	129.900	1.00	0.82	C
ATOM	4369	N	MET	A	547	30.678	12.464	129.218	1.00	0.75	N
ATOM	4370	CA	MET	A	547	30.652	13.693	128.443	1.00	0.75	C
ATOM	4371	C	MET	A	547	31.688	14.711	128.890	1.00	0.75	C
ATOM	4372	O	MET	A	547	32.254	15.453	128.087	1.00	0.75	O
ATOM	4373	CB	MET	A	547	29.239	14.304	128.403	1.00	0.75	C
ATOM	4374	CG	MET	A	547	28.227	13.395	127.686	1.00	0.75	C
ATOM	4375	SD	MET	A	547	26.501	13.912	127.956	1.00	0.75	S
ATOM	4376	CE	MET	A	547	26.504	15.472	127.020	1.00	0.75	C
ATOM	4377	N	GLY	A	548	31.960	14.745	130.206	1.00	0.82	N
ATOM	4378	CA	GLY	A	548	33.043	15.515	130.799	1.00	0.82	C
ATOM	4379	C	GLY	A	548	34.448	14.941	130.576	1.00	0.82	C
ATOM	4380	O	GLY	A	548	35.383	15.690	130.294	1.00	0.82	O
ATOM	4381	N	ASP	A	549	34.619	13.596	130.651	1.00	0.81	N
ATOM	4382	CA	ASP	A	549	35.828	12.859	130.284	1.00	0.81	C
ATOM	4383	C	ASP	A	549	36.196	13.067	128.801	1.00	0.81	C
ATOM	4384	O	ASP	A	549	37.359	13.269	128.447	1.00	0.81	O
ATOM	4385	CB	ASP	A	549	35.660	11.334	130.592	1.00	0.81	C
ATOM	4386	CG	ASP	A	549	35.760	10.890	132.050	1.00	0.81	C
ATOM	4387	OD1	ASP	A	549	36.112	11.633	132.989	1.00	0.81	O
ATOM	4388	OD2	ASP	A	549	35.518	9.658	132.222	1.00	0.81	O
ATOM	4389	N	PHE	A	550	35.194	13.061	127.893	1.00	0.80	N
ATOM	4390	CA	PHE	A	550	35.324	13.409	126.488	1.00	0.80	C
ATOM	4391	C	PHE	A	550	35.690	14.870	126.180	1.00	0.80	C
ATOM	4392	O	PHE	A	550	36.543	15.113	125.323	1.00	0.80	O
ATOM	4393	CB	PHE	A	550	34.051	12.948	125.742	1.00	0.80	C
ATOM	4394	CG	PHE	A	550	34.185	13.138	124.263	1.00	0.80	C
ATOM	4395	CD1	PHE	A	550	34.909	12.256	123.457	1.00	0.80	C
ATOM	4396	CD2	PHE	A	550	33.704	14.318	123.693	1.00	0.80	C
ATOM	4397	CE1	PHE	A	550	35.108	12.517	122.099	1.00	0.80	C
ATOM	4398	CE2	PHE	A	550	33.971	14.611	122.353	1.00	0.80	C
ATOM	4399	CZ	PHE	A	550	34.631	13.695	121.532	1.00	0.80	C
ATOM	4400	N	ALA	A	551	35.099	15.886	126.854	1.00	0.80	N
ATOM	4401	CA	ALA	A	551	35.471	17.279	126.640	1.00	0.80	C
ATOM	4402	C	ALA	A	551	36.926	17.548	126.981	1.00	0.80	C
ATOM	4403	O	ALA	A	551	37.671	18.137	126.201	1.00	0.80	O
ATOM	4404	CB	ALA	A	551	34.570	18.207	127.475	1.00	0.80	C
ATOM	4405	N	ALA	A	552	37.382	16.997	128.121	1.00	0.85	N
ATOM	4406	CA	ALA	A	552	38.756	17.088	128.550	1.00	0.85	C
ATOM	4407	C	ALA	A	552	39.749	16.496	127.551	1.00	0.85	C
ATOM	4408	O	ALA	A	552	40.794	17.076	127.276	1.00	0.85	O
ATOM	4409	CB	ALA	A	552	38.906	16.336	129.883	1.00	0.85	C
ATOM	4410	N	PHE	A	553	39.416	15.329	126.966	1.00	0.84	N
ATOM	4411	CA	PHE	A	553	40.176	14.643	125.939	1.00	0.84	C
ATOM	4412	C	PHE	A	553	40.335	15.447	124.641	1.00	0.84	C
ATOM	4413	O	PHE	A	553	41.429	15.528	124.079	1.00	0.84	O
ATOM	4414	CB	PHE	A	553	39.456	13.286	125.707	1.00	0.84	C
ATOM	4415	CG	PHE	A	553	39.783	12.574	124.420	1.00	0.84	C
ATOM	4416	CD1	PHE	A	553	41.065	12.069	124.184	1.00	0.84	C
ATOM	4417	CD2	PHE	A	553	38.816	12.459	123.410	1.00	0.84	C
ATOM	4418	CE1	PHE	A	553	41.369	11.432	122.976	1.00	0.84	C
ATOM	4419	CE2	PHE	A	553	39.103	11.780	122.220	1.00	0.84	C

```
ATOM   4420  CZ  PHE A 553      40.377  11.245 122.009  1.00  0.84           C
ATOM   4421  N   VAL A 554      39.243  16.069 124.143  1.00  0.84           N
ATOM   4422  CA  VAL A 554      39.285  16.946 122.974  1.00  0.84           C
ATOM   4423  C   VAL A 554      40.098  18.212 123.247  1.00  0.84           C
ATOM   4424  O   VAL A 554      41.000  18.544 122.477  1.00  0.84           O
ATOM   4425  CB  VAL A 554      37.895  17.292 122.420  1.00  0.84           C
ATOM   4426  CG1 VAL A 554      38.008  18.193 121.169  1.00  0.84           C
ATOM   4427  CG2 VAL A 554      37.152  15.997 122.037  1.00  0.84           C
ATOM   4428  N   ASP A 555      39.868  18.905 124.383  1.00  0.79           N
ATOM   4429  CA  ASP A 555      40.586  20.108 124.785  1.00  0.79           C
ATOM   4430  C   ASP A 555      42.093  19.888 124.956  1.00  0.79           C
ATOM   4431  O   ASP A 555      42.942  20.662 124.509  1.00  0.79           O
ATOM   4432  CB  ASP A 555      40.001  20.589 126.135  1.00  0.79           C
ATOM   4433  CG  ASP A 555      38.657  21.302 126.001  1.00  0.79           C
ATOM   4434  OD1 ASP A 555      38.063  21.582 127.074  1.00  0.79           O
ATOM   4435  OD2 ASP A 555      38.220  21.587 124.855  1.00  0.79           O
ATOM   4436  N   LYS A 556      42.447  18.760 125.595  1.00  0.79           N
ATOM   4437  CA  LYS A 556      43.792  18.248 125.773  1.00  0.79           C
ATOM   4438  C   LYS A 556      44.535  18.017 124.477  1.00  0.79           C
ATOM   4439  O   LYS A 556      45.679  18.447 124.321  1.00  0.79           O
ATOM   4440  CB  LYS A 556      43.644  16.871 126.476  1.00  0.79           C
ATOM   4441  CG  LYS A 556      44.796  15.852 126.456  1.00  0.79           C
ATOM   4442  CD  LYS A 556      45.964  16.172 127.388  1.00  0.79           C
ATOM   4443  CE  LYS A 556      47.098  15.158 127.217  1.00  0.79           C
ATOM   4444  NZ  LYS A 556      48.323  15.689 127.847  1.00  0.79           N
ATOM   4445  N   CYS A 557      43.898  17.325 123.510  1.00  0.88           N
ATOM   4446  CA  CYS A 557      44.562  16.953 122.282  1.00  0.88           C
ATOM   4447  C   CYS A 557      44.440  17.971 121.178  1.00  0.88           C
ATOM   4448  O   CYS A 557      45.229  17.961 120.237  1.00  0.88           O
ATOM   4449  CB  CYS A 557      44.097  15.560 121.811  1.00  0.88           C
ATOM   4450  SG  CYS A 557      44.728  14.223 122.867  1.00  0.88           S
ATOM   4451  N   CYS A 558      43.524  18.947 121.291  1.00  0.87           N
ATOM   4452  CA  CYS A 558      43.484  20.069 120.374  1.00  0.87           C
ATOM   4453  C   CYS A 558      44.553  21.127 120.669  1.00  0.87           C
ATOM   4454  O   CYS A 558      44.847  21.934 119.778  1.00  0.87           O
ATOM   4455  CB  CYS A 558      42.070  20.711 120.244  1.00  0.87           C
ATOM   4456  SG  CYS A 558      40.813  19.656 119.439  1.00  0.87           S
ATOM   4457  N   ALA A 559      45.187  21.096 121.874  1.00  0.88           N
ATOM   4458  CA  ALA A 559      46.208  22.035 122.314  1.00  0.88           C
ATOM   4459  C   ALA A 559      47.685  21.578 122.200  1.00  0.88           C
ATOM   4460  O   ALA A 559      48.592  22.405 122.236  1.00  0.88           O
ATOM   4461  CB  ALA A 559      45.955  22.344 123.806  1.00  0.88           C
ATOM   4462  N   ALA A 560      47.994  20.266 122.064  1.00  0.84           N
ATOM   4463  CA  ALA A 560      49.368  19.779 121.959  1.00  0.84           C
ATOM   4464  C   ALA A 560      50.085  20.059 120.618  1.00  0.84           C
ATOM   4465  O   ALA A 560      49.442  20.266 119.591  1.00  0.84           O
ATOM   4466  CB  ALA A 560      49.416  18.273 122.312  1.00  0.84           C
ATOM   4467  N   ASP A 561      51.451  20.046 120.612  1.00  0.80           N
ATOM   4468  CA  ASP A 561      52.336  20.250 119.465  1.00  0.80           C
ATOM   4469  C   ASP A 561      52.153  19.199 118.358  1.00  0.80           C
ATOM   4470  O   ASP A 561      52.249  19.486 117.168  1.00  0.80           O
ATOM   4471  CB  ASP A 561      53.787  20.352 120.023  1.00  0.80           C
ATOM   4472  CG  ASP A 561      54.774  20.936 119.015  1.00  0.80           C
ATOM   4473  OD1 ASP A 561      54.637  22.149 118.715  1.00  0.80           O
ATOM   4474  OD2 ASP A 561      55.696  20.193 118.596  1.00  0.80           O
ATOM   4475  N   ASP A 562      51.811  17.958 118.760  1.00  0.82           N
ATOM   4476  CA  ASP A 562      51.525  16.856 117.875  1.00  0.82           C
ATOM   4477  C   ASP A 562      50.154  16.367 118.354  1.00  0.82           C
ATOM   4478  O   ASP A 562      49.992  15.787 119.435  1.00  0.82           O
ATOM   4479  CB  ASP A 562      52.718  15.855 117.966  1.00  0.82           C
ATOM   4480  CG  ASP A 562      52.562  14.530 117.228  1.00  0.82           C
ATOM   4481  OD1 ASP A 562      51.424  14.180 116.817  1.00  0.82           O
ATOM   4482  OD2 ASP A 562      53.590  13.807 117.168  1.00  0.82           O
ATOM   4483  N   LYS A 563      49.091  16.708 117.585  1.00  0.76           N
ATOM   4484  CA  LYS A 563      47.719  16.366 117.916  1.00  0.76           C
ATOM   4485  C   LYS A 563      47.443  14.915 117.598  1.00  0.76           C
ATOM   4486  O   LYS A 563      46.764  14.208 118.342  1.00  0.76           O
ATOM   4487  CB  LYS A 563      46.690  17.269 117.192  1.00  0.76           C
ATOM   4488  CG  LYS A 563      46.815  18.749 117.591  1.00  0.76           C
ATOM   4489  CD  LYS A 563      47.026  19.652 116.371  1.00  0.76           C
ATOM   4490  CE  LYS A 563      45.727  20.115 115.719  1.00  0.76           C
ATOM   4491  NZ  LYS A 563      45.231  21.318 116.427  1.00  0.76           N
ATOM   4492  N   GLU A 564      47.986  14.433 116.474  1.00  0.81           N
ATOM   4493  CA  GLU A 564      47.847  13.089 115.968  1.00  0.81           C
ATOM   4494  C   GLU A 564      48.389  12.015 116.914  1.00  0.81           C
ATOM   4495  O   GLU A 564      47.727  11.013 117.185  1.00  0.81           O
```

```
ATOM   4496  CB  GLU A 564      48.548  12.988 114.592  1.00  0.81           C
ATOM   4497  CG  GLU A 564      48.143  14.060 113.534  1.00  0.81           C
ATOM   4498  CD  GLU A 564      48.916  15.387 113.576  1.00  0.81           C
ATOM   4499  OE1 GLU A 564      48.555  16.235 114.439  1.00  0.81           O
ATOM   4500  OE2 GLU A 564      49.790  15.589 112.700  1.00  0.81           O
ATOM   4501  N   ALA A 565      49.588  12.237 117.493  1.00  0.89           N
ATOM   4502  CA  ALA A 565      50.140  11.444 118.577  1.00  0.89           C
ATOM   4503  C   ALA A 565      49.309  11.515 119.858  1.00  0.89           C
ATOM   4504  O   ALA A 565      49.126  10.511 120.551  1.00  0.89           O
ATOM   4505  CB  ALA A 565      51.604  11.833 118.867  1.00  0.89           C
ATOM   4506  N   CYS A 566      48.753  12.704 120.193  1.00  0.88           N
ATOM   4507  CA  CYS A 566      47.833  12.868 121.313  1.00  0.88           C
ATOM   4508  C   CYS A 566      46.542  12.050 121.174  1.00  0.88           C
ATOM   4509  O   CYS A 566      46.194  11.279 122.067  1.00  0.88           O
ATOM   4510  CB  CYS A 566      47.515  14.365 121.569  1.00  0.88           C
ATOM   4511  SG  CYS A 566      46.691  14.690 123.165  1.00  0.88           S
ATOM   4512  N   PHE A 567      45.832  12.136 120.020  1.00  0.84           N
ATOM   4513  CA  PHE A 567      44.615  11.361 119.767  1.00  0.84           C
ATOM   4514  C   PHE A 567      44.886   9.862 119.745  1.00  0.84           C
ATOM   4515  O   PHE A 567      44.094   9.064 120.250  1.00  0.84           O
ATOM   4516  CB  PHE A 567      43.840  11.745 118.470  1.00  0.84           C
ATOM   4517  CG  PHE A 567      43.057  13.019 118.619  1.00  0.84           C
ATOM   4518  CD1 PHE A 567      41.788  13.010 119.222  1.00  0.84           C
ATOM   4519  CD2 PHE A 567      43.556  14.232 118.131  1.00  0.84           C
ATOM   4520  CE1 PHE A 567      41.059  14.195 119.381  1.00  0.84           C
ATOM   4521  CE2 PHE A 567      42.853  15.426 118.325  1.00  0.84           C
ATOM   4522  CZ  PHE A 567      41.600  15.408 118.945  1.00  0.84           C
ATOM   4523  N   ALA A 568      46.043   9.460 119.180  1.00  0.85           N
ATOM   4524  CA  ALA A 568      46.491   8.087 119.117  1.00  0.85           C
ATOM   4525  C   ALA A 568      46.661   7.405 120.472  1.00  0.85           C
ATOM   4526  O   ALA A 568      46.243   6.265 120.660  1.00  0.85           O
ATOM   4527  CB  ALA A 568      47.856   8.021 118.402  1.00  0.85           C
ATOM   4528  N   LEU A 569      47.286   8.093 121.448  1.00  0.85           N
ATOM   4529  CA  LEU A 569      47.462   7.577 122.791  1.00  0.85           C
ATOM   4530  C   LEU A 569      46.269   7.759 123.716  1.00  0.85           C
ATOM   4531  O   LEU A 569      45.897   6.835 124.443  1.00  0.85           O
ATOM   4532  CB  LEU A 569      48.713   8.200 123.443  1.00  0.85           C
ATOM   4533  CG  LEU A 569      50.042   7.748 122.803  1.00  0.85           C
ATOM   4534  CD1 LEU A 569      51.213   8.568 123.364  1.00  0.85           C
ATOM   4535  CD2 LEU A 569      50.311   6.242 122.982  1.00  0.85           C
ATOM   4536  N   GLU A 570      45.633   8.948 123.734  1.00  0.81           N
ATOM   4537  CA  GLU A 570      44.556   9.220 124.673  1.00  0.81           C
ATOM   4538  C   GLU A 570      43.190   8.623 124.262  1.00  0.81           C
ATOM   4539  O   GLU A 570      42.331   8.325 125.094  1.00  0.81           O
ATOM   4540  CB  GLU A 570      44.406  10.742 124.934  1.00  0.81           C
ATOM   4541  CG  GLU A 570      45.666  11.527 125.413  1.00  0.81           C
ATOM   4542  CD  GLU A 570      46.282  11.017 126.721  1.00  0.81           C
ATOM   4543  OE1 GLU A 570      45.545  10.897 127.732  1.00  0.81           O
ATOM   4544  OE2 GLU A 570      47.517  10.768 126.751  1.00  0.81           O
ATOM   4545  N   GLY A 571      42.957   8.395 122.949  1.00  0.85           N
ATOM   4546  CA  GLY A 571      41.758   7.756 122.389  1.00  0.85           C
ATOM   4547  C   GLY A 571      41.428   6.381 122.916  1.00  0.85           C
ATOM   4548  O   GLY A 571      40.317   6.174 123.406  1.00  0.85           O
ATOM   4549  N   PRO A 572      42.331   5.409 122.863  1.00  0.81           N
ATOM   4550  CA  PRO A 572      42.181   4.141 123.565  1.00  0.81           C
ATOM   4551  C   PRO A 572      41.929   4.247 125.072  1.00  0.81           C
ATOM   4552  O   PRO A 572      41.089   3.510 125.587  1.00  0.81           O
ATOM   4553  CB  PRO A 572      43.472   3.378 123.244  1.00  0.81           C
ATOM   4554  CG  PRO A 572      43.959   3.934 121.899  1.00  0.81           C
ATOM   4555  CD  PRO A 572      43.406   5.362 121.861  1.00  0.81           C
ATOM   4556  N   LYS A 573      42.621   5.156 125.800  1.00  0.78           N
ATOM   4557  CA  LYS A 573      42.463   5.396 127.232  1.00  0.78           C
ATOM   4558  C   LYS A 573      41.063   5.802 127.586  1.00  0.78           C
ATOM   4559  O   LYS A 573      40.497   5.374 128.590  1.00  0.78           O
ATOM   4560  CB  LYS A 573      43.410   6.521 127.719  1.00  0.78           C
ATOM   4561  CG  LYS A 573      44.767   5.995 128.192  1.00  0.78           C
ATOM   4562  CD  LYS A 573      45.823   7.093 128.404  1.00  0.78           C
ATOM   4563  CE  LYS A 573      47.229   6.497 128.428  1.00  0.78           C
ATOM   4564  NZ  LYS A 573      48.159   7.420 129.105  1.00  0.78           N
ATOM   4565  N   LEU A 574      40.479   6.641 126.722  1.00  0.82           N
ATOM   4566  CA  LEU A 574      39.092   7.005 126.832  1.00  0.82           C
ATOM   4567  C   LEU A 574      38.134   5.820 126.686  1.00  0.82           C
ATOM   4568  O   LEU A 574      37.267   5.571 127.523  1.00  0.82           O
ATOM   4569  CB  LEU A 574      38.739   8.039 125.751  1.00  0.82           C
ATOM   4570  CG  LEU A 574      37.556   8.902 126.188  1.00  0.82           C
ATOM   4571  CD1 LEU A 574      38.078  10.035 127.081  1.00  0.82           C
```

```
ATOM   4572  CD2 LEU A 574      36.777   9.425 124.982  1.00  0.82           C
ATOM   4573  N   VAL A 575      38.319   4.999 125.635  1.00  0.79           N
ATOM   4574  CA  VAL A 575      37.489   3.837 125.337  1.00  0.79           C
ATOM   4575  C   VAL A 575      37.486   2.785 126.439  1.00  0.79           C
ATOM   4576  O   VAL A 575      36.461   2.166 126.715  1.00  0.79           O
ATOM   4577  CB  VAL A 575      37.896   3.169 124.027  1.00  0.79           C
ATOM   4578  CG1 VAL A 575      37.133   1.849 123.766  1.00  0.79           C
ATOM   4579  CG2 VAL A 575      37.660   4.126 122.844  1.00  0.79           C
ATOM   4580  N   VAL A 576      38.655   2.550 127.080  1.00  0.78           N
ATOM   4581  CA  VAL A 576      38.770   1.691 128.253  1.00  0.78           C
ATOM   4582  C   VAL A 576      37.975   2.200 129.440  1.00  0.78           C
ATOM   4583  O   VAL A 576      37.102   1.495 129.943  1.00  0.78           O
ATOM   4584  CB  VAL A 576      40.213   1.497 128.712  1.00  0.78           C
ATOM   4585  CG1 VAL A 576      40.291   0.553 129.931  1.00  0.78           C
ATOM   4586  CG2 VAL A 576      41.046   0.860 127.590  1.00  0.78           C
ATOM   4587  N   LYS A 577      38.196   3.464 129.876  1.00  0.73           N
ATOM   4588  CA  LYS A 577      37.548   3.997 131.059  1.00  0.73           C
ATOM   4589  C   LYS A 577      36.068   4.146 130.858  1.00  0.73           C
ATOM   4590  O   LYS A 577      35.276   3.927 131.772  1.00  0.73           O
ATOM   4591  CB  LYS A 577      38.102   5.374 131.477  1.00  0.73           C
ATOM   4592  CG  LYS A 577      37.489   5.935 132.781  1.00  0.73           C
ATOM   4593  CD  LYS A 577      38.008   7.343 133.114  1.00  0.73           C
ATOM   4594  CE  LYS A 577      37.468   7.950 134.420  1.00  0.73           C
ATOM   4595  NZ  LYS A 577      36.023   8.215 134.304  1.00  0.73           N
ATOM   4596  N   THR A 578      35.654   4.518 129.631  1.00  0.80           N
ATOM   4597  CA  THR A 578      34.244   4.539 129.271  1.00  0.80           C
ATOM   4598  C   THR A 578      33.626   3.138 129.411  1.00  0.80           C
ATOM   4599  O   THR A 578      32.667   2.956 130.154  1.00  0.80           O
ATOM   4600  CB  THR A 578      34.021   5.093 127.855  1.00  0.80           C
ATOM   4601  OG1 THR A 578      34.538   6.403 127.707  1.00  0.80           O
ATOM   4602  CG2 THR A 578      32.536   5.246 127.501  1.00  0.80           C
ATOM   4603  N   ARG A 579      34.251   2.100 128.792  1.00  0.71           N
ATOM   4604  CA  ARG A 579      33.863   0.690 128.856  1.00  0.71           C
ATOM   4605  C   ARG A 579      33.778   0.147 130.268  1.00  0.71           C
ATOM   4606  O   ARG A 579      32.845  -0.584 130.574  1.00  0.71           O
ATOM   4607  CB  ARG A 579      34.819  -0.206 128.007  1.00  0.71           C
ATOM   4608  CG  ARG A 579      34.483  -1.718 127.967  1.00  0.71           C
ATOM   4609  CD  ARG A 579      35.517  -2.576 127.227  1.00  0.71           C
ATOM   4610  NE  ARG A 579      35.510  -2.161 125.785  1.00  0.71           N
ATOM   4611  CZ  ARG A 579      34.633  -2.572 124.858  1.00  0.71           C
ATOM   4612  NH1 ARG A 579      34.713  -2.065 123.633  1.00  0.71           N
ATOM   4613  NH2 ARG A 579      33.705  -3.488 125.115  1.00  0.71           N
ATOM   4614  N   GLU A 580      34.731   0.506 131.137  1.00  0.72           N
ATOM   4615  CA  GLU A 580      34.717   0.178 132.539  1.00  0.72           C
ATOM   4616  C   GLU A 580      33.640   0.863 133.371  1.00  0.72           C
ATOM   4617  O   GLU A 580      33.062   0.278 134.276  1.00  0.72           O
ATOM   4618  CB  GLU A 580      36.095   0.518 133.111  1.00  0.72           C
ATOM   4619  CG  GLU A 580      36.296   0.067 134.572  1.00  0.72           C
ATOM   4620  CD  GLU A 580      37.682   0.452 135.072  1.00  0.72           C
ATOM   4621  OE1 GLU A 580      38.456   1.045 134.272  1.00  0.72           O
ATOM   4622  OE2 GLU A 580      37.977   0.177 136.263  1.00  0.72           O
ATOM   4623  N   ALA A 581      33.354   2.155 133.117  1.00  0.79           N
ATOM   4624  CA  ALA A 581      32.291   2.878 133.791  1.00  0.79           C
ATOM   4625  C   ALA A 581      30.853   2.444 133.467  1.00  0.79           C
ATOM   4626  O   ALA A 581      29.943   2.568 134.289  1.00  0.79           O
ATOM   4627  CB  ALA A 581      32.459   4.366 133.453  1.00  0.79           C
ATOM   4628  N   ILE A 582      30.630   1.989 132.220  1.00  0.70           N
ATOM   4629  CA  ILE A 582      29.352   1.563 131.677  1.00  0.70           C
ATOM   4630  C   ILE A 582      29.154   0.045 131.506  1.00  0.70           C
ATOM   4631  O   ILE A 582      28.128  -0.382 130.960  1.00  0.70           O
ATOM   4632  CB  ILE A 582      29.186   2.160 130.303  1.00  0.70           C
ATOM   4633  CG1 ILE A 582      30.145   1.519 129.302  1.00  0.70           C
ATOM   4634  CG2 ILE A 582      29.183   3.716 130.389  1.00  0.70           C
ATOM   4635  CD1 ILE A 582      30.151   2.375 128.035  1.00  0.70           C
ATOM   4636  N   ALA A 583      30.122  -0.813 131.890  1.00  0.64           N
ATOM   4637  CA  ALA A 583      30.002  -2.244 131.671  1.00  0.64           C
ATOM   4638  C   ALA A 583      30.826  -3.124 132.645  1.00  0.64           C
ATOM   4639  O   ALA A 583      31.445  -2.574 133.587  1.00  0.64           O
ATOM   4640  CB  ALA A 583      30.416  -2.651 130.235  1.00  0.64           C
ATOM   4641  OXT ALA A 583      30.820  -4.371 132.435  1.00  0.64           O
TER    4642      ALA A 583
END
```
